# Global, regional, and national burden of cardiovascular disease attributable to high body mass index from 1990 to 2021 and projection to 2045

**DOI:** 10.3389/fendo.2025.1546176

**Published:** 2025-04-28

**Authors:** Hui Li, Lifang Liang, Zhenyu Song, Yongfeng Li

**Affiliations:** ^1^ GMU-GIBH Joint School of Life Sciences, The Guangdong-Hong Kong-Macao Joint Laboratory for Cell Fate Regulation and Diseases, Guangzhou Medical University, Guangzhou, China; ^2^ Guangxi Key Laboratory of Reproductive Health and Birth Defect Prevention, Maternal and Child Health Hospital of Guangxi Zhuang Autonomous Region, Nanning, China; ^3^ Laboratory Animal Center of Guangxi Medical University, Nanning, China; ^4^ School of Public Health, Guangxi Medical University, Nanning, China

**Keywords:** cardiovascular disease, high body mass index, epidemiology, global burden of disease, public health

## Abstract

**Background:**

High body mass index (HBMI) is strongly associated with cardiovascular disease (CVD), but the global burden of CVD attributable to HBMI remains poorly defined. This study aims to elucidate the current burden and temporal trends of CVD attributable to HBMI.

**Methods:**

We used data from the Global Burden of Disease Study (GBD) 2021 to estimate CVD deaths and disability-adjusted life years (DALYs) attributable to HBMI. Our analysis examines trends in deaths and DALYs by age, gender, and Socio-demographic Index (SDI) across global, regional, and national levels from 1990 to 2021. We used health inequality and decomposition analyses to quantify the influencing factors of disease burden and a Bayesian age-period-cohort (BAPC) model to predict the potential trend of HBMI on CVD burden.

**Results:**

In 2021, HBMI-related CVD resulted in approximately 1.9 million deaths and 45.43 million DALYs among urban and rural populations, with an age-standardized mortality rate (ASMR) of 22.77 (95% UI, 12.87-34.24) and an age-standardized disability rate (ASDR) of 529.00 (95% UI, 277.28-808.64) per 100,000 people. Over the study period, the overall CVD burden attributable to HBMI decreased significantly, while the burden of atrial fibrillation and flutter increased. The disease burden was closely tied to socioeconomic development and was unevenly distributed, with middle SDI regions experiencing a heavier burden. The highest burden was observed in individuals aged 84 and older, with a significant increase in the 20–44 age group. Decomposition analysis revealed that the increase in DALYs was driven by population growth. Projections from the BAPC model suggest that by 2045, global DALYs of CVD attributable to HBMI may continue to increase.

**Conclusions:**

This study provides a comprehensive epidemiological assessment of the CVD burden attributable to HBMI across various regions and populations, offering valuable insights for guiding policy and research efforts.

## 1 Introduction

Cardiovascular disease (CVD) consistently poses a major threat to public health. In 2019, CVD was the leading cause of death globally (1). The causes of CVD include unhealthy eating habits, physical inactivity, obesity, smoking, hypertension, hyperlipidemia, and diabetes (2). Among these, obesity and hypertension are the most common controllable factors and significantly impact CVD. Additionally, CVD can lead to complications such as heart failure and stroke (3), imposing a significant economic burden globally. Effective prevention and management of CVD are crucial and involve controlling risk factors, maintaining a healthy diet and lifestyle, regular check-ups, and early pharmacological intervention.

Obesity or being overweight is a lifestyle disease characterized by energy intake exceeding expenditure, reflecting a long-term positive energy balance (4). The development of this condition is a complex, multifactorial process involving genetic predisposition, environmental factors, socioeconomic background, and individual behavior. The interaction of these factors may trigger mild persistent inflammation, hormonal imbalance, and abnormal immune responses, ultimately leading to systemic metabolic disorders (5, 6). Obesity is linked to various diseases, including CVD, type 2 diabetes, certain cancers, and bone and joint disorders (7, 8), as well as chronic inflammation. Excess fat tissue releases pro-inflammatory factors, causing chronic low-grade inflammation and adversely affecting overall health (9, 10). The long-term management model of obesity is similar to that of chronic disease management.

Studies have identified high body mass index (HBMI), high systolic blood pressure, and smoking as the three major risk factors for the global burden of CVD in 2021 (11). However, these studies only reported the population-attributable proportions of deaths and disability-adjusted life years (DALYs) caused by these risk factors, without detailing the attributable burden of CVD. DALY refers to the total number of healthy life years lost from onset to death, including years lost due to premature death (Years of Life Lost, YLL) and years of healthy life lost due to disability (Years Lost due to Disability, YLD). It is a time-based indicator that comprehensively considers both the quantity and quality of life and reflects the impact of disease on health (12, 13). In 2021, the top four diseases contributing to DALYs due to HBMI were diabetes, ischemic heart disease, hypertensive heart disease, and stroke (14). These results highlight the rising rates of HBMI and its associated disease burden worldwide, emphasizing the urgent need for regular monitoring and surveillance. This study focuses on the burden of CVD attributable to HBMI, analyzing trends in deaths and DALYs by age, gender, and Socio-demographic Index (SDI) at global, regional, and national levels from 1990 to 2021. SDI is a composite indicator that incorporates per capita income, average education level, and fertility rate. It is used to assess the level of socio-demographic development in countries and regions (15). Additionally, the Bayesian Age-Period-Cohort (BAPC) model will be used to predict the potential trend of CVD burden attributable to HBMI from 2022 to 2045. The results will provide the most comprehensive epidemiological analysis to date of the CVD burden attributable to HBMI. Therefore, this study aims to provide epidemiological references for government officials to formulate CVD management policies and for researchers to select research directions. It is hoped that the research results will guide the prevention of CVD and inform decisions regarding HBMI control.

## 2 Materials and methods

### 2.1 Study data

To investigate the impact of HBMI on the burden of CVD, we used the 2021 GBD data from the Global Health Data Exchange GBD Results Tool (http://ghdx.healthdata.org/gbd-results-tool). GBD collaborators developed this tool to create the most comprehensive public epidemiological database for quantifying global health and disease trends (16, 17). It offers a comprehensive assessment of life expectancy, disability prevalence for 371 diseases and injuries, and age- and gender-specific mortality rates for 288 causes across 204 countries and territories. The detailed methodology of GBD 2021 and the comparative risk assessment for CVD and HBMI are described elsewhere (14, 18). The original data used in this article are available on the GBD 2021 website (https://www.healthdata.org/research-analysis/about-gbd), including: (1) the number and rate of DALYs and deaths from CVD at all ages attributable to HBMI in three gender groups (males, females, and both) from 1990 to 2021 at global, regional, and national levels; (2) the number and rate of age-standardized CVD DALYs and deaths attributable to HBMI in the three gender groups from 1990 to 2021 at global, regional, and national levels; (3) the SDIs of 204 countries and regions from 1990 to 2021; (4) forecast of global population changes from 2022 to 2045.

### 2.2 Definitions

This study aimed to describe the combined trends in the disease burden of total CVD and six specific types (ischemic heart disease, stroke, hypertensive heart disease, atrial fibrillation and flutter, aortic aneurysm, and lower extremity peripheral arterial disease) attributable to HBMI. Detailed International Classification of Disease (ICD) codes for CVD-related causes of death are listed in the Appendix (**Supplementary Table S1**), and standardized case definitions for CVD are available in a recent publication (18, 19). BMI is calculated by dividing a person’s weight in kilograms by the square of their height in meters (kg/m²), with a BMI over 25 kg/m² considered high for individuals aged 20 and older (11). DALYs, which combine years of life lost due to premature death and years lived with disability, were used to measure disease burden in this study. The GBD database calculates YLDs based on estimated age, gender, location, year-specific prevalence, and corresponding disability weights. We also focused on CVD mortality attributed to HBMI. The GBD research team estimated the burden of CVD, including 13 causes of death and 9 associated risk factors, using all available population-level data, including incidence, prevalence, mortality, and health risk factors. SDI is a composite indicator in the GBD used to measure a country’s socioeconomic development level and is closely related to health outcomes. It is calculated from the geometric mean of the total fertility rate for women under 25 (TFU25), the average years of education for those aged 15 and over (EDU15+), and per capita lagged distributed income (LDI) (20). SDI is typically divided into five levels, from low to high: low SDI, low-middle SDI, middle SDI, high-middle SDI, and high SDI.

### 2.3 Statistical analysis

Age-standardized rates (ASRs) are statistical tools used to assess health indicators such as mortality and DALY rates across countries, especially when age structures and demographic characteristics differ. The annual percentage change (EAPC) is estimated by converting the ratio to a natural logarithm and fitting a linear model with time (i.e., y = α + βx + ϵ, where x is the year and y is the natural logarithm of the ratio). EAPC is calculated using the formula 100(e^β-1), with a 95% confidence interval (CI) also calculated. Specifically, if both the EAPC value and the lower limit of its 95% CI are greater than 0, the ASR is considered to be trending upward. Conversely, if both the EAPC value and the upper limit of its 95% CI are below 0, the ASR is considered to be trending downward. If the EAPC value fluctuates around 0, the ASR is considered relatively stable during the period under consideration (21, 22). To analyze the relationship between ASR and SDI, we used the Gaussian process regression model combined with a Loess smoother for visualization, and evaluated the correlation using the Spearman/Pearson rank correlation test (23). Additionally, a decomposition analysis quantifies the contributions of three population-level factors—epidemiological changes, population aging, and population growth—to the total change in DALYs from 1990 to 2021. The specific method of this decomposition analysis is described in the literature (24). This approach provides a deeper understanding of how demographic factors affect changes in health indicators. We used the Pearson correlation coefficient to measure the relationship between SDI and DALYs of CVD burden attributable to the age-standardized HBMI index. The slope index and concentration index reflect the absolute and relative extents of health inequality, respectively.

To assess future trends of CVD burden attributable to HBMI, we used the integrated nested Laplace approximation (INLA) framework combined with the BAPC model for prediction. This combined method approximately estimates the marginal posterior distribution and effectively avoids the mixing and convergence problems encountered by traditional Bayesian methods that rely on Markov chain Monte Carlo (MCMC) sampling. The BAPC model, based on the Bayesian framework integrates prior knowledge with sample data to effectively separate and quantify age, period, and cohort effects. It also incorporates data uncertainty into the parameter estimation process, providing a statistical basis for evaluating the reliability of prediction results (25, 26). The BAPC model relies on GBD data from 1990 to 2021 and population forecast data from the World Health Organization, providing an accurate basis for predicting future trends (27, 28). We used the “BAPC” package (version 0.0.36) and the “INLA” package (version 24.06.27) in the R environment for analysis. We used R version 4.4.0 and Stata version 16.0 for statistical analyses and data visualization. In statistical analyses, a P value less than 0.05 indicated statistical significance. An effect was considered statistically significant if its 95% uncertainty interval (UI) did not include zero. Additionally, all rates are reported per 100,000 population.

## 3 Result

### 3.1 Spatiotemporal pattern of CVD burden attributable to HBMI

**Figure 1:**
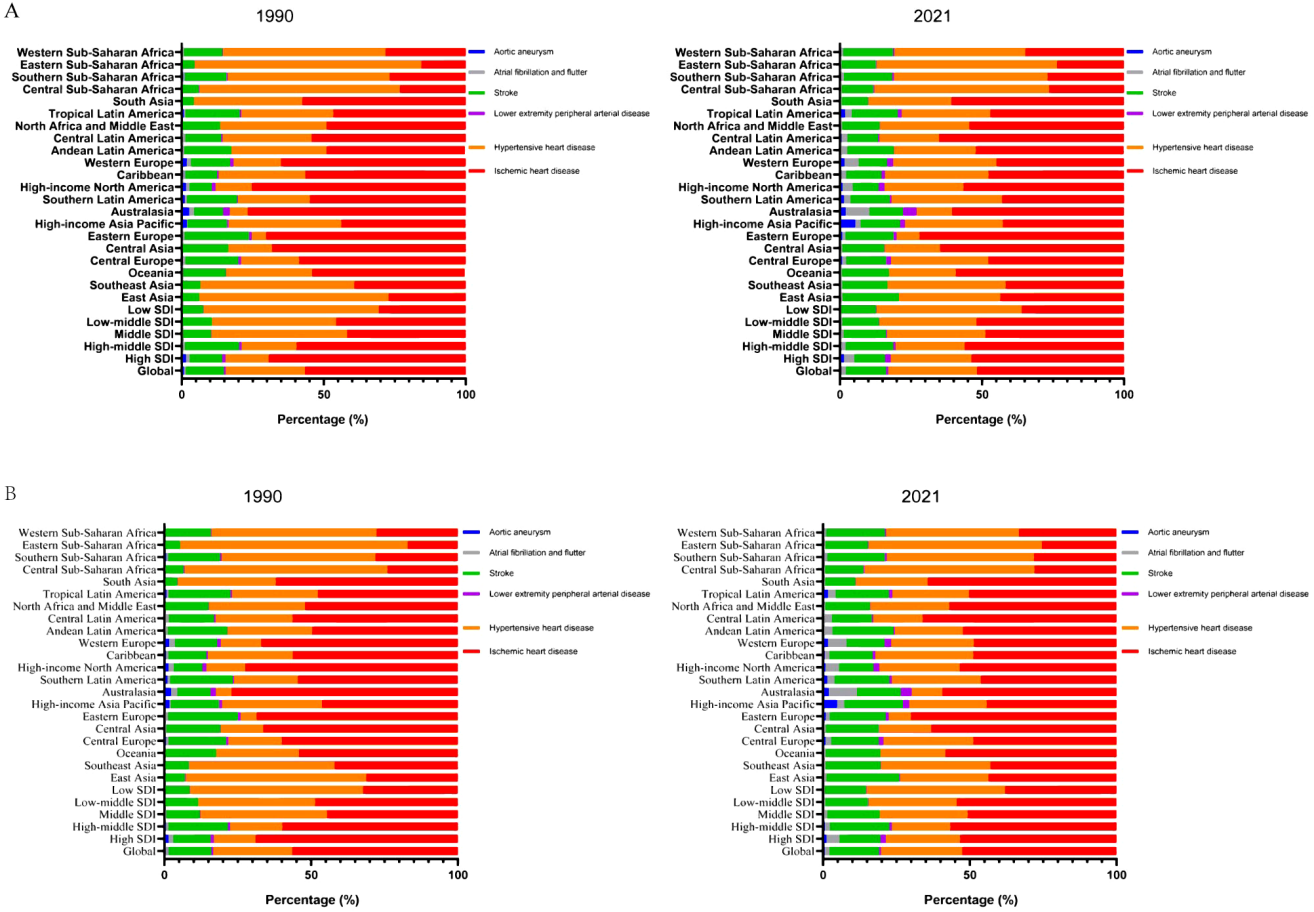
Contribution of the HBMI-attributable CVD burden in different locations. Contribution of the HBMI-attributable CVD deaths **(A)** and DALYs **(B)** for both genders, globally and by region, in 1990 and 2021. HBMI, high body mass index; CVD, cardiovascular disease; DALYs, disability-adjusted life years.

In 2021, CVD attributable to HBMI caused approximately 1.9 million deaths and 45.43 million DALYs among urban and rural residents, with an age-standardized mortality rate (ASMR) of 22.77 (95% UI, 12.87-34.24) and an age-standardized disability rate (ASDR) of 529.00 (95% UI, 277.28-808.64) per 100,000 population. Over the past 30 years, the burden of CVD attributable to HBMI has declined significantly, with the greatest reduction observed in aortic aneurysm (**Supplementary Tables S2, S3**). However, HBMI significantly increased the burden of atrial fibrillation and flutter (**Supplementary Tables S2, S3**). Compared to 1990, the primary contributors to CVD burden attributable to HBMI in 2021 were ischemic heart disease, hypertensive heart disease, and stroke (**Figure 1**).

In SDI regions, HBMI significantly reduced the CVD burden in high and high-middle SDI regions, while substantially increasing it in middle, low-middle, and low SDI regions (**Supplementary Tables S2, S3**, **Supplementary Figures S1, S2**). Over the past 30 years, the burden of aortic aneurysms attributable to HBMI has significantly decreased in high SDI regions, while the burden of atrial fibrillation and flutter have significantly increased. In high-middle SDI regions, the burden of ischemic heart disease attributable to HBMI significantly decreased, while the burden of atrial fibrillation and flutter significantly increased. Notably, the burden of atrial fibrillation and flutter attributable to HBMI significantly increased in middle, low-middle, and low SDI regions. Overall, the burden of atrial fibrillation and flutter attributable to HBMI significantly increased in all SDI regions (**Supplementary Tables S2, S3**, **Supplementary Figures S1, S2**).

Regionally, HBMI accounted for a larger proportion of CVD deaths and DALYs in economically less developed areas, such as North Africa, the Middle East, Central Asia, Eastern Europe, and Southern Sub-Saharan Africa. Ischemic heart disease was the primary contributor to DALYs attributable to HBMI among GBD level 2 causes (**Supplementary Figure S3**). North Africa and the Middle East bear the highest CVD burden attributable to HBMI among all regions. In these areas, ischemic heart disease, followed by hypertensive heart disease, represents the highest burden attributable to HBMI. ASMR and ASDR for CVD attributable to HBMI decreased in most regions, with the largest decline in Australasia. The burden of atrial fibrillation and flutter attributable to HBMI significantly increased in both ASMR and ASDR across regions, particularly in East Asia, Southeast Asia, and South Asia (**Supplementary Tables S2, S3**, **Supplementary Figure S3**).

**Figure 2:**
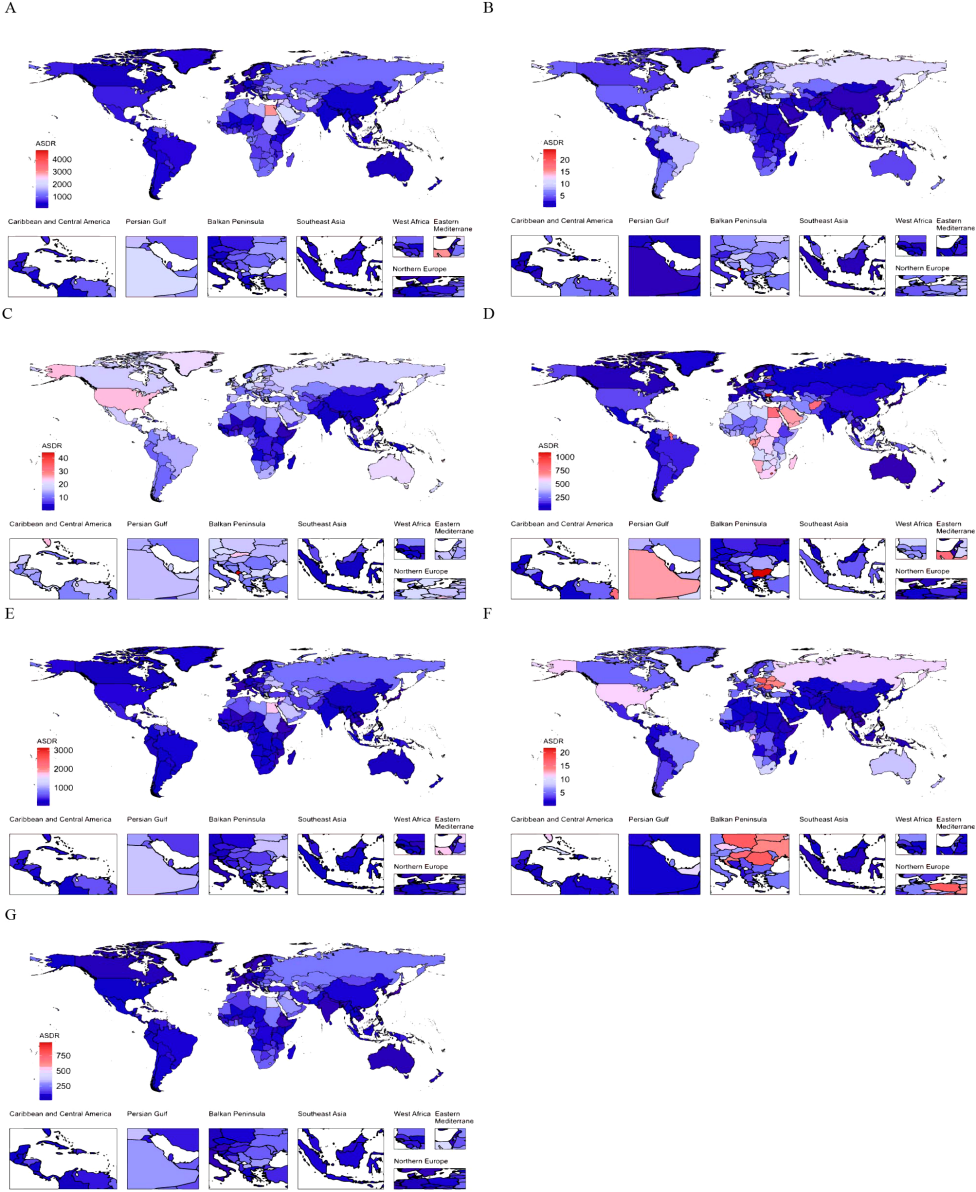
The global distribution of ASDR of CVD attributable to HBMI for both genders in 2021. ASDR of CVD **(A)**, aortic aneurysm **(B)**, atrial fibrillation and flutter **(C)**, hypertensive heart disease **(D)**, ischemic heart disease **(E)**, lower extremity peripheral arterial disease **(F)**, and stroke **(G)** attributable to HBMI for both genders in 204 countries and territories in 2021. ASDR, age-standardized DALYs (disability-adjusted life years) rate; CVD, cardiovascular disease; HBMI, high body mass index.

At the country level, the ASDR for CVD burden attributable to HBMI in 2021 varied widely globally, with Nauru and Egypt having the highest ASDRs (**Figure 2**). In 11 countries, the ASDR of CVD burden attributable to HBMI exceeded 2000/100,000, with Nauru having the highest ASDR (4730.31; 95% UI, 2013.73-7491.18). In 13 countries, the ASDR was below 200/100,000, with Japan having the lowest ASDR (81.64; 95% UI, 38.17-130.78). Among GBD level 2 causes, ischemic heart disease attributable to HBMI accounted for the highest proportion of ASDR in each country. For ischemic heart disease attributable to HBMI, 6 countries had ASDRs above 1500 per 100,000, with Nauru having the highest ASDR (3124.23; 95% UI, 1330.60-4921.45). Conversely, 22 countries had ASDRs below 100/100,000, with South Korea having the lowest ASDR (35.57; 95% UI, 13.05-58.82). Among GBD level 2 causes, aortic aneurysm and lower extremity peripheral arterial disease attributable to HBMI had the lowest ASDR proportion in all countries (**Figure 2**). Changes in the ASDR of CVD burden attributable to HBMI varied widely between countries from 1990 to 2021, with the largest increase in Zimbabwe and the largest decrease in Israel (**Supplementary Figure S4**, **Supplementary Tables S4**). Among GBD level 2 causes, the ASDR increase for atrial fibrillation and flutter attributable to HBMI was the largest. Specifically, the ASDR for atrial fibrillation and flutter attributable to HBMI increased in almost all countries except Finland, with the largest increase in Vietnam. The ASDR burden of hypertensive heart disease attributable to HBMI varied the most, with 43 countries experiencing a significant increase and 40 a significant decrease. The largest increase was in Latvia, and the largest decrease was in Belarus (**Supplementary Figure S4**, **Supplementary Table S4**). In summary, CVD burden attributable to HBMI exhibited geographically uneven distribution.

### 3.2 Age and gender patterns

**Figure 3:**
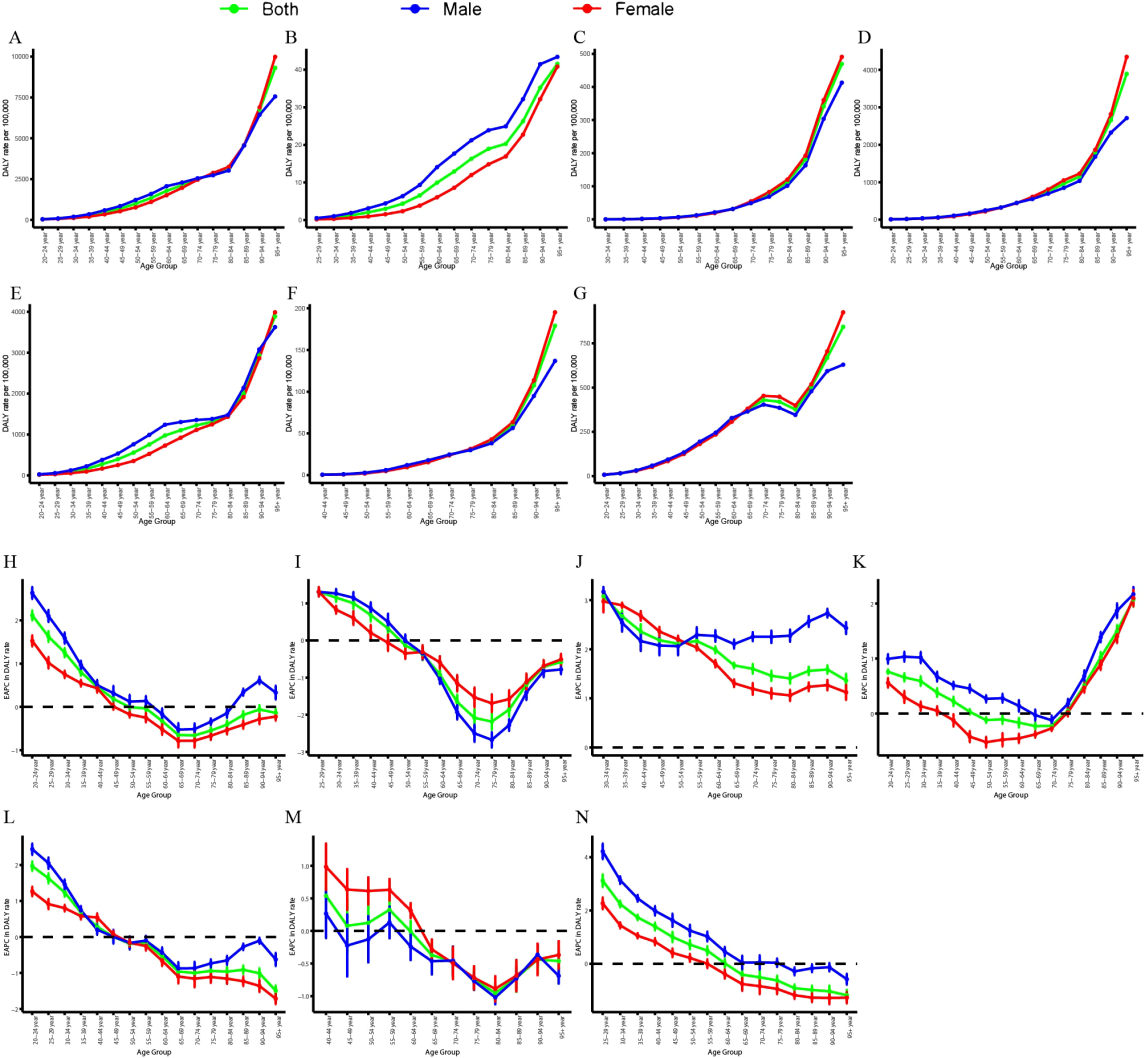
Age-specific rates of global DALYs of CVD attributable to HBMI, by gender, in 2021 and the corresponding EAPC from 1990 to 2021. Global DALY rates of CVD **(A)**, aortic aneurysm **(B)**, atrial fibrillation and flutter **(C)**, hypertensive heart disease **(D)**, ischemic heart disease **(E)**, lower extremity peripheral arterial disease **(F)**, and stroke **(G)** attributable to HBMI in different age groups, by gender, in 2021. EAPC of global DALY rates of CVD **(H)**, aortic aneurysm **(I)**, atrial fibrillation and flutter **(J)**, hypertensive heart disease **(K)**, ischemic heart disease **(L)**, lower extremity peripheral arterial disease **(M)**, and stroke **(N)** attributable to HBMI in different age groups, by gender, from 1990 to 2021. DALYs, disability-adjusted life years; CVD, cardiovascular disease; HBMI, high body mass index; EAPC, estimated annual percentage change.

**Figure 3** and **Supplementary Figure S5** illustrate the global age-specific DALYs and mortality rates of CVD attributable to HBMI in 2021, as well as the corresponding time trends from 1990 to 2021. DALYs in each age group exhibited a gradual upward trend, with a modest increase in the 20-84-year-old group and a pronounced increase in those aged 84 years and above (**Figure 3A**). Among GBD level 2 causes, the DALYs of the six CVDs also exhibited a gradual upward trend, with a marked increase in those aged over 84 years (**Figures 3B–G**). In the burden of aortic aneurysm attributable to HBMI, DALYs were consistently higher in males than in females (**Figure 3B**). In other CVDs, DALYs attributable to HBMI were comparable between males and females, but the DALY rate was higher in elderly females (**Figures 3C–G**). From 1990 to 2021, the mortality and DALY rates of CVD attributable to HBMI in different age groups exhibited varying trends. The mortality and DALY rates for individuals aged 20–44 increased significantly, whereas those for individuals aged 60–84 decreased (**Figure 3H**, **Supplementary Figure S5H**). Among GBD level 2 causes, the mortality and DALY rates of atrial fibrillation and flutter attributable to HBMI showed a significant upward trend across all age groups (**Figure 3J**, **Supplementary Figure S5J**). For other CVDs, the mortality and DALY rates increased significantly in the 20-44-year age group but decreased in other age groups (**Figures 3I, K–N**, **Supplementary Figure S5J, K–N**). However, for hypertensive heart disease, the mortality and DALY rates significantly increased in those aged over 80 years (**Figure 3K**, **Supplementary Figure S5K**). Among SDI regions, the ASMR in high, high-middle, and middle SDI regions exhibited an overall downward trend, with higher rates in males than in females. Conversely, the ASMR in low-middle and low SDI regions exhibited an overall upward trend, with higher rates in females than in males (**Supplementary Figure S6**). Collectively, the results highlighted the need for close attention to individuals aged 84 and above and those aged 20-44.

### 3.3 Association with the SDI

**Figure 4:**
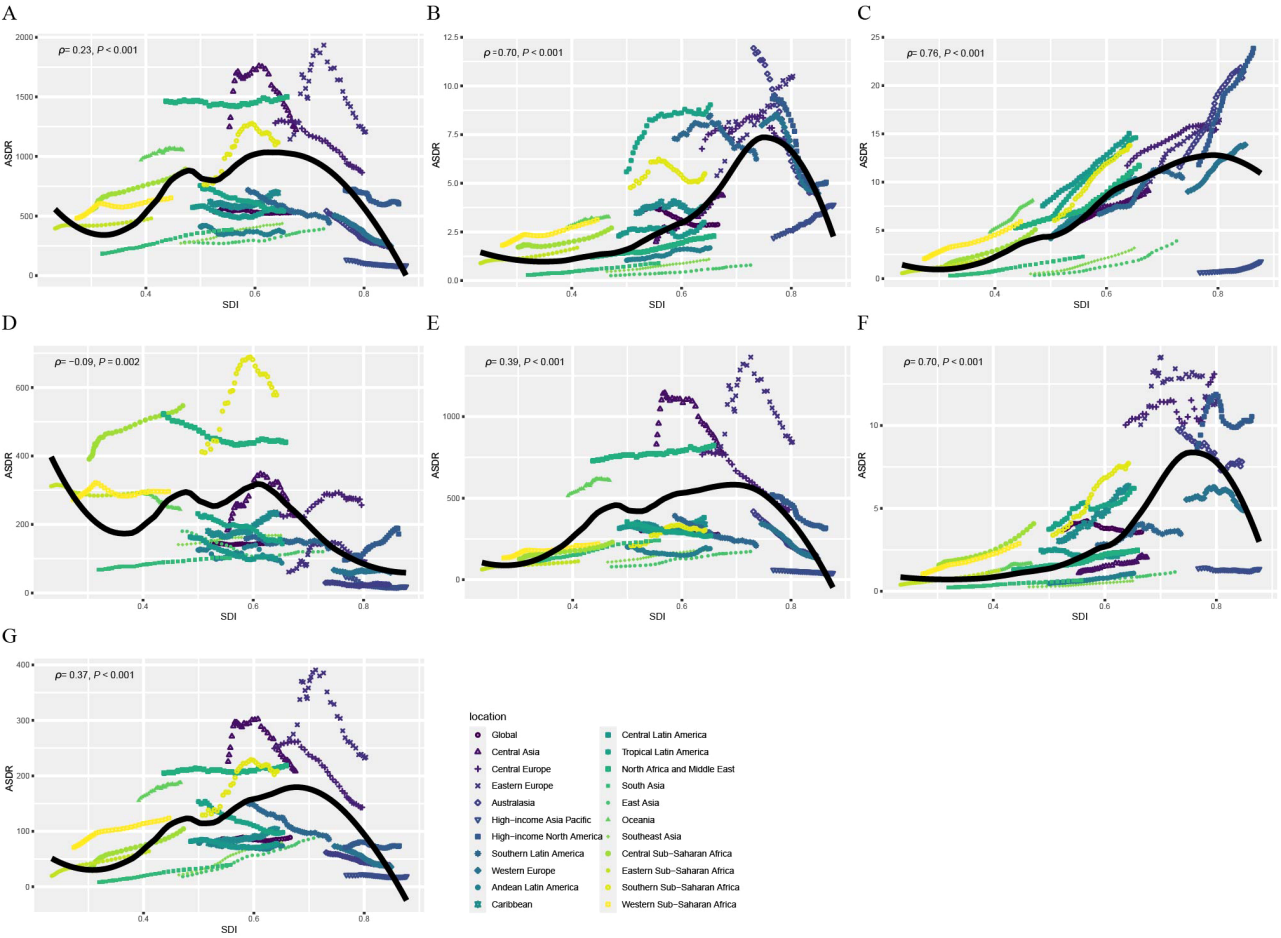
Correlations between ASDR of CVD attributable to HBMI and SDI at the regional level. ASDR of CVD **(A)**, aortic aneurysm **(B)**, atrial fibrillation and flutter **(C)**, hypertensive heart disease **(D)**, ischemic heart disease **(E)**, lower extremity peripheral arterial disease **(F)**, and stroke **(G)** attributable to HBMI at the global level and 21 regions, by SDI, 1990–2021. Black line represents the expected ASDR based on SDIs in all locations. ASDR, age standardized DALYs (disability-adjusted life years) rate; CVD, cardiovascular disease; HBMI, high body mass index; SDI, socio-demographic index.

**Figure 4** illustrates the observed and expected ASDRs for CVDs attributable to HBMI at the regional level based on SDI from 1990 to 2021. The ASDR is positively correlated with SDI, increasing as SDI rises until it reaches approximately 0.70. Globally, the observed ASDR was lower than expected (**Figure 4A**). However, in regions with middle SDI levels, such as Central Asia, Eastern Europe, North Africa, and the Middle East, the observed ASDR exceeded expectations. Among GBD level 2 causes, ASDRs for various CVDs attributable to HBMI, except hypertensive heart disease, increased with rising SDI values until reaching approximately 0.70 (**Figures 4B–G**). Regionally, observed ASMR patterns based on SDI were consistent with ASDR patterns (**Supplementary Figure S7**). **Supplementary Figures S8** and **S9** display the observed and expected ASDR and ASMR at the country level based on SDI values in 2021. At the country level, ASDR and ASMR of CVD attributable to HBMI was negatively correlated with SDI. Some countries, like Nauru, had ASDRs significantly higher than expected. These results collectively indicated that the disease burden was closely associated with socioeconomic development and was unevenly distributed.

### 3.4 Decomposition analysis of changes in DALYs

We conducted decomposition analyses to assess the contributions of three population-level determinants—population growth, population aging, and epidemiological changes—to the change in DALYs from CVD attributable to HBMI between 1990 and 2021 (**Supplementary Figure S10**, **Supplementary Table S6**). Based on ASDR trends, we found that population growth significantly influenced the overall change in DALYs of CVD burden attributable to HBMI across all SDI regions. Population aging significantly contributed to the increase in DALYs across all SDI regions. Epidemiological changes decreased DALYs in high-middle and middle SDI regions, but increased them in other SDI regions. Among GBD level 2 causes, the change in DALYs of the six CVDs attributable to HBMI is primarily driven by population growth. Currently, the increase in DALYs attributable to population growth offsets the decrease attributable to epidemiological changes, resulting in a net increase in DALYs of CVD attributable to HBMI. This suggested that the increase in DALYs was primarily driven by population growth.

### 3.5 Health inequality analysis

We further analyzed health inequities in changes in CVD DALYs attributable to HBMI (**Supplementary Figure S11**). The concentration index of CVD DALYs attributable to HBMI decreased from 0.14 (95% CI: 0.07, 0.20) in 1990 to -0.04 (95% CI: -0.09, 0.02) in 2021, while the slope index decreased from 371.64 (95% CI: 173.30, 570.00) to -160.84 (95% CI: -370.73, 49.04). Among GBD level 2 causes, the concentration index of six CVDs DALYs attributable to HBMI decreased. Collectively, these results suggested that while health inequalities in the DALYs of CVD attributable to HBMI had improved, disparities between SDI regions still required attention.

### 3.6 Prediction of CVD DALY caused by HBMI up to 2045

**Figure 5:**
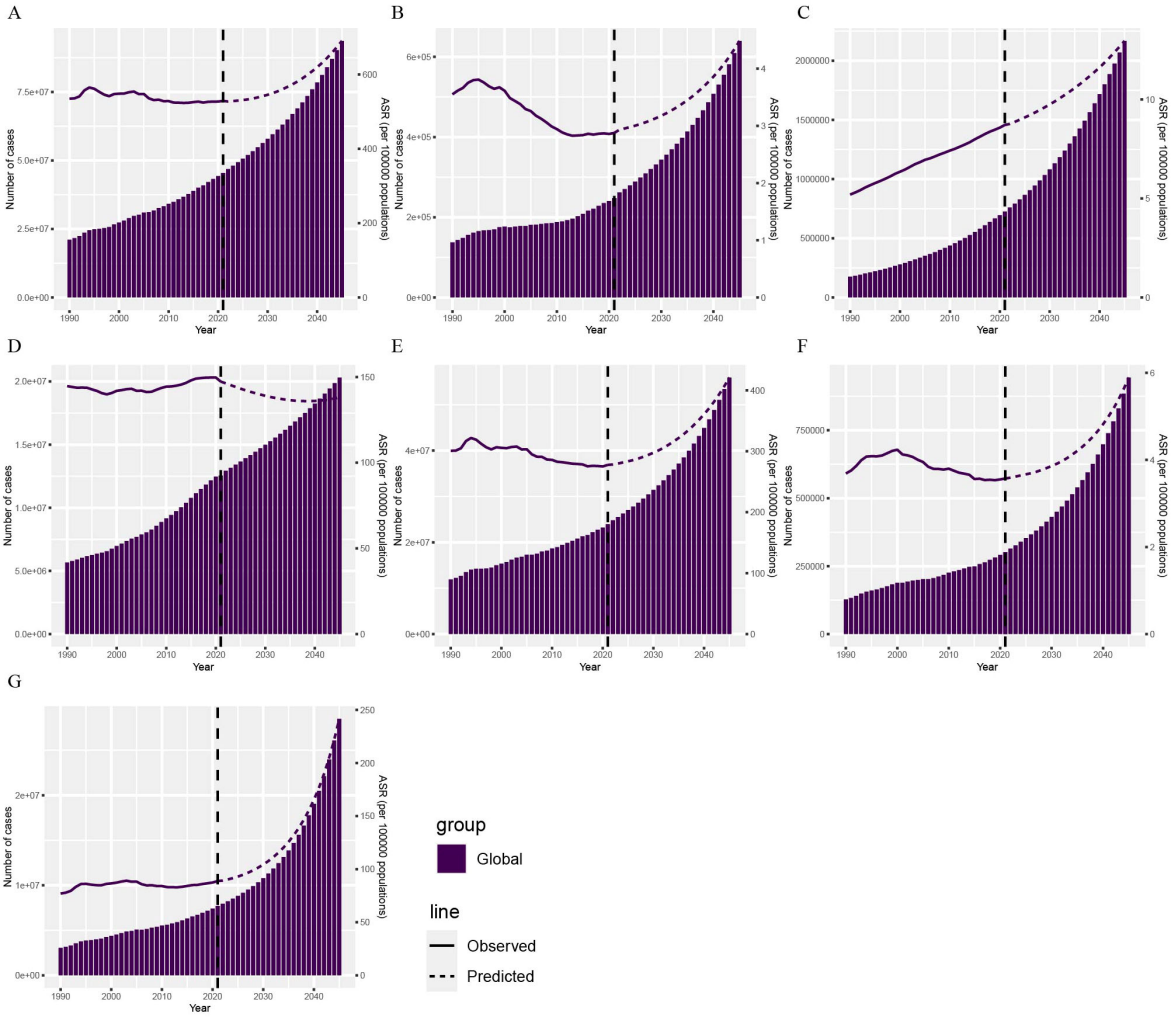
Projections of CVD DALYs by 2045 based on the BAPC model. The DALYs of CVD **(A)**, aortic aneurysm **(B)**, atrial fibrillation and flutter **(C)**, hypertensive heart disease **(D)**, ischemic heart disease **(E)**, lower extremity peripheral arterial disease **(F)**, and stroke **(G)** attributable to HBMI. BAPC, Bayesian age-period-cohort; DALYs, disability-adjusted life years; CVD, cardiovascular disease; HBMI, high body mass index.

This study combined predicted population changes from 2022 to 2045 with the BAPC model to forecast the DALYs of CVDs attributable to HBMI over the next 24 years (**Figure 5**). The solid line represents actual data from 1990 to 2021 from the GBD 2021 report, while the dashed line represents forecast data from 2022 to 2045 based on the INLA framework and BAPC method. Globally, CVD DALYs attributable to HBMI showed an overall increasing trend. Among GBD level 2 causes, the DALYs burden of various CVDs attributable to HBMI showed an overall upward trend, except hypertensive heart disease.

## 4 Discussion

Multiple studies have shown that HBMI is a significant predictor of CVD mortality (29–31). Additionally, HBMI may indirectly increase CVD risk by affecting factors such as blood pressure, lipids, and sugar. Previous GBD studies have generally focused on the impact of HBMI on global health or the global and regional burden of CVD (1, 14, 18, 32, 33). They reported the CVD burden attributable to risk factors but did not specifically analyze the burden attributable to HBMI. To fill this gap, our study first reported the number of CVD deaths and DALYs attributable to HBMI, comparing them across regions, countries, SDIs, genders, and age groups. The study highlighted the significant reduction in the burden of some CVDs, such as aortic aneurysm, and the increase in the burden of others, particularly atrial fibrillation and flutter. It emphasized the importance of DALY differences by age and gender. The higher DALY rates among older females and the significant increase in DALYs among individuals aged 20–44 highlighted the need to focus on HBMI in both elderly and younger populations. Concurrently, health inequality and decomposition analyses were employed to quantify the factors influencing the disease burden, and it was found that population growth was the primary driver of the increase in DALYs. Lastly, the BAPC model predicted an overall upward trend in CVD DALYs attributable to HBMI. The study conducted a comprehensive epidemiological assessment of the CVD burden attributable to HBMI across different regions and populations, filling gaps in early GBD research and offering valuable insights for policy and research.

The analysis showed that from 1990 to 2021, the CVD burden attributable to HBMI decreased significantly, with the greatest reduction in aortic aneurysm burden, while the burden of atrial fibrillation and flutter still increased significantly. These differences highlight the need for personalized public health strategies for different CVDs to address the increasing global burden attributable to HBMI. Additionally, analyses at the SDI and regional levels found that CVD burden attributable to HBMI increased significantly in economically less developed areas, indicating a stronger association between HBMI and CVD burden in these regions. Ezzati et al. showed that with global economic development, CVD and its nutrition-related risk factors (e.g., overweight, obesity, hypertension, high cholesterol) have become leading causes of death and morbidity worldwide and are expected to increase with further economic development (34). This further emphasizes HBMI’s impact on CVD burden in economically less developed regions, where increased HBMI is associated with multiple socioeconomic factors. First, rapid urbanization and a widening wealth gap have affected women’s ability to control their weight during urbanization (35). Furthermore, higher socioeconomic status (SES) at the individual and household levels in urban areas was positively associated with higher BMI (36). Additionally, higher education levels are associated with increased BMI. A study in Andhra Pradesh, India, found that with improved socioeconomic status and decreased physical activity were associated with increased average BMI (37).

Our study found that DALYs of CVD attributable to HBMI increased with age, particularly sharply in individuals over 84 years old. As people age, their muscle mass decreases and visceral fat accumulates, leading to insulin resistance, chronic inflammation, and lipid metabolism disorders (e.g., increased low-density lipoprotein and decreased high-density lipoprotein) (38, 39). HBMI indirectly increases the risk of ischemic heart disease and stroke by promoting pathological processes such as hypertension, hyperglycemia, and atherosclerosis (38, 40). Abnormal T cell metabolic pathways (e.g., mTOR and AMPK signaling pathways) in the elderly population exacerbate the release of inflammatory factors (e.g., IL-6 and TNF-α), promoting vascular endothelial damage and plaque formation (41). Additionally, mitochondrial dysfunction and oxidative stress associated with aging further amplify the metabolic toxicity of HBMI (40). Reduced activity, a more sedentary lifestyle, and a high-calorie, high-fat diet increase the risk of HBMI and CVD in the elderly (42, 43). Although smoking rates are lower among the elderly, the cumulative effects of long-term smoking and drinking still pose a threat to cardiovascular health (43). We also found that the DALY rate in older females was higher than in males. Following menopause, females experience a sharp decline in estrogen levels, which triggers disorders in lipid metabolism and vascular endothelial dysfunction, thereby accelerating atherosclerosis progression (44, 45). The attenuation of estrogen’s cardioprotective effects significantly elevates HBMI-associated inflammatory responses and insulin resistance. Notably, although females exhibit a predisposition for subcutaneous fat accumulation (e.g., abdominal), estrogen deficiency promotes visceral adipose redistribution which is a key driver of CVD risk (46, 47). From 1990 to 2021, mortality and DALY rates for those aged 20 to 44 increased significantly, while rates for other age groups showed a downward trend. Studies show that obesity can accelerate coronary atherosclerosis progression in young people, especially young men (48). This suggests that obesity’s negative effects on CVD are more direct and rapid in younger people. Furthermore, studies show that high childhood BMI is significantly associated with all-cause and cardiovascular mortality in adulthood (49). This means that early obesity may lay the foundation for long-term cardiovascular issues, increasing adult CVD risk.

When investigating the association between CVD burden attributable to HBMI and SDI, we found that the burden was greater in low-middle, middle, and high-middle SDI regions. Previous evidence is consistent with our findings (50), indicating that the CVD burden attributable to HBMI has increased in middle-income regions and countries over the past 30 years. In low-middle SDI regions, residents may have less access to healthy lifestyles and quality health care because of lower economic development. In resource-limited settings, people may consume more high-calorie, high-fat foods and lack exercise to maintain a healthy weight (51). Obesity can induce chronic low-grade inflammation, further impair endothelial function, and increase the risk of atherosclerosis (52, 53). HBMI directly leads to metabolic disorders such as insulin resistance, hypertension, and hyperlipidemia, which are major risk factors for CVD (52–54). Additionally, residents in low-middle SDI regions may face more socioeconomic stress, such as poverty, unemployment, and poor housing. They face greater psychosocial stress, which may increase CVD risk through neuroendocrine mechanisms (55). In terms of health care, residents in low-middle SDI regions may lack access to adequate services, including those for CVD prevention and treatment. This may lead to poor disease management and increasing CVD risk and burden (56), further exacerbating health inequalities. In high SDI regions, the CVD burden attributable to HBMI showed a downward trend due to improved medical interventions and living conditions, suggesting that the impact of HBMI on public health may have been alleviated in these regions. Comprehensive measures are needed to reduce the burden of CVD and promote health equity, including improving the allocation of medical resources, promoting healthy lifestyles, and strengthening policy support.

Decomposition analysis found that changes in DALYs of CVD burden attributable to HBMI were primarily driven by population growth and aging. As the global population grows, the overall CVD burden attributable to HBMI also rises. For example, from 1990 to 2019, CVD deaths and DALYs attributable to HBMI nearly doubled worldwide (50). This growth trend is particularly evident in low SDI regions (57). Additionally, studies in China and other countries show that as populations grow, the burden of HBMI-related diseases also increases (54, 58). In summary, population growth directly leads to more people facing HBMI risk, while aging makes more elderly individuals a high-risk group for HBMI-related diseases. Additionally, over the past three decades, health inequality in DALYs of CVD attributable to HBMI has slightly decreased and remained relatively stable, indicating a consistent gap between rich and poor. According to the BAPC model, the CVD burden attributable to HBMI may increase from 2022 to 2045. This suggests that to effectively reduce the CVD burden attributable to HBMI and promote health equity, interventions targeting these groups need to be strengthening.

However, this study has some limitations. First, the GBD database relies on health data from various countries, but many, especially low- and middle-income countries, may have underreporting or limited health surveillance systems. This may lead to underestimation or overestimation of the disease burden, affect the capture of disease dynamics, and thus impact the representativeness of research results. Therefore, low- and middle-income countries must establish more complete data collection and reporting mechanisms in their healthcare systems. Second, various statistical methods are used to reduce GBD data collection bias, including statistical imputation and covariate adjustment. Statistical attribution methods reduce bias by integrating data from multiple sources, such as epidemiological surveys and hospital records (59). However, the accuracy of statistical attribution depends heavily on data quality and model assumptions. If the data are biased, the attribution results may still deviate from reality. Concurrently, researchers adjust covariates such as age, gender, and socioeconomic status to more accurately estimate the independent effects of exposure factors (60). Nevertheless, the effectiveness of covariate adjustment depends on whether all relevant confounding variables are included in the model. If important variables are omitted, the adjusted results may still be biased. Although the GBD data collectors used rigorous statistical methods to address these biases, they may still overestimate or underestimate differences in disease burden, leading to inaccurate interpretations of regional differences. In conclusion, the study results should be considered the best estimates based on current evidence. Finally, the definition of HBMI may vary by region, culture, and study. In some studies, different BMI cutoff values may lead to inconsistent CVD burden estimates. In the future, we advocate the continued use of diverse analytical methods to validate the results of this study.

In summary, this study used the latest GBD 2021 database to comprehensively update the global trends and burden of CVD attributable to HBMI from 1990 to 2021. Through comparisons across different regions, countries, genders, and age groups, as well as decomposition analysis, we identified and quantified the contributions of various factors to changes in the disease burden. Over the past 30 years, the global burden of CVD attributable to HBMI has declined, but HBMI remains a significant factor in CVD burden. Exploring the interaction between HBMI and CVD development has been a major research hotspot in public health in recent years. The research results provide decision-makers with a precise scientific basis to more effectively prevent and reduce cardiovascular health problems attributable to HBMI.

## Data Availability

The original contributions presented in the study are included in the article/**Supplementary Material**. Further inquiries can be directed to the corresponding author.
