# Supplementary material for: Global, regional, and national burden of cardiovascular disease attributable to high body mass index from 1990 to 2021 and projection to 2045
Source: Front Endocrinol (Lausanne). 2025 Apr 28;16:1546176. doi: 10.3389/fendo.2025.1546176 (PMC12066273; doi:10.3389/fendo.2025.1546176)
Supplement: Supplementary file 1 [file Presentation1.pptx]

## Slide 1
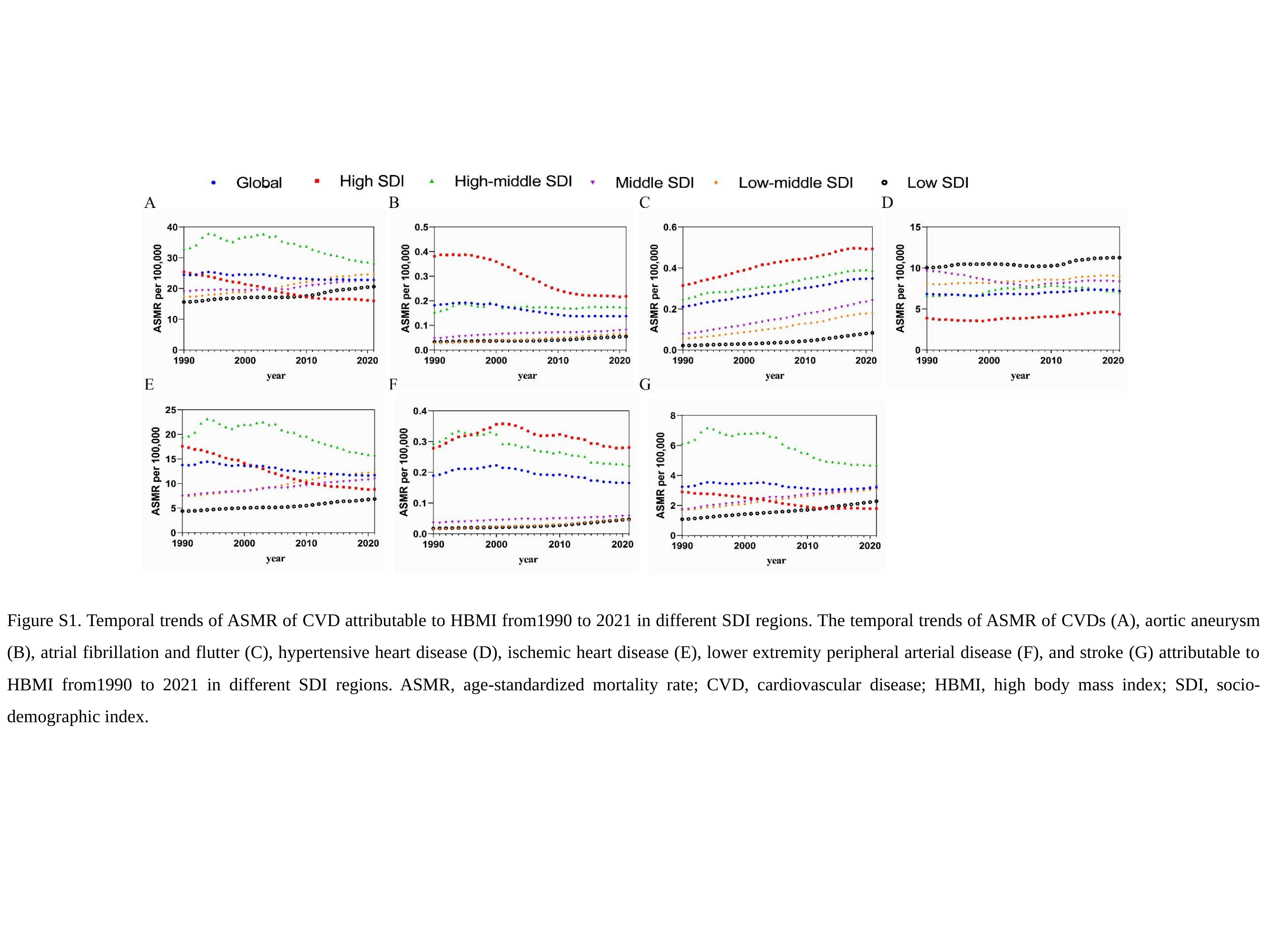

Figure S1. Temporal trends of ASMR of CVD attributable to HBMI from1990 to 2021 in different SDI regions. The temporal trends of ASMR of CVDs (A), aortic aneurysm (B), atrial fibrillation and flutter (C), hypertensive heart disease (D), ischemic heart disease (E), lower extremity peripheral arterial disease (F), and stroke (G) attributable to HBMI from1990 to 2021 in different SDI regions. ASMR, age-standardized mortality rate; CVD, cardiovascular disease; HBMI, high body mass index; SDI, socio-demographic index.

## Slide 2
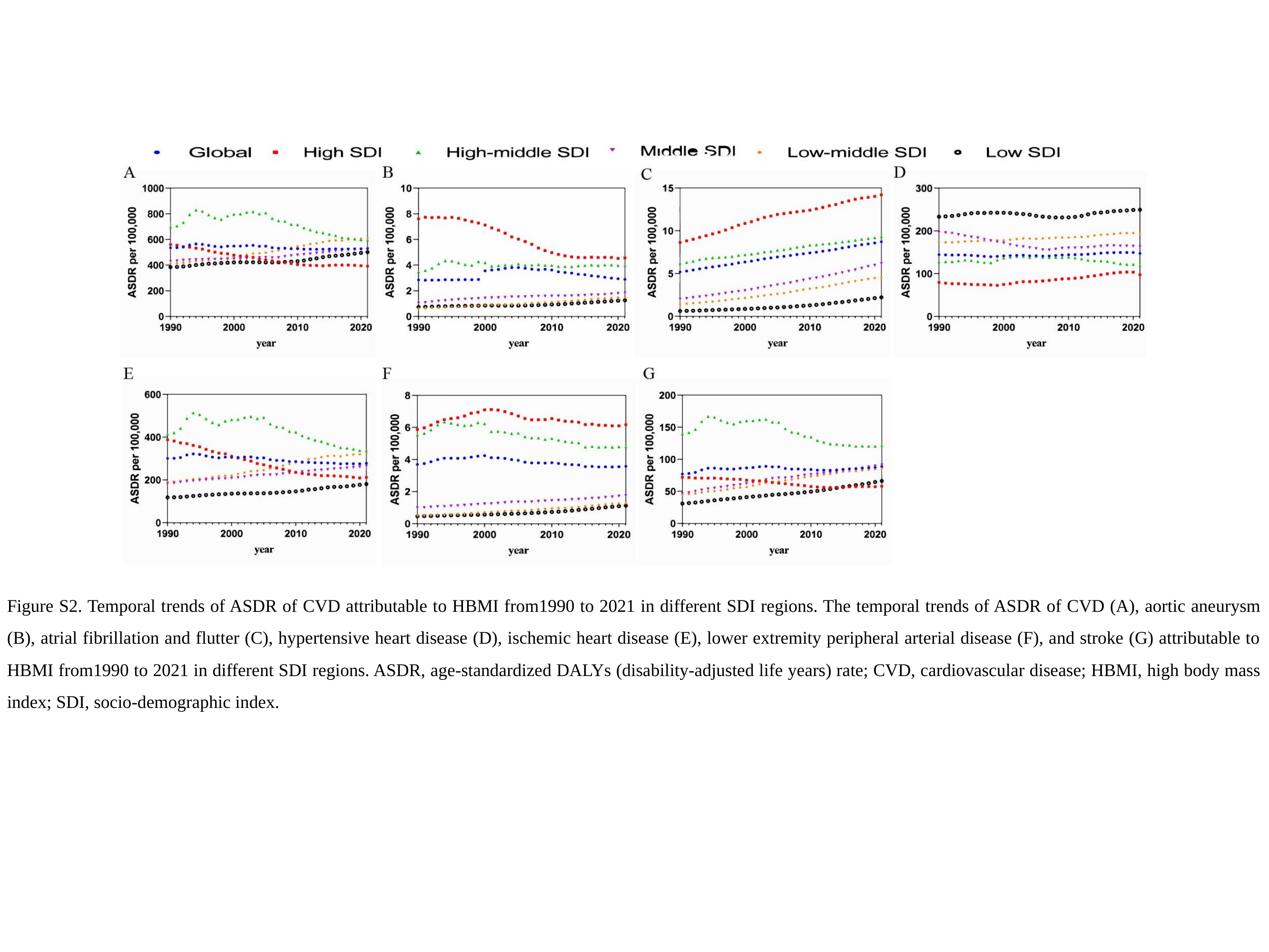

Figure S2. Temporal trends of ASDR of CVD attributable to HBMI from1990 to 2021 in different SDI regions. The temporal trends of ASDR of CVD (A), aortic aneurysm (B), atrial fibrillation and flutter (C), hypertensive heart disease (D), ischemic heart disease (E), lower extremity peripheral arterial disease (F), and stroke (G) attributable to HBMI from1990 to 2021 in different SDI regions. ASDR, age-standardized DALYs (disability-adjusted life years) rate; CVD, cardiovascular disease; HBMI, high body mass index; SDI, socio-demographic index.

## Slide 3
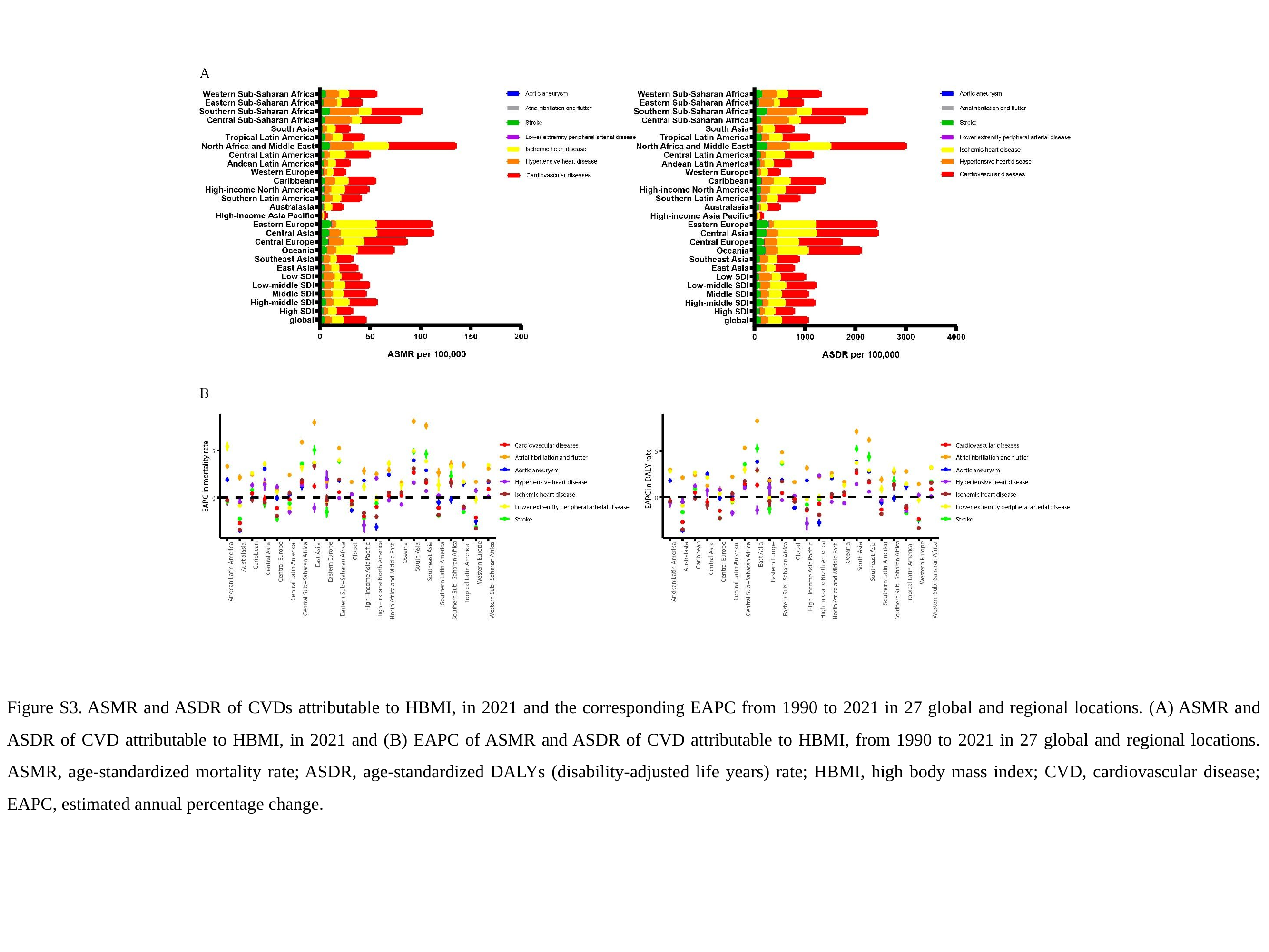

Figure S3. ASMR and ASDR of CVDs attributable to HBMI, in 2021 and the corresponding EAPC from 1990 to 2021 in 27 global and regional locations. (A) ASMR and ASDR of CVD attributable to HBMI, in 2021 and (B) EAPC of ASMR and ASDR of CVD attributable to HBMI, from 1990 to 2021 in 27 global and regional locations. ASMR, age-standardized mortality rate; ASDR, age-standardized DALYs (disability-adjusted life years) rate; HBMI, high body mass index; CVD, cardiovascular disease; EAPC, estimated annual percentage change.

## Slide 4
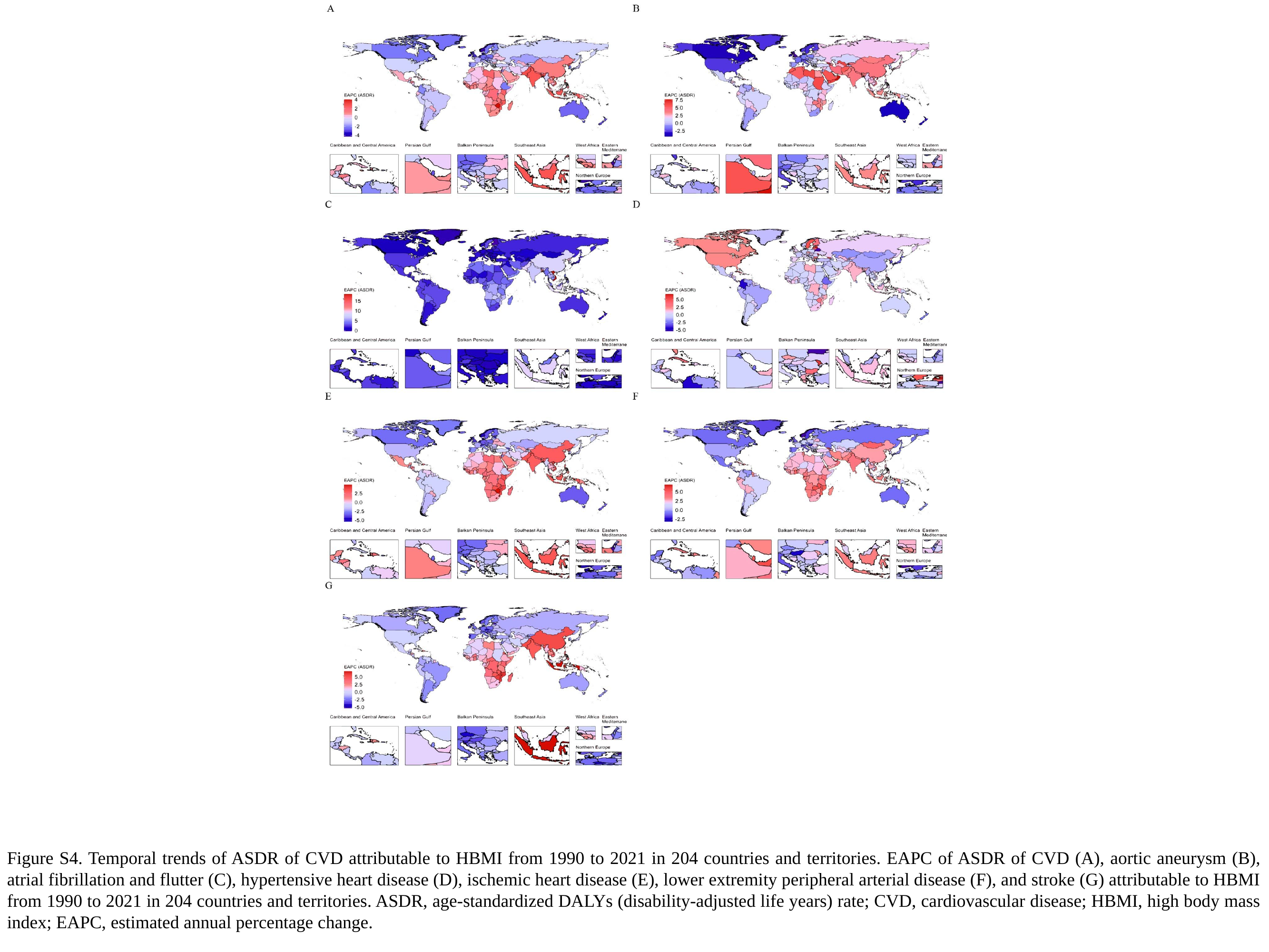

Figure S4. Temporal trends of ASDR of CVD attributable to HBMI from 1990 to 2021 in 204 countries and territories. EAPC of ASDR of CVD (A), aortic aneurysm (B), atrial fibrillation and flutter (C), hypertensive heart disease (D), ischemic heart disease (E), lower extremity peripheral arterial disease (F), and stroke (G) attributable to HBMI from 1990 to 2021 in 204 countries and territories. ASDR, age-standardized DALYs (disability-adjusted life years) rate; CVD, cardiovascular disease; HBMI, high body mass index; EAPC, estimated annual percentage change.

## Slide 5
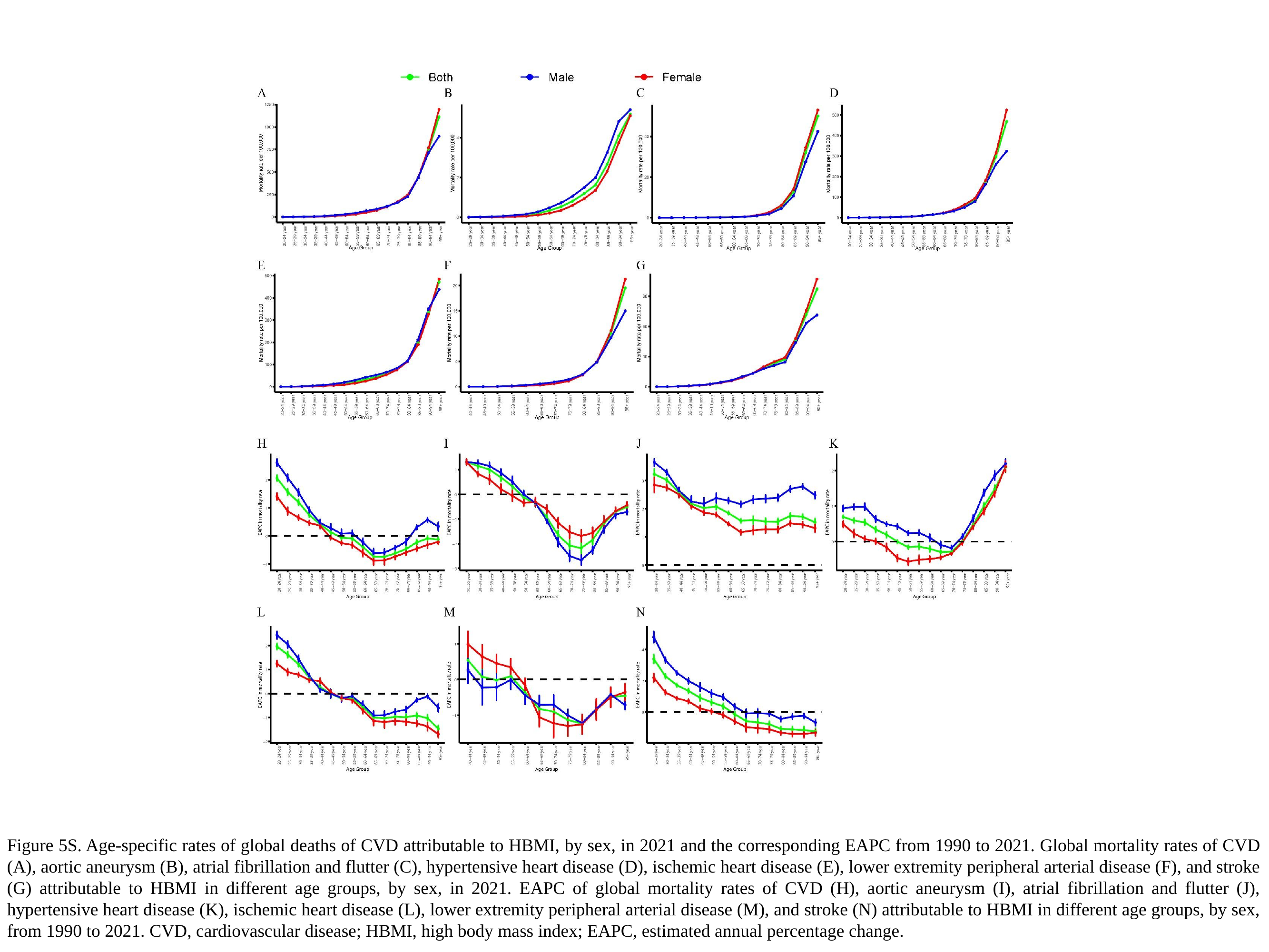

Figure 5S. Age-specific rates of global deaths of CVD attributable to HBMI, by sex, in 2021 and the corresponding EAPC from 1990 to 2021. Global mortality rates of CVD (A), aortic aneurysm (B), atrial fibrillation and flutter (C), hypertensive heart disease (D), ischemic heart disease (E), lower extremity peripheral arterial disease (F), and stroke (G) attributable to HBMI in different age groups, by sex, in 2021. EAPC of global mortality rates of CVD (H), aortic aneurysm (I), atrial fibrillation and flutter (J), hypertensive heart disease (K), ischemic heart disease (L), lower extremity peripheral arterial disease (M), and stroke (N) attributable to HBMI in different age groups, by sex, from 1990 to 2021. CVD, cardiovascular disease; HBMI, high body mass index; EAPC, estimated annual percentage change.

## Slide 6
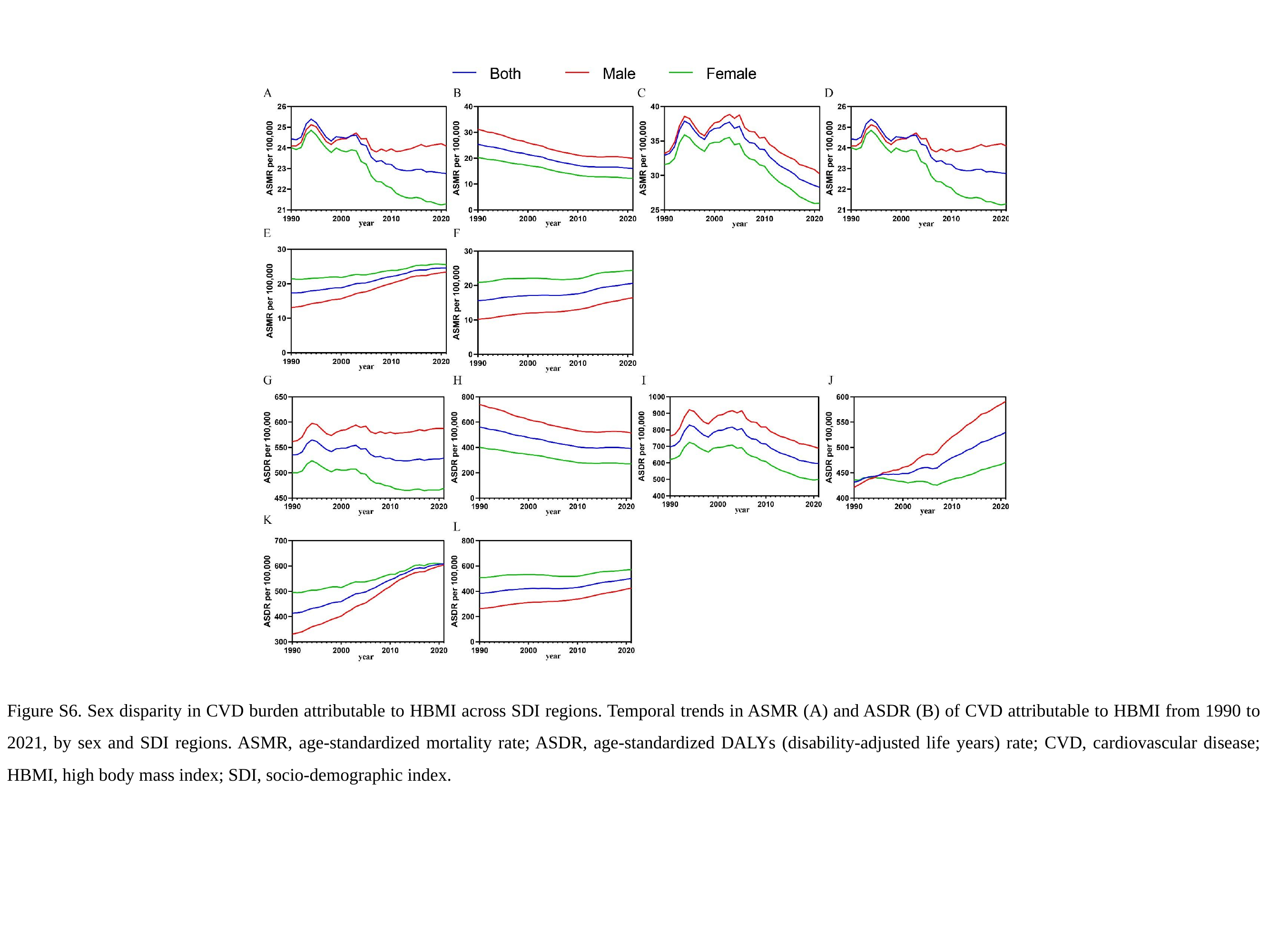

Figure S6. Sex disparity in CVD burden attributable to HBMI across SDI regions. Temporal trends in ASMR (A) and ASDR (B) of CVD attributable to HBMI from 1990 to 2021, by sex and SDI regions. ASMR, age-standardized mortality rate; ASDR, age-standardized DALYs (disability-adjusted life years) rate; CVD, cardiovascular disease; HBMI, high body mass index; SDI, socio-demographic index.

## Slide 7
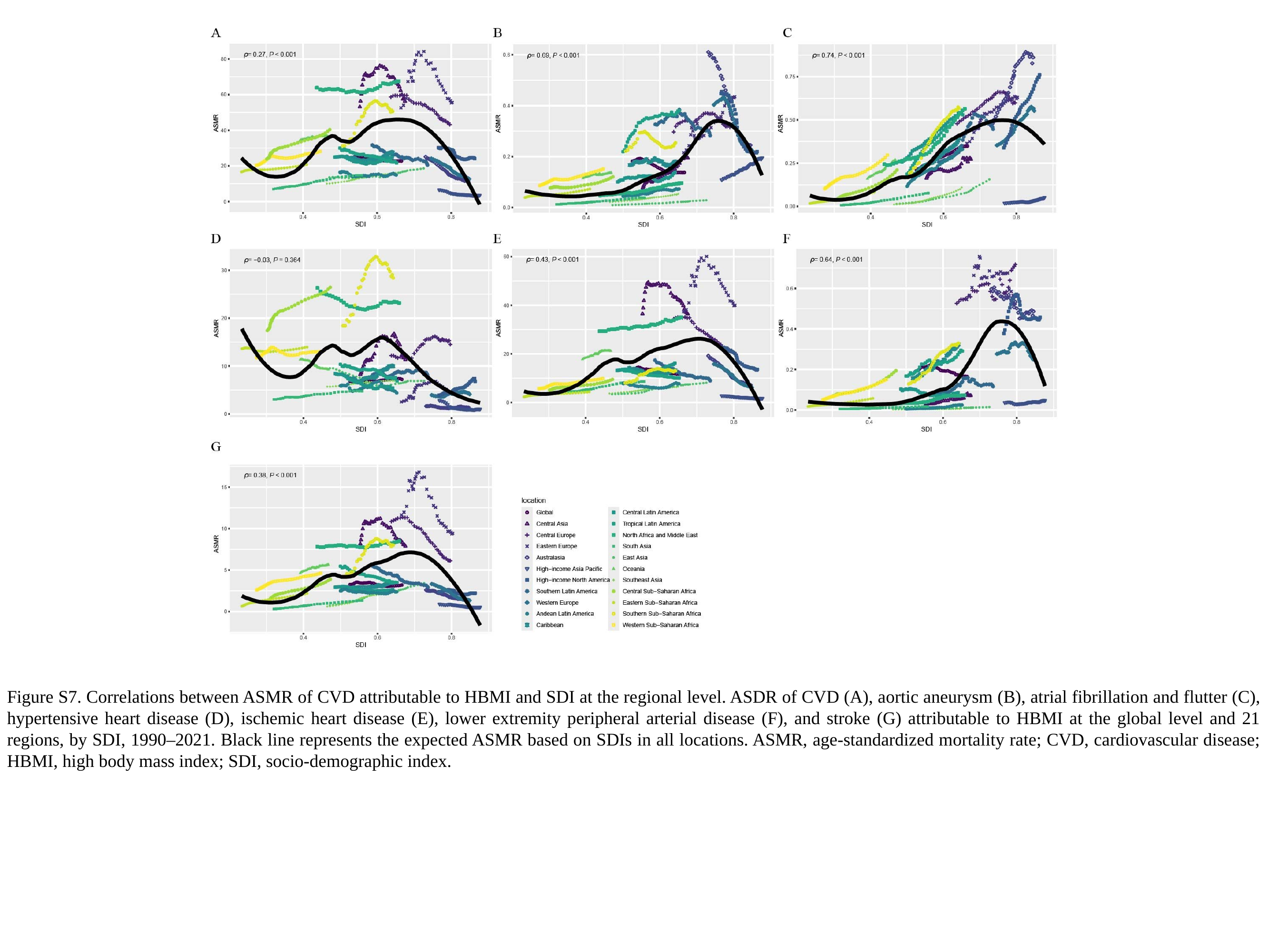

Figure S7. Correlations between ASMR of CVD attributable to HBMI and SDI at the regional level. ASDR of CVD (A), aortic aneurysm (B), atrial fibrillation and flutter (C), hypertensive heart disease (D), ischemic heart disease (E), lower extremity peripheral arterial disease (F), and stroke (G) attributable to HBMI at the global level and 21 regions, by SDI, 1990–2021. Black line represents the expected ASMR based on SDIs in all locations. ASMR, age-standardized mortality rate; CVD, cardiovascular disease; HBMI, high body mass index; SDI, socio-demographic index.

## Slide 8
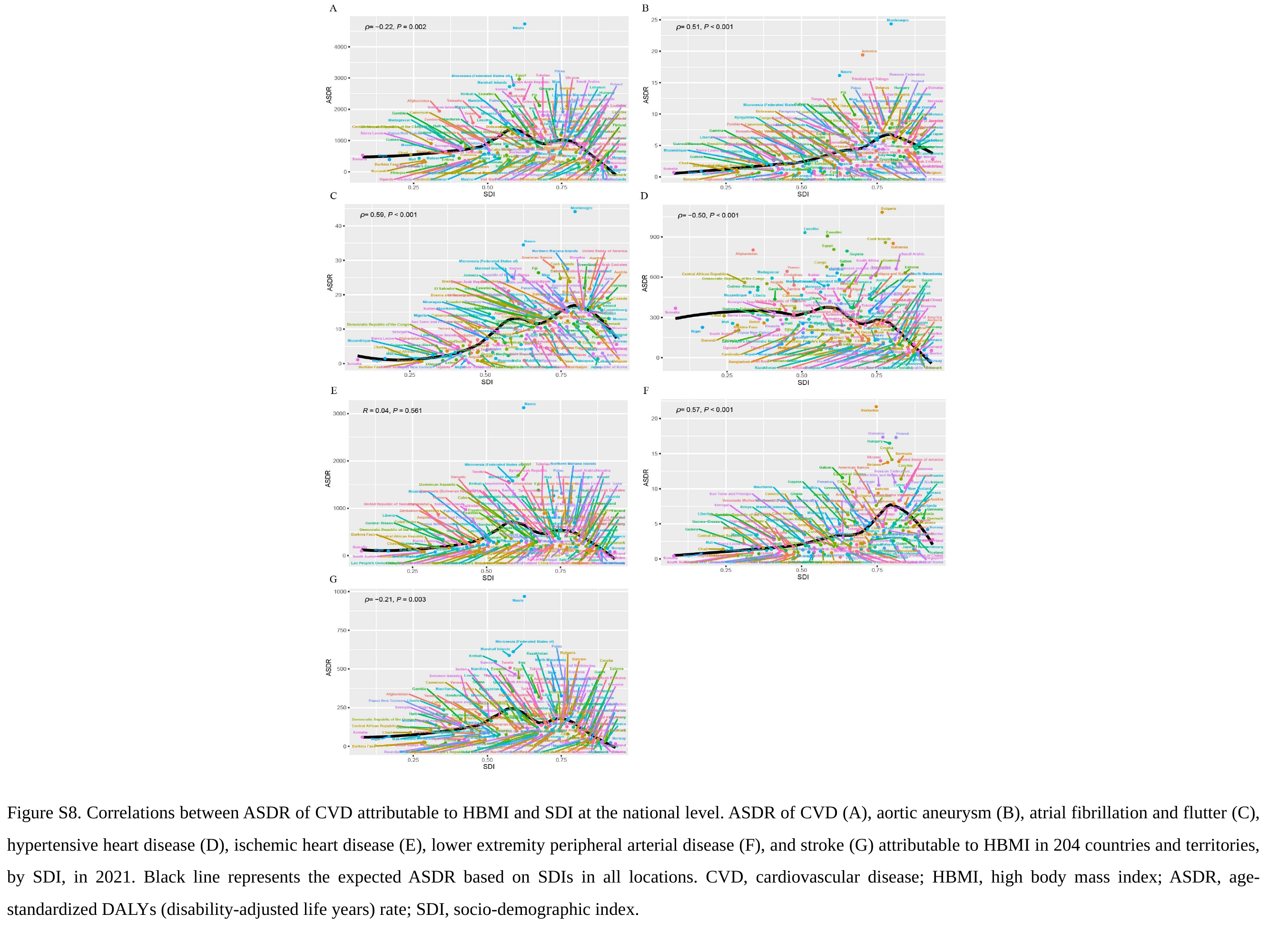

Figure S8. Correlations between ASDR of CVD attributable to HBMI and SDI at the national level. ASDR of CVD (A), aortic aneurysm (B), atrial fibrillation and flutter (C), hypertensive heart disease (D), ischemic heart disease (E), lower extremity peripheral arterial disease (F), and stroke (G) attributable to HBMI in 204 countries and territories, by SDI, in 2021. Black line represents the expected ASDR based on SDIs in all locations. CVD, cardiovascular disease; HBMI, high body mass index; ASDR, age-standardized DALYs (disability-adjusted life years) rate; SDI, socio-demographic index.

## Slide 9
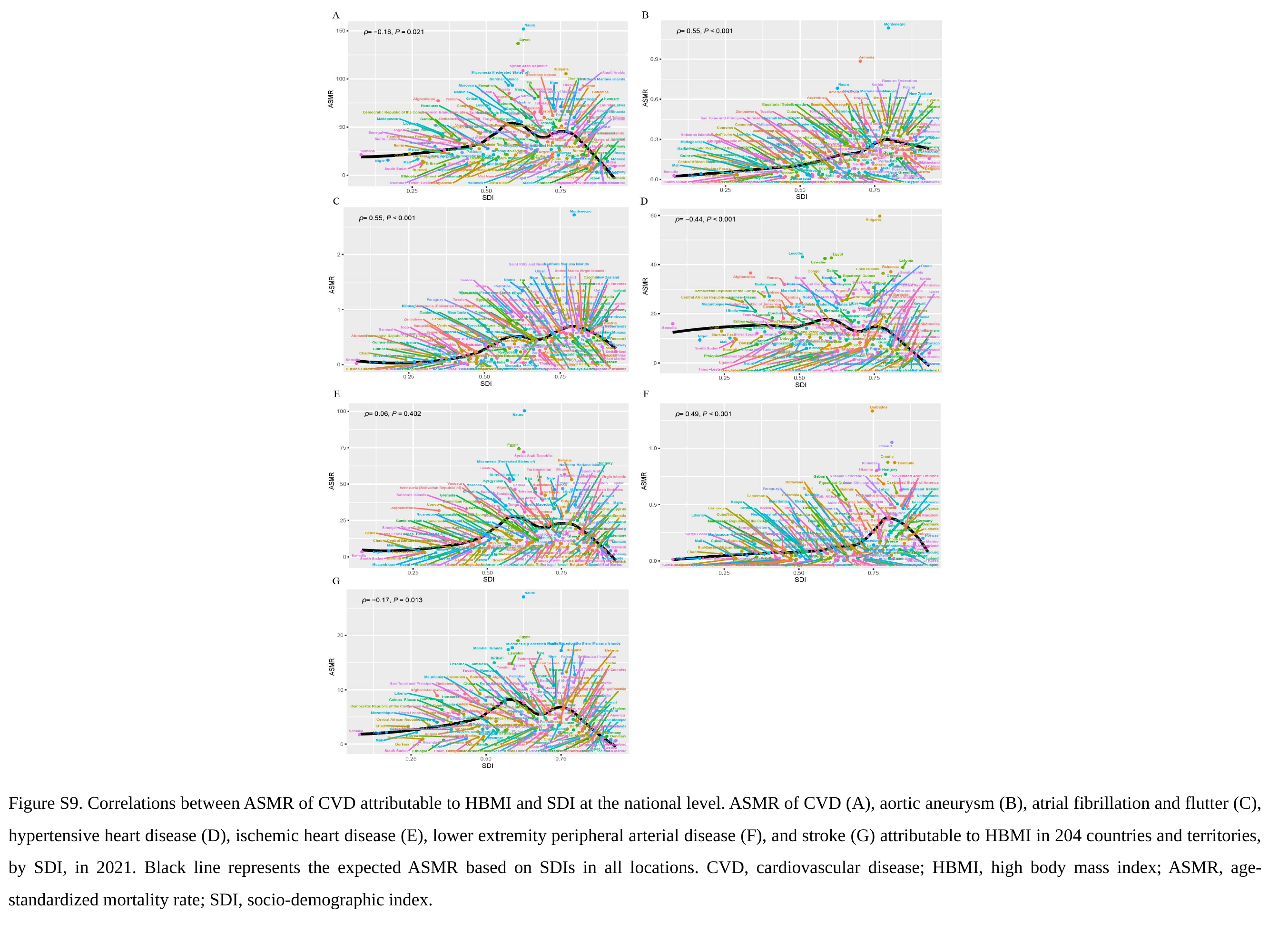

Figure S9. Correlations between ASMR of CVD attributable to HBMI and SDI at the national level. ASMR of CVD (A), aortic aneurysm (B), atrial fibrillation and flutter (C), hypertensive heart disease (D), ischemic heart disease (E), lower extremity peripheral arterial disease (F), and stroke (G) attributable to HBMI in 204 countries and territories, by SDI, in 2021. Black line represents the expected ASMR based on SDIs in all locations. CVD, cardiovascular disease; HBMI, high body mass index; ASMR, age-standardized mortality rate; SDI, socio-demographic index.

## Slide 10
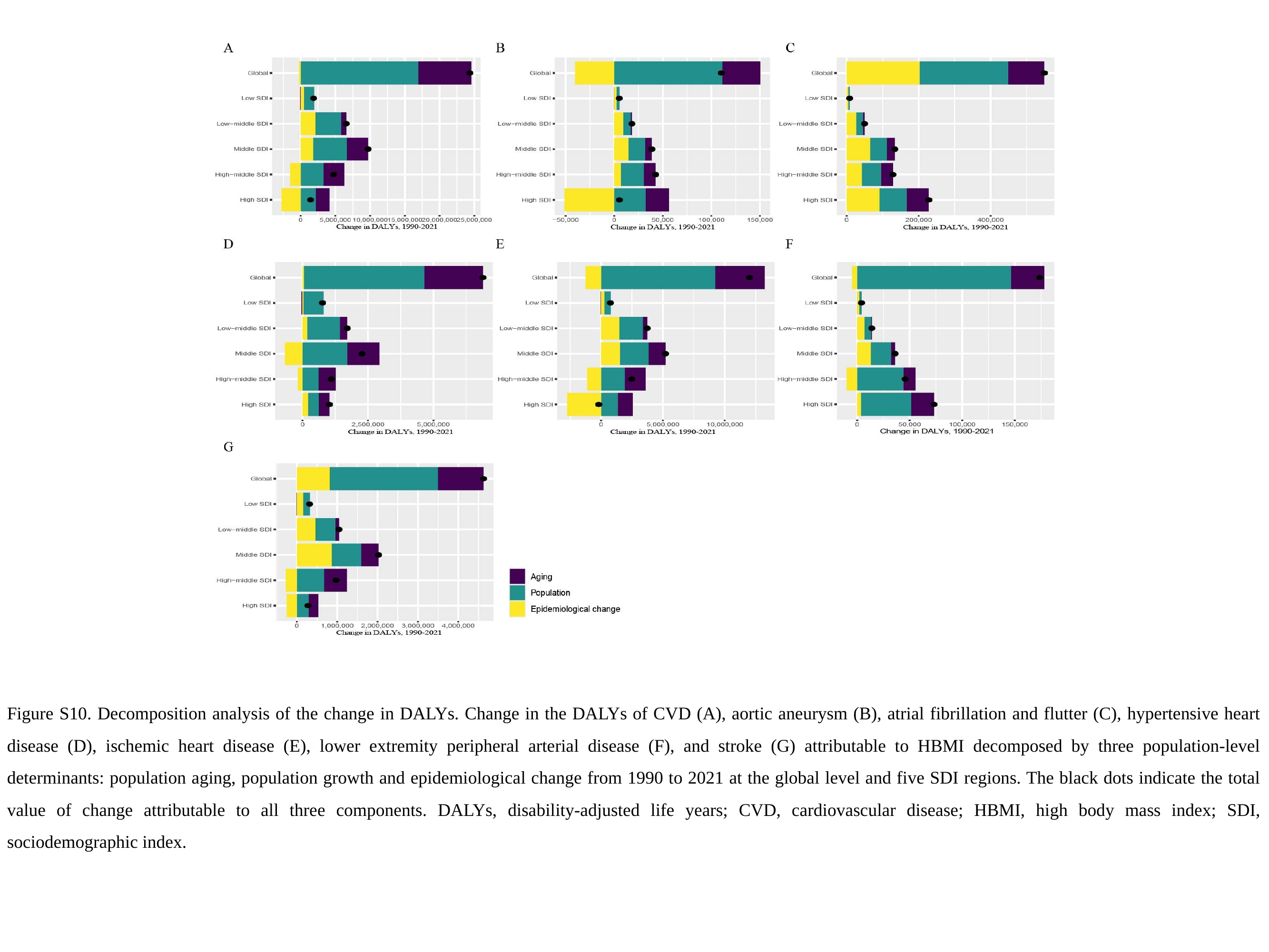

Figure S10. Decomposition analysis of the change in DALYs. Change in the DALYs of CVD (A), aortic aneurysm (B), atrial fibrillation and flutter (C), hypertensive heart disease (D), ischemic heart disease (E), lower extremity peripheral arterial disease (F), and stroke (G) attributable to HBMI decomposed by three population-level determinants: population aging, population growth and epidemiological change from 1990 to 2021 at the global level and five SDI regions. The black dots indicate the total value of change attributable to all three components. DALYs, disability-adjusted life years; CVD, cardiovascular disease; HBMI, high body mass index; SDI, sociodemographic index.

## Slide 11
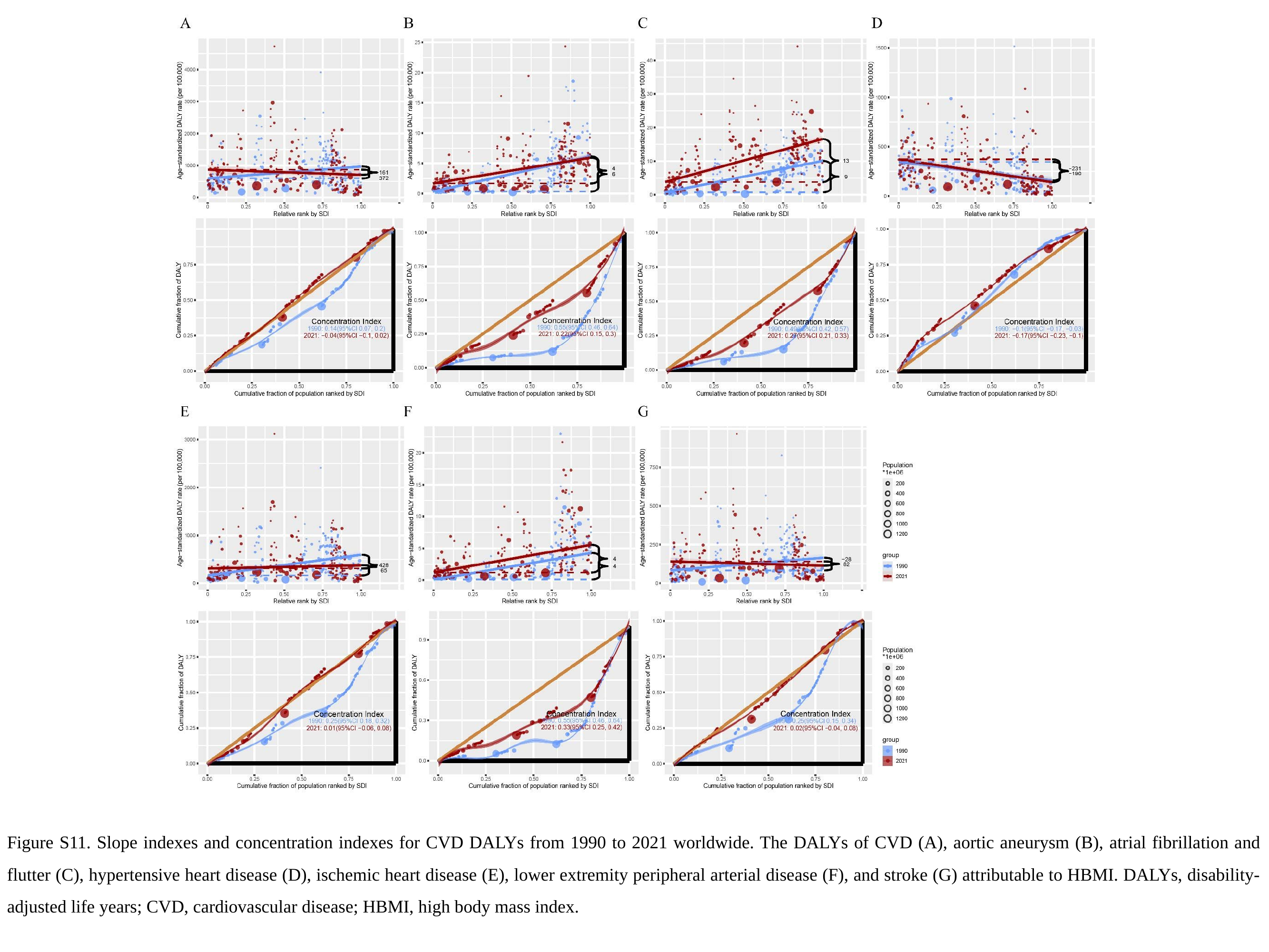

Figure S11. Slope indexes and concentration indexes for CVD DALYs from 1990 to 2021 worldwide. The DALYs of CVD (A), aortic aneurysm (B), atrial fibrillation and flutter (C), hypertensive heart disease (D), ischemic heart disease (E), lower extremity peripheral arterial disease (F), and stroke (G) attributable to HBMI. DALYs, disability-adjusted life years; CVD, cardiovascular disease; HBMI, high body mass index.
