# Supplementary material for: Global, regional, and national burden of cardiovascular disease attributable to high body mass index from 1990 to 2021 and projection to 2045
Source: Front Endocrinol (Lausanne). 2025 Apr 28;16:1546176. doi: 10.3389/fendo.2025.1546176 (PMC12066273; doi:10.3389/fendo.2025.1546176)
Supplement: Supplementary file 2 [file Table1.docx]

Table S1. ICD codes associated with CVD causes of death

| Cause | Level | Vital Registration Sample | Vital Registration | Verbal Autopsy | Surveillance | MITS diagnosed | ICD9 | ICD10 |
| --- | --- | --- | --- | --- | --- | --- | --- | --- |
| cardiovascular disease | 2 | 825 | 24191 | 1588 | 2 | 4 | 391-391.9, 392.0, 393-398.9, 402-402.9, 410-414.9, 416.0, 417-417.9, 420- 423, 423.1-423.9, 424.0-424.3, 424.8, 425.0- 425.5, 425.7-425.8, 427.0-427.3, 427.6- 427.8, 429.0, 430-435.9, 437.0-437.2, 437.5-437.8, 440.2, 440.4, 441- 443.9, 447-454.9, 456, 456.3-457, 457.1, 457.8-457.9, 459, 459.1-459.3 | B33.2, G45-G46.8, I01-I01.9, I02.0, I05-I09.9, I11-I11.9, I20-I25.9, I27.0, I27.2, I28- I28.9, I30-I31.1, I31.8-I37.8, I38-I41.9, I42.1-I42.8, I43-I43.9, I47- I48.9, I51.0-I51.4, I60-I63.9, I65-I66.9, I67.0-I67.3, I67.5-I67.6, I68.0-I68.2, I69.0-I69.3, I70.2-I70.8, I71-I73.9, I77- I83.9, I86-I89.0, I89.9, I98, K75.1 |
| ischemic heart disease | 3 | 825 | 23129 | 1474 |  |  | 410-414.9 | I20-I25.9 |
| stroke | 3 | 825 | 23629 | 1322 | 1 |  | 430-435.9, 437.0-437.2, 437.5-437.8 | G45-G46.8, I60-I63.9, I65-I66.9, I67.0- I67.3, I67.5-I67.6, I68.1-I68.2, I69.0- I69.3 |
| hypertensive heart disease | 3 | 825 | 22224 |  |  |  | 402-402.9 | I11-I11.9 |
| atrial fibrillation and flutter | 3 | 448 | 20570 |  |  |  | 427.3 | I48-I48.9 |
| aortic aneurysm | 3 | 448 | 21454 |  |  |  | 441-441.9 | I71-I71.9 |
| lower extremity peripheral arterial disease | 3 | 448 | 20566 |  |  |  | 440.2, 440.4, 443.0-443.9 | I70.2-I70.8, I73-I73.9 |

International Classification of Disease (ICD); Cardiovascular disease (CVD).

Table S2 Global and regional DALYs of CVD attributable to HBMI in 1990 and 2021, and EAPC of ASDR from 1990 to 2021

|  | CVD | | | | Aortic aneurysm | | | | Atrial fibrillation and flutter | | | | Stroke | | | | Lower extremity peripheral arterial disease | | | | Hypertensive heart disease | | | | Ischemic heart disease | | | |
| --- | --- | --- | --- | --- | --- | --- | --- | --- | --- | --- | --- | --- | --- | --- | --- | --- | --- | --- | --- | --- | --- | --- | --- | --- | --- | --- | --- | --- |
| location | DALY number in 1990 (×1000) | DALY number in 2021 (×1000) | ASDR in 2021 (per 100,000) | EAPC, 1990–2021 | DALY number in 1990 (×1000) | DALY number in 2021 (×1000) | ASDR in 2021 (per 100,000) | EAPC, 1990–2021 | DALY number in 1990 (×1000) | DALY number in 2021 (×1000) | ASDR in 2021 (per 100,000) | EAPC, 1990–2021 | DALY number in 1990 (×1000) | DALY number in 2021 (×1000) | ASDR in 2021 (per 100,000) | EAPC, 1990–2021 | DALY number in 1990 (×1000) | DALY number in 2021 (×1000) | ASDR in 2021 (per 100,000) | EAPC, 1990–2021 | DALY number in 1990 (×1000) | DALY number in 2021 (×1000) | ASDR in 2021 (per 100,000) | EAPC, 1990–2021 | DALY number in 1990 (×1000) | DALY number in 2021 (×1000) | ASDR in 2021 (per 100,000) | EAPC, 1990–2021 |
| global | 21082.66 (11331.04-32461.61) | 45426.88 (23767.19-69603.29) | 529.00 (277.28-808.64) | -0.19  (-0.24--0.13) | 137.24 (74.82-230.63) | 247.36 (134.54-414.71) | 2.88 (1.56-4.83) | -1.13  (-1.27--0.99) | 175.03 (68.00-298.16) | 724.57 (303.53-1246.37) | 8.71 (3.65-15.06) | 1.64  (1.6-1.69) | 3061.75 (389.20-6633.53) | 7687.49 (623.22-15857.42) | 88.84 (7.22-182.93) | 0.14  (0.02-0.26) | 127.31 (34.91-309.72) | 300.91 (88.47-649.35) | 3.57 (1.05-7.73) | -0.46  (-0.61--0.31) | 5666.02 (4251.06-7069.74) | 12551.75 (9489.12-15451.02) | 147.33 (109.06-183.45) | 0.15  (0.1-0.21) | 11915.31 (4691.69-19595.97) | 23914.79 (9468.66-38327.42) | 277.65 (109.80-445.24) | -0.47  (-0.55--0.4) |
| SDI |  |  |  |  |  |  |  |  |  |  |  |  |  |  |  |  |  |  |  |  |  |  |  |  |  |  |  |  |
| High SDI | 6061.55 (2832.14-9729.48) | 7481.11 (4022.04-11418.02) | 392.18 (208.92-599.02) | -1.25  (-1.36--1.13) | 85.14 (45.56-142.08) | 90.40 (48.60-152.05) | 4.56 (2.48-7.64) | -2.21  (-2.41--2.02) | 95.79 (37.10-167.99) | 324.19 (134.75-566.66) | 14.21 (5.95-24.65) | 1.58  (1.48-1.68) | 776.38 (78.92-161753) | 1049.20 (78.75-2119.66) | 58.13 (3.86-117.12) | -0.93  (-1.04--0.82) | 65.52 (18.34-155.83) | 138.75 (42.68-289.43) | 6.18 (1.90-12.77) | -0.16  (-0.35-0.03) | 858.43 (643.92-1067.63) | 1888.25 (1342.87-2367.63) | 97.25 (76.46-115.81) | 1.16  (0.99-1.33) | 4180.30 (1655.24-6868.74) | 3990.32 (1631.14-6343.08) | 211.85 (88.32-333.67) | -2.18  (-2.3--2.06) |
| High-middle SDI | 6767.96（3125.86-10909.56） | 11532.84（5403.65-18590.76） | 595.53（279.91-958.54） | -0.89  (-1.15--0.63) | 34.85（19.33-60.23） | 77.47（41.71-131.14） | 3.97（2.14-6.71） | 0.02 (-0.15-0.19) | 53.74（21.65-91.09） | 182.17（74.55-317.11） | 9.33（3.81-16.20） | 1.28 (1.24-1.32) | 1377.64（166.13-2862.11） | 2346.77（192.25-4872.14） | 121.40（9.80-251.57） | -1.04  (-1.29--0.78) | 49.26（12.16-124.84） | 95.03（26.54-212.00） | 4.79（1.33-10.71） | -0.92  (-1.09--0.75) | 1203.85（903.41-1536.88） | 2296.32（1583.47-3023.14） | 118.94（81.23-157.67） | -0.11  (-0.29-0.06) | 4048.62（1605.20-6607.68） | 6535.09（2601.99-10876.72） | 337.11（134.65-559.90） | -1.13  (-1.44--0.83) |
| Middle SDI | 4598.34（2946.82-6564.17） | 14315.23（7452.58-21827.67） | 530.25（284.53-798.72） | 0.65  (0.59-0.71) | 11.41（6.20-19.14） | 50.15（27.26-84.80） | 1.84（1.00-3.11） | 1.47 (1.3-1.63) | 16.89（6.48-28.28） | 150.96（62.58-256.71） | 6.18（2.57-10.42） | 3.67  (3.59-3.75) | 527.34（44.71-1301.31） | 2551.55（197.78-5325.53） | 91.82（7.28-192.11） | 2.07  (1.97-2.18) | 8.73（2.53-22.36） | 44.93（12.43-104.65） | 1.75（0.49-4.06） | 1.76  (1.7-1.82) | 1979.47（1355.21-2590.23） | 4255.95（3088.32-5431.40） | 163.33（112.87-214.45） | -0.6  (-0.77--0.42) | 2054.50（792.52-3326.41） | 7261.69（2915.60-11757.54） | 265.32（106.00-430.45） | 1.16  (1.13-1.19) |
| Low-middle SDI | 2678.79（1658.78-3853.84） | 9244.24（5209.81-13581.71） | 608.36（347.39-888.55） | 1.4  (1.36-1.45) | 3.91（2.00-6.84） | 22.14（11.88-37.24） | 1.49（0.80-2.50） | 2.87 (2.81-2.94) | 7.02（2.80-11.74） | 56.69（23.03-93.08） | 4.57（1.84-7.43） | 3.97  (3.9-4.05) | 295.30（29.49-669.13） | 1337.69（115.55-2732.60） | 85.75（7.63-175.19） | 2.22  (2.12-2.32) | 2.62（0.70-6.94） | 16.57（4.61-38.72） | 1.27（0.35-2.97） | 3.01  (2.97-3.05) | 1064.62（740.29-1387.68） | 2774.97（2163.09-3408.02） | 192.73（147.45-244.13） | 0.39  (0.37-0.41) | 1305.31（501.31-2102.08） | 5036.16（2015.82-7911.41） | 322.56（127.92-510.96） | 1.91  (1.83-1.99) |
| Low SDI | 932.98（616.29-1270.32） | 2795.04（1790.71-3953.35） | 501.03（325.37-702.90） | 0.75  (0.67-0.84) | 1.70（0.78-3.30） | 6.84（3.23-13.20） | 1.58  (1.41-1.76) | 1.58 (1.41-1.76) | 1.24（0.42-2.33） | 9.60（3.65-16.71） | 2.21（0.85-3.91） | 4.22  (4.07-4.36) | 77.41（2.32-201.86） | 392.91（30.46-848.99） | 66.19（5.54-139.35） | 2.35  (2.29-2.42) | 0.89（0.22-2.70） | 5.01（1.22-13.83） | 1.13（0.28-3.10） | 2.85  (2.67-3.03) | 550.42（306.31-765.53） | 1318.23（895.03-1781.08） | 249.65（164.84-328.72） | 0.1  (0.02-0.18) | 301.31（104.13-496.04） | 1062.46（400.81-1727.97） | 180.59（67.05-294.97） | 1.3  (1.22-1.39) |
| Region |  |  |  |  |  |  |  |  |  |  |  |  |  |  |  |  |  |  |  |  |  |  |  |  |  |  |  |  |
| East Asia | 2321.96（1667.59-3288.83） | 8218.28（4055.21-13358.56） | 392.39（197.58-631.99） | 1.3  (1.12-1.47) | 2.47（1.24-4.40） | 16.60（8.54-29.87） | 0.80（0.41-1.43） | 3.84  (3.72-3.96) | 2.36（0.58-4.98） | 78.96（30.81-135.78） | 3.90（1.53-6.67） | 8.24  (8.07-8.41) | 159.97（-59.28-680.02） | 2038.67（172.86-4450.79） | 93.47（7.91-203.80） | 5.27  (4.85-5.69) | 3.60（0.97-9.71） | 25.61（5.97-65.77） | 1.16（0.27-3.00） | 2.88  (2.84-2.93) | 1430.32（849.32-1974.45） | 2468.56（1553.65-3558.54） | 120.29（72.71-178.97） | -1.38  (-1.81--0.96) | 723.25（247.87-1209.62） | 3589.87（1327.42-6129.62） | 172.77（63.71-294.40） | 2.92  (2.72-3.13) |
| Southeast Asia | 785.11（518.96-1117.79） | 3114.70（1795.11-4638.46） | 437.64（257.84-646.45） | 1.61  (1.5-1.72) | 1.20（0.62-2.00） | 7.10（3.78-11.65） | 1.10（0.58-1.80） | 2.77  (2.69-2.85) | 1.29（0.37-2.50） | 19.00（7.63-32.06） | 3.17（1.24-5.43） | 6.2  (5.97-6.43) | 63.49（-22.26-212.45） | 586.19（46.94-1257.38） | 79.86（6.78-170.41） | 4.35  (3.93-4.78) | 0.61（0.16-1.64） | 3.93（0.98-9.98） | 0.62（0.15-1.60） | 2.9  (2.86-2.95) | 388.65（235.39-525.91） | 1159.65（809.40-1490.33） | 168.45（118.20-217.74） | 0.61  (0.55-0.67) | 329.87（115.71-527.06） | 1338.82（500.72-2147.68） | 184.43（69.04-296.62） | 1.78  (1.67-1.9) |
| Oceania | 34.89（17.47-57.34） | 98.57（46.04-163.50） | 1053.29（504.47-1728.29） | 0.24  (0.19-0.29) | 0.09（0.04-0.16） | 0.28（0.15-0.49） | 3.27（1.74-5.68） | 0.49  (0.4-0.58) | 0.13（0.05-0.25） | 0.57（0.24-1.01） | 8.11（3.35-14.25） | 1.65  (1.59-1.71) | 5.92（0.24-14.17） | 18.36（0.44-42.04） | 187.72（6.04-425.49） | 0.49  (0.41-0.57) | 0.03（0.01-0.07） | 0.12（0.04-0.25） | 1.69（0.53-3.62） | 1.3  (1.1-1.51) | 9.81（6.24-13.92） | 21.67（14.66-31.79） | 243.69（171.23-353.90） | -0.65  (-0.69--0.61) | 18.90（7.24-31.69） | 57.57（24.22-97.25） | 608.81（252.86-1030.24） | 0.56  (0.48-0.65) |
| Central Europe | 1881.70（885.13-3055.81） | 1885.40（1057.88-2924.07） | 864.56（483.14-1346.83） | -1.46  (-1.55--1.38) | 10.18（5.42-17.47） | 15.44（8.12-26.76） | 7.26（3.83-12.61） | -0.09  (-0.32-0.14) | 16.33（6.52-28.13） | 36.52（15.07-64.44） | 15.44（6.38-27.38） | 0.85  (0.7-1) | 369.70（29.68-780.36） | 305.49（29.51-628.18） | 143.09（12.74-292.45） | -2.26  (-2.45--2.07) | 14.37（3.75-33.13） | 30.17（9.32-64.21） | 13.10（3.99-27.79） | 0.4  (0.16-0.64) | 341.70（266.98-420.82） | 576.78（392.43-742.47） | 256.77（182.69-324.76） | 0.79  (0.55-1.04) | 1129.42（448.98-1827.48） | 921.00（371.49-1521.55） | 428.90（173.54-709.08） | -2.26  (-2.38--2.14) |
| Central Asia | 590.33（247.46-954.60） | 1001.89（461.57-1613.13） | 1223.30（573.34-1950.20） | -0.55  (-0.95--0.14) | 0.98（0.52-1.70） | 3.68（1.98-6.42） | 4.37（2.34-7.66） | 2.49  (2.3-2.67) | 2.56（1.02-4.38） | 6.54（2.67-11.65） | 9.09（3.67-16.08） | 1.24  (1.16-1.33) | 109.28（7.22-233.24） | 179.89（12.76-380.26） | 208.39（15.64-440.81） | -0.81  (-1.19--0.43) | 0.48（0.10-1.31） | 1.51（0.37-3.57） | 2.05（0.51-4.92） | 2.13  (2-2.27) | 85.51（68.29-103.81） | 177.45（135.76-223.72） | 225.17（167.21-286.40） | 0.73  (0.11-1.36) | 391.53（152.98-634.77） | 632.82（257.20-1033.59） | 774.23（310.58-1265.45） | -0.85  (-1.23--0.46) |
| Eastern Europe | 3117.49（1096.17-5255.90） | 4140.14（1642.52-6909.22） | 1207.62（475.73-2015.27） | -0.44(-1.01-0.13) | 15.18（8.30-25.58） | 35.56（18.92-59.69） | 10.50（5.61-17.58） | 1.77(1.5-2.05) | 24.53（10.15-40.58） | 59.34（25.17-103.40） | 16.43（6.97-28.51） | 1.78(1.65-1.92) | 741.52（76.30-1474.00） | 789.00（74.06-1560.77） | 233.66（20.74-462.53） | -1.25(-1.76--0.73) | 26.90（5.91-73.24） | 42.24（10.72-102.05） | 11.57（2.93-28.01） | -0.09(-0.4-0.22) | 168.68（139.92-195.27） | 312.74（239.27-385.02） | 90.73（71.47-110.51） | 1.05(0.11-1.99) | 2140.68（830.02-3454.95） | 2901.27（1125.69-4756.03） | 844.73（327.21-1379.46） | -0.4(-0.98-0.19) |
| High-income Asia Pacific | 257.81（147.34-394.69） | 355.59（180.46-557.57） | 84.69（40.79-134.90） | -1.45  (-1.65--1.24) | 4.42（2.42-7.46） | 17.24（8.83-27.31） | 3.89（2.03-6.20） | 1.81  (1.74-1.88) | 1.26（0.36-2.64） | 8.52（3.03-15.69） | 1.77（0.66-3.25） | 3.16  (2.9-3.42) | 42.31（4.36-107.31） | 71.10（8.04-155.85） | 18.79（1.95-40.03） | -0.8  (-0.95--0.66) | 2.62（0.78-6.64） | 7.61（2.24-17.14） | 1.34（0.38-3.09） | -0.23  (-0.36--0.09) | 87.92（58.62-119.64） | 93.54（44.90-149.94） | 18.25（11.86-25.80） | -2.84  (-3.51--2.16) | 119.29（42.65-194.49） | 157.58（59.03-254.15） | 40.64（15.57-65.35） | -1.29  (-1.34--1.24) |
| Australasia | 125.32（50.46-208.80） | 129.36（57.34-212.88） | 248.67（108.64-408.01） | -2.67  (-2.81--2.52) | 2.87（1.54-5.02） | 2.48（1.31-4.26） | 4.65（2.48-7.97） | -3.61  (-3.79--3.43) | 2.63（1.02-4.87） | 12.41（5.30-21.75） | 21.40（9.07-37.72） | 2.13  (1.98-2.28) | 14.20（1.10-29.56） | 19.39（1.41-40.67） | 39.14（2.52-80.29） | -1.62  (-1.72--1.51) | 2.25（0.57-5.47） | 4.74（1.53-9.33） | 7.81（2.57-15.22） | -0.89  (-1.02--0.76) | 6.70（4.80-8.68） | 13.46（8.33-17.82） | 24.32（16.24-31.00） | -0.52  (-0.95--0.09) | 96.67（37.67-158.50） | 76.88（31.52-124.37） | 151.36（61.90-243.27） | -3.48  (-3.61--3.35) |
| Southern Latin America | 329.76（157.82-529.62） | 382.27（211.20-580.59） | 443.99（242.31-675.09） | -1.33  (-1.42--1.25) | 3.40（1.85-5.93） | 5.38（2.76-9.35） | 6.25（3.21-10.89） | -0.56  (-0.8--0.33) | 2.90（1.14-5.27） | 9.42（3.83-16.72） | 10.44（4.26-18.55） | 1.89  (1.64-2.15) | 70.43（3.14-154.25） | 71.72（2.98-148.49） | 85.66（3.27-177.84） | -1.81  (-1.89--1.72) | 1.25（0.31-3.05） | 3.11（0.90-6.58） | 3.46（1.00-7.30） | 0.88  (0.54-1.23) | 71.89（55.36-88.61） | 115.62（75.59-149.42） | 129.88（86.97-166.68） | -0.34  (-0.47--0.2) | 179.88（71.91-293.43） | 177.02（71.83-284.13） | 208.30（84.82-334.19） | -1.81  (-1.92--1.7) |
| High-income North America | 2410.15（1120.41-3872.03） | 3598.61（2016.99-5332.23） | 602.11（343.10-879.10） | -0.71  (-0.83--0.6) | 33.02（17.49-56.44） | 29.81（15.84-50.51） | 5.05（2.71-8.51） | -2.74  (-3.05--2.43) | 42.43（16.69-79.11） | 165.66（70.08-283.00） | 23.85（10.09-40.69） | 2.23  (2.07-2.38) | 237.84（15.82-499.73） | 423.64（27.68-833.36） | 73.72（4.29-143.85） | -0.19  (-0.35--0.03) | 31.07（8.99-68.91） | 71.86（22.48-139.44） | 10.53（3.31-20.31） | -0.04  (-0.34-0.26) | 318.55（254.23-382.52） | 983.53（778.73-1178.29） | 172.21（142.82-201.13） | 2.31  (2.17-2.46) | 1747.24（692.87-2869.52） | 1924.09（806.24-3010.75） | 316.75（134.67-490.17） | -1.91  (-2.04--1.78) |
| Caribbean | 163.53（87.44-241.89） | 372.30（210.62-562.11） | 694.19（391.59-1047.19） | 0.5  (0.34-0.66) | 0.91（0.50-1.48） | 2.05（1.07-3.52） | 3.80（1.99-6.53） | -0.04  (-0.22-0.13) | 1.27（0.52-2.16） | 5.96（2.40-10.21） | 10.89（4.38-18.69） | 2.38  (2.31-2.45) | 21.29（1.75-46.58） | 54.84（2.72-114.78） | 103.26（5.05-215.62） | 0.92  (0.83-1.01) | 0.73（0.22-1.81） | 3.48（1.06-7.75） | 6.34（1.93-14.08） | 2.67  (2.55-2.78) | 47.20（36.94-58.83） | 124.03（94.91-153.88） | 230.58（176.69-285.28） | 1.2  (1.01-1.4) | 92.14（36.20-145.99） | 181.95（71.94-297.78） | 339.31（134.07-554.39） | -0.1  (-0.3-0.11) |
| Western Europe | 2835.37（1293.89-4580.66） | 2372.74（1252.57-3809.68） | 249.09（133.73-398.31） | -2.35  (-2.42--2.27) | 46.68（25.17-77.97） | 40.46（21.09-69.67） | 4.48（2.35-7.67） | -2.5  (-2.74--2.26) | 53.71（20.73-90.35） | 148.76（58.93-264.45） | 13.85（5.58-24.77） | 1.42  (1.3-1.53) | 410.31（38.74-852.05） | 309.31（24.94-656.46） | 35.71（2.43-75.64） | -2.5  (-2.6--2.39) | 33.14（8.72-85.39） | 52.44（14.54-124.74） | 4.89（1.39-11.61） | -0.32  (-0.57--0.07) | 390.36（248.71-526.13） | 667.21（285.61-993.37） | 59.46（31.29-83.16） | 0.23  (0.06-0.39) | 1901.19（740.56-3132.97） | 1154.56（453.20-1900.92） | 130.70（51.96-213.14） | -3.33  (-3.43--3.23) |
| Andean Latin America | 91.72（46.41-147.27） | 221.81（110.20-357.14） | 364.65（183.35-586.48） | -0.39  (-0.59--0.19) | 0.23（0.11-0.40） | 1.02（0.53-1.77） | 1.68（0.86-2.92） | 1.78  (1.61-1.96) | 0.81（0.30-1.54） | 5.97（2.41-10.71） | 10.37（4.16-18.54） | 2.99  (2.91-3.06) | 18.65（1.06-43.14） | 46.41（1.22-98.21） | 73.86（2.12-155.57） | -0.49  (-0.65--0.32) | 0.09（0.02-0.25） | 0.63（0.16-1.44） | 1.09（0.28-2.51） | 2.82  (2.67-2.97) | 26.36（20.15-32.83） | 51.49（36.48-68.30） | 87.42（60.88-117.12） | -0.59  (-1.01--0.15) | 45.58（17.56-75.61） | 116.29（46.44-190.68） | 190.23（75.75-312.05） | -0.44  (-0.81--0.07) |
| Central Latin America | 498.38（263.73-772.16） | 1474.96（727.35-2327.25） | 580.29（288.97-913.78） | -0.19  (-0.43-0.04) | 1.99（1.09-3.42） | 7.53（3.86-12.54） | 2.99（1.53-4.99） | 0.12  (-0.14-0.38) | 5.62（2.20-10.14） | 36.27（15.39-64.77） | 15.03（6.36-26.72） | 2.21  (2.17-2.25) | 77.15（4.33-164.89） | 201.98（6.86-425.26） | 77.85（2.83-163.36） | -0.51  (-0.68--0.33) | 1.83（0.56-4.23） | 5.80（1.74-12.66） | 2.38（0.71-5.22） | -0.46  (-0.69--0.24) | 130.91（103.85-159.74） | 248.38（179.78-324.94） | 101.12（71.30-132.94） | -1.7  (-1.94--1.45) | 280.88（113.03-454.91） | 975.01（408.10-1563.39） | 380.93（158.77-613.11） | 0.38  (0.13-0.62) |
| North Africa and Middle East | 2589.13（1448.19-3873.13） | 7129.55（3799.06-10548.49） | 1499.72（823.18-2207.62） | 0.05  (-0.01-0.1) | 2.32（1.04-4.38） | 11.27（5.96-19.91） | 2.28（1.19-4.05） | 2.05  (1.96-2.15) | 7.06（2.80-12.20） | 42.72（18.79-73.47） | 11.69（5.10-20.18） | 2.58  (2.44-2.71) | 381.93（30.43-819.17） | 1088.50（97.44-2145.66） | 218.45（20.96-436.62） | 0.09  (0.04-0.14) | 1.91（0.48-5.05） | 10.04（3.02-20.37） | 2.47（0.75-5.09） | 2.26  (2.16-2.37) | 845.48（596.10-1089.02） | 1910.27（1455.25-2382.73） | 440.20（317.57-558.72） | -0.49  (-0.61--0.36) | 1350.43（540.20-2177.88） | 4066.74（1735.59-6443.32） | 824.63（345.44-1313.89） | 0.32  (0.29-0.36) |
| Tropical Latin America | 742.56（384.53-1169.44） | 1406.61（728.84-2181.50） | 540.61（281.65-836.09） | -1.14  (-1.2--1.09) | 5.54（3.06-9.33） | 23.55（12.48-40.80） | 9.03（4.78-15.66） | 1.16  (0.89-1.44) | 5.16（1.99-9.37） | 36.49（14.65-64.47） | 14.59（5.85-25.88） | 2.8  (2.65-2.95) | 157.03（9.09-346.14） | 256.39（11.24-541.62） | 97.99（4.41-206.56） | -1.7  (-1.78--1.61) | 3.11（0.87-7.98） | 15.76（4.52-33.62） | 6.21（1.77-13.26） | 1.42  (1.23-1.61) | 216.91（178.70-256.74） | 366.92（286.76-442.70） | 143.42（110.18-174.44） | -1.49  (-1.63--1.36) | 354.81（140.49-575.73） | 707.50（283.53-1134.85） | 269.37（107.66-432.17） | -0.94  (-0.99--0.88) |
| South Asia | 1197.40（710.55-1755.95） | 6096.09（3097.74-9141.49） | 385.43（200.83-575.83） | 2.63  (2.54-2.72) | 1.73（0.73-3.42） | 13.66（6.80-24.12） | 0.90（0.44-1.57） | 3.86  (3.79-3.92) | 1.58（0.49-3.09） | 30.49（11.90-53.30） | 2.30（0.90-4.10） | 7.1  (6.88-7.33) | 50.14（-20.41-182.21） | 627.08（53.55-1331.28） | 38.97（3.49-82.45） | 5.22  (4.92-5.51) | 1.13（0.31-3.08） | 9.16（2.42-22.14） | 0.67（0.18-1.62） | 3.72  (3.61-3.83) | 399.11（230.38-577.00） | 1490.18（1049.06-2077.93） | 101.32（70.52-143.72） | 1.41  (1.35-1.46) | 743.71（260.03-1202.12） | 3925.51（1494.37-6266.03） | 241.27（91.27-384.42） | 2.93  (2.81-3.04) |
| Central Sub-Saharan Africa | 129.36（81.30-183.12） | 503.24（298.88-732.97） | 892.62（538.65-1285.60） | 1.44  (1.39-1.48) | 0.39（0.18-0.77） | 1.54（0.70-3.01） | 2.70（1.22-5.23） | 1.2  (0.94-1.47) | 0.20（0.07-0.39） | 2.22（0.81-4.06） | 5.08（1.85-9.34） | 5.33  (5.22-5.44) | 7.96（-0.16-22.27） | 65.15（3.58-148.61） | 104.28（6.01-237.00） | 3.54  (3.45-3.64) | 0.30（0.07-0.88） | 1.91（0.43-5.25） | 4.08（0.91-10.95） | 3  (2.64-3.36) | 89.50（45.61-137.88） | 292.00（171.82-431.34） | 546.75（322.13-825.58） | 1.03  (0.99-1.07) | 31.01（9.71-52.54） | 140.42（49.62-238.76） | 229.73（80.03-394.60） | 1.75  (1.63-1.87) |
| Southern Sub-Saharan Africa | 221.78（146.84-304.34） | 654.86（418.68-914.46） | 1116.80（720.36-1551.80） | 1.37(0.94-1.8) | 1.35（0.71-2.26） | 3.27（1.72-5.51） | 5.49（2.89-9.32） | -0.13(-0.43-0.17) | 1.44（0.60-2.41） | 6.61（2.82-11.34） | 13.80（5.94-23.50） | 2.72(2.5-2.95) | 39.51（2.01-83.54） | 127.86（4.93-266.21） | 208.22（9.65-435.40） | 1.76(1.33-2.2) | 0.88（0.20-2.33） | 4.33（1.09-10.85） | 7.71（1.99-19.35） | 2.93(2.7-3.17) | 116.31（95.26-146.33） | 328.75（265.70-399.28） | 578.12（437.99-722.53） | 1.27(0.82-1.73) | 62.28（25.13-100.44） | 184.04（76.84-296.97） | 303.46（126.23-495.55） | 1.26(0.87-1.66) |
| Eastern Sub-Saharan Africa | 315.75（209.63-413.92） | 882.36（596.59-1201.61） | 480.44（330.93-668.85） | 0.46  (0.37-0.54) | 0.70（0.32-1.39） | 3.17（1.39-6.33） | 1.69（0.75-3.34） | 1.85  (1.7-2.01) | 0.39（0.12-0.79） | 3.73（1.47-6.59） | 2.47（0.96-4.37） | 4.85  (4.79-4.91) | 15.71（-6.44-61.00） | 127.78（8.59-288.61） | 63.97（4.72-141.23） | 3.64  (3.46-3.82) | 0.24（0.06-0.73） | 1.82（0.41-5.18） | 1.25（0.29-3.50） | 3.78  (3.64-3.93) | 244.97（134.30-342.48） | 521.32（338.09-709.15） | 298.05（180.48-414.03） | -0.32  (-0.42--0.22) | 53.73（17.70-88.38） | 224.54（85.75-380.12） | 113.01（43.12-190.43） | 1.71  (1.62-1.8) |
| Western Sub-Saharan Africa | 443.16（298.87-621.75） | 1387.54（851.58-2036.08） | 652.42（400.58-946.98） | 0.84  (0.7-0.98) | 1.59（0.64-3.21） | 6.27（2.46-13.29） | 3.13（1.26-6.49） | 1.58  (1.46-1.71) | 1.38（0.54-2.41） | 8.39（3.41-14.02） | 5.90（2.42-9.83） | 3.2  (3.1-3.3) | 67.43（5.15-151.19） | 278.73（17.29-577.71） | 123.80（8.23-253.21） | 1.7  (1.57-1.83) | 0.76（0.16-2.31） | 4.64（1.05-11.94） | 2.88（0.64-7.45） | 3.23  (3.17-3.29) | 249.18（165.15-341.06） | 628.19（371.11-822.11） | 296.30（180.82-399.61） | 0.07  (-0.09-0.23) | 122.82（45.22-197.15） | 461.30（173.84-766.01） | 220.42（81.87-364.80） | 1.57  (1.42-1.72) |

CVD, cardiovascular disease; HBMI, high body mass index; EAPC, estimated annual percentage change; DALYs, disability-adjusted life years; ASDR, age-standardized DALYs (disability-adjusted life years) rate.

Table S3 Global and regional death of CVD attributable to HBMI in 1990 and 2021, and EAPC of ASMR from 1990 to 2021

|  | CVD | | | | Aortic aneurysm | | | | Atrial fibrillation and flutter | | | | Stroke | | | | Lower extremity peripheral arterial disease | | | | Hypertensive heart disease | | | | Ischemic heart disease | | | |
| --- | --- | --- | --- | --- | --- | --- | --- | --- | --- | --- | --- | --- | --- | --- | --- | --- | --- | --- | --- | --- | --- | --- | --- | --- | --- | --- | --- | --- |
| location | Death number in 1990 (×1000) | Death number in 2021 (×1000) | ASMR in 2021 (per 100,000) | EAPC, 1990–2021 | Death number in 1990 (×1000) | Death number in 2021 (×1000) | ASMR in 2021 (per 100,000) | EAPC, 1990–2021 | Death number in 1990 (×1000) | Death number in 2021 (×1000) | ASMR in 2021 (per 100,000) | EAPC, 1990–2021 | Death number in 1990 (×1000) | Death number in 2021 (×1000) | ASMR in 2021 (per 100,000) | EAPC, 1990–2021 | Death number in 1990 (×1000) | Death number in 2021 (×1000) | ASMR in 2021 (per 100,000) | EAPC, 1990–2021 | Death number in 1990 (×1000) | Death number in 2021 (×1000) | ASMR in 2021 (per 100,000) | EAPC, 1990–2021 | Death number in 1990 (×1000) | Death number in 2021 (×1000) | ASMR in 2021 (per 100,000) | EAPC, 1990–2021 |
| global | 863.07（477.90-1316.55） | 1904.24（1072.73-2864.24） | 22.77（12.87-34.24） | -0.35  (-0.41 to -0.3) | 6.43（3.45-10.80） | 11.54（6.20-19.52） | 0.14（0.07-0.23） | -1.36  (-1.52 to -1.21) | 5.72（2.35-9.91） | 27.24（11.75-46.61） | 0.35（0.15-0.60） | 1.65  (1.6 to 1.71) | 116.15（16.18-241.03） | 271.35（23.27-564.35） | 3.19（0.28-6.66） | -0.42  (-0.55 to -0.28) | 5.67（1.45-14.60） | 13.29（3.81-31.20） | 0.17（0.05-0.39） | -0.79  (-0.99 to -0.58) | 240.10（168.85-313.37） | 594.90（362.92-804.91） | 7.21（4.23-9.94） | 0.33  (0.27 to 0.39) | 489.00（188.71-807.45） | 985.92（378.30-1610.22） | 11.71（4.48-19.18） | -0.73  (-0.81 to -0.66) |
| SDI |  |  |  |  |  |  |  |  |  |  |  |  |  |  |  |  |  |  |  |  |  |  |  |  |  |  |  |  |
| High SDI | 277.60（453.03-132.42） | 358.20（548.40-198.07） | 16.03（24.47-9.08） | -1.62  (-1.73 to -1.51) | 4.33（7.27-2.29） | 4.92（8.24-2.57） | 0.22（0.37-0.12） | -2.4  (-2.61 to -2.19) | 3.34（5.95-1.38） | 13.04（22.83-5.61） | 0.49（0.86-0.21） | 1.5  (1.41 to 1.6) | 32.04（67.14-3.35） | 39.01（80.65-3.29） | 1.80（3.72-0.14） | -1.87  (-2.02 to -1.72) | 3.02（7.54-0.81） | 6.98（16.00-2.07） | 0.28（0.64-0.08） | -0.31  (-0.59 to -0.03) | 42.34（57.79-25.10） | 101.72（144.02-51.59） | 4.38（5.87-2.59） | 0.83  (0.69 to 0.98) | 192.53（319.33-74.11） | 192.54（318.34-76.13） | 8.85（14.39-3.55） | -2.5  (-2.62 to -2.38) |
| High-middle SDI | 288.82（140.67-460.61） | 546.18（277.70-863.73） | 28.29（14.36-44.72） | -0.77  (-0.99 to -0.55) | 1.45（0.80-2.50） | 3.42（1.81-5.80） | 0.17（0.09-0.29） | 0  (-0.16 to 0.17) | 1.70（0.71-2.82） | 7.21（3.11-12.45） | 0.39（0.17-0.67） | 1.47  (1.41 to 1.53) | 55.28（6.36-112.92） | 91.96（8.52-191.97） | 4.71（0.43-9.85） | -1.45  (-1.72 to -1.19) | 2.30（0.54-6.02） | 4.28（1.17-9.91） | 0.22（0.06-0.52） | -1.31  (-1.49 to -1.14) | 55.92（37.39-74.69） | 132.67（67.11-191.44） | 7.00（3.38-10.17） | 0.44  (0.26 to 0.62) | 172.18（66.39-281.26） | 306.64（118.00-511.49） | 15.80（6.08-26.38） | -1.05  (-1.31 to -0.78) |
| Middle SDI | 167.94（113.58-237.05） | 567.90（322.61-845.04） | 22.87（12.91-34.13） | 0.61  (0.53 to 0.69) | 0.42（0.23-0.71） | 2.05（1.11-3.46） | 0.08（0.04-0.14） | 1.49  (1.32 to 1.67) | 0.44（0.17-0.75） | 4.91（2.17-8.21） | 0.24（0.11-0.41） | 3.73  (3.6 to 3.85) | 16.47（1.66-39.72） | 85.28（6.84-181.31） | 3.26（0.27-6.92） | 1.96  (1.85 to 2.06) | 0.25（0.07-0.64） | 1.31（0.38-3.12） | 0.06（0.02-0.14） | 1.5  (1.4 to 1.61) | 80.18（52.53-106.38） | 196.80（122.49-276.06） | 8.30（4.53-12.23） | -0.44  (-0.63 to -0.26) | 70.17（26.78-113.66） | 277.54（108.45-453.73） | 10.94（4.23-18.04） | 1.22  (1.19 to 1.25) |
| Low-middle SDI | 94.73（60.10-134.04） | 333.45（193.98-489.32） | 24.60（14.77-35.86） | 1.29  (1.25 to 1.34) | 0.15（0.08-0.26） | 0.88（0.47-1.48） | 0.07（0.03-0.11） | 2.85  (2.79 to 2.91) | 0.20（0.08-0.35） | 1.78（0.76-2.96） | 0.18（0.08-0.30） | 4.03  (3.93 to 4.14) | 9.68（1.03-21.79） | 43.12（3.92-90.11） | 3.05（0.29-6.33） | 1.99  (1.91 to 2.07) | 0.06（0.02-0.18） | 0.51（0.14-1.21） | 0.05（0.01-0.11） | 3.57  (3.49 to 3.66) | 41.41（28.21-55.45） | 113.74（81.65-147.94） | 8.96（5.90-12.38） | 0.43  (0.39 to 0.47) | 43.23（16.57-69.90） | 173.42（67.96-277.13） | 12.30（4.74-19.84） | 1.85  (1.78 to 1.93) |
| Low SDI | 32.18（21.09-43.12） | 95.85（62.83-134.65） | 20.61（13.64-29.36） | 0.83  (0.75 to 0.92) | 0.06（0.03-0.13） | 0.25（0.12-0.49） | 0.06（0.03-0.11） | 1.53  (1.34 to 1.72) | 0.03（0.01-0.06） | 0.26（0.10-0.49） | 0.08（0.03-0.15） | 4.49  (4.23 to 4.75) | 2.37（0.11-6.10） | 11.62（0.92-24.48） | 2.28（0.20-4.77） | 2.33  (2.27 to 2.39) | 0.03（0.01-0.09） | 0.17（0.04-0.51） | 0.05（0.01-0.13） | 3.23  (2.97 to 3.49) | 19.84（11.28-27.74） | 49.04（31.97-64.99） | 11.27（6.94-15.88） | 0.3  (0.22 to 0.39) | 9.85（3.42-16.07） | 34.49（12.70-56.37） | 6.87（2.47-11.25） | 1.4  (1.3 to 1.49) |
| Region |  |  |  |  |  |  |  |  |  |  |  |  |  |  |  |  |  |  |  |  |  |  |  |  |  |  |  |  |
| East Asia | 90.90（64.32-126.19） | 364.66（194.22-581.29） | 18.60（10.02-29.02） | 1.2  (1.01 to 1.38) | 0.08（0.04-0.14） | 0.60（0.30-1.07） | 0.03（0.01-0.05） | 3.65  (3.55 to 3.76) | 0.06（0.01-0.13） | 2.54（1.00-4.45） | 0.16（0.06-0.28） | 7.96  (7.7 to 8.21) | 5.46（-1.57-21.53） | 72.36（6.16-160.78） | 3.41（0.30-7.42） | 5.03  (4.6 to 5.46) | 0.03（0.01-0.07） | 0.27（0.08-0.65） | 0.01（0.00-0.03） | 3.68  (3.55 to 3.82) | 60.55（35.30-84.65） | 129.83（67.19-204.26） | 6.89（3.18-11.52） | -1.11  (-1.52 to -0.69) | 24.72（8.36-41.68） | 159.06（56.61-268.21） | 8.11（2.87-13.74） | 3.33  (3.08 to 3.58) |
| Southeast Asia | 25.13（17.16-34.61） | 103.56（63.85-152.18） | 16.09（10.17-23.36） | 1.58  (1.49 to 1.68) | 0.05（0.02-0.08） | 0.30（0.16-0.50） | 0.05（0.03-0.09） | 2.88  (2.83 to 2.93) | 0.02（0.00-0.05） | 0.51（0.19-0.90） | 0.11（0.04-0.20） | 7.61  (7.32 to 7.9) | 1.59（-0.57-5.45） | 16.52（1.40-35.26） | 2.45（0.22-5.24） | 4.6  (4.19 to 5.02) | 0.00（0.00-0.01） | 0.04（0.01-0.09） | 0.01（0.00-0.02） | 3.84  (3.74 to 3.95) | 13.60（8.33-18.95） | 43.05（29.95-57.02） | 6.97（4.62-9.60） | 0.69  (0.63 to 0.75) | 9.87（3.50-15.66） | 43.14（16.23-69.83） | 6.50（2.43-10.51） | 1.86  (1.76 to 1.97) |
| Oceania | 1.03（0.54-1.68） | 2.90（1.41-4.75） | 36.57（18.30-59.43） | 0.19  (0.15 to 0.23) | 0.00（0.00-0.01） | 0.01（0.00-0.02） | 0.14（0.07-0.24） | 0.37  (0.29 to 0.46) | 0.00（0.00-0.01） | 0.01（0.01-0.02） | 0.27（0.12-0.49） | 1.52  (1.44 to 1.59) | 0.15（0.01-0.38） | 0.48（0.01-1.12） | 5.64（0.24-12.89） | 0.5  (0.44 to 0.57) | 0.00（0.00-0.00） | 0.00（0.00-0.00） | 0.03（0.01-0.06） | 1.35  (0.87 to 1.82) | 0.31（0.20-0.43） | 0.68（0.48-1.00） | 9.27（6.25-13.18） | -0.75  (-0.81 to -0.7) | 0.55（0.21-0.93） | 1.71（0.71-2.89） | 21.23（8.54-36.33） | 0.58  (0.52 to 0.65) |
| Central Europe | 81.72（40.10-131.70） | 100.60（57.79-154.55） | 43.16（24.92-66.31） | -1.13  (-1.21 to -1.04) | 0.43（0.23-0.74） | 0.73（0.38-1.27） | 0.32（0.17-0.56） | -0.05  (-0.26 to 0.17) | 0.57（0.25-0.98） | 1.46（0.65-2.58） | 0.60（0.27-1.06） | 0.86  (0.62 to 1.09) | 15.35（1.40-32.34） | 14.18（1.52-29.55） | 6.12（0.62-12.82） | -2.31  (-2.5 to -2.12) | 0.69（0.18-1.60） | 1.71（0.52-3.71） | 0.72（0.22-1.55） | 0.62  (0.39 to 0.85) | 16.69（11.46-22.13） | 34.42（18.12-47.35） | 14.52（7.94-19.75） | 1.11  (0.85 to 1.37) | 47.99（18.59-78.72） | 48.10（19.02-81.10） | 20.88（8.29-35.12） | -1.95  (-2.06 to -1.84) |
| Central Asia | 23.15（10.11-37.01） | 40.40（19.54-63.84） | 56.25（27.49-89.36） | -0.17  (-0.55 to 0.2) | 0.04（0.02-0.06） | 0.15（0.08-0.26） | 0.20（0.11-0.34） | 3.05  (2.84 to 3.26) | 0.06（0.03-0.10） | 0.16（0.07-0.28） | 0.27（0.12-0.46） | 1.47  (1.24 to 1.7) | 3.69（0.25-8.01） | 6.01（0.46-12.88） | 7.88（0.65-17.20） | -0.66  (-1.04 to -0.29) | 0.01（0.00-0.03） | 0.05（0.01-0.12） | 0.07（0.02-0.18） | 3.54  (3.29 to 3.79) | 3.55（2.50-4.65） | 7.83（5.33-10.30） | 11.36（7.06-15.74） | 1.39  (0.78 to 2.01) | 15.81（6.12-25.73） | 26.21（10.46-42.73） | 36.47（14.37-60.15） | -0.52  (-0.87 to -0.18) |
| Eastern Europe | 135.17（47.18-229.42） | 196.34（78.77-327.89） | 55.57（22.28-92.96） | -0.33  (-0.86 to 0.2) | 0.60（0.32-1.00） | 1.53（0.80-2.58） | 0.44（0.23-0.73） | 1.94  (1.62 to 2.25) | 0.78（0.33-1.30） | 2.28（0.98-3.92） | 0.63（0.27-1.09） | 1.69  (1.52 to 1.87) | 30.66（3.45-61.50） | 33.18（3.43-67.87） | 9.44（0.95-19.26） | -1.52  (-2.04 to -0.99) | 1.34（0.29-3.61） | 2.07（0.52-5.03） | 0.57（0.14-1.38） | -0.39  (-0.72 to -0.06) | 6.65（5.13-8.09） | 15.86（10.10-21.12） | 4.49（2.96-5.91） | 1.92  (1 to 2.85) | 95.14（35.99-156.28） | 141.41（54.35-232.47） | 40.00（15.37-65.70） | -0.26  (-0.78 to 0.26) |
| High-income Asia Pacific | 11.73（6.90-17.59） | 19.56（10.44-29.96） | 3.47（1.80-5.32） | -1.98  (-2.29 to -1.68) | 0.21（0.11-0.35） | 1.07（0.53-1.73） | 0.19（0.10-0.31） | 1.8  (1.7 to 1.89) | 0.03（0.01-0.07） | 0.34（0.12-0.65） | 0.05（0.02-0.10） | 2.83  (2.5 to 3.15) | 1.63（0.23-3.78） | 2.74（0.35-6.00） | 0.52（0.06-1.16） | -2.17  (-2.34 to -2.01) | 0.06（0.02-0.15） | 0.33（0.10-0.76） | 0.05（0.01-0.11） | 1.14  (0.78 to 1.5) | 4.66（2.31-7.15） | 6.72（2.09-11.98） | 1.00（0.42-1.66） | -2.93  (-3.67 to -2.19) | 5.13（1.79-8.42） | 8.35（3.00-13.58） | 1.66（0.61-2.66） | -1.65  (-1.77 to -1.53) |
| Australasia | 5.73（2.36-9.63） | 6.78（3.17-11.29） | 11.42（5.22-18.93） | -2.71  (-2.82 to -2.61) | 0.15（0.08-0.26） | 0.14（0.07-0.25） | 0.25（0.13-0.43） | -3.53  (-3.7 to -3.35) | 0.10（0.04-0.18） | 0.56（0.23-1.04） | 0.86（0.36-1.59） | 2.13  (1.89 to 2.38) | 0.59（0.05-1.22） | 0.81（0.07-1.77） | 1.37（0.10-2.99） | -2.25  (-2.35 to -2.16) | 0.13（0.03-0.32） | 0.32（0.09-0.64） | 0.49（0.14-0.98） | -0.86  (-0.99 to -0.72) | 0.36（0.20-0.51） | 0.84（0.34-1.22） | 1.34（0.62-1.90） | -0.47  (-0.87 to -0.07) | 4.42（1.69-7.34） | 4.11（1.65-6.82） | 7.12（2.91-11.66） | -3.43  (-3.53 to -3.34) |
| Southern Latin America | 13.86（7.19-22.10） | 18.34（10.43-27.60） | 20.44（11.55-30.74） | -1.09  (-1.19 to -1) | 0.15（0.08-0.26） | 0.25（0.13-0.44） | 0.28（0.15-0.49） | -0.5  (-0.75 to -0.26) | 0.10（0.04-0.18） | 0.42（0.18-0.76） | 0.45（0.19-0.81） | 2.65  (2.23 to 3.07) | 2.46（0.14-5.40） | 2.55（0.15-5.38） | 2.90（0.16-6.11） | -1.91  (-2.01 to -1.81) | 0.04（0.01-0.09） | 0.11（0.03-0.25） | 0.12（0.03-0.27） | 1.33  (0.72 to 1.95) | 3.50（2.28-4.66） | 7.12（3.48-10.12） | 7.75（3.88-10.95） | 0.21  (0.05 to 0.36) | 7.62（2.98-12.64） | 7.90（3.11-12.93） | 8.95（3.54-14.62） | -1.85  (-1.98 to -1.73) |
| High-income North America | 106.55（50.48-174.89） | 161.24（91.77-242.30） | 24.08（13.78-35.84） | -0.99  (-1.13 to -0.84) | 1.64（0.85-2.85） | 1.45（0.75-2.46） | 0.22（0.11-0.37） | -3.12  (-3.44 to -2.81) | 1.24（0.50-2.41） | 5.73（2.43-9.90） | 0.76（0.32-1.31） | 2.5  (2.35 to 2.66) | 8.39（0.68-18.06） | 14.79（0.99-30.56） | 2.22（0.13-4.55） | -0.63  (-0.85 to -0.42) | 1.39（0.38-3.34） | 3.29（1.03-6.89） | 0.46（0.14-0.95） | -0.23  (-0.62 to 0.17) | 13.62（9.18-17.79） | 44.70（28.22-58.03） | 6.81（4.69-8.59） | 2.04  (1.89 to 2.2) | 80.27（31.34-134.07） | 91.28（37.14-149.62） | 13.61（5.58-22.07） | -2.03  (-2.18 to -1.88) |
| Caribbean | 6.23（3.49-9.09） | 15.01（8.90-22.65） | 27.56（16.26-41.59） | 0.43  (0.27 to 0.6) | 0.04（0.02-0.07） | 0.10（0.05-0.17） | 0.18（0.09-0.30） | -0.09  (-0.25 to 0.07) | 0.04（0.02-0.06） | 0.22（0.10-0.36） | 0.39（0.18-0.65） | 2.42  (2.32 to 2.52) | 0.70（0.07-1.50） | 1.88（0.12-3.95） | 3.49（0.22-7.34） | 0.81  (0.73 to 0.89) | 0.03（0.01-0.08） | 0.18（0.05-0.42） | 0.32（0.10-0.74） | 2.57  (2.45 to 2.69) | 1.90（1.37-2.47） | 5.46（3.58-7.23） | 9.98（6.59-13.15） | 1.29  (1.07 to 1.52) | 3.52（1.36-5.56） | 7.17（2.73-11.99） | 13.19（5.04-22.07） | -0.27  (-0.47 to -0.08) |
| Western Europe | 140.81（66.22-227.54） | 144.49（78.12-225.16） | 12.63（6.75-20.00） | -2.11  (-2.18 to -2.05) | 2.46（1.32-4.11） | 2.34（1.21-4.09） | 0.23（0.12-0.39） | -2.53  (-2.77 to -2.29) | 2.05（0.86-3.50） | 7.15（2.95-12.84） | 0.55（0.23-1.00） | 1.68  (1.58 to 1.77) | 19.55（2.10-40.78） | 14.56（1.32-32.45） | 1.34（0.11-2.92） | -3.14  (-3.26 to -3.02) | 1.63（0.42-4.37） | 3.02（0.84-7.56） | 0.25（0.07-0.62） | -0.21  (-0.56 to 0.13) | 23.44（11.49-34.45） | 52.47（16.49-84.21） | 4.11（1.50-6.37） | 0.72  (0.54 to 0.91) | 91.67（34.74-153.38） | 64.95（24.69-109.62） | 6.16（2.38-10.32） | -3.28  (-3.38 to -3.18) |
| Andean Latin America | 3.26（1.79-5.06） | 8.65（4.69-13.66） | 14.90（8.16-23.54） | -0.26  (-0.46 to -0.07) | 0.01（0.00-0.02） | 0.04（0.02-0.07） | 0.07（0.04-0.13） | 1.88  (1.71 to 2.05) | 0.02（0.01-0.04） | 0.18（0.07-0.33） | 0.33（0.13-0.60） | 3.31  (3.15 to 3.48) | 0.54（0.03-1.22） | 1.42（0.06-3.01） | 2.37（0.10-5.02） | -0.46  (-0.63 to -0.29) | 0.00（0.00-0.00） | 0.01（0.00-0.03） | 0.02（0.01-0.06） | 5.4  (4.99 to 5.81) | 1.09（0.74-1.48） | 2.48（1.38-3.57） | 4.38（2.37-6.42） | -0.33  (-0.74 to 0.09) | 1.59（0.60-2.65） | 4.51（1.75-7.57） | 7.72（2.97-13.00） | -0.33  (-0.7 to 0.05) |
| Central Latin America | 18.92（10.90-28.64） | 60.77（31.55-94.87） | 24.93（13.01-39.08） | -0.21  (-0.43 to 0.01) | 0.08（0.04-0.13） | 0.33（0.17-0.56） | 0.14（0.07-0.23） | 0.31  (0.05 to 0.57) | 0.16（0.06-0.29） | 1.24（0.55-2.21） | 0.54（0.24-0.96） | 2.39  (2.3 to 2.49) | 2.40（0.15-5.19） | 6.63（0.32-14.14） | 2.66（0.14-5.65） | -0.64  (-0.8 to -0.48) | 0.07（0.02-0.16） | 0.20（0.06-0.47） | 0.09（0.02-0.20） | -1.1  (-1.44 to -0.77) | 5.93（4.04-7.93） | 12.77（7.67-17.93） | 5.40（3.14-7.69） | -1.55  (-1.76 to -1.34) | 10.29（4.02-16.93） | 39.59（16.10-65.23） | 16.10（6.54-26.64） | 0.45  (0.21 to 0.69) |
| North Africa and Middle East | 94.74（55.89-140.51） | 272.09（153.28-400.69） | 67.54（38.92-100.73） | 0.2  (0.11 to 0.29) | 0.08（0.04-0.14） | 0.42（0.22-0.74） | 0.10（0.05-0.17） | 2.4  (2.31 to 2.5) | 0.25（0.10-0.44） | 1.66（0.70-2.81） | 0.56（0.24-0.96） | 2.91  (2.68 to 3.15) | 12.42（1.06-27.10） | 36.07（3.47-72.73） | 8.50（0.89-17.36） | 0.19  (0.12 to 0.26) | 0.04（0.01-0.10） | 0.26（0.08-0.53） | 0.07（0.02-0.15） | 3.6  (3.31 to 3.88) | 35.44（24.34-47.30） | 85.25（57.66-110.09） | 23.20（14.02-31.44） | -0.29  (-0.45 to -0.13) | 46.52（18.42-75.43） | 148.43（61.42-236.86） | 35.10（14.11-56.38） | 0.53  (0.49 to 0.58) |
| Tropical Latin America | 25.99（14.30-39.94） | 54.96（31.08-84.78） | 21.68（12.30-33.27） | -1.01  (-1.06 to -0.95) | 0.20（0.11-0.33） | 0.97（0.50-1.70） | 0.38（0.20-0.67） | 1.49  (1.2 to 1.77) | 0.12（0.04-0.21） | 1.27（0.51-2.20） | 0.53（0.21-0.92） | 3.45  (3.19 to 3.7) | 5.00（0.33-10.90） | 8.98（0.53-19.39） | 3.51（0.22-7.56） | -1.54  (-1.6 to -1.48) | 0.12（0.03-0.32） | 0.72（0.20-1.63） | 0.29（0.08-0.66） | 1.63  (1.43 to 1.84) | 8.42（6.36-10.54） | 17.13（10.98-22.65） | 6.90（4.29-9.26） | -1.15  (-1.3 to -1.01) | 12.14（4.71-19.75） | 25.90（10.08-42.18） | 10.07（3.90-16.43） | -0.95  (-1 to -0.9) |
| South Asia | 38.70（23.19-56.43） | 210.87（115.25-313.44） | 14.79（8.31-22.09） | 2.64  (2.55 to 2.74) | 0.06（0.03-0.13） | 0.55（0.27-0.95） | 0.04（0.02-0.07） | 3.94  (3.84 to 4.03) | 0.03（0.01-0.07） | 0.82（0.35-1.51） | 0.08（0.03-0.15） | 8.07  (7.86 to 8.29) | 1.56（-0.49-5.22） | 19.56（1.87-41.63） | 1.33（0.13-2.82） | 4.89  (4.63 to 5.15) | 0.02（0.00-0.05） | 0.21（0.06-0.54） | 0.02（0.00-0.05） | 4.94  (4.81 to 5.07) | 14.75（8.20-21.66） | 61.43（40.11-90.56） | 4.73（2.81-7.34） | 1.58  (1.5 to 1.67) | 22.29（7.69-35.89） | 128.29（48.00-206.54） | 8.60（3.18-13.92） | 3.06  (2.94 to 3.18) |
| Central Sub-Saharan Africa | 4.62（2.92-6.58） | 18.19（10.86-26.01） | 40.35（25.53-59.34） | 1.61  (1.57 to 1.65) | 0.01（0.01-0.03） | 0.06（0.03-0.11） | 0.12（0.06-0.23） | 1.17  (0.89 to 1.45) | 0.01（0.00-0.01） | 0.07（0.02-0.14） | 0.21（0.07-0.42） | 5.88  (5.69 to 6.06) | 0.26（0.00-0.69） | 2.01（0.11-4.61） | 3.89（0.23-8.74） | 3.57  (3.48 to 3.65) | 0.01（0.00-0.03） | 0.07（0.02-0.20） | 0.19（0.04-0.52） | 3.23  (2.82 to 3.65) | 3.26（1.62-4.94） | 11.19（6.48-17.05） | 26.46（14.82-40.53） | 1.32  (1.28 to 1.35) | 1.07（0.33-1.81） | 4.79（1.68-8.24） | 9.48（3.23-16.09） | 1.83  (1.71 to 1.95) |
| Southern Sub-Saharan Africa | 7.80（5.36-10.73） | 25.24（16.77-35.04） | 50.54（33.10-71.27） | 1.68  (1.23 to 2.12) | 0.05（0.03-0.09） | 0.13（0.07-0.22） | 0.25（0.13-0.44） | -0.22  (-0.55 to 0.11) | 0.04（0.02-0.06） | 0.21（0.09-0.34） | 0.57（0.26-0.94） | 3.51  (3.11 to 3.91) | 1.14（0.06-2.51） | 4.30（0.21-9.06） | 8.00（0.48-16.88） | 2.27  (1.77 to 2.78) | 0.03（0.01-0.08） | 0.16（0.04-0.40） | 0.33（0.09-0.82） | 3.32  (3.05 to 3.59) | 4.45（3.19-5.89） | 13.65（9.93-17.28） | 28.40（17.52-37.54） | 1.56  (1.1 to 2.01) | 2.09（0.84-3.42） | 6.79（2.81-11.12） | 12.99（5.31-21.35） | 1.56  (1.16 to 1.96) |
| Eastern Sub-Saharan Africa | 10.90（7.21-14.51） | 30.69（21.26-42.92） | 20.72（14.10-29.08） | 0.59  (0.51 to 0.66) | 0.03（0.01-0.05） | 0.11（0.05-0.22） | 0.07（0.03-0.14） | 1.78  (1.62 to 1.95) | 0.01（0.00-0.02） | 0.09（0.03-0.17） | 0.08（0.03-0.15） | 5.25  (5.13 to 5.36) | 0.45（-0.18-1.68） | 3.70（0.24-8.17） | 2.19（0.17-4.70） | 3.83  (3.65 to 4.01) | 0.01（0.00-0.03） | 0.07（0.02-0.19） | 0.06（0.01-0.16） | 3.94  (3.76 to 4.13) | 8.70（4.74-12.35） | 19.48（11.56-27.47） | 13.95（7.65-21.25） | -0.04  (-0.13 to 0.05) | 1.71（0.56-2.77） | 7.24（2.76-12.21） | 4.36（1.63-7.33） | 1.89  (1.81 to 1.97) |
| Western Sub-Saharan Africa | 16.12（10.94-22.04） | 48.90（30.11-71.04） | 28.09（17.11-40.94） | 0.9  (0.76 to 1.03) | 0.07（0.03-0.13） | 0.25（0.10-0.52） | 0.15（0.06-0.31） | 1.63  (1.51 to 1.75) | 0.05（0.02-0.09） | 0.30（0.13-0.52） | 0.30（0.12-0.51） | 3.08  (2.92 to 3.23) | 2.18（0.18-4.71） | 8.60（0.54-17.69） | 4.63（0.33-9.27） | 1.75  (1.63 to 1.86) | 0.03（0.01-0.09） | 0.20（0.04-0.53） | 0.15（0.03-0.40） | 3.43  (3.35 to 3.51) | 9.23（6.02-12.93） | 22.55（13.46-30.63） | 13.03（7.79-18.62） | 0.1  (-0.05 to 0.26) | 4.56（1.69-7.37） | 16.99（6.29-28.15） | 9.84（3.56-16.32） | 1.74  (1.61 to 1.88) |

CVD, cardiovascular disease; HBMI, high body mass index; EAPC, estimated annual percentage change; ASMR, age-standardized mortality rate.

Table S4. DALYs of CVD attributable to HBMI in 1990 and 2021, and EAPC of ASDR from 1990 to 2021 in 204 countries and territories

|  | CVD | | | | Aortic aneurysm | | | | Atrial fibrillation and flutter | | | | Stroke | | | | Lower extremity peripheral arterial disease | | | | Hypertensive heart disease | | | | Ischemic heart disease | | | |
| --- | --- | --- | --- | --- | --- | --- | --- | --- | --- | --- | --- | --- | --- | --- | --- | --- | --- | --- | --- | --- | --- | --- | --- | --- | --- | --- | --- | --- |
| location | DALY number in 1990 | DALY number in 2021 | ASDR in 2021 (per 100,000) | EAPC, 1990–2021 | DALY number in 1990 | DALY number in 2021 | ASDR in 2021 | EAPC, 1990–2021 | DALY number in 1990 | DALY number in 2021 | ASDR in 2021 (per 100,000) | EAPC, 1990–2021 | DALY number in 1990 | DALY number in 2021 | ASDR in 2021 | EAPC, 1990–2021 | DALY number in 1990 | DALY number in 2021 | ASDR in 2021 (per 100,000) | EAPC, 1990–2021 | DALY number in 1990 | DALY number in 2021 | ASDR in 2021 (per 100,000) | EAPC, 1990–2021 | DALY number in 1990 | DALY number in 2021 | ASDR in 2021 (per 100,000) | EAPC, 1990–2021 |
| Afghanistan | 140679.45（73520.21-222189.63） | 232522.21（121687.02-360313.60） | 1940.30（1061.27-2987.70） | -0.19   (-0.3--0.07) | 8.20（3.23-17.11） | 66.20（28.44-137.76） | 0.52（0.24-1.01） | 5.74   (5.45-6.03) | 150.83（51.71-299.03） | 413.38（160.74-738.56） | 5.14（1.98-9.27） | 2.79   (2.67-2.92) | 16673.78（1117.29-37014.04） | 34892.59（2194.26-74796.61） | 260.37（19.56-562.48） | 0.22   (0.04-0.4) | 26.79（5.58-70.45） | 65.33（15.04-159.46） | 0.88（0.21-2.15） | 2.86   (2.5-3.23) | 61150.83（21259.22-104982.29） | 83305.43（38781.64-135678.17） | 802.84（387.04-1302.15） | -0.37   (-0.51--0.23) | 62669.02（23236.71-106776.32） | 113779.27（45050.43-199069.69） | 870.56（337.89-1520.80） | -0.14   (-0.25--0.02) |
| Albania | 14148.07（6119.09-23897.40） | 26711.55（11112.84-45881.41） | 632.64（263.98-1090.62） | -0.09   (-0.28-0.1) | 34.01（17.54-58.79） | 103.24（45.49-202.10） | 2.40（1.05-4.70） | 1.27   (1.13-1.41) | 141.31（52.26-264.52） | 525.14（203.17-1042.62） | 12.20（4.70-24.34） | 1.44   (1.33-1.56) | 3110.34（152.31-7158.26） | 5451.39（182.84-12772.11） | 128.68（4.30-302.00） | -0.46   (-0.66--0.26) | 25.49（5.50-66.65） | 102.58（23.53-225.42） | 2.30（0.53-5.05） | 1.64   (1.56-1.72) | 2391.94（1521.42-3435.51） | 3921.05（2159.88-6239.47） | 92.36（49.77-146.75） | -0.65   (-0.88--0.42) | 8444.98（3252.39-14195.35） | 16608.15（6263.33-27708.10） | 394.71（148.72-658.55） | 0.14   (-0.05-0.33) |
| Algeria | 133329.96（81232.68-203416.46） | 406650.54（229501.05-599635.20） | 1196.61（691.98-1784.39） | 0.15   (0.1-0.2) | 26.19（12.33-49.89） | 307.98（150.06-554.63） | 0.82（0.40-1.47） | 5.26   (4.98-5.54) | 368.08（136.63-709.35） | 3222.20（1326.11-5945.63） | 12.19（5.16-22.43） | 4.12   (3.96-4.28) | 15706.37（1408.56-35585.98） | 57510.14（5313.46-119187.18） | 156.66（15.60-331.47） | 0.66   (0.61-0.71) | 75.31（15.57-192.56） | 460.66（113.22-1050.76） | 1.41（0.35-3.25） | 2.48   (2.34-2.62) | 53557.44（34555.70-79683.63） | 145615.51（89971.19-210695.50） | 459.09（268.54-676.13） | 0.08   (-0.04-0.2) | 63596.57（23441.22-107204.16） | 199534.05（81392.33-334049.32） | 566.44（229.35-950.33） | 0.02   (-0.09-0.14) |
| American Samoa | 448.24（186.52-695.83） | 931.81（417.48-1464.44） | 1797.45（802.81-2827.73） | 0.38   (0.28-0.48) | 2.26（1.23-3.85） | 4.24（2.23-6.95） | 8.53（4.47-13.95） | -0.44   (-0.65--0.23) | 3.73（1.58-6.25） | 12.51（5.70-20.70） | 28.01（12.58-47.89） | 1.7   (1.65-1.75) | 114.18（-1.44-228.82） | 194.90（-1.70-373.68） | 376.25（-2.53-725.86） | -0.22   (-0.36--0.08) | 1.71（0.59-3.26） | 4.56（1.66-7.77） | 9.50（3.36-16.97） | 0.56   (0.03-1.09) | 70.18（50.94-87.41） | 111.20（86.24-141.14） | 219.90（168.73-278.56） | -1   (-1.27--0.74) | 256.17（117.34-389.68） | 604.40（287.80-936.60） | 1155.26（544.30-1795.91） | 0.92   (0.84-1.01) |
| Andorra | 177.14（84.26-302.16） | 317.16（160.84-549.32） | 197.79（99.08-347.53） | -1.34   (-1.54--1.13) | 5.44（2.54-10.33） | 9.06（4.07-17.12） | 5.88（2.64-11.17） | -1.4   (-1.62--1.17) | 3.77（1.33-7.36） | 14.33（5.24-27.77） | 8.74（3.21-17.02） | 0.73   (0.64-0.82) | 20.66（1.77-48.11） | 37.32（3.18-84.04） | 24.69（2.01-55.93） | -1.09   (-1.29--0.9) | 2.90（0.70-7.39） | 7.62（1.93-17.53） | 4.59（1.15-10.49） | -0.48   (-0.66--0.31) | 39.92（23.52-60.79） | 92.14（44.53-151.02） | 53.72（28.77-87.03） | -0.9   (-1.08--0.72) | 104.44（37.60-185.48） | 156.69（59.40-285.16） | 100.18（38.18-182.10） | -1.75   (-1.98--1.52) |
| Angola | 24956.75（15376.50-36428.62） | 97892.98（60658.14-145880.35） | 772.91（486.84-1118.73） | 0.59   (0.48-0.71) | 64.59（29.62-126.51） | 374.33（184.51-692.66） | 2.92（1.38-5.49） | 1.85   (1.72-1.97) | 18.92（4.52-41.39） | 326.47（122.87-609.54） | 3.09（1.13-5.88） | 6.17   (6.08-6.26) | 1048.22（-341.23-3755.36） | 11889.47（788.01-27193.81） | 83.21（5.84-188.66） | 3.67   (3.48-3.86) | 23.27（6.45-69.62） | 225.99（56.06-598.87） | 2.18（0.55-5.63） | 3.72   (3.53-3.92) | 18801.53（9617.17-28056.16） | 57503.06（36082.38-83904.13） | 480.53（299.48-700.29） | -0.08   (-0.19-0.02) | 5000.23（1591.48-8684.85） | 27573.65（9706.34-47672.35） | 200.97（70.62-350.13） | 1.68   (1.57-1.8) |
| Antigua and Barbuda | 391.55（252.78-567.40） | 856.92（615.77-1164.52） | 808.58（578.82-1096.56） | 0.28   (-0.04-0.6) | 1.81（0.98-3.14） | 3.30（1.68-5.64） | 3.08（1.59-5.27） | -0.85   (-1.12--0.58) | 3.30（1.25-5.95） | 12.93（5.07-23.34） | 13.34（5.18-24.36） | 2.58   (2.45-2.72) | 55.45（3.20-120.98） | 109.89（4.25-228.32） | 99.88（4.21-208.01） | -0.45   (-0.64--0.26) | 1.49（0.45-3.83） | 4.93（1.44-11.56） | 4.91（1.44-11.57） | 1.96   (1.76-2.15) | 184.58（138.63-235.21） | 508.52（404.63-612.92） | 486.53（370.60-600.67） | 1.46   (1-1.92) | 144.91（57.18-238.17） | 217.34（88.91-349.54） | 200.83（81.28-322.94） | -1.45   (-1.64--1.25) |
| Argentina | 246625.17（119853.31-395576.93） | 261466.58（145562.30-394247.12） | 472.44（259.59-714.91） | -1.35   (-1.45--1.24) | 2462.27（1311.12-4273.34） | 3428.15（1739.67-5996.62） | 6.20（3.15-10.84） | -0.61   (-0.83--0.39) | 2004.79（781.88-3631.35） | 5542.08（2207.61-10135.87） | 9.60（3.83-17.48） | 1.67   (1.42-1.91) | 50245.47（2015.82-111043.96） | 47179.83（1723.25-98700.04） | 87.49（2.94-183.63） | -1.85   (-1.98--1.72) | 849.08（207.76-2047.37） | 2026.63（549.66-4356.95） | 3.51（0.95-7.49） | 1.03   (0.83-1.24) | 55802.30（43117.49-68642.90） | 79771.39（53240.29-103843.16） | 139.87（95.04-180.37） | -0.44   (-0.61--0.28) | 135261.25（54015.87-222684.71） | 123518.49（50248.00-198756.80） | 225.78（92.20-362.48） | -1.75   (-1.88--1.62) |
| Armenia | 30643.56（13456.90-48873.44） | 38304.84（18364.64-61494.02） | 897.00（429.85-1438.42） | -1.25   (-1.44--1.06) | 268.68（140.98-465.44） | 843.95（434.69-1443.74） | 19.42（10.03-33.49） | 2.64   (2.32-2.96) | 149.72（58.17-272.84） | 423.91（176.05-758.67） | 9.66（3.97-17.33） | 1.65   (1.49-1.81) | 4670.10（371.01-9826.33） | 4903.49（554.83-10101.64） | 115.30（12.91-238.62） | -2.13   (-2.45--1.81) | 132.04（30.95-374.96） | 324.71（79.81-826.35） | 7.43（1.81-18.87） | 1.4   (1.09-1.72) | 4511.12（3256.72-5858.57） | 6286.21（4068.22-8708.55） | 145.55（95.58-201.08） | -0.34   (-0.65--0.04) | 20911.89（8185.47-34040.25） | 25522.56（10242.80-41598.16） | 599.64（240.91-979.17） | -1.43   (-1.62--1.24) |
| Australia | 99417.67（39679.14-166585.51） | 105866.40（47142.83-174759.08） | 240.98（105.93-395.94） | -2.59   (-2.73--2.45) | 2183.52（1162.77-3868.12） | 1892.50（1008.78-3261.25） | 4.21（2.27-7.23） | -3.68   (-3.87--3.48) | 2125.21（828.25-4052.46） | 10663.89（4620.66-18754.50） | 21.73（9.39-38.12） | 2.23   (2.03-2.42) | 11349.70（897.67-23581.26） | 15999.86（1182.96-33531.18） | 38.37（2.51-79.05） | -1.55   (-1.65--1.46) | 1910.34（480.06-4647.54） | 4050.78（1297.34-7915.58） | 7.87（2.57-15.24） | -0.96   (-1.07--0.85) | 5140.07（3610.53-6736.78） | 11620.86（7093.09-15465.92） | 24.76（16.42-31.56） | -0.05   (-0.49-0.38) | 76708.83（29828.15-126176.13） | 61638.52（25432.76-100486.66） | 144.04（59.23-231.92） | -3.47   (-3.59--3.35) |
| Austria | 61079.22（28081.37-100098.94） | 55516.87（29124.43-88653.94） | 286.79（154.70-459.28） | -2.09   (-2.27--1.91) | 595.95（320.03-1020.37） | 551.22（279.11-940.31） | 3.12（1.59-5.29） | -1.82   (-2.02--1.62) | 997.24（393.61-1805.66） | 3404.06（1391.62-6439.31） | 16.34（6.57-30.83） | 2.46   (2.23-2.68) | 8504.60（851.32-18091.58） | 5201.45（454.88-11257.01） | 30.06（2.42-65.30） | -3.12   (-3.41--2.83) | 810.27（168.44-2345.69） | 1239.25（288.99-3051.18） | 5.97（1.41-14.71） | 0.07   (-0.57-0.71) | 9464.64（5951.69-12649.94） | 17158.84（7300.55-25909.46） | 78.65（40.80-113.33） | 0.8   (0.49-1.11) | 40706.53（15279.90-67072.87） | 27962.04（10930.74-47350.08） | 152.66（59.87-254.72） | -3.17   (-3.42--2.93) |
| Azerbaijan | 74302.59（34690.03-119186.02） | 131014.30（58714.17-212541.30） | 1285.60（591.72-2084.94） | -0.58   (-0.83--0.33) | 76.23（39.61-131.29） | 382.56（156.11-777.94） | 3.43（1.49-6.76） | 3.2   (2.92-3.48) | 232.58（91.69-427.96） | 788.44（312.02-1453.74） | 8.60（3.37-15.50） | 1.73   (1.62-1.84) | 9075.27（456.46-20233.42） | 16800.42（793.16-37675.19） | 153.95（7.77-343.16） | -0.54   (-0.84--0.24) | 50.37（9.92-138.63） | 209.03（46.58-541.87） | 2.20（0.49-5.77） | 2.54   (2.38-2.69) | 13915.95（9705.80-18930.30） | 22172.83（14660.53-32322.51） | 228.30（147.87-332.85） | -0.55   (-0.81--0.29) | 50952.19（20071.76-85030.70） | 90661.01（37784.71-154806.80） | 889.12（363.74-1520.81） | -0.63   (-0.88--0.38) |
| Bahamas | 2081.45（1446.77-2867.46） | 5645.52（3945.92-7748.83） | 1330.39（942.50-1823.40） | 0.37   (0.24-0.49) | 9.33（4.92-16.19） | 25.29（12.62-45.01） | 6.11（3.03-10.90） | 0.04   (-0.3-0.38) | 12.36（4.90-21.93） | 63.76（25.90-116.50） | 17.04（6.75-31.29） | 2.22   (2.07-2.37) | 223.32（8.18-497.46） | 542.74（11.28-1115.89） | 123.51（3.17-256.11） | -0.13   (-0.2--0.05) | 7.02（1.97-17.09） | 30.70（9.33-70.91） | 7.83（2.36-18.51） | 1.78   (1.67-1.89) | 1122.47（917.70-1330.00） | 3568.05（2700.59-4475.53） | 851.26（632.91-1078.31） | 0.98   (0.77-1.19) | 706.96（286.67-1133.84） | 1414.98（583.15-2298.28） | 324.64（133.02-530.46） | -0.83   (-1.01--0.66) |
| Bahrain | 3098.92（1440.39-4937.31） | 9617.34（4635.35-14755.10） | 1003.22（526.70-1541.98） | -2.11   (-2.39--1.84) | 2.50（1.25-4.36） | 21.51（11.04-37.29） | 1.88（0.95-3.25） | 1.75   (1.41-2.1) | 9.36（3.40-18.53） | 89.40（37.15-162.05） | 16.90（6.33-32.41） | 2.14   (1.86-2.41) | 375.55（23.15-781.36） | 1557.66（109.09-3059.64） | 144.36（13.42-292.02） | -1.31   (-1.66--0.96) | 2.71（0.83-6.34） | 66.42（21.35-123.26） | 9.37（2.87-17.42） | 6.95   (6.06-7.85) | 491.37（369.76-624.61） | 1705.54（1207.78-2365.79） | 249.19（149.96-358.19） | -1.24   (-1.43--1.04) | 2217.43（913.56-3597.54） | 6176.81（2618.83-9790.35） | 581.52（236.49-941.82） | -2.73   (-3.06--2.41) |
| Bangladesh | 70568.79（45435.04-102186.69） | 389755.56（189434.87-629324.09） | 269.25（134.18-432.51） | 2.73   (2.55-2.91) | 99.93（37.06-210.92） | 884.46（394.12-1678.19） | 0.63（0.28-1.17） | 3.93   (3.69-4.18) | 18.81（-21.50-72.39） | 1386.44（461.90-2903.37） | 1.06（0.33-2.31） | 11.93   (11.43-12.42) | -2955.36（-12492.66-10947.94） | 64584.32（4439.11-149322.04） | 43.17（3.13-99.59） | NA | 59.56（16.54-177.07） | 639.71（176.81-1589.73） | 0.49（0.14-1.19） | 4.39   (4.18-4.59) | 35524.56（20846.10-54108.32） | 130349.06（75491.13-257674.63） | 96.18（53.84-183.83） | 1.17   (0.98-1.36) | 37821.29（11736.22-61939.18） | 191911.56（67379.50-332560.06） | 127.73（44.84-221.22） | 2.72   (2.48-2.97) |
| Barbados | 1647.99（891.80-2574.47） | 2844.35（1548.24-4471.51） | 568.40（307.11-895.65） | -0.15   (-0.29--0.01) | 10.40（5.52-17.51） | 21.64（10.60-38.71） | 4.15（2.03-7.38） | -0.1   (-0.41-0.22) | 21.17（7.80-37.51） | 83.29（33.57-150.60） | 15.98（6.45-28.97） | 2.54   (2.36-2.73) | 319.95（21.29-682.20） | 591.60（33.79-1243.56） | 122.49（6.29-256.69） | -0.11   (-0.24-0.02) | 41.63（13.35-90.30） | 112.92（34.58-236.43） | 21.69（6.63-45.40） | 1.26   (1.03-1.48) | 510.15（369.39-649.85） | 990.53（712.07-1327.26） | 196.58（141.24-263.64） | 0.82   (0.66-0.97) | 744.70（289.43-1233.13） | 1044.37（423.15-1751.29） | 207.52（84.27-347.65） | -1.17   (-1.43--0.91) |
| Belarus | 136321.32（50509.52-231903.56） | 218598.22（79379.95-378432.57） | 1386.09（504.02-2403.44） | 0.2   (-0.3-0.71) | 753.96（394.58-1329.22） | 1791.76（889.05-3218.07） | 11.61（5.77-20.83） | 1.8   (1.34-2.26) | 991.22（402.21-1814.29） | 2408.63（1008.07-4179.86） | 14.62（6.11-25.45） | 1.86   (1.73-2) | 26211.78（2728.17-54383.89） | 32217.19（3154.37-66205.37） | 206.48（19.36-425.94） | -0.77   (-1.3--0.23) | 755.94（168.52-2251.89） | 2298.19（509.52-6427.12） | 13.74（3.03-38.52） | 2.52   (2.23-2.81) | 8400.05（6171.54-10906.63） | 2557.84（1968.51-3288.16） | 16.97（13.09-21.78） | -5.4   (-6.54--4.24) | 99208.38（38361.93-163496.77） | 177324.62（68527.31-295067.32） | 1122.65（434.76-1868.63） | 0.58   (0.09-1.08) |
| Belgium | 50869.33（19667.45-83873.58） | 37600.87（16065.61-62735.02） | 163.59（68.30-272.31） | -2.5   (-2.64--2.36) | 892.11（466.59-1506.17） | 829.69（436.81-1435.30） | 3.63（1.89-6.26） | -1.86   (-2.08--1.64) | 954.68（390.16-1682.30） | 2683.72（1080.45-4624.41） | 10.03（4.05-17.44） | 1.72   (1.5-1.94) | 8094.06（773.02-16784.00） | 6788.34（502.50-14189.61） | 31.40（2.00-65.57） | -1.87   (-1.94--1.79) | 522.70（122.71-1326.97） | 1025.87（261.92-2434.73） | 3.86（0.99-9.01） | 0.43   (0.05-0.81) | 2740.82（1602.55-3876.28） | 4377.31（2078.47-6473.64） | 15.90（9.08-21.91） | -0.43   (-0.9-0.05) | 37664.96（14191.76-60318.15） | 21895.94（8345.62-35997.26） | 98.77（38.45-160.67） | -3.22   (-3.37--3.07) |
| Belize | 699.70（379.55-1068.24） | 2445.94（1554.49-3412.28） | 769.78（495.34-1079.17） | -0.03   (-0.31-0.26) | 1.46（0.74-2.55） | 5.48（2.81-9.18） | 1.83（0.93-3.08） | -0.14   (-0.84-0.57) | 8.26（3.02-15.87） | 47.07（20.76-80.86） | 17.36（7.57-29.90） | 2.01   (1.66-2.38) | 110.43（2.46-236.32） | 392.63（6.82-778.75） | 115.77（2.96-232.04） | -0.28   (-0.75-0.19) | 1.02（0.25-2.70） | 6.22（1.69-14.46） | 2.24（0.62-5.27） | 2.12   (1.64-2.59) | 215.77（173.62-259.71） | 1105.54（915.98-1326.06） | 356.34（277.77-439.27） | 1.92   (1.67-2.17) | 362.75（148.40-589.56） | 889.01（387.42-1401.17） | 276.24（119.01-439.17） | -1.61   (-1.94--1.28) |
| Benin | 9529.99（5453.87-14387.10） | 32851.14（18387.46-51629.63） | 568.97（325.36-871.80） | 0.7   (0.6-0.8) | 26.15（11.02-51.67） | 107.53（36.67-230.69） | 1.93（0.67-4.15） | 1.13   (0.94-1.32) | 31.13（12.50-55.54） | 167.06（64.97-294.70） | 3.66（1.42-6.50） | 2.6   (2.48-2.72) | 2570.16（147.72-5682.81） | 8546.14（392.45-19027.29） | 143.17（7.17-313.31） | 0.48   (0.34-0.62) | 11.34（2.28-34.69） | 72.78（15.14-211.56） | 1.58（0.34-4.46） | 3.11   (2.99-3.23) | 4717.43（2818.34-6552.75） | 14811.74（8804.36-21058.13） | 258.90（152.65-365.51） | 0.44   (0.32-0.56) | 2173.78（743.61-3542.87） | 9145.88（3428.46-15999.46） | 159.73（58.94-280.33） | 1.38   (1.17-1.59) |
| Bermuda | 566.09（247.60-927.63） | 616.23（319.84-981.91） | 469.54（241.40-747.00） | -2.07   (-2.44--1.7) | 10.59（5.70-18.66） | 11.36（5.56-19.64） | 8.23（4.06-14.23） | -2.5   (-2.66--2.34) | 5.72（2.13-11.30） | 23.58（9.61-43.09） | 16.02（6.63-29.42） | 1.45   (1.4-1.5) | 60.46（3.95-131.20） | 77.43（6.15-155.77） | 62.47（4.26-126.14） | -1.52   (-1.67--1.36) | 7.70（2.11-18.11） | 20.77（6.33-42.08） | 13.87（4.18-27.97） | 0.29   (0.05-0.54) | 64.82（49.61-80.13） | 143.09（96.78-188.68） | 104.76（75.09-134.87） | 0.4   (-0.16-0.95) | 416.81（162.90-685.68） | 340.00（141.65-571.28） | 264.18（111.34-442.70） | -2.98   (-3.35--2.6) |
| Bhutan | 1225.87（613.17-2094.14） | 3043.71（1541.01-5105.40） | 476.22（245.09-789.88） | 0.27   (0.24-0.29) | 1.66（0.71-3.46） | 10.24（4.78-19.41） | 1.64（0.77-3.08） | 3.18   (3.11-3.26) | 2.55（0.90-4.97） | 19.14（6.96-34.89） | 3.25（1.17-5.82） | 4.01   (3.94-4.08) | 165.21（10.41-412.16） | 435.40（25.64-1032.35） | 67.08（4.18-159.81） | 0.46   (0.41-0.51) | 0.81（0.19-2.36） | 3.98（1.06-11.10） | 0.69（0.19-1.92） | 2.04   (1.87-2.22) | 418.54（203.41-631.92） | 838.44（538.48-1221.29） | 139.12（88.89-204.39） | -0.66   (-0.7--0.62) | 637.10（220.80-1128.41） | 1736.50（676.45-3090.66） | 264.44（102.48-469.57） | 0.77   (0.74-0.81) |
| Bolivia (Plurinational State of) | 19042.47（9259.23-30655.35） | 46089.78（22451.85-76121.69） | 490.10（246.08-798.43） | -0.44   (-0.58--0.29) | 40.61（17.36-78.76） | 193.48（93.98-356.86） | 2.04（0.99-3.73） | 1.8   (1.74-1.85) | 105.98（37.45-206.83） | 859.89（346.36-1512.12） | 10.25（4.13-18.21） | 3.64   (3.5-3.77) | 3909.70（192.02-9173.89） | 10089.28（249.07-22229.71） | 100.20（2.93-221.45） | -0.34   (-0.46--0.23) | 12.01（2.96-32.47） | 76.72（17.87-182.10） | 0.89（0.20-2.09） | 2.66   (2.6-2.71) | 5183.75（2280.31-7817.94） | 12771.55（7209.60-19099.66） | 144.71（79.27-216.13） | -0.36   (-0.4--0.32) | 9790.41（3651.21-17459.48） | 22098.87（8189.71-39165.43） | 232.00（84.87-410.18） | -0.65   (-0.88--0.41) |
| Bosnia and Herzegovina | 35549.04（15164.91-58495.45） | 42934.08（19654.14-69509.47） | 700.44（316.78-1132.84） | -0.97   (-1.14--0.81) | 173.18（83.88-325.14） | 423.75（186.28-792.70） | 6.95（3.03-12.91） | 1.86   (1.7-2.03) | 243.38（92.87-440.55） | 788.46（332.85-1357.76） | 12.25（5.16-21.05） | 1.9   (1.77-2.03) | 8080.73（757.52-16866.90） | 9884.84（1178.51-20321.18） | 162.43（18.70-330.33） | -0.96   (-1.11--0.81) | 50.90（12.51-131.97） | 161.47（45.33-356.76） | 2.49（0.69-5.52） | 1.97   (1.89-2.05) | 5342.00（3848.68-7327.49） | 8367.61（5117.15-12214.52） | 132.67（81.87-192.99） | 0.09   (-0.09-0.27) | 21658.86（8551.05-35840.87） | 23307.94（8844.63-38759.85） | 383.66（146.36-636.40） | -1.4   (-1.62--1.17) |
| Botswana | 4229.36（2681.49-6390.17） | 12935.15（7833.01-18627.80） | 882.17（548.41-1249.18） | 0.8   (0.49-1.12) | 11.85（5.35-22.86） | 52.32（24.18-96.16） | 3.52（1.65-6.47） | 1.54   (1.38-1.71) | 15.42（5.75-29.54） | 111.12（44.86-188.62） | 9.87（3.93-16.74） | 4.09   (3.84-4.33) | 605.74（45.84-1459.76） | 2260.76（95.38-4677.34） | 146.73（7.52-306.00） | 1.5   (1.09-1.91) | 8.19（1.88-24.59） | 69.77（16.49-180.75） | 5.12（1.23-13.31） | 4.02   (3.86-4.18) | 2780.15（1821.36-4071.82） | 7020.32（4903.25-10010.15） | 497.27（340.74-698.25） | 0.29   (-0.02-0.6) | 808.00（276.58-1434.00） | 3420.85（1257.49-5736.97） | 219.65（80.90-368.22） | 1.62   (1.32-1.92) |
| Brazil | 729389.45（377886.64-1148959.54） | 1367939.86（706284.12-2126841.75） | 538.31（279.48-834.68） | -1.18   (-1.24--1.13) | 5465.45（3017.07-9216.81） | 23158.31（12255.20-40158.74） | 9.09（4.80-15.78） | 1.14   (0.87-1.42) | 5024.09（1930.32-9137.60） | 35706.41（14338.90-63076.16） | 14.60（5.86-25.86） | 2.8   (2.64-2.95) | 154292.31（8945.94-340189.22） | 249336.88（10948.65-526427.35） | 97.63（4.40-205.68） | -1.73   (-1.82--1.65) | 3082.48（859.37-7911.59） | 15625.67（4477.94-33343.82） | 6.29（1.80-13.45） | 1.41   (1.22-1.6) | 213068.17（175781.03-252164.66） | 355514.68（277792.23-428607.16） | 142.20（109.04-173.05） | -1.54   (-1.68--1.41) | 348456.94（138020.48-565136.15） | 688597.92（276303.45-1103320.42） | 268.50（107.45-430.29） | -0.97   (-1.03--0.92) |
| Brunei Darussalam | 536.09（269.68-820.48） | 1830.19（815.57-2818.42） | 414.77（194.61-631.05） | 0.35   (0.19-0.51) | 4.88（2.33-8.49） | 23.92（12.71-42.61） | 5.81（3.10-10.12） | 1.45   (1.3-1.6) | 1.86（0.67-3.62） | 25.47（10.21-42.54） | 6.22（2.48-10.46） | 4.75   (4.44-5.05) | 88.66（6.12-209.15） | 387.06（9.43-761.85） | 82.78（2.81-163.93） | 0.84   (0.74-0.94) | 0.95（0.29-2.41） | 5.22（1.35-11.95） | 1.64（0.45-3.79） | 1.55   (1.47-1.64) | 135.12（92.91-178.19） | 347.63（264.09-471.54） | 94.64（65.00-131.26） | -0.35   (-0.54--0.16) | 304.62（111.24-485.18） | 1040.90（419.09-1642.08） | 223.68（88.63-356.27） | 0.41   (0.22-0.61) |
| Bulgaria | 200368.61（98255.11-319959.89） | 281739.73（187185.66-403370.74） | 2101.91（1386.33-3006.46） | 0.51   (0.24-0.79) | 564.05（293.55-1007.84） | 937.55（478.86-1649.53） | 7.34（3.76-12.95） | 1.2   (0.89-1.52) | 1395.85（561.72-2391.63） | 2728.73（1142.01-4882.30） | 18.25（7.66-32.61） | 1.07   (0.99-1.16) | 43001.73（2786.15-91590.96） | 40005.29（4372.73-81773.07） | 299.68（30.51-606.76） | -0.96   (-1.12--0.8) | 369.42（99.93-825.31） | 673.27（190.15-1500.70） | 4.61（1.30-10.24） | 0.56   (0.09-1.03) | 49421.91（39525.89-60557.78） | 149847.56（105423.26-194683.90） | 1083.92（779.93-1396.41） | 3.84   (3.11-4.57) | 105615.65（41096.10-170486.85） | 87547.34（35170.70-145736.30） | 688.11（279.04-1134.15） | -1.81   (-2.13--1.49) |
| Burkina Faso | 11619.85（7894.66-16391.32） | 36812.53（22916.57-53783.73） | 360.17（232.26-527.40） | 1.38   (1.3-1.45) | 32.08（12.92-70.10） | 109.83（41.73-232.83） | 1.16（0.45-2.46） | 1.61   (1.54-1.69) | 5.79（-0.28-16.88） | 49.59（11.23-118.66） | 0.57（0.10-1.44） | 5.27   (5.12-5.42) | 485.60（-290.94-2156.48） | 3046.86（138.97-8205.22） | 27.52（1.67-74.34） | 3.48   (3.37-3.59) | 9.68（2.08-33.06） | 49.31（10.20-164.24） | 0.62（0.13-1.99） | 3   (2.92-3.09) | 8468.13（5071.08-12581.89） | 24697.64（13078.76-38619.97） | 243.06（128.27-382.08） | 1.13   (1.04-1.22) | 2618.57（861.62-4460.48） | 8859.31（2990.80-16224.41） | 87.25（29.47-157.98） | 1.6   (1.53-1.67) |
| Burundi | 7599.48（2742.17-12252.90） | 16408.59（10596.73-23128.18） | 309.74（201.69-444.63） | -0.67   (-0.92--0.43) | 22.50（8.48-48.34） | 39.82（15.87-84.96） | 0.76（0.31-1.67） | -1.15   (-1.5--0.79) | 4.49（0.15-12.62） | 30.28（8.36-63.58） | 0.69（0.18-1.52） | 3.74   (3.51-3.96) | 70.91（-812.94-1408.97） | 1353.82（-142.52-4015.44） | 25.36（-0.16-65.59） | 3.31   (2.9-3.72) | 6.34（1.63-17.94） | 18.64（4.64-55.49） | 0.48（0.12-1.43） | 1.15   (0.8-1.51) | 5751.13（556.37-10005.70） | 10425.18（5171.97-15226.18） | 204.86（100.92-302.88） | -1.06   (-1.28--0.84) | 1744.12（516.50-3101.97） | 4540.85（1531.02-8049.76） | 77.59（25.29-136.93） | -0.26   (-0.54-0.01) |
| Cabo Verde | 862.88（547.86-1215.51） | 2846.43（1601.80-4416.58） | 612.68（346.84-950.12） | 1.1   (0.89-1.32) | 3.77（1.24-8.75） | 16.45（6.31-34.76） | 3.59（1.37-7.49） | 2.13   (1.94-2.32) | 3.49（1.15-6.67） | 27.39（10.87-48.09） | 6.52（2.63-11.29） | 4.67   (4.5-4.85) | 119.60（11.48-266.41） | 555.29（41.94-1154.82） | 115.31（9.18-240.82） | 2.07   (1.89-2.25) | 2.57（0.54-7.66） | 19.05（4.80-52.58） | 4.37（1.12-12.03） | 4.32   (4.11-4.52) | 522.71（344.79-736.18） | 1064.27（772.44-1426.51） | 231.29（166.60-312.19） | -0.32   (-0.56--0.09) | 210.74（76.49-360.55） | 1163.98（453.16-2009.96） | 251.61（97.59-433.86） | 2.57   (2.28-2.87) |
| Cambodia | 13887.74（8215.02-20601.91） | 43282.52（26837.15-62305.51） | 321.97（203.74-463.94） | 0.42   (0.29-0.54) | 8.25（2.95-16.74） | 39.74（17.70-79.12） | 0.32（0.14-0.63） | 1.91   (1.73-2.08) | 10.26（0.79-24.59） | 114.84（34.56-214.64） | 0.91（0.25-1.86） | 5.24   (5.17-5.3) | 446.85（-979.43-2953.12） | 5443.13（130.44-13036.83） | 38.91（1.63-92.32） | 4.36   (4.15-4.56) | 5.43（1.43-15.08） | 28.08（7.12-73.24） | 0.24（0.06-0.62） | 1.78   (1.68-1.89) | 9228.03（4273.47-14475.96） | 23262.09（12537.83-34221.81） | 178.51（95.73-265.98） | -0.22   (-0.35--0.09) | 4188.92（1308.13-7173.84） | 14394.64（4846.73-24833.90） | 103.08（34.27-176.93） | 0.76   (0.64-0.87) |
| Cameroon | 34730.32（20861.50-51288.73） | 144636.23（72983.70-240527.58） | 1035.93（537.77-1709.15） | 1.19   (0.84-1.55) | 117.44（51.14-231.47） | 667.07（288.79-1365.14） | 5.03（2.19-10.31） | 1.94   (1.69-2.19) | 149.65（56.03-269.39） | 972.58（413.32-1778.63） | 10.62（4.45-18.59） | 2.96   (2.9-3.01) | 7449.05（402.24-16261.94） | 37915.74（1022.32-82713.52） | 253.13（9.15-549.11） | 1.81   (1.39-2.22) | 77.43（15.13-240.14） | 736.50（177.54-1836.41） | 6.91（1.70-17.00） | 3.97   (3.84-4.1) | 18907.77（10056.35-25996.90） | 58225.38（28234.46-87725.04） | 422.29（204.91-637.60） | 0.14   (-0.07-0.34) | 8028.98（2774.82-14149.75） | 46118.95（17358.52-85013.17） | 337.96（125.53-619.47） | 2.35   (1.75-2.96) |
| Canada | 163605.53（66048.44-277698.94） | 182776.18（83746.48-288674.62） | 270.25（124.53-426.30） | -2.25   (-2.42--2.08) | 3341.95（1724.16-5894.26） | 2687.37（1416.34-4699.15） | 3.88（2.03-6.75） | -3.84   (-4.17--3.52) | 4292.43（1710.32-8134.35） | 12931.86（5262.90-23456.60） | 16.63（6.86-30.37） | 0.65   (0.54-0.77) | 18301.14（1407.99-39376.31） | 26217.04（2064.52-54126.15） | 41.22（2.92-83.97） | -1.36   (-1.53--1.2) | 1904.49（432.49-5251.14） | 3694.91（964.56-9119.97） | 4.76（1.24-11.64） | -0.98   (-1.2--0.76) | 7123.67（5438.90-8793.91） | 26238.10（19263.57-32745.65） | 40.78（32.40-48.52） | 2.38   (1.99-2.77) | 128641.84（50641.94-213569.35） | 111006.90（44422.09-177610.42） | 162.99（65.80-260.49） | -3.13   (-3.26--2.99) |
| Central African Republic | 7196.39（3632.01-11040.01） | 22554.75（12441.04-35003.07） | 905.03（514.58-1391.91） | 1.37   (1.32-1.42) | 16.07（7.19-31.83） | 46.49（20.04-100.65） | 1.90（0.81-4.02） | 0.89   (0.78-1.01) | 7.12（1.96-16.56） | 55.22（20.19-112.03） | 2.71（0.97-5.76） | 5   (4.93-5.07) | 381.92（-99.60-1288.74） | 2960.07（135.24-7015.58） | 104.48（5.99-238.23） | 4.19   (4.06-4.31) | 9.07（2.02-27.44） | 44.37（10.32-133.89） | 2.20（0.51-6.36） | 3.03   (2.92-3.13) | 5100.42（1616.35-8955.57） | 13092.30（5017.87-22155.85） | 560.91（216.52-923.31） | 0.85   (0.8-0.91) | 1681.80（507.24-3059.05） | 6356.29（1985.43-11873.44） | 232.83（71.45-433.65） | 1.87   (1.8-1.94) |
| Chad | 11360.56（6791.81-16747.14） | 35167.41（19474.01-51928.43） | 553.50（318.15-822.22） | 0.98   (0.76-1.19) | 24.26（8.91-56.03） | 70.29（27.31-155.53） | 1.18（0.44-2.62） | 0.79   (0.67-0.9) | 28.01（8.97-55.20） | 104.36（37.68-194.92） | 2.26（0.75-4.45） | 2.23   (2.18-2.28) | 1542.74（100.10-3633.67） | 5841.12（407.45-13082.04） | 89.51（6.86-199.46） | 1.59   (1.45-1.72) | 18.20（3.67-58.41） | 67.16（14.70-216.82） | 1.39（0.29-4.55） | 2.06   (2.02-2.11) | 7114.70（3224.96-10323.23） | 19833.59（9756.87-31364.99） | 312.25（151.22-476.80） | 0.64   (0.4-0.88) | 2632.63（779.15-4732.64） | 9250.88（2913.27-15944.54） | 146.92（45.28-251.10） | 1.42   (1.2-1.63) |
| Chile | 58345.98（27254.95-96746.55） | 97263.96（53305.23-148619.74） | 384.71（208.98-588.18） | -1.07   (-1.16--0.99) | 550.92（296.22-964.78） | 1460.06（751.76-2582.35） | 5.73（2.96-10.12） | 0   (-0.36-0.36) | 663.80（256.49-1234.54） | 3255.39（1341.77-5784.84） | 12.42（5.12-22.08） | 2.32   (1.97-2.67) | 14546.54（727.14-32034.67） | 19815.69（957.00-39874.78） | 80.20（3.62-161.60） | -1.57   (-1.67--1.46) | 357.94（87.30-884.41） | 1008.04（294.22-2256.39） | 3.83（1.12-8.59） | 0.2   (-0.47-0.87) | 12637.50（9536.20-15690.67） | 29270.12（18811.45-37706.92） | 113.04（73.79-144.73） | -0.08   (-0.3-0.15) | 29589.28（11824.30-48187.34） | 42454.66（17333.16-66408.83） | 169.49（69.58-265.10） | -1.63   (-1.71--1.54) |
| China | 2253093.12（1616542.24-3195666.99） | 8000896.35（3901647.02-13000730.61） | 396.09（197.27-638.54） | 1.3   (1.13-1.47) | 2183.24（1073.80-3984.95） | 14984.83（7677.08-27247.13） | 0.74（0.38-1.35） | 4.11   (3.92-4.31) | 2095.27（478.16-4445.32） | 75251.71（29279.94-129907.15） | 3.84（1.51-6.57） | 8.58   (8.42-8.75) | 153660.47（-58315.98-659099.00） | 2006701.14（168750.22-4379523.17） | 95.25（7.96-207.77） | 5.34   (4.9-5.77) | 3425.85（925.93-9199.58） | 24742.04（5762.55-63490.28） | 1.17（0.27-3.00） | 2.93   (2.88-2.97) | 1393487.86（812664.35-1929010.26） | 2373039.87（1485461.40-3462005.03） | 120.03（71.90-180.13） | -1.46   (-1.89--1.03) | 698240.43（239691.73-1169252.46） | 3506176.76（1295665.94-5987176.68） | 175.05（64.48-298.21） | 2.97   (2.76-3.19) |
| Colombia | 115760.86（68450.56-168734.47） | 215257.11（105260.73-344735.56） | 387.00（189.31-619.03） | -1.98   (-2.2--1.76) | 852.16（451.27-1468.57） | 3431.89（1735.02-5719.15） | 6.24（3.16-10.41） | -0.09   (-0.46-0.28) | 795.26（314.23-1386.65） | 6576.36（2605.15-11805.56） | 11.76（4.67-21.09） | 2.61   (2.53-2.69) | 13820.27（1093.35-30464.07） | 29538.30（1363.60-63721.48） | 53.49（2.46-115.14） | -1.43   (-1.63--1.23) | 248.93（68.79-666.57） | 1070.04（278.07-2553.76） | 1.91（0.50-4.55） | 0.34   (0.01-0.67) | 41200.44（32172.61-50434.97） | 40958.75（27362.69-55516.45） | 73.42（49.29-99.00） | -4.34   (-4.6--4.08) | 58843.80（22493.87-95020.12） | 133681.76（55143.25-219617.98） | 240.19（98.95-394.64） | -1.1   (-1.3--0.91) |
| Comoros | 1039.66（609.64-1546.32） | 3200.87（1854.42-4822.97） | 629.06（381.46-938.70） | 0.43   (0.22-0.65) | 3.10（1.00-6.62） | 12.26（4.15-26.86） | 2.38（0.81-5.23） | 1.15   (0.9-1.4) | 1.27（0.35-2.78） | 15.79（6.12-28.78） | 3.59（1.36-6.63） | 5.06   (5-5.12) | 74.27（-5.91-227.19） | 514.17（29.83-1109.67） | 94.31（6.03-203.57） | 2.86   (2.67-3.05) | 0.61（0.13-1.74） | 4.28（0.75-11.38） | 1.00（0.18-2.65） | 2.99   (2.85-3.13) | 775.59（360.69-1259.93） | 1802.70（1043.43-2744.34） | 369.91（208.35-574.37） | -0.39   (-0.6--0.17) | 184.81（59.77-305.20） | 851.67（320.60-1473.64） | 157.87（59.31-275.22） | 1.77   (1.59-1.95) |
| Congo | 10940.45（6731.44-16198.90） | 35900.93（22000.97-54316.91） | 1213.72（767.05-1813.19） | 0.42   (0.24-0.6) | 36.56（18.69-67.53） | 137.82（64.20-258.27） | 4.62（2.19-8.48） | 0.77   (0.57-0.96) | 17.21（5.89-34.10） | 168.79（70.43-297.66） | 7.33（2.81-13.57） | 4.98   (4.77-5.18) | 894.75（42.17-2296.87） | 5232.69（206.29-11181.85） | 157.75（7.33-336.67） | 1.92   (1.55-2.3) | 15.67（4.04-45.73） | 104.51（26.83-284.89） | 4.20（1.13-10.80） | 3.11   (2.98-3.24) | 7133.09（3332.19-11160.57） | 18744.81（10494.66-28548.29） | 676.86（383.18-1007.27） | -0.12   (-0.27-0.04) | 2843.17（918.07-4980.61） | 11512.31（4132.96-20138.22） | 362.95（130.28-623.96） | 0.96   (0.76-1.16) |
| Cook Islands | 360.21（250.08-487.81） | 424.42（278.39-577.85） | 1741.51（1123.18-2392.00） | -1.26   (-1.41--1.1) | 0.93（0.47-1.69） | 2.04（0.79-4.21） | 8.38（3.28-17.23） | 0.61   (0.55-0.67) | 2.09（0.86-3.69） | 6.02（2.52-10.58） | 23.84（10.06-41.88） | 1.12   (1.08-1.16) | 46.33（-0.01-94.76） | 49.69（0.85-99.02） | 213.62（3.02-423.96） | -1.2   (-1.44--0.96) | 0.34（0.11-0.72） | 1.30（0.46-2.49） | 5.02（1.75-9.57） | 1.9   (1.74-2.07) | 199.45（150.65-251.84） | 213.50（160.80-276.00） | 858.60（639.22-1119.65） | -1.79   (-1.91--1.66) | 111.07（46.69-175.00） | 151.87（67.54-241.31） | 632.05（282.12-1000.82） | -0.53   (-0.71--0.34) |
| Costa Rica | 8354.41（4386.71-13206.67） | 20702.16（11305.41-32505.06） | 373.34（203.46-585.54） | -1.17   (-1.33--1.01) | 65.39（34.36-114.01） | 303.31（154.17-521.39） | 5.50（2.79-9.42） | 0.64   (0.37-0.91) | 114.33（45.31-219.49） | 737.71（291.92-1308.34） | 13.23（5.25-23.50） | 1.96   (1.84-2.08) | 1000.70（57.34-2206.67） | 2728.49（124.23-5584.06） | 49.64（2.25-101.58） | -0.67   (-0.96--0.38) | 31.65（8.60-79.16） | 121.05（33.44-280.00） | 2.17（0.60-5.02） | -0.17   (-0.45-0.11) | 1932.58（1470.22-2405.83） | 5078.33（3446.33-6601.00） | 91.11（62.22-118.03） | -1.66   (-2.03--1.3) | 5209.75（2045.16-8610.55） | 11733.28（4773.11-19335.24） | 211.69（86.10-348.94） | -1.22   (-1.42--1.02) |
| Coted'Ivoire | 24537.58（14227.25-36647.22） | 104395.67（57392.13-163975.96） | 798.76（455.74-1232.11） | 1.32   (1.09-1.56) | 94.13（36.66-188.12） | 437.52（159.98-976.34） | 3.50（1.29-7.80） | 1.17   (1-1.34) | 62.38（24.12-110.05） | 432.45（174.12-742.06） | 5.20（2.19-9.01） | 2.87   (2.78-2.95) | 4733.56（390.63-10892.11） | 22354.85（1145.37-48438.04） | 157.89（9.25-335.68） | 1.72   (1.52-1.92) | 35.48（7.26-113.94） | 270.13（59.14-806.15） | 2.79（0.62-8.28） | 2.74   (2.61-2.88) | 11948.35（6871.79-17030.61） | 46069.75（25947.98-66259.12） | 360.60（204.47-520.59） | 1.03   (0.78-1.27) | 7663.67（2617.36-12644.79） | 34830.97（12601.58-61449.28） | 268.77（94.46-461.90） | 1.49   (1.25-1.74) |
| Croatia | 66303.57（32086.48-106388.07） | 54691.23（27317.22-87370.61） | 621.80（310.84-993.84） | -1.88   (-2--1.76) | 379.30（198.73-657.94） | 729.13（367.59-1258.23） | 8.49（4.30-14.62） | 0.84   (0.55-1.14) | 350.88（138.74-634.27） | 1068.08（453.51-1867.42） | 11.00（4.62-19.29） | 2.21   (1.97-2.45) | 13114.38（961.29-27826.07） | 8681.27（825.95-18568.48） | 100.20（8.56-211.32） | -2.85   (-3.03--2.66) | 602.24（169.78-1472.94） | 1349.49（391.82-2903.00） | 14.15（4.06-30.23） | 0.68   (0.45-0.92) | 13129.50（8981.43-17450.00） | 11106.47（6715.84-15327.83） | 121.21（77.57-162.83） | -1.35   (-1.78--0.91) | 38727.27（15128.95-62902.50） | 31756.80（12660.62-53234.74） | 366.75（147.15-607.59） | -1.95   (-2.03--1.86) |
| Cuba | 52424.61（22292.38-81987.48） | 110040.27（60350.43-171036.53） | 577.64（310.95-897.02） | 0.35   (0.15-0.55) | 485.93（267.11-780.61） | 978.60（509.74-1704.18） | 5.06（2.64-8.75） | -0.14   (-0.38-0.09) | 446.54（175.46-750.28） | 2248.96（901.73-3778.79） | 10.94（4.43-18.41） | 2.98   (2.89-3.06) | 7266.64（460.10-15118.44） | 15101.63（988.16-31354.94） | 82.78（5.04-170.44） | 0.42   (0.31-0.54) | 342.55（97.01-882.93） | 1931.41（574.82-4471.12） | 9.53（2.85-21.95） | 3.86   (3.65-4.07) | 6408.72（5266.17-7661.78） | 32206.55（23774.52-40642.06） | 165.76（125.45-207.12） | 3.65   (3.47-3.84) | 37474.25（14618.78-58774.93） | 57573.11（22433.27-95922.80） | 303.57（119.26-502.25） | -0.83   (-1.08--0.57) |
| Cyprus | 3837.61（1918.27-5856.28） | 6237.46（3237.27-10132.25） | 343.57（187.54-548.00） | -1.87   (-2.01--1.73) | 57.32（28.30-99.27） | 125.92（62.71-221.48） | 6.09（3.09-10.65） | -1.03   (-1.3--0.76) | 50.90（20.63-91.85） | 209.51（84.23-384.05） | 11.66（4.78-21.20） | 1.21   (1.08-1.34) | 431.15（37.57-904.31） | 676.75（51.96-1483.58） | 36.65（2.87-80.76） | -2.09   (-2.26--1.92) | 21.59（6.20-57.26） | 72.19（20.17-176.28） | 4.05（1.14-10.36） | 0.44   (0.3-0.58) | 861.71（404.76-1410.38） | 1461.21（836.83-2148.36） | 89.31（42.91-135.43） | -1.76   (-2.02--1.5) | 2414.93（923.49-3862.75） | 3691.89（1502.22-6387.65） | 195.80（79.26-337.01） | -2.05   (-2.22--1.88) |
| Czechia | 186493.03（66568.51-313196.19） | 123795.02（61741.93-201389.11） | 582.06（287.89-941.70） | -2.76   (-2.85--2.66) | 1046.21（551.38-1788.66） | 1475.65（745.08-2551.63） | 7.16（3.63-12.41） | -0.24   (-0.54-0.07) | 1727.95（695.13-3109.50） | 4585.01（1864.99-8302.55） | 19.72（8.02-35.59） | 1.72   (1.51-1.94) | 38700.14（3907.91-79852.09） | 15250.22（1608.55-32299.34） | 74.44（7.29-155.63） | -4.45   (-4.61--4.28) | 1432.73（385.90-3168.19） | 2537.03（758.22-5467.06） | 11.39（3.41-24.49） | -0.4   (-1.12-0.32) | 6706.26（5372.60-8002.08） | 21180.39（13511.08-28223.00） | 95.73（64.02-125.74） | 1.93   (1.16-2.71) | 136879.73（53898.89-219194.06） | 78766.72（31102.82-131064.11） | 373.62（148.41-619.35） | -3.12   (-3.25--3) |
| Democratic People's Republic of Korea | 27537.51（15999.04-44259.21） | 120840.94（70768.73-205440.42） | 392.99（229.88-667.25） | 2.41   (2.37-2.46) | 65.30（29.63-124.38） | 173.21（84.83-317.16） | 0.53（0.26-0.96） | 1.22   (1.11-1.32) | 21.49（-2.42-70.57） | 1090.13（349.51-2339.87） | 4.20（1.22-9.43） | 10   (9.83-10.17) | -1373.86（-4996.69-4868.28） | 14690.95（597.62-47740.24） | 48.43（3.06-148.28） | #VALUE! | 54.24（13.79-160.19） | 291.08（67.20-813.97） | 0.96（0.22-2.66） | 2.54   (2.47-2.61) | 16712.42（9433.06-26523.52） | 53922.08（34411.04-81005.93） | 177.24（109.72-267.15） | 1.46   (1.37-1.56) | 12057.91（3612.51-22356.60） | 50673.49（16211.46-94578.87） | 161.64（52.38-298.39） | 2.49   (2.44-2.54) |
| Democratic Republic of the Congo | 77921.05（45526.27-115820.84） | 327465.62（186051.64-481420.12） | 889.29（520.67-1318.23） | 1.93   (1.9-1.96) | 242.93（105.69-482.64） | 872.22（355.15-1845.56） | 2.35（0.99-4.92） | 1.08   (0.71-1.45) | 124.31（35.65-255.25） | 1510.18（530.39-2879.01） | 5.27（1.87-10.04） | 5.81   (5.65-5.96) | 4695.28（-180.08-14133.45） | 42158.76（2017.54-97329.60） | 104.16（5.55-239.06） | 4.1   (4.06-4.15) | 230.28（51.43-701.10） | 1381.18（262.83-3957.42） | 4.42（0.83-12.77） | 2.95   (2.49-3.42) | 53144.77（23562.48-86057.22） | 193107.45（106134.57-304850.76） | 551.64（301.35-855.44） | 1.62   (1.55-1.69) | 19483.50（5994.22-34474.67） | 88435.82（30053.26-153507.52） | 221.44（73.44-390.99） | 1.96   (1.8-2.11) |
| Denmark | 39439.39（15174.82-64951.71） | 19086.87（8036.95-32806.16） | 167.61（70.94-285.17） | -3.84   (-3.97--3.71) | 823.19（438.82-1375.09） | 813.43（422.18-1412.16） | 6.71（3.56-11.44） | -2.06   (-2.42--1.7) | 589.19（239.06-983.93） | 1564.10（594.27-2782.56） | 12.25（4.70-21.71） | 1.7   (1.38-2.02) | 4563.11（408.91-9364.35） | 3460.07（265.94-7415.66） | 31.66（2.09-67.16） | -2.28   (-2.48--2.07) | 497.44（104.21-1423.71） | 698.02（166.75-1745.46） | 5.30（1.26-13.17） | -0.74   (-1.09--0.4) | 1654.49（1109.93-2230.27） | 2077.92（1148.80-2953.25） | 16.45（10.14-22.48） | -0.65   (-0.9--0.4) | 31311.97（12177.10-50705.81） | 10473.32（4108.32-17436.68） | 95.23（37.77-156.80） | -5   (-5.21--4.8) |
| Djibouti | 462.59（309.36-686.71） | 2533.00（1554.01-3790.10） | 357.40（228.75-528.04） | 0.27   (0.19-0.34) | 1.34（0.58-2.51） | 8.83（3.52-17.78） | 1.24（0.50-2.46） | 0.84   (0.73-0.95) | 0.17（-0.01-0.56） | 4.64（1.12-10.01） | 0.78（0.17-1.72） | 6.17   (6.04-6.3) | -5.52（-39.61-46.10） | 210.79（3.62-555.08） | 26.89（1.31-67.14） | NA | 0.36（0.10-0.96） | 4.77（1.21-12.58） | 0.93（0.24-2.43） | 3.05   (2.95-3.16) | 393.58（236.48-597.03） | 1577.77（979.62-2469.58） | 237.15（141.92-378.77） | -0.6   (-0.68--0.51) | 72.64（21.80-137.64） | 726.21（222.30-1288.83） | 90.42（27.29-158.08） | 2.36   (2.22-2.5) |
| Dominica | 698.49（453.90-991.80） | 989.84（629.38-1399.03） | 1191.23（770.10-1688.15） | 0.02   (-0.18-0.22) | 3.52（1.65-6.60） | 7.19（3.58-12.17） | 8.57（4.30-14.47） | 0.92   (0.74-1.1) | 8.37（3.35-14.85） | 19.34（8.44-33.61） | 24.69（10.88-42.85） | 1.65   (1.57-1.73) | 94.77（4.65-198.20） | 150.69（7.33-303.76） | 179.69（9.01-364.23） | 0.25   (0.11-0.4) | 2.06（0.56-5.22） | 6.13（1.80-13.13） | 7.61（2.21-16.32） | 2.4   (2.32-2.48) | 351.26（255.66-440.76） | 503.37（363.84-671.37） | 611.43（437.42-811.42） | 0.24   (0.04-0.43) | 238.50（94.46-380.78） | 303.13（126.60-485.70） | 359.24（149.45-575.31） | -0.58   (-0.85--0.31) |
| Dominican Republic | 21236.58（11767.21-32863.36） | 85670.35（41964.81-137959.94） | 825.19（407.58-1329.56） | 1.99   (1.84-2.14) | 50.41（27.27-90.04） | 267.11（131.59-477.70） | 2.63（1.29-4.71） | 2.24   (2.08-2.4) | 83.82（28.97-158.83） | 794.52（300.57-1424.85） | 8.04（3.04-14.45） | 4.15   (3.96-4.34) | 2754.04（178.99-6700.42） | 13083.42（418.54-28413.29） | 123.82（4.23-269.67） | 2.47   (2.31-2.63) | 21.69（5.39-61.44） | 167.87（41.32-379.78） | 1.71（0.42-3.87） | 3.56   (3.4-3.72) | 7025.09（5367.34-9206.65） | 21128.69（14641.32-29318.44） | 207.60（142.35-288.03） | 0.92   (0.75-1.1) | 11301.53（4105.38-18606.28） | 50228.75（19185.96-83612.01） | 481.38（183.80-805.34） | 2.41   (2.25-2.57) |
| Ecuador | 30159.98（17207.26-46546.61） | 81985.44（40055.50-128884.21） | 501.62（250.25-791.42） | 0.29   (-0.04-0.62) | 85.22（43.33-150.11） | 430.55（212.49-780.55） | 2.59（1.28-4.70） | 2.3   (1.88-2.72) | 244.69（88.50-471.00） | 2078.09（846.54-3642.15） | 13.27（5.36-23.26） | 3.4   (3.28-3.53) | 5912.35（334.71-12953.03） | 14849.37（328.75-31589.82） | 87.51（2.13-187.13） | -0.3   (-0.53--0.06) | 33.27（7.25-86.67） | 271.84（74.68-566.64） | 1.72（0.47-3.61） | 3.12   (2.87-3.37) | 11226.79（8808.19-13944.43） | 20046.28（13454.73-28553.09） | 128.42（82.05-185.69） | -0.12   (-1.01-0.78) | 12657.66（5138.74-20759.86） | 44309.31（17712.58-72007.58） | 268.11（106.52-438.04） | 0.56   (0.07-1.05) |
| Egypt | 705187.47（408586.87-1050874.68） | 1922952.26（994790.87-2921556.55） | 2961.49（1586.30-4447.25） | 0.8   (0.68-0.92) | 182.91（84.53-338.85） | 1012.48（516.17-1735.58） | 1.41（0.71-2.47） | 2.93   (2.85-3) | 1437.57（589.09-2550.53） | 6842.45（3074.27-11425.06） | 16.07（7.06-26.75） | 2.25   (2.18-2.32) | 106623.70（8711.21-228595.41） | 295923.00（34890.27-565341.06） | 443.21（55.22-863.13） | 0.85   (0.73-0.97) | 198.16（39.26-529.61） | 961.73（267.14-2135.78） | 1.79（0.50-3.91） | 2.04   (1.92-2.16) | 243839.87（179802.33-332337.70） | 462209.21（351183.19-602730.54） | 806.63（564.79-1086.13） | -0.43   (-0.57--0.29) | 352905.26（143186.23-562464.04） | 1156003.39（507414.10-1824347.34） | 1692.38（723.55-2692.59） | 1.52   (1.33-1.71) |
| El Salvador | 17032.89（7071.46-28149.34） | 33524.78（14534.24-54939.32） | 538.06（232.17-881.20） | -0.12   (-0.4-0.15) | 28.58（15.15-49.34） | 82.37（40.38-145.11） | 1.35（0.66-2.37） | 1.03   (0.86-1.2) | 237.37（87.67-434.87） | 1095.68（473.30-1978.12） | 16.58（7.20-29.86） | 2.17   (2.1-2.25) | 3106.45（111.92-6938.04） | 4733.66（135.09-9922.48） | 76.80（2.10-161.16） | -0.89   (-1.27--0.51) | 38.34（9.11-99.80） | 129.19（34.55-307.16） | 1.97（0.52-4.65） | 1.21   (1.11-1.32) | 2311.50（1759.37-2940.21） | 3824.77（2574.85-5365.97） | 59.88（40.61-83.52） | -0.88   (-1.13--0.62) | 11310.65（4640.46-18506.55） | 23659.11（9875.14-38923.40） | 381.48（159.92-629.85） | 0.11   (-0.16-0.37) |
| Equatorial Guinea | 2119.59（1283.85-3106.28） | 5778.53（3052.11-9557.80） | 1069.77（590.89-1729.07） | -0.19   (-0.51-0.14) | 4.73（2.15-9.47） | 33.43（12.52-75.52） | 6.01（2.29-13.02） | 3.03   (2.91-3.15) | 3.87（1.35-7.64） | 41.71（15.66-72.58） | 10.60（3.89-18.41） | 5.51   (5.44-5.58) | 222.74（10.11-515.25） | 838.01（31.52-1803.33） | 137.37（6.61-293.76） | 0.48   (0.17-0.79) | 3.14（0.71-9.84） | 45.85（10.99-109.19） | 10.68（2.54-25.23） | 6.43   (6.27-6.59) | 1375.91（702.04-2211.49） | 2644.80（1434.39-4272.23） | 524.48（272.37-864.79） | -1.17   (-1.5--0.84) | 509.21（180.98-879.70） | 2174.72（757.29-3967.42） | 380.64（133.78-683.98） | 1.27   (1-1.55) |
| Eritrea | 4743.98（2495.70-7094.54） | 13126.51（8033.46-19628.55） | 434.18（272.29-632.74） | 0.45   (0.32-0.58) | 7.29（2.75-15.05） | 29.37（10.63-63.79） | 0.96（0.35-2.05） | 1.47   (1.31-1.62) | 3.22（0.95-6.72） | 27.58（9.63-52.84） | 1.13（0.38-2.27） | 4.32   (4.2-4.45) | 72.32（-225.18-641.13） | 1276.82（45.16-3166.42） | 38.41（2.16-91.02） | 5.04   (4.9-5.17) | 1.34（0.33-4.36） | 11.12（2.59-33.86） | 0.49（0.12-1.38） | 3.88   (3.81-3.95) | 4034.64（1620.14-6344.16） | 8760.22（4862.87-13257.30） | 306.46（165.12-452.80） | -0.2   (-0.33--0.07) | 625.18（176.47-1107.04） | 3021.41（919.87-5245.23） | 86.73（26.59-148.77） | 2.27   (2.21-2.33) |
| Estonia | 28437.81（11930.96-45817.79） | 28112.20（18323.18-38135.74） | 999.93（702.10-1356.35） | -1.02   (-1.31--0.74) | 141.82（74.80-249.99） | 213.86（110.46-364.23） | 8.60（4.48-14.61） | 0.15   (-0.23-0.53) | 208.43（84.86-357.37） | 495.35（204.43-898.85） | 16.33（6.87-29.65） | 1.46   (1.4-1.52) | 5141.26（524.76-10311.87） | 1762.49（179.00-3670.58） | 73.55（7.00-148.32） | -5.35   (-5.83--4.85) | 59.87（13.03-161.19） | 149.27（38.20-349.47） | 4.98（1.27-11.51） | 1.6   (1.02-2.19) | 3594.98（2859.58-4285.91） | 18481.22（10965.29-25065.08） | 631.73（413.55-825.35） | 5.88   (4.92-6.85) | 19291.45（7484.18-31432.77） | 7010.00（2688.93-11584.65） | 264.74（103.20-434.62） | -4.69   (-5.1--4.28) |
| Eswatini | 3319.46（2057.44-4750.16） | 10249.21（5723.27-16161.97） | 1781.10（1020.36-2761.50） | 1.79   (1.2-2.38) | 9.55（4.41-17.53） | 32.97（14.73-61.92） | 5.72（2.60-10.59） | 1.7   (1.4-2.01) | 19.11（7.75-32.38） | 75.50（32.67-124.28） | 18.71（8.09-30.59） | 2.54   (2.24-2.84) | 556.35（31.15-1260.47） | 2102.05（50.17-4542.19） | 356.11（12.40-755.61） | 2.45   (1.77-3.14) | 7.14（1.48-21.74） | 43.95（10.08-128.15） | 8.29（1.98-23.51） | 3.67   (3.34-4.01) | 2072.30（1278.16-2758.81） | 5083.70（2629.16-7926.90） | 906.55（483.63-1372.17） | 1.05   (0.52-1.57) | 655.00（259.15-1123.04） | 2911.05（1084.05-5182.13） | 485.73（184.65-861.63） | 3.02   (2.3-3.74) |
| Ethiopia | 92299.18（55382.27-124525.76） | 120413.47（85761.60-162048.30） | 254.58（179.20-345.48） | -2.22   (-2.44--2.01) | 89.93（37.78-182.97） | 332.90（146.50-644.88） | 0.69（0.30-1.33） | 1.37   (1.15-1.59) | 61.55（15.67-133.21） | 388.38（143.10-745.65） | 0.92（0.33-1.76） | 3.82   (3.68-3.96) | 2209.38（-3604.10-13695.74） | 10604.09（307.09-26501.30） | 21.60（0.87-53.49） | 1.63   (1.26-2) | 53.76（13.29-154.10） | 282.57（68.48-729.49） | 0.74（0.19-1.88） | 2.74   (2.5-2.97) | 72523.83（33372.66-105387.99） | 76753.78（52643.87-110255.48） | 167.65（109.45-244.03） | -2.88   (-3.1--2.66) | 17360.74（5396.35-29543.14） | 32051.75（11165.83-55964.55） | 62.98（21.78-110.17） | -0.92   (-1.1--0.75) |
| Fiji | 8471.89（3872.28-13703.91） | 17687.63（8213.77-28612.39） | 2119.31（1004.07-3421.67） | 0.22   (0.12-0.32) | 23.65（11.38-44.90） | 77.81（38.67-133.55） | 9.89（4.90-17.17） | 1.58   (1.44-1.71) | 39.31（15.89-71.77） | 165.21（76.20-272.70） | 26.44（11.98-44.22） | 2.4   (2.22-2.58) | 1299.25（15.73-2933.59） | 2705.95（13.13-5361.50） | 318.75（3.61-645.98） | 0.25   (0.14-0.37) | 8.89（2.62-20.52） | 36.51（12.20-69.74） | 5.32（1.72-10.13） | 2.04   (1.77-2.3) | 1765.62（1351.49-2279.01） | 2967.04（2140.68-4005.66） | 376.47（272.84-503.69） | -0.67   (-0.8--0.55) | 5335.16（2083.47-8916.39） | 11735.12（5073.48-19317.17） | 1382.44（588.66-2288.74） | 0.46   (0.35-0.56) |
| Finland | 48956.68（20356.56-80427.31） | 46793.66（25697.49-72704.25） | 366.53（204.80-573.91） | -2   (-2.11--1.89) | 1093.56（581.44-1889.89） | 833.69（422.53-1482.33） | 6.95（3.57-12.19） | -2.55   (-2.75--2.36) | 991.61（393.62-1748.64） | 1966.61（782.47-3510.60） | 13.77（5.52-25.10） | -0.23   (-0.44--0.02) | 6071.85（567.65-12941.21） | 5050.07（423.26-10782.78） | 44.38（3.04-93.41） | -2.25   (-2.31--2.19) | 606.23（165.38-1366.28） | 873.92（249.32-2102.86） | 5.93（1.73-14.25） | -0.34   (-0.89-0.22) | 3867.55（2660.90-5139.05） | 14387.34（7302.38-20370.10） | 103.06（61.97-137.64） | 3.33   (2.68-3.98) | 36325.88（14100.66-59503.84） | 23682.03（9436.23-39547.03） | 192.43（78.14-319.14） | -3.26   (-3.31--3.21) |
| France | 194639.58（96225.59-302299.00） | 227225.62（115351.54-367010.42） | 156.22（77.79-251.55） | -1.43   (-1.54--1.33) | 3689.22（2005.77-6140.16） | 4290.35（2172.62-7685.99） | 3.19（1.64-5.63） | -1.67   (-1.94--1.4) | 5517.45（2208.69-9521.13） | 17684.26（6785.39-30575.44） | 10.61（4.08-18.58） | 1.68   (1.54-1.82) | 30298.86（2888.13-63682.86） | 36141.83（2916.93-76300.51） | 27.94（1.85-59.28） | -0.91   (-1--0.82) | 1831.73（388.13-4804.92） | 4544.10（1059.44-10752.64） | 2.80（0.65-6.58） | 0.92   (0.51-1.33) | 36886.41（19298.63-55235.45） | 58325.60（23942.71-90870.98） | 32.28（17.86-46.61） | -0.83   (-0.94--0.71) | 116415.91（44221.79-186966.34） | 106239.48（41354.97-174108.05） | 79.41（31.34-130.44） | -2.08   (-2.2--1.96) |
| Gabon | 6224.36（4094.89-8721.74） | 13648.31（8169.42-21341.35） | 1292.97（794.56-2010.35） | 0.37   (0.19-0.55) | 27.70（12.94-46.81） | 78.98（35.87-151.88） | 7.30（3.28-13.97） | 1.02   (0.86-1.17) | 26.46（10.57-46.29） | 117.50（48.24-211.26） | 13.68（5.70-24.17） | 3.16   (3.03-3.29) | 713.00（44.31-1640.78） | 2074.03（83.43-4476.46） | 179.12（8.71-395.18） | 1.08   (0.85-1.31) | 20.04（4.62-51.46） | 107.34（28.18-242.53） | 11.57（3.05-25.97） | 3.57   (3.43-3.71) | 3943.86（2294.40-5697.26） | 6903.06（4057.43-10505.17） | 691.05（395.41-1079.35） | -0.23   (-0.41--0.05) | 1493.30（544.63-2487.11） | 4367.40（1664.18-7604.34） | 390.26（149.07-677.35） | 1.21   (1.02-1.4) |
| Gambia | 2199.54（1278.33-3223.27） | 10032.46（5462.93-15691.57） | 927.64（518.96-1450.96） | 1.41   (1.2-1.62) | 7.31（2.72-16.79） | 35.38（14.03-73.77） | 3.35（1.33-7.00） | 1.35   (1.14-1.55) | 6.01（2.20-11.01） | 37.69（15.29-65.25） | 4.62（1.76-8.15） | 2.72   (2.64-2.79) | 424.16（27.51-945.66） | 2008.03（107.15-4566.88） | 176.88（10.02-391.98） | 1.58   (1.4-1.76) | 3.51（0.72-11.42） | 24.62（5.19-70.10） | 2.83（0.60-8.02） | 2.87   (2.75-2.99) | 1105.00（698.97-1592.92） | 4669.56（2717.57-6835.08） | 435.11（247.71-638.75） | 1.15   (0.92-1.37) | 653.56（217.84-1097.41） | 3257.18（1194.51-5614.57） | 304.84（110.60-526.99） | 1.7   (1.49-1.91) |
| Georgia | 83934.54（34390.35-138213.31） | 63579.42（37291.53-94809.62） | 1100.95（631.25-1652.87） | -0.79   (-1.18--0.39) | 96.85（49.67-164.60） | 412.26（209.48-719.45） | 7.33（3.76-12.75） | 7.31   (5.98-8.66) | 484.67（188.74-851.46） | 839.23（355.72-1428.07） | 13.32（5.63-22.84） | 1.52   (1.12-1.93) | 14726.32（533.52-31390.53） | 12509.00（914.15-26537.00） | 221.57（15.29-469.18） | -0.53   (-0.89--0.16) | 37.11（6.46-107.39） | 164.97（43.88-413.49） | 2.70（0.71-6.84） | 5.63   (5.02-6.25) | 9615.77（7376.67-12543.24） | 26247.29（17349.47-34303.66） | 434.00（309.91-555.50） | 5.52   (4.34-6.72) | 58973.83（23415.92-96027.20） | 23406.67（9454.65-39199.18） | 422.02（171.48-703.68） | -3.66   (-4.18--3.14) |
| Germany | 898111.55（432126.18-1446647.64） | 646389.12（344717.07-1028805.47） | 325.13（176.80-513.12） | -2.44   (-2.54--2.34) | 8134.66（4309.77-13957.06） | 8413.55（4233.41-14271.92） | 4.72（2.42-7.99） | -1.3   (-1.56--1.03) | 17335.24（6612.42-30679.85） | 41697.19（16784.02-76351.41） | 19.02（7.63-34.65） | 1.38   (1.21-1.55) | 117207.00（11628.44-240862.12） | 74532.36（7000.68-155849.58） | 42.92（3.60-89.36） | -2.7   (-2.89--2.51) | 6837.50（1781.67-18180.60） | 12411.51（3530.15-31302.00） | 5.82（1.65-14.46） | 1.2   (0.41-2) | 169390.65（108702.68-224681.87） | 198362.96（78567.00-294689.41） | 85.13（42.50-119.89） | -0.25   (-0.6-0.09) | 579206.51（225384.28-956052.52） | 310971.55（119748.54-510930.07） | 167.52（65.90-270.39） | -3.5   (-3.63--3.38) |
| Ghana | 41843.64（25526.10-61509.86） | 150667.76（82711.74-241694.53） | 809.60（447.73-1294.90） | 0.8   (0.6-1.01) | 154.01（64.42-297.56） | 671.07（269.13-1369.86） | 3.83（1.51-7.78） | 1.28   (1.04-1.53) | 54.25（17.26-109.97） | 744.63（287.58-1267.75） | 6.04（2.22-10.96） | 5.62   (5.44-5.8) | 6696.24（443.37-15633.27） | 41720.01（2371.68-91965.86） | 210.32（12.81-453.79） | 3   (2.73-3.27) | 61.57（14.92-181.31） | 762.56（176.75-1929.68） | 5.70（1.26-14.35） | 4.96   (4.83-5.09) | 22560.48（13844.21-31536.27） | 66851.88（40176.36-92699.84） | 366.62（221.46-517.49） | 0.21   (-0.03-0.45) | 12317.10（4408.92-20158.26） | 39917.62（14731.67-70347.61） | 217.10（78.56-379.79） | 0.17   (-0.19-0.52) |
| Greece | 69983.44（28901.31-117251.17） | 93406.37（47472.65-149858.11） | 416.47（200.23-675.81） | -0.54   (-0.74--0.33) | 837.81（444.84-1455.32） | 1645.40（854.88-2825.93） | 7.78（4.03-13.36） | 0.42   (0.03-0.81) | 943.74（373.60-1660.82） | 3203.80（1267.39-6114.21） | 11.56（4.54-21.80） | 1.74   (1.53-1.96) | 14415.01（1180.43-30609.64） | 13979.12（935.79-30275.96） | 62.89（3.16-137.72） | -1.95   (-2.16--1.74) | 430.93（106.52-1060.11） | 1057.84（279.04-2488.32） | 3.76（0.99-8.85） | 0.84   (0.65-1.03) | 7806.51（4683.09-10902.23） | 20968.87（8966.59-30952.20） | 71.42（41.05-97.73） | 1.67   (1.14-2.2) | 45549.45（17537.92-75266.17） | 52551.34（20942.63-86538.84） | 259.06（106.23-420.56） | -0.73   (-0.97--0.49) |
| Greenland | 428.02（176.00-713.56） | 382.12（161.32-624.01） | 534.53（229.79-880.20） | -2.38   (-2.46--2.29) | 2.36（1.27-4.17） | 2.26（1.11-4.00） | 3.26（1.58-5.87） | -2.7   (-2.89--2.5) | 5.93（2.38-10.79） | 13.37（5.51-23.84） | 22.01（9.17-39.28） | 0.16   (0.06-0.27) | 96.49（2.82-215.44） | 78.93（2.87-166.52） | 113.26（4.87-237.83） | -2.66   (-2.76--2.56) | 1.97（0.38-5.83） | 3.04（0.76-8.25） | 4.69（1.18-12.67） | -1.54   (-1.67--1.4) | 62.79（49.02-80.05） | 69.53（52.10-89.29） | 99.37（73.88-126.86） | -1.06   (-1.31--0.82) | 258.49（102.88-419.35） | 214.99（84.58-357.14） | 291.94（113.76-490.36） | -2.78   (-2.93--2.63) |
| Grenada | 526.56（293.29-791.58） | 934.81（578.72-1348.09） | 805.49（509.27-1165.53） | 0.24   (-0.05-0.54) | 3.48（1.85-6.18） | 8.32（4.46-14.23） | 7.13（3.80-12.17） | 0.54   (0.05-1.03) | 3.02（1.12-5.48） | 12.19（4.97-21.32） | 11.74（4.73-20.96） | 3.36   (3.22-3.49) | 103.10（7.63-221.28） | 143.28（6.36-299.60） | 121.27（5.80-251.43） | -0.67   (-0.9--0.45) | 2.12（0.67-5.40） | 7.90（2.30-18.49） | 7.40（2.18-17.50） | 3.06   (2.95-3.17) | 192.06（148.35-237.78） | 421.05（338.89-501.42） | 371.21（286.67-452.73） | 1.43   (1.1-1.75) | 222.79（85.22-359.35） | 342.07（135.85-540.69） | 286.73（113.05-453.60） | -0.66   (-1.02--0.3) |
| Guam | 1170.03（668.62-1704.48） | 2324.88（1142.01-3555.41） | 1168.06（575.22-1788.55） | -0.13   (-0.44-0.18) | 8.73（4.49-14.81） | 10.93（5.51-18.98） | 5.54（2.80-9.60） | -2.01   (-2.13--1.88) | 7.18（2.77-13.51） | 25.91（10.80-47.13） | 12.15（5.06-22.07） | 0.62   (0.41-0.82) | 147.83（3.51-317.71） | 287.41（5.87-584.49） | 149.19（2.78-297.97） | 0.04   (-0.18-0.27) | 1.05（0.29-2.38） | 5.37（1.57-11.57） | 2.57（0.77-5.48） | 1.9   (1.11-2.69) | 427.25（273.35-536.51） | 422.88（334.06-613.90） | 207.24（164.56-300.50） | -2.99   (-3.6--2.38) | 577.99（241.06-931.30） | 1572.37（666.99-2474.47） | 791.39（335.83-1242.73） | 1.22   (1.02-1.41) |
| Guatemala | 20113.19（8406.21-32459.40） | 49087.71（19734.44-82887.31） | 430.49（179.40-725.85） | -0.75   (-1.21--0.27) | 26.61（13.84-47.19） | 106.59（55.19-187.62） | 0.91（0.47-1.60） | 0.34   (0.09-0.59) | 190.28（74.07-334.26） | 1159.59（486.96-2095.10） | 11.46（4.81-20.61） | 1.85   (1.73-1.96) | 3546.00（127.28-7501.36） | 9346.20（108.51-20077.28） | 76.47（1.30-163.69） | -0.94   (-1.27--0.61) | 21.61（4.82-57.34） | 91.58（21.53-234.63） | 0.90（0.21-2.29） | 0.45   (0.31-0.59) | 2625.15（2213.31-3086.68） | 4776.85（3490.67-6113.64） | 46.77（31.47-61.39） | -1.28   (-1.79--0.76) | 13703.53（5560.25-21898.84） | 33606.91（13785.01-55305.06） | 293.99（118.98-486.12） | -0.66   (-1.22--0.09) |
| Guinea | 15814.74（9575.86-23281.06） | 39489.31（23538.85-58740.67） | 639.06（387.01-944.74） | 1.19   (1.08-1.3) | 38.08（14.92-81.73） | 104.66（38.39-219.28） | 1.75（0.62-3.73） | 1.28   (1.18-1.38) | 36.75（11.81-72.37） | 138.05（51.64-254.43） | 2.87（1.04-5.38） | 2.81   (2.71-2.91) | 2496.89（145.88-5736.37） | 7529.41（456.12-16528.37） | 116.84（7.40-255.15） | 1.9   (1.8-1.99) | 21.51（4.55-70.13） | 90.81（20.52-269.81） | 1.80（0.42-5.37） | 3.07   (3.03-3.11) | 9315.72（4882.11-14213.27） | 20343.11（10986.38-30224.77） | 330.78（177.17-481.93） | 0.65   (0.47-0.83) | 3905.79（1336.02-6376.06） | 11283.27（3673.20-19178.05） | 185.01（60.04-310.81） | 1.88   (1.78-1.97) |
| Guinea-Bissau | 3245.24（1685.65-4830.27） | 8983.37（5073.98-13855.80） | 1027.47（589.78-1528.09） | 1.12   (0.99-1.25) | 7.60（3.32-16.19） | 21.07（9.17-40.51） | 2.59（1.11-5.17） | 0.92   (0.81-1.03) | 4.29（1.55-8.75） | 19.49（7.61-33.18） | 3.48（1.41-6.17） | 3.35   (3.3-3.4) | 484.20（18.75-1246.00） | 1742.47（102.62-3929.96） | 187.92（12.71-403.50） | 1.99   (1.94-2.04) | 2.66（0.62-8.55） | 11.42（2.84-30.36） | 1.87（0.45-4.95） | 2.79   (2.75-2.83) | 1881.39（857.41-2890.87） | 4546.77（2223.59-6949.80） | 525.19（257.42-810.74） | 0.67   (0.48-0.85) | 865.09（289.56-1552.94） | 2642.14（945.59-4598.77） | 306.42（108.87-520.43） | 1.5   (1.41-1.59) |
| Guyana | 6733.58（4252.20-9720.72） | 10194.14（6311.80-15148.07） | 1496.01（923.95-2204.30） | 0.17   (-0.04-0.38) | 9.23（4.90-15.50） | 41.24（19.78-73.30） | 6.17（2.97-10.94） | 2.76   (2.12-3.4) | 16.32（6.10-28.59） | 61.74（25.12-110.65） | 10.60（4.28-18.83） | 2.88   (2.76-3) | 1101.12（77.58-2445.19） | 1433.12（59.96-3089.86） | 204.43（9.75-436.17） | -0.31   (-0.48--0.13) | 6.77（2.05-16.80） | 27.67（7.96-57.74） | 4.56（1.31-9.56） | 3.24   (3-3.48) | 3264.03（2609.26-3926.67） | 5320.75（3965.39-7087.39） | 794.66（576.88-1060.21） | 0.56   (0.16-0.96) | 2336.11（877.52-3891.86） | 3309.61（1298.76-5474.98） | 475.59（186.67-791.97） | -0.24   (-0.45--0.02) |
| Haiti | 19234.09（10171.04-29912.60） | 61588.43（31106.82-99525.34） | 727.23（391.46-1142.26） | 1.28   (1.18-1.37) | 35.99（15.08-78.06） | 150.81（65.10-313.93） | 1.97（0.86-4.04） | 2.15   (2.09-2.21) | 20.61（3.78-43.80） | 193.45（79.03-360.14） | 2.90（1.12-5.39） | 5.49   (5.36-5.62) | 1967.20（-200.35-5817.88） | 10401.45（576.04-22818.99） | 110.29（7.46-238.26） | 3.01   (2.87-3.15) | 16.22（5.02-46.81） | 78.77（21.75-225.96） | 1.21（0.34-3.35） | 2.56   (2.44-2.68) | 8732.94（3174.96-14818.93） | 23041.35（10227.29-37306.18） | 286.98（130.74-467.14） | 0.6   (0.53-0.66) | 8461.14（2768.85-14191.23） | 27722.61（9866.52-49335.24） | 323.87（116.60-572.03） | 1.45   (1.31-1.59) |
| Honduras | 12975.55（7415.20-20065.67） | 62239.91（32643.63-97266.25） | 975.66（526.18-1505.22） | 1.69   (1.53-1.85) | 21.74（10.46-40.57） | 134.23（59.67-259.63） | 2.10（0.93-4.04） | 2.39   (2.28-2.5) | 96.74（36.21-174.98） | 783.93（305.93-1432.55） | 14.43（5.80-26.84） | 3.37   (3.2-3.55) | 2140.44（127.49-5058.82） | 11266.11（390.68-23565.04） | 164.37（6.48-347.69） | 1.87   (1.69-2.06) | 21.22（5.46-58.20） | 104.15（27.82-259.67） | 1.86（0.51-4.68） | 1.36   (1.27-1.44) | 5180.11（3923.75-6970.26） | 20049.41（14777.97-27202.26） | 326.55（235.11-450.74） | 1.02   (0.87-1.16) | 5515.31（2089.04-9023.96） | 29902.07（11573.97-50035.31） | 466.34（178.67-770.42） | 2.15   (1.98-2.32) |
| Hungary | 223579.37（109161.22-353695.77） | 191677.57（112025.75-287348.57） | 1012.24（590.57-1515.66） | -1.48   (-1.61--1.34) | 1282.05（685.07-2126.07） | 1732.56（875.70-2847.31） | 9.46（4.79-15.51） | -0.24   (-0.42--0.06) | 1963.56（778.76-3402.17） | 3150.49（1329.29-5491.90） | 15.09（6.45-26.11） | 0.4   (0.34-0.47) | 44334.95（3813.05-91739.11） | 22124.71（2384.16-43545.25） | 123.45（12.31-240.65） | -3.44   (-3.62--3.27) | 3379.02（855.86-7989.41） | 3148.89（809.90-7185.27） | 16.47（4.20-37.23） | -2.41   (-3.1--1.72) | 45298.25（34434.01-55523.97） | 61055.72（42676.68-77477.61） | 310.20（229.91-384.36） | 0.77   (0.31-1.24) | 127321.54（51705.76-205405.71） | 100465.20（40846.16-164132.27） | 537.56（220.36-871.25） | -1.9   (-2.02--1.78) |
| Iceland | 1498.53（588.89-2552.57） | 1379.26（615.76-2310.99） | 238.52（105.67-395.62） | -2.67   (-2.73--2.62) | 19.03（9.86-32.98） | 25.72（12.82-44.54） | 4.45（2.21-7.69） | -1.77   (-2.06--1.47) | 30.90（12.27-58.61） | 104.35（40.99-192.15） | 16.19（6.43-30.32） | 1.56   (1.44-1.68) | 183.90（15.12-390.67） | 167.14（12.72-360.06） | 30.28（2.03-64.38） | -2.59   (-2.68--2.5) | 9.88（2.34-24.75） | 19.76（5.35-45.98） | 3.10（0.83-7.27） | -0.17   (-0.35-0.02) | 76.96（47.11-104.90） | 160.94（85.21-230.92） | 24.81（14.56-34.72） | 0.71   (0.42-0.99) | 1177.85（469.89-1970.37） | 901.35（356.05-1509.00） | 159.69（63.83-262.95） | -3.31   (-3.39--3.23) |
| India | 920902.45（523530.65-1365265.18） | 4623157.46（2297407.17-6956757.90） | 365.47（188.46-545.29） | 2.67   (2.58-2.76) | 1288.87（532.97-2519.83） | 10403.25（5147.34-18396.74） | 0.85（0.42-1.50） | 4.03   (3.95-4.11) | 1352.34（429.13-2669.15） | 24727.30（9521.16-43933.89） | 2.32（0.88-4.19） | 6.75   (6.59-6.91) | 40312.71（-13620.33-138554.46） | 428951.78（37322.13-913963.76） | 33.48（3.03-70.41） | 4.72   (4.47-4.98) | 810.27（214.63-2187.37） | 7048.36（1783.66-17269.67） | 0.64（0.17-1.57） | 3.94   (3.85-4.03) | 289985.47（160729.01-430766.97） | 1106653.94（761712.74-1570791.61） | 93.42（63.88-132.15） | 1.57   (1.49-1.66) | 587152.79（201839.84-936172.41） | 3045372.82（1142870.82-4931388.31） | 234.77（87.60-380.40） | 2.94   (2.82-3.06) |
| Indonesia | 285958.33（179410.26-401239.28） | 1441911.61（812504.63-2142818.77） | 527.14（310.04-775.64） | 2.69   (2.48-2.91) | 295.83（138.45-544.86） | 1932.47（988.68-3257.57） | 0.81（0.42-1.33） | 3.35   (3.26-3.44) | 212.72（10.68-522.42） | 5363.98（1974.24-9863.49） | 2.32（0.77-4.42） | 8.93   (8.46-9.4) | 14959.11（-17058.35-76281.66） | 283966.07（19849.98-609067.48） | 98.22（7.70-207.76） | 6.9   (6.06-7.73) | 180.14（46.81-499.02） | 1373.93（335.61-3605.33） | 0.61（0.15-1.57） | 3.54   (3.45-3.63) | 162632.81（86300.63-233137.35） | 551509.96（335102.98-755838.53） | 216.25（129.28-304.59） | 1.31   (1.15-1.46) | 107677.73（35231.21-174160.00） | 597765.20（226514.02-957309.72） | 208.93（79.00-335.48） | 3.22   (3.01-3.43) |
| Iran (Islamic Republic of) | 256135.25（144680.72-371540.23） | 732328.68（398637.23-1063738.85） | 931.16（526.08-1349.94） | -0.07   (-0.16-0.02) | 100.03（48.72-173.73） | 961.39（513.42-1646.10） | 1.15（0.62-1.98） | 4.68   (4.32-5.04) | 706.54（276.67-1173.25） | 7061.38（2941.62-12080.77） | 10.54（4.38-18.06） | 3.49   (3.42-3.57) | 32991.84（3506.93-63865.21） | 100651.32（11575.77-196729.84） | 122.47（14.46-240.48） | 0.06   (-0.07-0.2) | 122.47（25.55-347.03） | 970.59（248.47-2197.98） | 1.37（0.35-3.14） | 3.32   (3.13-3.52) | 81781.34（61268.68-108317.41） | 225779.11（172035.77-277482.39） | 304.65（218.62-388.26） | 0.15   (-0.02-0.31) | 140433.03（55547.13-224200.27） | 396904.90（162614.90-623894.12） | 490.98（198.45-778.72） | -0.29   (-0.45--0.13) |
| Iraq | 154207.04（69585.03-252010.47） | 426764.83（189246.45-696747.64） | 1726.95（791.82-2812.84） | -0.66   (-0.8--0.52) | 73.81（34.04-131.22） | 352.36（155.93-623.22） | 1.25（0.56-2.19） | 1.25   (1.19-1.31) | 683.82（251.63-1356.07） | 2602.43（1105.49-4655.39） | 14.47（6.11-26.28） | 1.14   (1.03-1.25) | 35127.19（2366.81-73081.11） | 93133.14（7896.14-188606.44） | 350.82（33.82-724.65） | -0.93   (-1.08--0.78) | 150.72（41.64-345.37） | 446.98（131.89-935.01） | 2.18（0.62-4.61） | 0   (-0.07-0.07) | 35579.04（20653.43-50515.00） | 81875.79（57550.96-110031.73） | 375.81（246.43-509.09） | -1.06   (-1.21--0.91) | 82592.47（32369.51-135479.03） | 248354.13（101848.13-410865.68） | 982.41（397.89-1644.21） | -0.41   (-0.54--0.28) |
| Ireland | 25108.27（9930.98-42299.07） | 16569.81（7341.12-27579.27） | 214.02（94.03-355.46） | -3.6   (-3.76--3.45) | 411.72（211.93-703.56） | 375.68（190.07-664.81） | 4.71（2.41-8.36） | -3   (-3.43--2.56) | 343.19（136.72-588.53） | 1032.13（408.68-1857.31） | 12.38（4.91-22.30） | 1.06   (0.85-1.27) | 2752.38（240.98-5996.23） | 2041.17（139.29-4360.44） | 27.30（1.71-57.70） | -3.15   (-3.32--2.98) | 396.64（99.74-1031.32） | 575.85（160.75-1292.01） | 6.88（1.92-15.45） | -1.29   (-1.48--1.09) | 1009.67（673.42-1349.98） | 1454.66（799.52-2035.86） | 17.78（10.26-24.43） | -0.27   (-0.52--0.02) | 20194.67（7875.15-33198.49） | 11090.32（4401.98-18354.51） | 144.96（57.92-238.38） | -4.19   (-4.36--4.02) |
| Israel | 24730.69（10336.66-40777.44） | 19410.98（8537.94-32688.08） | 155.90（68.16-263.91） | -4.26   (-4.52--4) | 168.96（89.96-294.62） | 261.79（133.15-458.78） | 2.10（1.07-3.71） | -1.96   (-2.13--1.78) | 529.41（219.72-925.92） | 2133.37（907.16-3790.56） | 16.18（6.91-28.94） | 1.46   (1.25-1.68) | 2904.43（206.88-6088.97） | 3314.57（231.03-7156.48） | 27.78（1.79-59.62） | -3.05   (-3.24--2.86) | 294.90（79.35-691.51） | 666.20（178.07-1546.16） | 4.99（1.34-11.65） | -0.55   (-1.01--0.09) | 1980.52（1337.28-2684.08） | 2532.91（1370.73-3622.17） | 18.97（11.24-26.23） | -2.52   (-3.25--1.77) | 18852.46（7349.05-31023.08） | 10502.14（4072.08-17524.45） | 85.88（33.66-142.22） | -5.39   (-5.64--5.14) |
| Italy | 327429.78（171533.88-514704.95） | 408448.10（235392.79-607339.24） | 250.05（146.88-377.35） | -1.48   (-1.55--1.4) | 4704.73（2579.99-7765.31） | 4714.62（2389.95-8443.23） | 3.41（1.78-6.09） | -2.26   (-2.6--1.92) | 5842.36（2241.77-10397.90） | 19002.40（7515.11-35242.12） | 10.76（4.26-19.93） | 1.57   (1.54-1.61) | 49226.43（4896.37-102094.10） | 41028.96（3443.80-90412.04） | 28.30（2.03-61.63） | -2.5   (-2.68--2.31) | 6696.12（1726.08-15936.92） | 9172.79（2570.58-19819.76） | 5.08（1.40-10.94） | -1.39   (-1.59--1.18) | 81839.32（52234.89-111995.33） | 188763.56（74349.47-288894.20） | 101.33（50.64-144.00） | 0.41   (0.29-0.53) | 179120.83（68279.68-298179.53） | 145765.77（56496.33-247598.62） | 101.16（39.37-168.53） | -2.6   (-2.7--2.51) |
| Jamaica | 11405.57（7934.56-15929.17） | 23202.12（14331.98-34570.23） | 739.06（455.42-1101.44） | 0.99   (0.5-1.49) | 33.10（17.37-58.07） | 99.08（47.74-177.30） | 3.20（1.55-5.72） | 1.7   (1.37-2.03) | 104.63（41.49-189.35） | 478.51（209.50-820.15） | 14.66（6.38-25.04） | 3.08   (2.98-3.18) | 2073.37（123.25-4401.85） | 4414.61（200.33-9184.21） | 142.10（6.33-296.32） | 0.84   (0.42-1.27) | 42.03（12.39-121.69） | 166.07（43.17-444.71） | 5.15（1.33-13.75） | 2.69   (2.47-2.91) | 6604.02（5005.91-8462.49） | 12353.78（8936.19-16461.24） | 392.20（284.14-527.44） | 0.96   (0.36-1.57) | 2548.43（989.65-4183.68） | 5690.06（2317.46-9703.64） | 181.75（74.28-309.48） | 1.03   (0.6-1.46) |
| Japan | 203489.29（112094.60-311583.61） | 255690.44（127397.45-402370.90） | 81.64（38.17-130.78） | -1.27   (-1.55--1) | 4022.63（2205.07-6547.93） | 15794.02（8091.34-25202.42） | 4.86（2.55-7.77） | 2.37   (2.31-2.43) | 1154.53（325.19-2492.45） | 5414.60（1909.07-10250.73） | 1.39（0.51-2.62） | 1.94   (1.64-2.24) | 33908.71（3949.46-77471.00） | 48687.10（5469.73-105797.50） | 17.62（1.75-37.63） | -0.71   (-0.87--0.54) | 2395.09（703.87-6036.94） | 6386.48（1896.77-14227.83） | 1.37（0.39-3.10） | -0.3   (-0.43--0.17) | 64151.10（40601.20-91065.65） | 63562.97（28397.78-102206.93） | 15.53（10.15-21.43） | -2.91   (-3.75--2.05) | 97857.23（35211.40-158130.31） | 115845.26（42760.05-185024.18） | 40.86（15.66-65.35） | -1.09   (-1.2--0.99) |
| Jordan | 24176.88（12940.12-36175.67） | 85118.97（49137.36-129668.94） | 1122.27（674.35-1690.48） | -1.81   (-2.11--1.51) | 41.68（19.34-74.35） | 275.71（147.03-489.45） | 3.13（1.63-5.43） | 0.35   (0.15-0.55) | 85.62（34.99-150.74） | 765.63（344.71-1307.15） | 13.62（6.10-23.16） | 1.48   (1.28-1.68) | 4204.91（397.25-8463.84） | 14693.41（1595.43-28701.42） | 182.54（21.31-363.83） | -2.05   (-2.44--1.66) | 15.07（3.80-35.51） | 129.85（38.34-276.65） | 2.09（0.61-4.54） | 1.52   (1.46-1.59) | 8221.46（5851.60-11003.99） | 29545.10（21782.03-37672.43） | 441.04（297.66-576.11） | -1.39   (-1.62--1.17) | 11608.14（4496.21-18893.26） | 39709.27（16819.20-65718.59） | 479.85（201.55-784.61） | -2.16   (-2.52--1.81) |
| Kazakhstan | 148584.96（60012.70-247342.31） | 172061.30（68566.91-294900.52） | 1001.42（406.26-1702.29） | -1.71   (-2.53--0.88) | 412.86（207.64-730.14） | 1085.24（563.00-1934.43） | 5.76（2.99-10.17） | 1.08   (0.64-1.52) | 853.31（354.64-1492.79） | 1851.52（774.69-3281.79） | 12.14（5.13-21.28） | 1.19   (1.06-1.33) | 33168.59（2714.45-70625.32） | 45946.46（3236.99-97427.40） | 255.48（19.15-538.93） | -0.78   (-1.33--0.22) | 104.99（20.91-298.16） | 240.30（58.18-589.97） | 1.52（0.36-3.77） | 1.14   (0.87-1.41) | 16998.10（13847.15-20172.62） | 18663.59（14353.99-24394.43） | 107.38（79.59-140.22） | -1.87   (-3.58--0.12) | 97047.11（37768.37-160270.93） | 104274.19（42981.27-172363.21） | 619.14（254.21-1019.74） | -2.07   (-2.79--1.33) |
| Kenya | 22879.50（16578.79-31038.92） | 117148.21（74993.81-167904.08） | 479.44（308.33-685.52） | 2.35   (2.18-2.52) | 81.20（37.86-151.68） | 512.69（231.38-903.13） | 2.06（0.91-3.63） | 2.49   (2.42-2.57) | 54.08（18.36-102.93） | 696.78（288.92-1203.05） | 3.28（1.30-5.66） | 5.39   (5.35-5.43) | 2081.85（92.56-5418.83） | 19846.39（1098.76-44513.72） | 71.58（4.58-156.04） | 4.17   (3.93-4.41) | 32.70（8.38-92.42） | 364.73（79.84-961.19） | 1.81（0.41-4.70） | 4.53   (4.46-4.6) | 16971.20（11497.43-23074.56） | 65298.19（43060.45-87437.75） | 286.37（175.82-398.55） | 1.58   (1.43-1.73) | 3658.47（1290.50-6092.65） | 30429.45（11772.95-51140.43） | 114.34（43.62-196.43） | 3.8   (3.44-4.15) |
| Kiribati | 694.14（256.55-1170.50） | 1824.65（664.66-3063.48） | 1976.80（742.07-3318.65） | 0.9   (0.84-0.95) | 0.31（0.16-0.56） | 0.97（0.48-1.78） | 1.11（0.56-2.03） | 1.37   (1.27-1.48) | 2.49（1.01-4.66） | 9.28（3.99-15.98） | 13.20（5.43-23.16） | 2.49   (2.43-2.56) | 199.58（-0.89-446.23） | 529.22（-3.78-1107.85） | 547.71（-1.14-1154.60） | 0.93   (0.83-1.03) | 0.87（0.25-2.26） | 3.91（1.22-8.72） | 5.25（1.65-11.81） | 2.72   (2.58-2.87) | 101.87（66.50-129.92） | 193.75（127.13-264.31） | 234.11（154.43-313.76） | -0.14   (-0.17--0.12) | 389.02（149.14-643.78） | 1087.53（465.01-1761.65） | 1175.42（495.80-1914.45） | 1.11   (1.06-1.16) |
| Kuwait | 10324.54（5846.53-14839.55） | 35577.20（17579.73-55390.05） | 951.58（495.99-1482.87） | -1.07   (-1.46--0.67) | 21.43（11.15-36.97） | 111.19（59.65-192.95） | 2.85（1.50-4.87） | 0.91   (0-1.84) | 30.19（12.28-55.80） | 368.31（172.18-625.32） | 15.45（7.00-25.78） | 3.32   (2.99-3.65) | 937.47（74.14-1835.95） | 4434.33（469.07-8438.23） | 109.91（12.39-210.40） | 0.3   (-0.51-1.11) | 5.43（1.52-13.22） | 64.50（21.49-126.88） | 2.78（0.88-5.56） | 4.1   (3.44-4.76) | 2939.48（2467.21-3442.49） | 5417.89（3972.15-7191.90） | 196.80（129.64-273.79） | -2.65   (-2.99--2.31) | 6390.54（2583.22-9856.76） | 25180.99（11252.08-39814.07） | 623.80（267.40-1004.73） | -0.6   (-1.03--0.16) |
| Kyrgyzstan | 34017.77（12892.04-57256.99） | 59464.76（26467.69-95633.73） | 1221.51（551.86-1942.64） | -0.14   (-0.55-0.27) | 16.88（8.50-29.79） | 98.52（51.37-173.33） | 1.88（0.97-3.34） | 4.42   (3.84-4.99) | 161.21（66.27-281.38） | 384.64（152.96-671.48） | 9.00（3.60-15.50） | 1.31   (1.21-1.4) | 8897.44（580.91-19201.32） | 11955.58（923.74-24446.99） | 219.88（18.21-447.71） | -1.73   (-2.33--1.13) | 38.20（6.87-111.42） | 122.31（29.40-310.48） | 2.71（0.67-6.93） | 1.94   (1.35-2.53) | 4113.57（3215.51-5017.00） | 10215.81（8006.78-12828.05） | 214.81（164.14-273.56） | 1.01   (0.7-1.33) | 20790.47（8284.91-33871.79） | 36687.89（14809.88-59378.74） | 773.24（311.50-1260.13） | 0.16   (-0.3-0.62) |
| Lao People's Democratic Republic | 10989.51（5807.69-17006.26） | 29938.58（16692.02-46950.42） | 564.92（319.92-893.42） | 0.56   (0.53-0.6) | 6.59（2.56-13.24） | 28.27（13.97-49.61） | 0.58（0.29-1.01） | 2.19   (2.13-2.25) | 7.18（1.10-17.20） | 95.25（33.97-182.97） | 2.22（0.74-4.24） | 6.38   (6.1-6.66) | 471.65（-550.76-2369.90） | 4769.36（301.65-11286.56） | 85.35（5.96-201.36） | 4.47   (3.93-5.01) | 4.78（1.35-13.30） | 20.16（5.00-57.35） | 0.48（0.12-1.30） | 2.1   (2.02-2.19) | 6040.99（2063.60-9942.95） | 12188.41（7005.65-17179.90） | 243.06（140.67-342.51） | -0.36   (-0.4--0.33) | 4458.32（1483.57-7741.30） | 12837.13（4279.92-22408.00） | 233.24（76.16-406.90） | 0.74   (0.7-0.78) |
| Latvia | 46954.03（16239.47-81289.47） | 37151.70（18850.12-59704.68） | 958.50（473.75-1530.69） | -1.53   (-1.9--1.17) | 183.44（95.89-319.36） | 309.41（167.90-540.80） | 8.69（4.67-15.18） | 1.04   (0.67-1.41) | 346.81（138.41-626.89） | 682.54（282.49-1195.88） | 15.62（6.49-27.61） | 1.74   (1.65-1.82) | 10203.09（973.09-21095.94） | 6949.85（800.02-14417.49） | 180.52（19.60-372.07） | -2.22   (-2.55--1.89) | 224.47（49.61-604.06） | 416.64（105.38-957.15） | 9.62（2.38-21.83） | 1.49   (1-1.98) | 1544.84（1230.87-1832.11） | 8740.13（5707.94-11284.64） | 216.24（157.87-267.10） | 6.79   (5.65-7.94) | 34451.38（13565.70-56871.91） | 20053.13（7916.88-33651.85） | 527.82（209.33-885.72） | -2.65   (-3.06--2.25) |
| Lebanon | 28051.75（13260.27-46278.90） | 37711.25（18225.54-58510.25） | 616.37（293.36-957.17） | -2.44   (-2.8--2.08) | 138.13（47.32-300.44） | 360.94（181.68-639.53） | 6.01（3.03-10.66） | 0.17   (-0.21-0.54) | 148.73（55.52-290.15） | 743.47（300.92-1357.14） | 11.43（4.62-20.81） | 0.93   (0.77-1.09) | 4460.01（261.15-9986.65） | 6087.37（489.56-12074.76） | 100.58（7.99-199.15） | -2.32   (-2.69--1.94) | 41.83（10.11-115.72） | 219.72（66.68-440.10） | 3.58（1.08-7.20） | 2.11   (1.77-2.45) | 6251.67（2387.12-9907.66） | 8212.64（5189.00-11212.50） | 129.90（86.09-173.96） | -3.07   (-3.29--2.86) | 17011.37（6582.68-28217.08） | 22087.12（9113.72-35551.30） | 364.87（151.13-584.60） | -2.34   (-2.76--1.92) |
| Lesotho | 6244.14（4016.24-8951.14） | 17167.89（9838.50-25941.67） | 1568.85（937.00-2294.83） | 3.38   (2.82-3.95) | 14.29（5.86-29.65） | 35.97（13.50-76.60） | 3.32（1.29-7.04） | 2.48   (2.18-2.78) | 33.26（13.12-60.96） | 91.19（40.45-160.76） | 10.49（4.67-18.52） | 3.35   (3.05-3.66) | 974.60（66.67-2192.25） | 3613.31（113.88-8026.25） | 315.77（11.85-692.71） | 4.57   (3.92-5.23) | 9.07（1.69-27.64） | 35.24（6.73-94.15） | 3.45（0.67-9.49） | 4.02   (3.74-4.31) | 4399.03（2890.49-6108.48） | 9962.03（5290.51-14646.35） | 932.26（490.14-1361.03） | 2.69   (2.16-3.22) | 813.89（295.38-1403.06） | 3430.15（1249.46-5926.94） | 303.55（109.45-532.20） | 4.95   (4.26-5.64) |
| Liberia | 9133.39（5639.11-13716.55） | 27719.62（15309.50-43194.07） | 1091.73（616.99-1702.24） | 1.12   (0.99-1.25) | 29.89（11.81-64.42） | 80.96（28.37-179.26） | 3.44（1.21-7.68） | 1   (0.8-1.2) | 27.33（11.01-46.19） | 115.37（46.51-203.70） | 6.57（2.55-11.76） | 3.08   (2.99-3.17) | 1928.51（100.90-4408.50） | 6460.26（186.26-13568.75） | 234.88（9.23-493.28） | 1.17   (1.03-1.32) | 15.85（3.01-50.70） | 65.94（13.95-176.95） | 3.44（0.75-9.49） | 3.05   (2.86-3.25) | 4556.30（2831.51-6396.49） | 12525.57（6882.90-18842.37） | 503.34（274.68-757.18） | 0.85   (0.7-1.01) | 2575.50（989.82-4249.58） | 8471.53（3258.81-14219.07） | 340.06（129.33-574.56） | 1.48   (1.35-1.61) |
| Libya | 18454.62（9458.83-28805.05） | 95021.52（48296.40-148275.93） | 1632.73（874.49-2543.25） | 2.25   (2.09-2.41) | 5.13（2.06-10.53） | 79.49（30.12-171.67） | 1.24（0.48-2.60） | 6.33   (5.93-6.73) | 73.79（27.13-132.65） | 600.13（234.69-1088.34） | 13.72（5.32-25.06） | 4.02   (3.88-4.16) | 2434.69（207.84-5252.65） | 14661.10（1128.22-29024.31） | 235.52（20.78-475.28） | 2.6   (2.46-2.74) | 9.81（1.93-26.57） | 57.80（13.13-135.97） | 1.30（0.30-3.06） | 2.83   (2.73-2.93) | 5920.22（3344.64-9189.34） | 24361.94（13751.42-38589.48） | 471.02（260.00-729.20） | 1.69   (1.56-1.83) | 10010.97（3833.19-16748.65） | 55261.05（23216.34-90358.16） | 909.94（375.21-1478.97） | 2.45   (2.24-2.67) |
| Lithuania | 49045.31（18947.20-82936.28） | 49660.39（23242.31-81293.99） | 880.64（410.01-1433.01） | -0.76   (-1.09--0.43) | 219.95（113.58-377.11） | 484.79（252.03-818.40） | 9.39（4.92-15.90） | 1.86   (1.6-2.12) | 393.16（158.13-706.67） | 1033.53（434.38-1846.25） | 16.13（6.69-28.91） | 1.99   (1.87-2.11) | 6929.62（715.07-14310.61） | 7018.65（763.83-14897.09） | 130.33（13.50-273.14） | -0.61   (-0.95--0.27) | 205.00（43.41-572.41） | 481.25（118.13-1157.07） | 7.76（1.89-18.81） | 1.72   (1.18-2.27) | 2136.89（1654.59-2597.76） | 7220.85（5109.86-9139.24） | 127.75（96.09-160.59） | 4   (3.28-4.73) | 39160.69（15711.47-65488.28） | 33421.32（13450.80-54619.19） | 589.28（239.47-965.52） | -1.39   (-1.73--1.06) |
| Luxembourg | 2665.02（1043.24-4411.28） | 2303.20（1156.29-3764.55） | 209.82（104.71-341.76） | -2.93   (-3.05--2.8) | 36.96（19.75-64.50） | 41.56（20.90-73.76） | 3.90（1.96-6.92） | -2.42   (-2.7--2.14) | 58.95（23.41-101.68） | 182.22（72.16-316.65） | 15.51（6.19-26.92） | 1.35   (1.2-1.5) | 525.40（45.53-1050.03） | 312.31（22.41-678.13） | 29.70（2.00-64.45） | -4.05   (-4.16--3.93) | 23.15（4.79-57.48） | 36.17（9.87-82.85） | 3.19（0.88-7.32） | -0.91   (-1.27--0.54) | 230.15（145.10-319.35） | 460.76（227.96-664.02） | 38.61（21.12-54.35） | 0.11   (-0.12-0.33) | 1790.41（680.33-2947.03） | 1270.19（492.87-2122.34） | 118.91（46.67-198.77） | -3.64   (-3.82--3.47) |
| Madagascar | 28153.81（19500.32-39488.62） | 102388.60（62298.89-150600.75） | 826.71（519.92-1195.50） | 1.31   (1.23-1.39) | 67.40（28.75-132.05） | 242.14（98.98-468.06） | 1.93（0.77-3.70） | 1.26   (1.06-1.46) | 35.75（9.91-76.03） | 284.23（106.03-540.29） | 3.27（1.22-6.11） | 4.41   (4.26-4.56) | 1179.02（-662.03-4663.18） | 12235.19（710.66-29883.35） | 93.29（6.43-222.51） | 4.05   (3.95-4.15) | 21.28（5.06-63.78） | 120.16（24.47-374.37） | 1.37（0.30-3.90） | 3.27   (3.04-3.5) | 23417.87（14193.25-33364.51） | 71253.28（42697.04-104949.02） | 592.02（375.91-866.64） | 0.87   (0.79-0.95) | 3432.49（1143.79-5778.70） | 18253.60（6618.56-33689.57） | 134.83（48.50-245.11） | 2.21   (2.08-2.33) |
| Malawi | 11836.63（7296.08-17266.50） | 42286.45（25783.97-65168.12） | 509.68（321.92-767.28） | 1.55   (1.36-1.74) | 36.63（14.47-80.12） | 164.18（62.62-336.34） | 2.02（0.77-4.13） | 2.36   (2.2-2.52) | 8.52（1.54-21.46） | 140.03（51.83-259.85） | 2.38（0.88-4.52） | 6.41   (6.29-6.52) | 402.47（-419.35-2296.02） | 6684.66（471.14-14846.98） | 75.40（6.22-165.71） | 5.98   (5.49-6.48) | 8.54（1.82-26.40） | 72.89（14.04-211.97） | 1.22（0.24-3.58） | 4.54   (4.46-4.63) | 8552.80（3645.54-13330.40） | 22161.87（11797.25-32596.48） | 280.95（150.50-410.59） | 0.62   (0.46-0.79) | 2827.67（929.42-4658.34） | 13062.83（4980.76-22169.32） | 147.72（56.00-250.54） | 2.36   (2.05-2.67) |
| Malaysia | 47056.71（19876.48-75602.76） | 164469.49（65525.08-262617.46） | 546.42（219.16-875.11） | 0.52   (0.39-0.64) | 231.38（121.84-378.73） | 1356.61（717.54-2292.47） | 4.82（2.55-8.24） | 2.06   (1.9-2.23) | 192.46（73.21-344.19） | 1915.74（804.66-3133.55） | 7.53（3.09-12.44） | 4.3   (4.08-4.52) | 9490.65（673.16-20350.61） | 33490.02（1908.79-70108.59） | 108.89（6.41-228.14） | 0.78   (0.68-0.89) | 83.05（22.05-204.29） | 462.29（117.19-1044.61） | 1.68（0.42-3.83） | 1.8   (1.72-1.87) | 7302.63（4874.12-9503.64） | 17351.52（13119.30-21579.52） | 60.16（44.76-76.29） | -1.32   (-1.6--1.03) | 29756.53（11425.26-46721.20） | 109893.32（43298.79-175242.04） | 363.33（142.21-581.40） | 0.76   (0.64-0.88) |
| Maldives | 495.57（238.41-786.05） | 1209.43（539.35-2019.23） | 271.53（133.14-443.08） | -1.94   (-2.13--1.76) | 0.42（0.21-0.73） | 2.56（0.68-6.27） | 0.60（0.19-1.39） | 0.91   (0.73-1.1) | 0.79（0.25-1.63） | 10.10（3.91-18.89） | 2.79（1.04-5.03） | 4.85   (4.77-4.93) | 98.16（3.00-251.66） | 294.78（15.75-667.17） | 61.73（3.50-137.40） | -1.29   (-1.44--1.15) | 0.27（0.06-0.75） | 1.95（0.47-5.06） | 0.62（0.15-1.62） | 2.26   (2.24-2.29) | 127.71（42.18-202.86） | 254.34（188.74-342.02） | 67.43（48.30-93.97） | -2.32   (-2.54--2.1) | 268.22（94.95-424.12） | 645.70（254.20-1065.92） | 138.35（53.14-231.16） | -2.09   (-2.27--1.9) |
| Mali | 17620.72（9961.86-25572.27） | 43317.80（24818.71-64849.49） | 429.32（254.75-646.52） | 0.15   (0.06-0.24) | 34.26（10.51-82.14） | 90.32（36.00-195.12） | 0.98（0.38-2.13） | 0.34   (0.09-0.6) | 31.44（10.36-62.34） | 117.60（41.59-213.50） | 1.55（0.53-2.74） | 1.64   (1.41-1.87) | 2239.11（66.82-5831.02） | 6837.00（386.08-15280.44） | 64.76（4.02-141.50） | 0.83   (0.61-1.06) | 26.94（5.66-84.52） | 123.99（28.22-339.72） | 1.54（0.36-4.30） | 2.46   (2.31-2.61) | 11458.98（4183.65-17582.61） | 25756.63（11385.34-39188.80） | 255.45（115.88-385.24） | -0.16   (-0.22--0.09) | 3829.99（1221.78-6646.30） | 10392.27（3471.88-17994.24） | 105.04（35.10-180.25） | 0.55   (0.4-0.7) |
| Malta | 2026.69（823.17-3263.61） | 2546.76（1283.46-4141.58） | 274.79（136.72-446.80） | -1.76   (-1.92--1.59) | 13.73（7.34-23.58） | 20.77（10.63-36.78） | 2.23（1.12-3.93） | -1.72   (-2.09--1.36) | 22.57（8.82-39.85） | 113.80（46.91-205.09） | 10.47（4.34-18.92） | 2.31   (2.15-2.47) | 241.20（22.95-497.99） | 247.07（19.61-535.86） | 28.52（1.92-60.97） | -2.35   (-2.56--2.14) | 19.26（4.91-51.11） | 50.80（14.06-122.60） | 4.70（1.31-11.25） | -0.05   (-0.58-0.49) | 184.69（127.86-244.75） | 453.29（248.05-631.14） | 43.82（26.81-59.08） | 0.62   (0.35-0.89) | 1545.24（561.18-2494.61） | 1661.03（647.49-2768.60） | 185.06（72.72-307.52） | -2.22   (-2.4--2.04) |
| Marshall Islands | 433.30（201.64-694.04） | 1215.20（507.33-1992.23） | 2723.44（1182.64-4483.66） | 0.64   (0.51-0.77) | 0.91（0.38-1.75） | 2.76（1.15-5.63） | 6.91（2.95-13.97） | 0.83   (0.75-0.91) | 2.14（0.88-3.92） | 7.65（3.27-13.68） | 23.95（10.22-43.05） | 1.77   (1.69-1.85) | 104.25（-0.48-222.90） | 277.57（-3.32-583.90） | 587.75（-4.65-1256.72） | 0.55   (0.42-0.69) | 0.32（0.10-0.76） | 1.29（0.44-2.85） | 3.74（1.30-8.16） | 1.88   (1.78-1.99) | 100.49（60.77-143.56） | 217.87（128.75-321.69） | 534.88（322.78-768.21） | -0.29   (-0.41--0.18) | 225.20（92.27-361.81） | 708.06（294.72-1159.76） | 1566.21（646.36-2564.71） | 1.04   (0.9-1.18) |
| Mauritania | 10720.61（5947.80-16324.49） | 22778.47（12798.71-36285.48） | 1028.31（576.96-1627.98） | -0.27   (-0.37--0.17) | 35.03（15.15-68.25） | 92.91（32.80-195.68） | 4.35（1.53-9.20） | 0.29   (0.09-0.49) | 48.06（19.29-84.66） | 204.87（80.52-351.49） | 12.45（4.77-21.59） | 2.1   (2.04-2.17) | 2187.71（102.53-4767.51） | 4780.76（234.16-10584.22） | 205.45（11.08-457.25） | -0.18   (-0.33--0.04) | 21.76（4.64-68.17） | 112.79（25.12-316.45） | 6.15（1.36-17.41） | 2.58   (2.48-2.69) | 5272.41（3043.57-7794.31） | 10258.32（6009.74-16658.03） | 465.29（268.67-744.76） | -0.49   (-0.58--0.39) | 3155.64（1129.58-5293.29） | 7328.83（2661.26-12903.48） | 334.61（118.31-588.55） | -0.09   (-0.27-0.09) |
| Mauritius | 7340.11（4354.86-10656.38） | 12014.82（7149.45-17516.82） | 680.41（407.64-994.84） | -1.27   (-1.67--0.87) | 9.56（5.15-15.75） | 22.01（11.54-38.11） | 1.23（0.64-2.14） | 0.17   (-0.26-0.6) | 22.62（9.06-39.45） | 137.78（55.90-237.09） | 7.84（3.17-13.41） | 2.49   (2.3-2.67) | 1131.73（99.71-2386.29） | 1874.84（98.69-3991.10） | 106.75（5.42-228.00） | -1.8   (-2.29--1.3) | 6.86（1.76-17.04） | 79.26（22.69-169.41） | 4.30（1.24-9.06） | 4.4   (3.71-5.09) | 2879.27（2295.32-3450.68） | 4880.94（3902.56-5954.46） | 275.63（217.10-342.22） | -0.78   (-1.45--0.1) | 3290.07（1279.31-5236.86） | 5020.00（2008.37-8192.28） | 284.67（114.08-462.90） | -1.56   (-1.85--1.26) |
| Mexico | 209700.66（101028.27-334937.31） | 758903.27（362899.15-1215230.79） | 590.63（286.69-942.92） | 0.56   (0.31-0.81) | 487.22（261.22-844.17） | 1860.10（964.85-3152.53） | 1.44（0.75-2.45） | 0.57   (0.4-0.74) | 3125.24（1209.10-5904.26） | 19766.18（8322.24-35974.44） | 16.54（6.95-29.97） | 2.28   (2.22-2.35) | 38993.66（2348.01-82881.69） | 100148.01（3442.38-203195.09） | 75.33（2.83-153.25） | -0.47   (-0.62--0.31) | 1220.40（392.96-2722.84） | 3428.19（1040.21-6989.99） | 2.85（0.86-5.80） | -0.85   (-1.08--0.62) | 42337.80（31987.75-53009.73） | 97360.53（66872.97-128508.42） | 80.47（53.12-107.55） | -0.78   (-1.08--0.48) | 123536.34（50392.88-200499.47） | 536340.26（230938.14-859574.79） | 414.00（176.93-663.77） | 1.14   (0.87-1.41) |
| Micronesia (Federated States of) | 1356.72（612.38-2289.31） | 2447.07（1014.79-3953.28） | 2768.58（1177.52-4476.35） | 0.41   (0.37-0.44) | 3.23（1.43-6.47） | 6.40（2.96-12.10） | 7.83（3.71-14.72） | 0.6   (0.56-0.65) | 6.90（2.77-12.35） | 17.66（7.34-30.09） | 25.20（10.19-43.20） | 1.98   (1.96-2) | 333.00（-3.30-748.72） | 561.62（-6.38-1172.23） | 611.12（-4.01-1277.63） | 0.21   (0.15-0.27) | 1.23（0.37-2.91） | 3.40（1.10-7.24） | 4.16（1.29-9.01） | 1.64   (1.47-1.8) | 324.98（186.70-461.80） | 443.89（278.13-618.04） | 540.42（344.13-744.75） | -0.55   (-0.58--0.51) | 687.38（274.72-1154.57） | 1414.10（603.04-2318.88） | 1579.85（660.52-2596.36） | 0.87   (0.83-0.9) |
| Monaco | 305.09（117.61-519.02） | 283.21（137.31-474.73） | 299.81（137.85-502.84） | -1.47   (-1.51--1.43) | 6.74（3.08-12.52） | 8.89（4.39-15.78） | 9.28（4.56-16.43） | -0.2   (-0.36--0.04) | 8.00（2.96-14.80） | 14.58（5.65-27.45） | 13.05（5.06-24.52） | 0.73   (0.63-0.84) | 70.58（6.07-149.67） | 52.07（5.54-110.74） | 58.88（5.09-125.28） | -2.07   (-2.18--1.95) | 5.85（1.36-13.81） | 9.48（2.72-20.45） | 8.35（2.42-18.02） | 0.34   (0.21-0.47) | 36.99（20.25-59.25） | 64.09（30.96-98.73） | 58.00（32.61-84.69） | 0.69   (0.28-1.1) | 176.93（65.73-301.74） | 134.10（52.13-221.64） | 152.25（60.51-251.54） | -2.08   (-2.15--2.01) |
| Mongolia | 11102.83（4196.01-18981.75） | 19295.96（6673.88-33348.59） | 774.13（279.32-1334.88） | -1.35   (-1.69--1.01) | 5.74（2.77-10.41） | 37.36（19.46-67.85） | 1.44（0.75-2.60） | 3.69   (3.39-3.99) | 39.80（15.65-69.74） | 108.90（44.11-191.52） | 5.53（2.23-9.56） | 0.73   (0.61-0.86) | 2369.72（60.81-5432.74） | 4930.07（92.51-10862.31） | 172.89（3.90-379.98） | -0.88   (-1.39--0.37) | 9.44（1.82-28.47） | 75.24（15.69-190.25） | 3.80（0.82-9.68） | 4.82   (4.29-5.34) | 1451.73（873.86-2124.17） | 2081.42（1365.95-3037.56） | 92.13（57.69-136.41） | -1.85   (-2.11--1.6) | 7226.40（2748.94-12149.08） | 12062.97（4681.40-20122.49） | 498.34（189.36-835.29） | -1.45   (-1.78--1.13) |
| Montenegro | 5873.18（2280.45-10193.55） | 10743.74（4336.30-18250.45） | 1149.99（463.34-1949.27） | 0.67   (0.51-0.84) | 111.89（55.51-196.34） | 236.79（113.10-433.18） | 24.35（11.78-44.77） | 1.31   (1.22-1.39) | 144.81（59.26-252.79） | 394.88（172.34-694.65） | 44.18（18.82-77.58） | 2.06   (1.88-2.25) | 1386.55（20.21-3130.95） | 2474.49（41.64-5410.98） | 262.83（4.32-576.09） | 0.41   (0.15-0.68) | 12.53（3.15-28.13） | 25.51（6.87-54.41） | 2.62（0.71-5.59） | 0.81   (0.71-0.91) | 576.82（391.74-805.61） | 1117.20（726.93-1663.06） | 121.75（75.15-179.32） | 0.92   (0.84-1.01) | 3640.58（1500.33-6074.37） | 6494.87（2845.84-10848.97） | 694.27（300.51-1158.85） | 0.65   (0.49-0.8) |
| Morocco | 187717.82（104558.55-288430.09） | 535931.07（283477.94-831531.15） | 1535.24（845.96-2350.01） | 0.68   (0.64-0.73) | 30.39（12.77-60.50） | 327.44（158.24-611.01） | 0.91（0.44-1.69） | 5.75   (5.48-6.03) | 322.60（124.00-609.93） | 2372.02（966.42-4354.86） | 7.94（3.19-14.64） | 3.98   (3.89-4.07) | 23475.29（2002.32-53250.84） | 76624.07（6565.03-162211.36） | 211.73（18.84-447.58） | 1.13   (1.06-1.2) | 106.00（23.26-276.79） | 356.88（92.48-820.97） | 1.11（0.29-2.59） | 0.89   (0.69-1.1) | 65049.17（34160.65-97080.92） | 162263.70（92589.95-239538.12） | 489.60（286.75-725.69） | 0.44   (0.31-0.58) | 98734.36（37441.34-164014.38） | 293986.96（111938.93-493521.59） | 823.95（312.39-1388.33） | 0.7   (0.66-0.75) |
| Mozambique | 23763.94（15131.65-33210.66） | 87397.31（53379.61-135113.61） | 707.42（431.10-1054.45） | 2.47   (2.27-2.67) | 65.22（24.26-151.01） | 315.92（113.10-714.48） | 2.41（0.89-5.36） | 3.03   (2.96-3.1) | 25.87（4.98-61.56） | 272.68（90.57-513.60） | 2.45（0.80-4.73） | 6.26   (6.08-6.44) | 1666.23（-598.46-6046.45） | 19010.87（1051.75-44457.87） | 136.98（8.58-323.01） | 6.19   (5.98-6.41) | 12.93（2.97-39.12） | 109.03（24.42-339.45） | 1.07（0.24-3.30） | 5.1   (4.88-5.31) | 20179.95（11621.58-29030.97） | 57204.98（32844.17-88434.60） | 487.20（276.82-751.69） | 1.72   (1.53-1.91) | 1813.75（622.59-3155.85） | 10483.82（3636.12-18550.31） | 77.31（27.38-134.83） | 3.94   (3.75-4.14) |
| Myanmar | 123665.82（68548.42-199713.25） | 221994.29（129210.84-345133.62） | 421.12（244.55-651.27） | -0.69   (-0.84--0.54) | 82.87（31.08-164.59） | 285.71（148.27-492.90） | 0.57（0.29-1.00） | 1.64   (1.59-1.68) | 110.89（19.50-235.21） | 750.93（251.07-1429.64） | 1.50（0.48-2.97） | 4.06   (3.92-4.2) | 10078.61（-3466.30-33293.05） | 36505.75（2290.81-88307.40） | 66.32（4.44-160.23） | 1.37   (1-1.73) | 80.75（22.09-208.18） | 249.74（64.66-632.44） | 0.52（0.14-1.30） | 1.16   (1.09-1.23) | 62317.13（23290.62-100476.64） | 96445.65（49984.29-146390.85） | 187.96（95.84-286.12） | -1.24   (-1.37--1.12) | 50995.56（16741.96-86918.14） | 87756.52（31491.70-151796.43） | 164.25（58.45-284.13） | -0.67   (-0.84--0.49) |
| Namibia | 5114.02（3430.04-7255.49） | 15886.67（9632.88-24121.05） | 1165.02（713.80-1752.34） | 1   (0.62-1.37) | 13.77（6.73-24.53） | 54.95（25.63-103.27） | 4.02（1.84-7.57） | 1.71   (1.48-1.94) | 16.55（6.44-30.54） | 113.77（45.51-195.12） | 10.97（4.46-19.03） | 3.84   (3.62-4.07) | 748.04（50.75-1645.61） | 2844.87（114.12-6271.08） | 200.18（9.57-431.97） | 1.62   (1.23-2) | 14.21（3.23-44.59） | 91.81（22.16-249.55） | 7.12（1.81-19.34） | 3.57   (3.32-3.82) | 3227.81（2250.11-4424.98） | 8287.69（5440.78-12054.99） | 631.26（398.28-920.96） | 0.44   (0.07-0.81) | 1093.64（384.82-1809.99） | 4493.57（1659.70-7798.44） | 311.48（115.13-534.98） | 1.87   (1.46-2.27) |
| Nauru | 244.60（103.13-382.89） | 356.29（148.68-572.66） | 4730.31（2013.73-7491.18） | 0.53   (0.17-0.88) | 0.67（0.31-1.26） | 1.04（0.49-1.88） | 16.14（7.89-28.78） | 0.86   (0.67-1.04) | 0.99（0.43-1.70） | 1.89（0.83-3.14） | 34.50（14.40-61.45） | 1.88   (1.77-2) | 55.56（-0.65-113.77） | 77.67（-0.87-149.87） | 968.82（-6.62-1883.47） | 0.37   (-0.08-0.82) | 0.13（0.04-0.29） | 0.30（0.09-0.61） | 4.98（1.50-10.35） | 1.82   (1.69-1.94) | 35.54（16.68-51.97） | 39.03（19.13-57.82） | 581.64（284.42-863.54） | -0.45   (-0.72--0.18) | 151.71（64.69-239.26） | 236.35（101.71-372.86） | 3124.23（1330.60-4921.45） | 0.78   (0.44-1.13) |
| Nepal | 20161.66（11166.90-32114.47） | 80870.46（42482.92-129514.06） | 320.55（172.73-501.92） | 2.25   (1.96-2.53) | 20.95（7.04-44.73） | 155.74（70.50-318.05） | 0.63（0.29-1.29） | 4.14   (3.98-4.3) | 13.69（1.54-40.56） | 218.93（72.41-428.53） | 0.94（0.29-1.78） | 7.26   (7.11-7.4) | 1170.81（-439.04-4505.91） | 8151.41（551.40-19277.09） | 31.45（2.21-73.91） | 3.91   (3.75-4.07) | 13.19（3.32-37.82） | 105.74（30.65-276.61） | 0.47（0.14-1.20） | 3.45   (3.31-3.59) | 7484.39（3712.38-11670.55） | 24421.68（16470.58-35024.51） | 102.71（68.67-147.04） | 1.4   (1-1.8) | 11458.64（3294.78-20364.13） | 47816.96（17355.13-84059.06） | 184.34（66.41-320.87） | 2.53   (2.28-2.78) |
| Netherlands | 76556.13（28868.63-130310.26） | 54919.55（24373.35-93200.20） | 157.76（69.00-268.70） | -3.36   (-3.53--3.19) | 1785.94（959.70-3021.83） | 1855.41（948.47-3071.18） | 5.19（2.68-8.58） | -2.56   (-3.03--2.08) | 1695.26（665.74-2956.90） | 4267.57（1613.59-7621.55） | 11.10（4.22-20.13） | 0.65   (0.54-0.76) | 10261.18（995.64-21565.33） | 10396.03（945.06-22427.15） | 31.67（2.60-67.35） | -2.04   (-2.26--1.82) | 1364.81（346.07-3623.94） | 2703.85（692.31-6805.21） | 6.96（1.79-17.50） | 0.07   (-0.05-0.2) | 3550.48（2343.34-4821.97） | 7543.75（3489.89-11293.20） | 19.59（10.00-28.49） | 0.8   (0.53-1.06) | 57898.46（21679.94-96316.16） | 28152.93（10689.86-46588.05） | 83.24（31.87-137.48） | -4.66   (-4.87--4.45) |
| New Zealand | 25901.26（10776.23-42741.73） | 23494.95（10160.11-38024.33） | 288.54（124.80-465.26） | -2.98   (-3.17--2.79) | 684.08（371.11-1160.21） | 589.42（305.93-1034.08） | 7.01（3.64-12.26） | -3.36   (-3.54--3.18) | 502.28（205.06-876.30） | 1750.44（719.38-3114.72） | 19.61（8.15-35.06） | 1.58   (1.43-1.73) | 2855.06（203.02-5875.53） | 3385.93（231.13-7063.42） | 43.18（2.58-88.96） | -1.87   (-2.04--1.71) | 343.68（92.82-811.49） | 690.05（211.25-1418.75） | 7.45（2.30-15.21） | -0.52   (-1.07-0.03) | 1558.93（1178.41-1926.58） | 1835.79（1198.85-2408.22） | 21.80（15.27-27.72） | -2.48   (-2.88--2.08) | 19957.23（7901.50-32755.41） | 15243.32（6266.06-24249.97） | 189.48（78.71-298.41） | -3.52   (-3.69--3.35) |
| Nicaragua | 7686.46（3867.37-12035.60） | 24058.93（11724.40-38611.52） | 482.38（237.69-771.61） | 0.15   (0-0.29) | 9.57（4.83-17.31） | 41.50（20.66-71.00） | 0.82（0.40-1.40） | 1.08   (0.88-1.29) | 104.08（40.80-183.55） | 597.95（243.53-1066.87） | 13.36（5.41-23.98） | 2.02   (1.9-2.14) | 1551.15（73.19-3386.76） | 3770.61（152.54-7919.92） | 70.66（3.26-147.42） | -0.71   (-0.82--0.59) | 10.14（2.39-27.38） | 44.13（10.63-108.55） | 0.98（0.24-2.43） | 0.86   (0.83-0.9) | 2055.80（1566.65-2617.59） | 5342.24（3902.97-7223.58） | 114.12（80.32-155.69） | -0.69   (-0.85--0.53) | 3955.72（1571.76-6342.46） | 14262.50（5868.54-23304.68） | 282.45（114.60-464.07） | 0.74   (0.57-0.92) |
| Niger | 10567.12（5911.56-15709.51） | 35369.35（19042.10-54698.38） | 391.33（218.35-596.63） | 0.31   (0.17-0.46) | 24.13（7.50-61.37） | 77.87（26.15-189.55） | 0.93（0.31-2.26） | 0.01   (-0.15-0.17) | 22.21（7.10-43.34） | 106.43（37.67-209.29） | 1.49（0.49-3.05） | 1.29   (1.14-1.45) | 1632.81（62.72-4124.52） | 6098.74（328.07-13310.86） | 63.94（3.66-138.56） | 0.73   (0.61-0.85) | 9.71（1.45-30.61） | 50.20（8.15-151.86） | 0.69（0.12-2.18） | 1.62   (1.48-1.75) | 6618.55（2410.90-10534.59） | 20255.53（7135.43-32318.99） | 226.05（76.82-358.20） | -0.02   (-0.18-0.14) | 2259.72（684.74-4019.60） | 8780.57（2702.11-16684.13） | 98.23（30.12-185.20） | 0.89   (0.76-1.02) |
| Nigeria | 203875.95（136515.51-291630.22） | 572127.29（320646.69-846883.77） | 603.54（336.04-880.18） | 0.65   (0.48-0.82) | 831.34（305.63-1753.88） | 3272.88（1122.63-7124.67） | 3.61（1.29-7.63） | 1.93   (1.79-2.07) | 781.30（303.47-1418.64） | 4651.21（1866.85-7982.42） | 7.25（2.93-12.45） | 3.3   (3.16-3.44) | 26253.98（2277.18-59547.00） | 99344.63（7407.24-208950.66） | 101.22（8.01-212.94） | 1.46   (1.29-1.64) | 373.82（82.18-1106.51） | 1830.99（371.26-4974.99） | 2.56（0.51-7.02） | 2.78   (2.68-2.88) | 116968.32（77723.62-168839.00） | 241906.93（131764.78-341699.54） | 254.16（146.87-359.53） | -0.51   (-0.73--0.29) | 58667.19（22569.36-97423.94） | 221120.66（81303.38-371956.82） | 234.75（85.58-390.19） | 1.92   (1.78-2.06) |
| Niue | 36.24（17.15-58.67） | 42.47（19.43-68.02） | 2009.05（913.06-3223.15） | 0.23   (0.15-0.31) | 0.14（0.07-0.26） | 0.18（0.09-0.32） | 8.59（4.10-15.10） | 0.58   (0.46-0.7) | 0.26（0.10-0.51） | 0.50（0.21-0.89） | 23.93（10.10-42.38） | 2.12   (2.03-2.2) | 6.85（0.08-15.28） | 7.48（0.04-15.71） | 362.41（0.56-752.91） | -0.12   (-0.22--0.01) | 0.05（0.01-0.11） | 0.11（0.04-0.21） | 5.11（1.69-9.90） | 2.66   (2.49-2.82) | 8.41（5.54-11.62） | 7.41（4.94-9.86） | 347.48（232.57-463.35） | -0.6   (-0.66--0.54) | 20.53（8.16-33.37） | 26.80（11.75-43.43） | 1261.55（554.85-2043.03） | 0.57   (0.47-0.67) |
| North Macedonia | 24917.38（11505.27-39839.57） | 36987.74（18089.05-60858.91） | 1246.19（643.11-2001.18） | -0.4   (-0.7--0.11) | 71.96（37.71-127.21） | 194.84（83.92-375.24） | 5.83（2.57-11.12） | 1.09   (0.9-1.28) | 168.31（65.76-296.36） | 509.09（204.92-939.31） | 18.40（7.59-33.88） | 1.97   (1.67-2.26) | 6419.12（516.26-13325.26） | 10119.06（985.26-21318.91） | 326.25（32.47-692.20） | -0.42   (-0.73--0.11) | 37.30（9.52-95.27） | 90.41（26.32-215.96） | 3.11（0.90-7.44） | 0.87   (0.77-0.97) | 5847.07（3853.31-8001.20） | 10077.32（6468.77-14803.34） | 368.00（205.71-552.64） | 0.44   (0.11-0.77) | 12373.61（5049.83-20360.97） | 15997.02（6561.96-27645.56） | 524.60（214.41-910.42） | -0.98   (-1.28--0.68) |
| Northern Mariana Islands | 323.15（106.94-559.82） | 762.16（309.16-1204.79） | 1296.78（525.02-2070.67） | 0.67   (0.51-0.83) | 2.40（1.21-4.29） | 5.05（2.59-8.52） | 9.04（4.75-15.19） | -1.31   (-1.63--0.99) | 2.71（1.14-4.80） | 12.85（5.73-21.33） | 27.47（12.16-46.85） | 2   (1.88-2.12) | 104.56（-0.75-229.95） | 166.74（-0.59-332.94） | 284.26（0.19-574.22） | -0.65   (-0.85--0.44) | 0.28（0.09-0.58） | 2.13（0.66-4.19） | 4.04（1.22-8.11） | 3.12   (2.73-3.51) | 27.20（18.61-37.62） | 56.91（44.68-67.66） | 103.33（79.20-126.22） | -0.3   (-0.4--0.19) | 186.00（76.46-308.53） | 518.48（239.18-794.16） | 868.64（397.12-1336.22） | 1.34   (1.16-1.52) |
| Norway | 31145.94（12050.76-51855.19） | 14469.71（6257.97-24146.36） | 143.41（61.00-240.11） | -4.17   (-4.28--4.06) | 809.60（440.99-1352.38） | 668.01（351.19-1146.33） | 6.46（3.43-11.11） | -2.66   (-2.96--2.36) | 577.32（225.74-960.83） | 1062.31（419.95-1807.22） | 9.51（3.73-16.16） | 0.39   (0.26-0.53) | 3938.28（398.24-8254.83） | 2321.28（208.18-4843.37） | 24.25（1.97-50.34） | -3.22   (-3.33--3.11) | 723.44（201.30-1684.54） | 481.27（126.55-1052.19） | 4.19（1.10-9.25） | -3.16   (-3.51--2.81) | 1393.61（869.46-1938.51） | 1531.75（719.85-2310.02） | 13.21（7.13-19.13） | -0.82   (-1.4--0.23) | 23703.69（9133.20-38973.30） | 8405.08（3219.05-13820.23） | 85.80（33.27-140.56） | -5   (-5.1--4.89) |
| Oman | 10236.89（5231.60-16605.33） | 32132.23（17235.72-47307.96） | 1416.60（804.00-2074.10） | 0.7   (0.54-0.86) | 2.25（0.98-4.44） | 51.62（19.59-115.65） | 1.80（0.71-4.02） | 7.71   (7.22-8.21) | 24.15（9.69-41.48） | 230.61（95.55-398.53） | 15.13（6.39-26.18） | 4.7   (4.5-4.9) | 1242.25（94.16-2682.58） | 4777.86（470.62-9003.02） | 171.46（18.65-331.30） | 1.07   (0.9-1.24) | 2.64（0.58-7.23） | 24.89（6.50-55.20） | 1.53（0.41-3.47） | 4.26   (4.12-4.39) | 3192.47（2054.43-4767.61） | 9309.20（6343.67-12707.50） | 498.82（333.96-675.71） | 1.11   (0.74-1.48) | 5773.12（2012.67-9981.97） | 17738.06（7449.79-28153.76） | 727.87（292.91-1182.40） | 0.31   (0.06-0.55) |
| Pakistan | 184542.23（102902.28-285094.20） | 999262.53（509713.06-1609589.20） | 707.26（376.05-1111.88） | 2.85   (2.63-3.08) | 320.10（134.80-610.05） | 2209.97（1089.74-3988.46） | 1.71（0.85-3.09） | 3.71   (3.5-3.91) | 189.85（37.58-446.27） | 4136.55（1555.99-6993.97） | 3.90（1.48-6.89） | 8.93   (8.35-9.51) | 11447.60（-182.01-34806.91） | 124957.95（8561.49-273999.03） | 86.55（6.24-187.34） | 5.37   (4.93-5.82) | 246.40（67.29-636.98） | 1363.12（378.29-3199.16） | 1.28（0.36-3.06） | 3.5   (3.13-3.87) | 65694.43（38073.86-94840.37） | 227920.22（152006.47-326158.74） | 185.96（121.14-272.14） | 1.4   (1.06-1.73) | 106643.85（39199.49-179152.01） | 638674.73（230678.48-1071443.78） | 427.86（154.24-723.78） | 3.24   (3.06-3.42) |
| Palau | 198.03（69.50-338.73） | 468.36（184.08-793.52） | 1917.26（751.60-3217.41） | 0.37   (0.29-0.44) | 0.64（0.31-1.27） | 1.80（0.94-3.21） | 7.88（4.17-13.99） | 0.76   (0.68-0.85) | 1.12（0.46-2.03） | 3.70（1.65-6.52） | 18.86（8.09-33.37） | 1.44   (1.39-1.5) | 49.75（0.42-107.57） | 106.43（0.97-217.23） | 438.61（5.25-897.86） | 0.18   (0.12-0.24) | 0.25（0.07-0.54） | 1.00（0.33-1.91） | 4.54（1.49-8.76） | 1.85   (1.66-2.05) | 16.28（11.64-22.14） | 31.49（23.21-40.80） | 133.36（97.08-175.11） | -0.51   (-0.56--0.46) | 129.99（51.23-211.34） | 323.94（140.33-533.01） | 1314.01（564.14-2153.33） | 0.51   (0.43-0.6) |
| Palestine | 14623.37（7905.79-23894.90） | 35188.80（18061.74-51618.86） | 1397.72（773.04-2054.68） | -0.7   (-0.88--0.52) | 10.22（4.86-19.45） | 56.08（28.48-98.42） | 1.96（1.00-3.56） | 1.95   (1.84-2.06) | 59.54（22.26-113.88） | 289.24（121.30-495.74） | 16.35（6.77-27.68） | 2.03   (1.89-2.17) | 2334.91（213.15-5105.68） | 5979.60（578.92-11283.29） | 223.09（24.19-435.31） | -0.54   (-0.76--0.32) | 6.48（1.40-16.95） | 28.87（7.09-71.46） | 1.34（0.33-3.28） | 1.56   (1.5-1.62) | 4630.26（2877.08-6943.76） | 8711.73（6235.30-11172.80） | 403.91（260.58-538.63） | -1.1   (-1.24--0.95) | 7581.96（3043.77-13470.24） | 20123.29（8567.44-31212.09） | 751.07（317.61-1188.97） | -0.55   (-0.75--0.36) |
| Panama | 6273.43（2729.54-10638.03） | 18795.16（9113.69-30287.41） | 421.74（204.34-679.54） | -0.1   (-0.31-0.12) | 55.79（28.36-96.87） | 198.29（100.23-349.13） | 4.51（2.28-7.93） | 0.11   (-0.1-0.31) | 147.11（59.71-272.41） | 863.65（362.79-1509.75） | 19.04（7.99-33.31） | 1.86   (1.8-1.92) | 1167.58（96.59-2609.33） | 3762.99（179.46-7899.49） | 84.83（3.99-177.91） | 0.01   (-0.14-0.16) | 58.80（15.38-146.17） | 212.44（55.52-481.65） | 4.68（1.22-10.58） | -0.31   (-0.67-0.05) | 924.08（694.30-1144.75） | 4050.79（2756.18-5322.26） | 90.46（61.98-118.45） | 0.78   (0.28-1.28) | 3920.06（1526.10-6543.21） | 9706.99（4106.34-16093.26） | 218.23（92.33-361.30） | -0.54   (-0.81--0.27) |
| Papua New Guinea | 13359.80（6518.15-23887.50） | 49956.26（23164.68-91493.56） | 729.53（344.79-1313.77） | 0.69   (0.6-0.79) | 24.00（9.72-49.10） | 112.61（53.00-215.79） | 1.73（0.82-3.35） | 1.25   (1.18-1.33) | 28.83（8.83-60.87） | 186.88（70.49-370.21） | 2.90（1.07-5.64） | 2.55   (2.46-2.65) | 1984.84（3.88-5918.37） | 9378.39（276.78-24155.91） | 131.92（4.62-342.43） | 1.31   (1.13-1.48) | 8.31（2.16-22.42） | 35.95（9.34-93.17） | 0.68（0.19-1.68） | 1.04   (0.96-1.13) | 4898.25（2360.48-7894.44） | 13384.81（7187.26-22305.67） | 207.21（115.01-349.31） | -0.31   (-0.35--0.27) | 6415.56（2254.64-11904.04） | 26857.62（10292.89-49724.95） | 385.08（147.18-706.42） | 1.13   (0.98-1.28) |
| Paraguay | 13170.30（6716.70-21266.07） | 38667.15（20531.74-64384.91） | 649.33（351.00-1078.62） | 0.55   (0.45-0.64) | 76.01（38.84-127.88） | 392.78（191.12-723.12） | 6.54（3.19-12.07） | 2.36   (2.23-2.48) | 133.37（46.94-250.77） | 788.21（313.33-1476.12） | 14.27（5.63-26.86） | 2.82   (2.72-2.92) | 2733.37（141.55-6132.23） | 7048.72（283.88-15627.96） | 115.27（5.19-255.46） | -0.04   (-0.13-0.05) | 29.99（7.93-77.43） | 132.62（38.02-296.46） | 2.36（0.68-5.32） | 1.76   (1.7-1.82) | 3839.87（2863.99-4983.74） | 11406.30（7967.95-15646.89） | 198.20（136.05-273.43） | 0.63   (0.51-0.75) | 6357.69（2473.69-10614.10） | 18898.52（7550.05-32255.11） | 312.68（123.79-536.16） | 0.62   (0.46-0.78) |
| Peru | 42512.70（19803.88-72095.52） | 93738.38（42156.79-155971.08） | 268.79（122.33-447.57） | -0.98   (-1.39--0.58) | 100.62（45.73-186.44） | 396.80（186.25-695.51） | 1.15（0.54-2.02） | 1.18   (1.03-1.33) | 462.70（168.73-882.65） | 3035.21（1251.19-5432.02） | 9.10（3.74-16.24） | 2.59   (2.49-2.69) | 8823.38（472.99-21188.28） | 21471.39（638.34-46717.75） | 60.06（1.92-130.52） | -0.73   (-1.09--0.36) | 44.77（10.21-126.29） | 276.77（66.46-677.19） | 0.84（0.20-2.06） | 2.56   (2.42-2.71) | 9950.41（7240.93-12932.68） | 18672.78（12800.49-26390.47） | 54.81（37.35-77.92） | -1.44   (-1.73--1.15) | 23130.84（8968.32-39074.64） | 49885.42（19964.84-84724.14） | 142.82（56.90-243.11） | -1.07   (-1.56--0.57) |
| Philippines | 155272.23（103781.89-215254.94） | 674767.31（386301.37-1007194.30） | 734.32（442.13-1080.78） | 1.69   (1.58-1.81) | 210.77（112.04-342.77） | 1048.44（561.16-1738.36） | 1.21（0.66-1.99） | 1.9   (1.8-2) | 332.88（110.52-621.33） | 3166.75（1198.85-5548.47） | 4.28（1.59-7.66） | 4.17   (3.92-4.41) | 13195.10（384.49-32304.26） | 94944.20（5404.46-207653.51） | 98.24（5.82-211.80） | 3.42   (3.08-3.76) | 93.17（24.59-260.78） | 578.06（128.38-1615.02） | 0.74（0.17-2.06） | 2.5   (2.4-2.6) | 71259.97（54987.02-87457.46） | 281426.59（211302.00-355909.39） | 321.32（234.10-411.39） | 1.4   (1.28-1.52) | 70180.33（25089.99-114125.50） | 293603.28（107184.31-484780.49） | 308.53（111.83-507.56） | 1.55   (1.45-1.65) |
| Poland | 500795.73（221722.25-821393.56） | 429986.65（228806.11-664245.50） | 602.10（316.84-933.08） | -2.38   (-2.54--2.23) | 4009.37（2089.86-6685.72） | 4978.71（2565.40-8682.17） | 7.10（3.66-12.38） | -1.22   (-1.58--0.86) | 5524.54（2235.03-9739.57） | 12557.21（5126.96-22382.61） | 16.28（6.64-29.05） | 0.54   (0.15-0.94) | 84533.82（7944.38-177379.10） | 63299.17（6238.44-131415.38） | 91.88（8.41-190.20） | -2.85   (-2.99--2.71) | 5464.12（1496.15-12658.81） | 13305.65（4322.16-27667.54） | 17.30（5.62-36.08） | 0.8   (0.62-0.97) | 63955.15（49314.77-77981.53） | 107121.39（71758.38-138898.21） | 148.20（103.98-189.19） | -0.06   (-0.36-0.24) | 337308.73（135775.24-551069.16） | 228724.52（90181.18-373930.90） | 321.34（127.06-524.42） | -3.16   (-3.33--3) |
| Portugal | 57606.24（21515.79-101874.11） | 54803.61（27763.95-92633.02） | 227.12（107.58-381.29） | -2.52   (-2.73--2.31) | 335.80（185.32-570.63） | 612.26（316.79-1069.16） | 2.77（1.43-4.86） | 0   (-0.23-0.23) | 738.92（260.55-1426.83） | 2733.66（1034.66-5041.69） | 9.49（3.65-17.65） | 1.62   (1.48-1.77) | 18753.57（1696.70-40076.50） | 10983.09（805.74-23980.46） | 47.92（2.88-104.69） | -4.01   (-4.22--3.8) | 1198.84（341.37-2944.00） | 2405.56（717.70-5299.77） | 8.23（2.48-18.27） | -0.64   (-0.94--0.34) | 6972.00（4427.12-9528.72） | 13514.31（5503.84-20437.87） | 45.92（23.75-65.77） | -0.44   (-0.59--0.29) | 29607.11（11353.23-49161.92） | 24554.72（9413.15-41497.91） | 112.80（43.92-185.87） | -2.75   (-3.07--2.44) |
| Puerto Rico | 26052.06（13739.56-40180.04） | 30266.64（17785.06-44469.85） | 475.84（277.07-706.35） | -1.63   (-1.81--1.45) | 133.29（71.02-233.29） | 143.52（74.10-241.05） | 2.14（1.13-3.59） | -2.48   (-2.74--2.22) | 397.85（153.56-751.72） | 1314.08（561.11-2318.74） | 16.57（7.10-29.04） | 0.95   (0.83-1.06) | 2484.73（115.90-5234.20） | 2731.97（131.72-5538.32） | 47.95（1.56-95.80） | -1.83   (-2.07--1.58) | 175.30（52.99-396.86） | 654.87（197.90-1283.15） | 8.36（2.58-16.27） | 1.28   (1.06-1.51) | 6061.54（4855.09-7207.27） | 10076.69（7150.31-13267.33） | 148.65（116.08-186.73） | -0.14   (-0.48-0.21) | 16799.36（6826.83-27220.71） | 15345.51（6501.17-25189.49） | 252.18（108.64-405.40） | -2.49   (-2.76--2.22) |
| Qatar | 2227.45（959.60-3558.53） | 9999.76（4405.02-15944.06） | 801.56（393.89-1252.47） | -2.84   (-3.39--2.28) | 4.65（2.23-8.49） | 44.57（20.64-86.51） | 3.00（1.38-5.73） | -0.07   (-0.43-0.3) | 7.42（2.60-14.31） | 98.93（43.73-170.71） | 17.43（6.71-32.18） | 0.83   (0.38-1.29) | 351.18（12.80-730.43） | 2049.68（126.83-4077.64） | 121.86（10.45-248.54） | -2.03   (-2.54--1.52) | 1.36（0.37-3.14） | 36.92（12.31-71.45） | 5.81（1.98-10.36） | 5.17   (4.6-5.75) | 293.26（215.81-372.95） | 1228.03（878.37-1786.68） | 154.21（96.21-215.63） | -2.52   (-3--2.05) | 1569.58（644.03-2518.56） | 6541.64（2934.84-10434.42） | 499.25（209.44-818.99） | -3.21   (-3.81--2.59) |
| Republic of Korea | 46404.29（28519.67-77263.42） | 81042.91（39604.40-129634.46） | 90.99（44.64-145.04） | -2.21   (-2.36--2.05) | 342.65（133.81-686.15） | 1243.46（612.16-2125.38） | 1.36（0.67-2.30） | -0.02   (-0.28-0.23) | 88.97（16.32-209.89） | 2833.79（1070.67-5417.47） | 3.04（1.16-5.84） | 8.16   (7.84-8.47) | 7685.82（-1545.97-29177.45） | 20147.06（2349.68-44180.54） | 22.47（2.67-48.40） | -1.59   (-1.88--1.29) | 199.04（60.40-544.01） | 1024.81（274.02-2502.54） | 1.08（0.29-2.65） | 0.59   (0.34-0.84) | 21498.67（12661.70-29386.38） | 24234.05（11109.90-41022.97） | 27.47（12.46-46.46） | -3.41   (-3.64--3.17) | 16589.14（5739.76-28826.24） | 31559.73（11675.11-52607.42） | 35.57（13.05-58.82） | -1.78   (-2.03--1.53) |
| Republic of Moldova | 53514.89（19505.43-89996.37） | 85830.52（45756.21-130694.52） | 1445.68（768.33-2208.32） | -0.02   (-0.34-0.31) | 95.27（51.45-166.78） | 295.89（152.08-504.13） | 5.00（2.58-8.50） | 2.73   (2.38-3.08) | 384.51（156.88-666.50） | 1008.46（432.12-1725.82） | 16.60（7.12-28.50） | 1.49   (1.39-1.59) | 10410.48（435.51-21969.94） | 12002.31（818.59-24328.80） | 203.46（13.42-411.98） | -0.91   (-1.34--0.49) | 101.79（21.96-257.57） | 354.99（93.75-809.37） | 5.73（1.51-13.16） | 2.96   (2.46-3.46) | 3220.10（2657.85-3764.36） | 21734.81（16139.42-27287.89） | 360.00（269.91-450.70） | 5.92   (5.5-6.34) | 39302.74（15504.85-64094.53） | 50434.06（20720.03-81474.44） | 854.90（351.91-1380.93） | -0.93   (-1.32--0.54) |
| Romania | 359786.47（190983.16-551882.14） | 393592.67（225528.27-605662.75） | 1081.65（601.54-1662.17） | -1.09   (-1.3--0.87) | 917.41（495.70-1570.49） | 1875.07（926.38-3347.70） | 5.51（2.75-9.73） | 1.13   (0.89-1.38) | 2222.30（851.54-3909.39） | 4312.59（1761.82-7402.94） | 10.75（4.39-18.43） | 0.35   (0.21-0.48) | 71212.11（4890.93-151811.81） | 70234.92（5602.57-144242.34） | 197.40（13.77-405.50） | -1.7   (-2.07--1.33) | 1990.92（458.85-4688.04） | 6443.67（1891.85-12879.95） | 17.34（4.94-34.94） | 2.01   (1.45-2.56) | 105140.59（80450.03-130039.90） | 135026.91（84590.33-177964.21） | 350.94（237.75-451.91） | 0.25   (-0.11-0.61) | 178303.13（70006.07-286481.34） | 175699.50（68974.31-294167.29） | 499.71（198.13-832.35） | -1.7   (-1.97--1.43) |
| Russian Federation | 2009166.71（690250.91-3391730.02） | 2595014.44（1002186.91-4333793.33） | 1112.96（426.18-1860.04） | -0.73   (-1.38--0.08) | 10288.68（5621.86-17170.96） | 26936.20（14000.60-45615.58） | 11.56（6.06-19.57） | 1.95   (1.58-2.32) | 15294.79（6340.89-25880.62） | 41694.55（17493.96-72255.14） | 17.03（7.16-29.53） | 2.01   (1.85-2.18) | 506372.51（52565.01-1017090.77） | 570301.83（53359.14-1148107.91） | 247.19（22.01-495.28） | -1.33   (-1.92--0.73) | 19602.62（4346.14-54021.98） | 27208.08（7202.16-63762.23） | 10.93（2.88-25.75） | -0.84   (-1.24--0.45) | 97219.61（81579.37-110768.75） | 183315.52（139859.89-222963.17） | 78.14（60.34-94.07） | 0.76   (-0.51-2.05) | 1360388.49（534013.95-2183914.61） | 1745558.25（691155.85-2854398.87） | 748.10（296.58-1223.93） | -0.74   (-1.38--0.1) |
| Rwanda | 16225.96（6471.61-23537.24） | 24080.17（13974.47-36202.05） | 369.30（210.71-548.35） | -2.27   (-2.7--1.83) | 42.26（16.46-90.62） | 89.30（40.43-165.57） | 1.32（0.60-2.41） | -1.02   (-1.47--0.57) | 7.16（0.58-22.28） | 65.54（19.72-132.68） | 1.12（0.32-2.39） | 4.23   (3.93-4.53) | 257.80（-1142.24-2893.16） | 2674.10（116.88-7175.19） | 37.31（2.22-96.39） | 2.69   (2.19-3.19) | 5.93（1.20-16.62） | 26.65（4.03-92.63） | 0.52（0.08-1.64） | 1.82   (1.49-2.16) | 13149.16（2821.33-20604.90） | 15668.59（4282.93-23506.22） | 251.39（70.11-394.66） | -2.8   (-3.21--2.39) | 2763.65（765.67-4794.73） | 5555.99（1844.96-10469.25） | 77.65（25.63-146.52） | -1.53   (-2.01--1.05) |
| Saint Kitts and Nevis | 371.16（184.98-583.74） | 591.39（329.98-879.65） | 840.93（481.85-1241.95） | -0.61   (-0.94--0.28) | 1.75（0.92-2.99） | 3.07（1.50-5.31） | 4.40（2.17-7.65） | -0.52   (-0.73--0.31) | 2.69（0.98-4.98） | 9.39（3.81-16.69） | 16.23（6.45-29.25） | 2.71   (2.56-2.85) | 82.12（5.41-175.51） | 133.99（6.12-276.66） | 182.00（9.66-379.31） | -1.12   (-1.37--0.88) | 1.80（0.52-4.60） | 6.31（1.84-14.12） | 10.52（3.06-24.04） | 2.47   (2.34-2.59) | 107.29（82.28-132.30） | 227.89（173.91-288.28） | 338.60（247.25-438.36） | 1.3   (0.79-1.82) | 175.51（65.64-286.78） | 210.73（86.06-343.94） | 289.17（114.66-472.50） | -2   (-2.28--1.72) |
| Saint Lucia | 652.84（415.09-950.88） | 1414.97（889.79-2042.37） | 591.38（373.02-850.36） | -0.9   (-1.28--0.52) | 9.09（4.93-15.73） | 16.69（8.21-28.51） | 6.97（3.44-11.92） | -1.94   (-2.21--1.67) | 3.80（1.39-7.09） | 25.06（10.06-44.64） | 10.62（4.24-18.87） | 2.37   (2.27-2.48) | 123.92（8.75-261.42） | 261.14（12.51-549.27） | 108.92（5.28-227.89） | -1.18   (-1.42--0.94) | 3.83（1.18-9.79） | 18.14（4.92-43.42） | 7.73（2.10-18.65） | 0.98   (0.66-1.3) | 305.28（240.01-378.90） | 768.26（573.21-998.67） | 322.64（236.46-418.56） | -0.1   (-0.59-0.41) | 206.92（80.03-336.06） | 325.68（130.34-530.95） | 134.50（53.77-219.68） | -2.3   (-2.62--1.97) |
| Saint Vincent and the Grenadines | 495.20（344.63-699.66） | 1245.58（895.55-1697.73） | 896.07（637.97-1224.52） | 0.7   (0.35-1.05) | 1.49（0.78-2.53） | 3.74（1.89-6.41） | 2.63（1.32-4.50） | 0.26   (-0.11-0.63) | 1.88（0.66-3.65） | 11.28（4.42-19.88） | 8.40（3.38-14.90） | 3.75   (3.65-3.85) | 52.37（5.06-120.12） | 136.27（7.40-289.10） | 97.07（5.34-206.12） | 0.7   (0.58-0.83) | 1.20（0.37-3.05） | 4.14（1.25-9.77） | 3.06（0.92-7.28） | 1.89   (1.6-2.19) | 248.94（191.44-312.55） | 772.25（604.70-966.53） | 558.16（425.97-705.61） | 1.55   (1.01-2.1) | 189.32（71.21-309.18） | 317.90（124.55-527.64） | 226.76（88.68-374.59） | -0.81   (-1.01--0.61) |
| Samoa | 1752.55（821.24-2735.90） | 3524.87（1576.85-5617.95） | 2226.02（1007.88-3541.93） | 0.62   (0.54-0.71) | 6.02（2.96-10.72） | 11.99（5.54-22.82） | 7.82（3.69-14.88） | 0.44   (0.37-0.51) | 12.83（5.11-22.51） | 34.16（14.52-58.48） | 24.70（10.40-42.89） | 1.62   (1.58-1.66) | 415.15（-2.59-862.13） | 762.57（-8.50-1551.57） | 466.61（-3.10-943.59） | 0.34   (0.21-0.48) | 2.06（0.60-4.49） | 6.20（1.96-12.74） | 4.33（1.34-8.99） | 2.04   (1.99-2.09) | 442.78（290.80-585.37） | 639.56（427.37-846.06） | 420.62（280.04-553.50） | -0.54   (-0.69--0.4) | 873.71（359.90-1399.79） | 2070.40（894.05-3293.74） | 1301.93（559.82-2077.13） | 1.19   (1.13-1.25) |
| San Marino | 107.23（50.59-178.89） | 126.38（61.97-215.39） | 158.77（75.11-270.38） | -1.54   (-1.81--1.27) | 1.72（0.86-2.98） | 2.21（0.93-4.48） | 3.05（1.27-6.14） | -0.61   (-0.95--0.27) | 3.81（1.51-6.76） | 9.30（3.49-17.24） | 10.56（3.97-19.18） | 0.38   (0.22-0.55) | 20.59（2.02-43.06） | 22.42（2.01-47.67） | 31.63（2.45-67.44） | -1.71   (-1.94--1.49) | 1.76（0.42-4.30） | 3.59（0.91-8.09） | 3.92（0.96-8.76） | -0.1   (-0.32-0.12) | 22.01（11.75-34.10） | 38.33（16.60-60.61） | 41.00（21.78-63.73） | -0.12   (-0.47-0.23) | 57.35（21.63-95.61） | 50.53（18.51-91.74） | 68.60（25.09-122.99） | -2.4   (-2.68--2.11) |
| Sao Tome and Principe | 286.91（167.47-449.76） | 837.99（399.42-1327.15） | 682.81（332.14-1075.72） | 1.24   (1.06-1.42) | 1.24（0.50-2.71） | 4.53（1.67-9.68） | 3.86（1.45-8.23） | 2.16   (2.06-2.27) | 1.32（0.49-2.41） | 6.22（2.56-11.01） | 7.17（2.92-13.00） | 3.88   (3.74-4.03) | 70.97（5.75-157.66） | 248.76（12.83-521.07） | 190.53（10.83-398.31） | 1.61   (1.4-1.82) | 0.58（0.13-1.84） | 3.64（0.80-10.35） | 3.83（0.82-10.59） | 4.68   (4.45-4.92) | 122.19（91.03-156.25） | 258.79（184.29-358.27） | 215.30（152.15-294.08） | 0.23   (0.05-0.42) | 90.62（35.16-150.97） | 316.05（128.51-529.92） | 262.12（104.72-442.98） | 1.95   (1.76-2.13) |
| Saudi Arabia | 109962.36（57821.25-177459.72） | 565848.01（292810.28-871371.05） | 2116.44（1177.84-3148.97） | 0.77   (0.58-0.96) | 11.23（4.79-23.22） | 208.46（102.04-368.38） | 0.71（0.36-1.22） | 5.83   (5.39-6.27) | 278.20（110.84-484.21） | 2120.24（971.03-3574.42） | 15.59（6.84-26.63） | 3.19   (3.08-3.3) | 17142.87（1071.44-37701.83） | 86790.45（5581.59-165835.53） | 295.24（24.96-584.53） | 0.66   (0.51-0.81) | 34.74（7.48-87.05） | 254.49（76.08-529.31） | 1.70（0.47-3.73） | 2.65   (2.52-2.78) | 37962.41（22400.19-57394.81） | 135582.10（73387.59-208491.90） | 661.34（359.51-975.85） | -0.08   (-0.21-0.06) | 54532.90（20955.82-92043.85） | 340892.27（149807.60-538299.41） | 1141.86（485.42-1774.95） | 1.37   (1.11-1.63) |
| Senegal | 19484.22（12106.74-29268.71） | 58758.50（35253.99-87892.13） | 706.20（426.41-1063.91） | 0.71   (0.62-0.8) | 65.76（27.37-131.44） | 203.89（74.68-459.45） | 2.58（0.93-5.82） | 0.53   (0.4-0.66) | 51.17（18.48-91.57） | 276.77（108.47-489.81） | 4.27（1.71-7.54） | 2.71   (2.63-2.8) | 3703.45（249.58-8052.26） | 11549.50（612.14-23618.24） | 132.43（7.54-272.83） | 0.83   (0.72-0.95) | 43.83（9.31-129.17） | 217.91（51.59-611.54） | 3.19（0.76-8.80） | 2.16   (2.03-2.29) | 9346.10（6093.42-13105.19） | 27540.87（17142.07-38646.64） | 332.99（203.21-469.65） | 0.72   (0.61-0.82) | 6273.90（2258.85-10394.17） | 18969.56（6814.77-32288.34） | 230.74（83.13-391.04） | 0.59   (0.5-0.69) |
| Serbia | 126607.16（58347.20-209022.86） | 167102.73（84875.83-261882.26） | 1016.27（511.51-1589.86） | -1.15   (-1.4--0.9) | 884.72（467.79-1557.30） | 1680.92（856.80-2824.74） | 10.40（5.30-17.44） | 0.81   (0.71-0.91) | 801.08（315.99-1506.64） | 2338.75（974.49-4142.92） | 13.40（5.62-23.79） | 1.1   (0.9-1.29) | 31203.56（2743.67-66660.73） | 39038.33（4317.37-80840.94） | 238.13（25.38-490.17） | -1.36   (-1.69--1.04) | 298.61（81.54-694.46） | 693.41（189.61-1589.58） | 3.98（1.10-9.20） | 0.42   (0.19-0.66) | 26103.96（17492.40-37447.56） | 39869.92（23996.37-55791.60） | 234.09（143.24-325.33） | -0.8   (-0.96--0.65) | 67315.22（26251.05-113006.24） | 83481.39（33776.50-139229.34） | 516.26（210.11-854.20） | -1.29   (-1.56--1.02) |
| Seychelles | 687.31（512.20-887.04） | 1276.47（857.48-1745.48） | 1068.00（727.45-1452.48） | -0.38   (-0.51--0.25) | 0.68（0.37-1.14） | 2.17（0.98-4.03） | 1.86（0.83-3.39） | 1.51   (1.32-1.69) | 2.31（0.95-4.02） | 12.68（5.27-21.83） | 11.90（4.78-20.42） | 3.62   (3.46-3.78) | 69.77（5.43-148.82） | 189.75（7.29-387.80） | 151.00（6.32-307.60） | 0.9   (0.78-1.02) | 0.86（0.23-2.04） | 2.76（0.79-6.21） | 2.39（0.66-5.49） | 1.4   (1.32-1.48) | 443.63（338.33-540.58） | 689.97（546.37-876.66） | 594.82（454.14-769.85） | -0.88   (-1.04--0.71) | 170.06（67.61-268.41） | 379.14（152.62-597.31） | 306.04（122.59-486.95） | 0.06   (-0.07-0.18) |
| Sierra Leone | 8699.57（5288.26-12886.71） | 27180.16（14880.99-42293.14） | 647.65（358.72-998.77） | 1.62   (1.42-1.81) | 37.35（14.98-81.76） | 89.60（34.05-209.02） | 2.25（0.86-5.18） | 0.49   (0.3-0.68) | 19.05（6.17-39.25） | 87.90（33.88-158.18） | 2.71（1.05-4.98） | 3.13   (3-3.25) | 1233.65（87.38-2926.69） | 5379.70（377.65-11948.35） | 122.47（8.80-269.54） | 2.61   (2.48-2.75) | 13.94（2.80-41.92） | 54.98（13.06-157.81） | 1.65（0.40-4.64） | 2.66   (2.53-2.79) | 4426.27（2700.00-6381.54） | 12044.62（6280.42-17728.63） | 288.83（156.27-428.00） | 1.16   (0.9-1.41) | 2969.31（977.56-5113.96） | 9523.36（3350.90-16261.83） | 229.73（81.43-398.86） | 1.8   (1.63-1.97) |
| Singapore | 7383.69（4149.45-10796.66） | 17026.18（9833.37-24984.86） | 197.12（114.52-288.49） | -1.45   (-1.59--1.31) | 47.68（25.69-77.30） | 175.61（88.37-300.81） | 2.04（1.02-3.51） | -0.18   (-0.38-0.02) | 9.72（2.50-22.37） | 250.48（98.17-445.56） | 2.87（1.12-5.08） | 6   (5.51-6.5) | 622.67（63.21-1510.02） | 1875.57（111.78-3885.60） | 21.80（1.31-45.25） | -1.02   (-1.33--0.72) | 29.63（9.45-67.13） | 197.45（55.21-429.65） | 2.32（0.65-5.09） | 1.65   (1.49-1.82) | 2139.68（1673.92-2695.07） | 5396.64（4263.63-6642.34） | 62.61（49.04-77.74） | -0.9   (-1.3--0.5) | 4534.30（1645.52-7320.96） | 9130.43（3432.21-14868.17） | 105.47（39.70-171.47） | -1.96   (-2.2--1.72) |
| Slovakia | 88854.54（37062.53-144334.20） | 80601.80（37893.18-127575.03） | 856.63（403.17-1356.25） | -1.81   (-1.91--1.71) | 375.11（192.62-653.33） | 610.92（294.75-1061.88） | 6.60（3.17-11.52） | 0.14   (0-0.29) | 1106.24（459.34-1941.91） | 2330.35（1022.30-4080.69） | 23.84（10.51-41.55） | 0.78   (0.68-0.87) | 14770.18（1157.38-30402.16） | 12258.50（1236.54-24999.84） | 131.10（12.63-267.34） | -2.16   (-2.26--2.07) | 314.41（81.09-702.10） | 676.82（205.85-1340.94） | 6.94（2.09-13.75） | 0.84   (0.72-0.96) | 8111.97（5685.61-11809.68） | 12555.61（8126.42-17694.81） | 132.58（86.26-186.50） | 0.36   (0.09-0.64) | 64176.63（25743.17-103288.49） | 52169.60（22100.96-87438.55） | 555.58（235.59-931.08） | -2.22   (-2.32--2.12) |
| Slovenia | 18333.53（9170.31-28936.63） | 17391.93（9823.87-26208.56） | 364.92（211.32-548.63） | -2.48   (-2.57--2.39) | 169.73（89.09-291.56） | 231.75（115.01-411.95） | 5.36（2.67-9.51） | -1.13   (-1.45--0.8) | 279.17（110.65-494.48） | 700.88（289.80-1217.87） | 14.12（5.83-24.61） | 0.82   (0.61-1.04) | 3918.55（344.11-8080.94） | 2224.04（233.43-4782.17） | 50.35（4.90-106.61） | -4.02   (-4.14--3.89) | 159.46（44.14-350.75） | 520.82（148.66-1042.87） | 10.15（2.87-20.24） | 0.38   (-0.35-1.11) | 4210.71（3069.07-5230.54） | 7138.63（3096.08-10561.03） | 135.55（69.70-193.57） | -0.24   (-0.5-0.03) | 9595.91（3871.82-15616.46） | 6575.83（2598.85-10976.58） | 149.39（60.09-248.18） | -3.62   (-3.8--3.44) |
| Solomon Islands | 2000.61（846.35-3579.80） | 7037.41（2792.97-12108.61） | 1589.88（643.98-2767.08） | 0.96   (0.84-1.08) | 2.31（0.96-4.86） | 9.90（3.92-19.73） | 2.54（1.11-4.92） | 1.49   (1.33-1.64) | 4.35（1.50-9.24） | 25.07（9.51-49.13） | 6.73（2.42-13.48） | 2.95   (2.9-3) | 353.81（10.95-910.59） | 1507.34（11.11-3554.17） | 326.17（5.04-765.05） | 1.64   (1.54-1.75) | 0.91（0.21-2.44） | 3.89（1.01-10.60） | 1.10（0.30-3.03） | 1.32   (1.16-1.48) | 430.09（192.24-690.42） | 1162.60（693.36-1774.91） | 282.24（168.71-424.63） | 0.13   (0.07-0.18) | 1209.14（425.24-2176.39） | 4328.61（1626.32-7386.43） | 971.10（367.07-1674.32） | 1.02   (0.87-1.16) |
| Somalia | 14767.32（7728.89-22690.99） | 39029.58（21218.88-59914.40） | 529.94（299.17-795.83） | 0.16   (0.04-0.28) | 17.71（6.38-41.58） | 52.60（17.75-140.98） | 0.71（0.25-1.92） | 0.33   (0.2-0.46) | 11.52（3.23-24.29） | 73.81（24.74-145.24） | 1.13（0.35-2.33） | 3.53   (3.41-3.66) | 941.58（-326.39-3596.99） | 5251.79（123.33-13676.43） | 59.71（1.54-153.16） | 2.78   (2.68-2.88) | 4.54（0.93-13.65） | 21.41（4.03-69.18） | 0.42（0.08-1.31） | 1.86   (1.73-2) | 11743.92（4899.31-18856.19） | 25321.80（10974.57-41438.62） | 368.41（166.02-567.92） | -0.39   (-0.52--0.25) | 2048.05（605.19-3820.85） | 8308.16（2616.57-15888.14） | 99.56（30.85-188.61） | 1.5   (1.41-1.59) |
| South Africa | 186390.04（119188.57-258764.11） | 520149.69（326298.03-730327.89） | 1108.29（700.75-1554.81） | 0.98   (0.56-1.4) | 1208.86（629.67-2042.41） | 2627.84（1381.59-4433.63） | 5.51（2.89-9.25） | -0.72   (-1.04--0.4) | 1299.71（537.66-2185.24） | 5809.88（2481.67-9939.34） | 14.72（6.33-25.11） | 2.42   (2.19-2.64) | 34540.35（1580.06-72007.53） | 100889.87（4019.59-208682.82） | 206.18（9.76-428.11） | 1.2   (0.8-1.6) | 830.48（191.06-2196.25） | 4044.70（1019.65-10130.32） | 8.86（2.28-22.21） | 2.7   (2.47-2.93) | 93259.97（74129.55-118417.10） | 259860.16（206910.04-321624.27） | 569.58（422.84-712.96） | 1.02   (0.56-1.48) | 55250.67（22441.00-87702.53） | 146917.24（62697.16-236506.05） | 303.44（127.65-492.07） | 0.69   (0.33-1.06) |
| South Sudan | 6690.45（3836.75-10331.65） | 12319.32（7369.11-18835.09） | 284.49（171.71-428.59） | 0.15   (-0.21-0.51) | 18.03（7.12-37.30） | 31.83（12.12-70.69） | 0.74（0.29-1.64） | 0.05   (-0.24-0.34) | 1.96（-0.72-7.08） | 14.63（2.29-36.73） | 0.37（0.04-0.91） | 5.65   (5.37-5.94) | -165.64（-682.34-615.09） | 606.90（-313.75-2387.58） | 13.59（-4.89-49.60） | NA | 6.03（1.55-15.67） | 15.76（3.70-48.17） | 0.50（0.12-1.43） | 1.92   (1.7-2.13) | 5644.87（2582.77-9093.06） | 8255.85（3545.82-12986.13） | 197.97（88.18-301.63） | -0.5   (-0.88--0.12) | 1185.21（345.23-2105.32） | 3394.35（1029.86-6123.71） | 71.31（21.50-129.06） | 1.51   (1.29-1.73) |
| Spain | 198971.62（81407.42-335444.51） | 237324.69（123664.68-380669.93） | 235.42（119.76-379.59） | -1.77   (-1.89--1.65) | 2320.51（1248.50-4030.90） | 4006.32（2050.42-7024.92） | 4.40（2.27-7.75） | -0.72   (-1.1--0.34) | 5868.77（2359.32-10687.62） | 18188.03（7321.34-32506.31） | 15.88（6.49-28.41） | 1.16   (0.96-1.36) | 43518.28（3736.77-93235.61） | 35357.63（2650.38-77750.91） | 38.26（2.43-82.20） | -2.64   (-2.77--2.52) | 2995.24（795.59-7307.95） | 5524.67（1615.91-12952.83） | 4.89（1.46-11.21） | -0.98   (-1.2--0.75) | 21385.44（12827.10-29589.77） | 62243.02（25397.50-92010.90） | 49.46（25.76-69.01） | 0.81   (0.67-0.95) | 122883.38（47570.59-203995.29） | 112005.02（44432.26-186587.16） | 122.53（48.91-200.18） | -2.48   (-2.65--2.31) |
| Sri Lanka | 42051.27（27858.62-58356.46） | 99223.48（48548.18-167344.74） | 369.97（180.19-627.74） | 0.01   (-0.17-0.18) | 17.42（9.16-29.06） | 79.12（36.03-149.24） | 0.30（0.14-0.56） | 2.41   (2.23-2.59) | 119.16（39.99-234.03） | 1130.14（425.52-2120.90） | 4.53（1.67-8.83） | 4.53   (4.38-4.68) | 4761.08（476.30-10674.56） | 16660.53（1318.36-36320.16） | 62.94（4.99-136.77） | 1.5   (1.28-1.72) | 26.67（6.34-80.65） | 184.63（41.64-500.63） | 0.67（0.15-1.82） | 3.13   (2.94-3.33) | 20226.16（14713.88-26262.61） | 35027.66（20667.08-59216.94） | 130.50（77.75-221.27） | -1.33   (-1.55--1.11) | 16900.78（5873.38-27457.75） | 46141.40（17525.42-85678.98） | 171.03（65.46-317.06） | 0.77   (0.54-1) |
| Sudan | 169136.83（92641.52-266719.45） | 401602.50（198854.87-646722.40） | 1825.53（926.16-2852.79） | 0.13   (0.07-0.2) | 17.70（5.59-46.08） | 215.19（86.63-423.29） | 0.90（0.37-1.70） | 6.23   (5.96-6.49) | 293.09（111.78-523.68） | 1498.94（594.00-2600.99） | 9.32（3.59-15.90） | 3.01   (2.91-3.11) | 22423.48（1532.94-48535.92） | 59151.97（4409.38-125798.69） | 246.75（21.10-525.74） | 0.28   (0.21-0.34) | 42.03（8.40-117.90） | 171.83（39.99-426.53） | 1.01（0.23-2.56） | 2.35   (2.23-2.48) | 57712.39（28530.59-88331.93） | 114245.08（63211.51-172714.73） | 581.26（333.71-885.15） | -0.17   (-0.31--0.04) | 88648.15（34124.29-155958.93） | 226319.49（90801.74-396720.25） | 986.29（393.13-1743.59） | 0.27   (0.21-0.34) |
| Suriname | 1756.06（1012.44-2642.21） | 4270.17（2240.29-6804.72） | 653.34（344.70-1034.39） | 0.07   (-0.16-0.29) | 5.58（2.86-9.78） | 17.20（8.34-31.62） | 2.67（1.29-4.96） | 0.55   (0.37-0.74) | 6.28（2.06-11.58） | 44.03（15.63-81.58） | 7.12（2.48-13.26） | 3.48   (3.37-3.6) | 211.56（13.21-490.73） | 785.43（36.08-1703.56） | 119.45（5.76-259.08） | 1.23   (0.95-1.52) | 1.87（0.56-4.70） | 8.62（2.45-20.27） | 1.38（0.39-3.25） | 2.01   (1.92-2.11) | 629.06（483.25-798.14） | 1546.19（1101.59-2100.35） | 240.71（171.15-326.63） | 0.23   (0.06-0.41) | 901.70（353.38-1492.42） | 1868.71（721.39-3126.88） | 282.00（108.23-472.06） | -0.51   (-0.81--0.21) |
| Sweden | 65325.73（24524.16-109744.87） | 47622.50（24797.94-77625.31） | 210.81（105.86-345.63） | -2.25   (-2.32--2.17) | 1687.28（872.67-2891.63） | 1256.21（636.41-2213.64） | 5.65（2.90-9.82） | -2.81   (-3.12--2.5) | 1351.75（514.38-2416.27） | 4882.49（1906.27-9042.37） | 19.49（7.62-36.40） | 2.78   (2.62-2.94) | 7705.33（726.50-16642.30） | 5594.98（491.22-12018.03） | 28.07（2.17-59.89） | -2.24   (-2.37--2.1) | 962.12（236.61-2685.53） | 818.52（213.14-1925.55） | 3.22（0.84-7.62） | -1.78   (-1.93--1.63) | 2270.51（1275.75-3279.44） | 10978.35（4594.12-16746.15） | 41.57（19.93-60.42） | 4.99   (4.42-5.56) | 51348.73（18982.71-84460.68） | 24091.95（9592.40-41215.32） | 112.81（45.43-191.22） | -3.58   (-3.63--3.53) |
| Switzerland | 39577.29（19051.59-63660.35） | 32158.77（18269.28-49597.06） | 158.97（88.46-239.40） | -2.81   (-2.86--2.76) | 657.16（355.75-1151.40） | 521.52（263.44-937.10） | 2.81（1.45-4.97） | -2.75   (-2.85--2.65) | 490.76（199.07-838.18） | 1196.86（470.53-2095.63） | 5.58（2.18-9.75） | 1.2   (0.96-1.44) | 4330.42（409.14-8909.04） | 3056.09（280.94-6629.27） | 17.07（1.40-36.43） | -2.89   (-3.02--2.76) | 508.81（135.86-1355.85） | 585.82（156.89-1392.04） | 2.76（0.76-6.57） | -1.77   (-1.92--1.63) | 5984.98（3325.73-8748.29） | 12448.90（5145.71-19790.96） | 54.32（26.10-81.52） | 0.46   (0.12-0.79) | 27605.16（10600.01-45336.36） | 14349.58（5381.25-24158.02） | 76.43（29.13-126.37） | -4.25   (-4.33--4.18) |
| Syrian Arab Republic | 106016.95（51731.77-165860.96） | 309527.87（142431.99-510285.38） | 2336.31（1101.92-3804.96） | 0.37   (0.15-0.58) | 38.68（17.08-73.01） | 196.88（96.53-349.46） | 1.40（0.69-2.49） | 2.32   (2.19-2.45) | 291.01（117.78-534.10） | 1733.81（740.57-3027.07） | 17.30（7.27-30.38） | 2.74   (2.6-2.89) | 13204.01（946.92-28388.35） | 38223.66（2298.87-77595.51） | 277.08（18.08-567.37） | 0.3   (0.01-0.58) | 38.72（8.24-102.28） | 197.95（51.12-433.65） | 1.60（0.40-3.54） | 2.09   (2.03-2.16) | 23820.91（16051.42-31946.62） | 50017.99（33643.52-69782.72） | 429.61（262.54-614.01） | -0.77   (-1.1--0.45) | 68623.61（26366.40-113619.00） | 219157.58（93123.14-365185.81） | 1609.32（674.11-2693.00） | 0.73   (0.57-0.9) |
| Taiwan (Province of China) | 41328.34（26918.25-60507.24） | 96540.53（58525.75-141967.76） | 237.87（141.54-351.16） | 0.26   (-0.15-0.67) | 225.00（123.70-369.86） | 1442.93（780.95-2536.52） | 3.67（1.98-6.49） | 2.3   (1.6-3) | 241.36（80.30-501.61） | 2616.83（1020.59-4470.36） | 5.91（2.31-10.04） | 3.62   (3.24-4) | 7681.95（595.50-18385.17） | 17282.32（1234.82-37723.40） | 45.32（3.17-98.32） | -0.16   (-0.29--0.02) | 117.24（26.75-324.08） | 580.53（133.22-1407.08） | 1.31（0.30-3.15） | 1.54   (1.33-1.75) | 20115.70（15276.72-24972.09） | 41594.08（27875.70-54339.82） | 99.27（70.22-127.78） | 0.35   (-0.52-1.23) | 12947.09（4742.93-20668.79） | 33023.83（12744.66-54324.40） | 82.39（31.92-135.59） | 0.24   (0.02-0.46) |
| Tajikistan | 39315.82（20146.03-60124.97） | 69596.57（34035.47-113377.79） | 1197.59（601.97-1911.38） | -0.93   (-1.26--0.61) | 12.88（6.48-23.41） | 32.38（16.39-59.71） | 0.50（0.26-0.91） | 0.23   (0.03-0.43) | 113.24（43.35-200.35） | 280.24（109.32-513.11） | 5.76（2.34-10.21） | 0.6   (0.47-0.74) | 6171.50（397.52-12941.69） | 12287.85（810.22-27882.39） | 192.87（14.05-434.76） | -0.79   (-1.11--0.46) | 32.46（6.62-80.32） | 76.00（17.87-179.94） | 1.58（0.38-3.80） | 0.59   (0.48-0.71) | 11726.46（7766.96-17201.86） | 18501.21（12149.82-26285.52） | 340.78（219.36-484.00） | -1.07   (-1.43--0.72) | 21259.28（8324.78-34854.33） | 38418.89（15403.95-64675.38） | 656.09（257.45-1100.33） | -0.91   (-1.22--0.59) |
| Thailand | 55251.15（24076.31-92614.94） | 226066.59（84886.25-398163.70） | 221.41（81.14-391.13） | 1.01   (0.68-1.33) | 280.56（143.03-485.64） | 1824.47（866.52-3154.06） | 1.77（0.84-3.07） | 2.34   (2.18-2.51) | 272.73（69.43-574.98） | 5401.64（2155.81-9723.90） | 4.92（1.96-8.90） | 5.98   (5.7-6.26) | 12280.15（161.51-31568.94） | 73400.23（5155.31-156140.99） | 73.23（4.96-157.25） | 2.48   (2.06-2.91) | 90.10（21.89-243.05） | 719.04（162.19-1829.29） | 0.64（0.14-1.64） | 2.76   (2.72-2.8) | 10252.72（6760.52-14175.74） | 33579.53（24021.17-46614.71） | 31.81（22.91-44.35） | 0.12   (-0.06-0.29) | 32074.88（10593.97-52277.22） | 111141.68（41785.28-192609.23） | 109.04（41.32-188.56） | 0.39   (0.02-0.76) |
| Timor-Leste | 468.91（238.72-703.10） | 2357.34（1477.18-3505.40） | 261.50（165.11-385.74） | 2.19   (1.85-2.54) | 0.39（0.15-0.83） | 2.54（1.18-4.67） | 0.29（0.13-0.53） | 2.75   (2.5-2.99) | -0.11（-0.21-0.01） | 2.35（0.25-5.40） | 0.25（0.00-0.59） | NA | -57.27（-135.41-33.59） | 159.48（-47.23-534.55） | 17.18（-4.61-57.93） | #VALUE! | 0.17（0.05-0.46） | 1.30（0.37-3.32） | 0.16（0.05-0.40） | 1.97   (1.88-2.07) | 355.75（156.23-572.57） | 1257.28（778.95-1911.46） | 141.89（88.63-212.25） | 0.87   (0.58-1.16) | 169.99（45.01-300.15） | 934.40（287.61-1712.51） | 101.72（31.40-182.81） | 3.08   (2.71-3.46) |
| Togo | 7014.16（4290.39-10360.13） | 33551.73（19519.59-50591.16） | 791.37（459.69-1179.28） | 1.38   (1.23-1.54) | 24.11（9.99-49.44） | 114.81（42.64-254.43） | 2.94（1.08-6.55） | 1.07   (0.94-1.21) | 15.97（6.08-30.05） | 134.16（51.43-237.45） | 4.95（1.88-8.93） | 3.67   (3.64-3.71) | 1175.45（76.03-2790.18） | 6771.62（374.49-14212.59） | 149.13（9.08-313.16） | 1.97   (1.9-2.03) | 9.32（1.85-29.19） | 79.93（14.79-233.81） | 2.71（0.49-8.20） | 3.26   (3.21-3.31) | 3861.45（2360.65-5619.10） | 16525.81（8557.23-25165.71） | 395.40（211.24-602.52） | 1.15   (0.96-1.34) | 1927.86（691.76-3127.36） | 9925.40（3358.04-17099.84） | 236.24（80.43-411.10） | 1.43   (1.25-1.6) |
| Tokelau | 22.63（10.91-37.52） | 25.94（11.70-42.17） | 1804.05（812.65-2931.30） | 0.06   (0-0.11) | 0.07（0.03-0.13） | 0.12（0.06-0.20） | 8.01（3.94-13.79） | 1.11   (0.96-1.25) | 0.15（0.06-0.30） | 0.33（0.14-0.59） | 22.41（9.52-40.33） | 2.07   (1.96-2.18) | 4.81（0.02-10.86） | 5.09（0.01-10.69） | 358.38（-0.22-751.96） | -0.26   (-0.33--0.2) | 0.03（0.01-0.06） | 0.06（0.02-0.12） | 3.98（1.25-8.15） | 2.33   (2.14-2.52) | 6.08（4.00-8.51） | 4.70（3.13-6.60） | 322.83（214.51-453.87） | -1.32   (-1.36--1.28) | 11.49（4.60-19.95） | 15.64（6.76-25.76） | 1088.44（472.03-1794.79） | 0.67   (0.59-0.76) |
| Tonga | 631.79（251.41-1003.90） | 1045.43（462.27-1668.01） | 1252.85（556.72-2002.15） | 0.71   (0.62-0.8) | 3.31（1.68-5.86） | 7.67（3.60-13.59） | 9.38（4.40-16.70） | 1.56   (1.4-1.73) | 7.43（3.19-12.57） | 19.08（8.27-32.29） | 24.35（10.45-41.44） | 1.99   (1.9-2.09) | 137.85（1.07-280.78） | 207.25（2.00-404.46） | 246.33（3.00-480.90） | 0.41   (0.35-0.48) | 1.26（0.35-3.04） | 3.25（0.97-6.75） | 4.08（1.21-8.52） | 1.78   (1.58-1.98) | 73.92（52.75-98.80） | 100.05（71.35-133.31） | 122.06（87.26-162.51） | -0.15   (-0.29--0.01) | 408.01（174.28-640.90） | 708.15（329.79-1120.06） | 846.66（392.75-1340.44） | 0.9   (0.81-1) |
| Trinidad and Tobago | 9983.83（5797.15-14285.28） | 15767.16（8402.33-24628.42） | 832.89（442.27-1297.09） | -1.68   (-1.96--1.4) | 64.47（34.91-110.04） | 166.11（80.35-288.83） | 8.51（4.12-14.83） | -0.38   (-0.77-0.02) | 67.32（29.85-111.85） | 282.99（118.00-505.82） | 14.89（6.26-26.54） | 1.5   (1.43-1.57) | 1354.01（83.61-2772.45） | 2381.54（113.40-4860.59） | 127.21（5.78-257.41） | -1.22   (-1.5--0.93) | 23.76（7.24-59.34） | 90.34（23.80-205.41） | 4.63（1.22-10.66） | 1.11   (0.95-1.27) | 3274.76（2671.63-3899.27） | 4816.52（3580.32-6373.02） | 256.31（189.83-340.45） | -1.68   (-1.95--1.41) | 5199.52（2103.22-8169.36） | 8029.65（3223.58-13281.92） | 421.33（169.06-695.01） | -1.91   (-2.23--1.59) |
| Tunisia | 42774.16（25647.55-64219.49） | 138298.27（69753.19-218988.68） | 1054.52（542.91-1643.13） | 0.55   (0.49-0.62) | 10.83（5.16-20.54） | 122.81（56.29-229.22） | 0.91（0.42-1.68） | 5.45   (5.16-5.73) | 117.32（44.02-213.89） | 1195.14（478.86-2093.00） | 10.29（4.10-18.52） | 4.52   (4.36-4.68) | 4949.55（451.26-11408.13） | 18159.90（1766.29-39287.22） | 135.43（13.48-292.11） | 1.03   (0.93-1.12) | 19.42（4.06-51.26） | 139.83（33.53-334.69） | 1.08（0.26-2.58） | 3.39   (3.3-3.48) | 16379.95（10974.06-22966.04） | 48994.87（24831.43-87022.07） | 385.35（194.06-684.24） | 0.43   (0.37-0.48) | 21297.09（8126.87-35436.42） | 69685.72（27128.39-120338.46） | 521.45（203.87-895.61） | 0.48   (0.36-0.61) |
| Turkey | 402708.19（204490.99-619381.25） | 817601.53（425992.26-1265241.32） | 889.47（467.15-1373.77） | -0.98   (-1.26--0.7) | 1563.74（692.60-3000.85） | 6160.13（3214.24-11127.11） | 6.35（3.32-11.49） | 1.27   (1.13-1.42) | 1888.28（769.76-3363.68） | 9315.45（4012.93-16305.19） | 11.07（4.71-19.19） | 1.67   (1.26-2.08) | 69446.73（4563.32-150012.86） | 127548.94（9129.48-258298.19） | 135.62（10.11-274.44） | -1.36   (-1.62--1.09) | 984.13（232.33-2687.88） | 5009.96（1555.72-9945.09） | 5.45（1.67-10.98） | 2.02   (1.85-2.18) | 107445.73（65406.27-152464.01） | 209593.22（132729.49-288110.67） | 241.80（145.20-339.30） | -1.06   (-1.43--0.69) | 221379.58（86567.55-355298.13） | 459973.82（185950.63-745754.54） | 489.18（197.31-798.37） | -0.91   (-1.15--0.66) |
| Turkmenistan | 29006.44（11827.41-47646.06） | 64577.70（26479.37-110926.08） | 1543.50（649.84-2657.80） | -0.6   (-0.98--0.22) | 42.03（22.09-74.71） | 198.46（94.67-366.45） | 4.51（2.16-8.31） | 2.55   (2.38-2.71) | 91.11（34.68-163.90） | 300.92（116.06-560.86） | 8.33（3.16-15.23） | 1.29   (1.18-1.4) | 4615.45（364.37-9859.39） | 13974.64（961.65-29860.65） | 310.68（22.56-670.06） | 0.91   (0.46-1.36) | 19.04（3.90-52.88） | 76.76（18.61-192.25） | 2.04（0.50-5.08） | 2.03   (1.94-2.12) | 3779.65（2609.96-5031.01） | 10119.92（6742.70-14958.15） | 249.52（162.64-374.88） | 0.5   (0.25-0.74) | 20459.17（8090.57-33308.82） | 39907.01（15740.45-68006.11） | 968.41（377.54-1646.71） | -1.23   (-1.68--0.78) |
| Tuvalu | 170.37（78.13-274.56） | 279.22（125.71-447.77） | 2513.18（1148.39-3998.23） | 0.46   (0.41-0.51) | 0.32（0.13-0.65） | 0.80（0.38-1.49） | 7.46（3.62-13.95） | 1.51   (1.41-1.6) | 0.78（0.31-1.44） | 2.10（0.89-3.68） | 21.12（9.04-37.35） | 2.3   (2.28-2.31) | 39.12（-0.22-88.12） | 57.68（-0.37-122.75） | 507.37（-2.35-1077.11） | 0.14   (0.07-0.22) | 0.07（0.02-0.20） | 0.24（0.06-0.58） | 2.25（0.60-5.56） | 2.41   (2.25-2.57) | 43.22（24.71-60.45） | 47.04（32.27-62.70） | 437.90（297.01-591.49） | -0.99   (-1.06--0.92) | 86.86（34.12-139.49） | 171.36（75.20-270.34） | 1537.08（669.51-2438.20） | 1.11   (1.06-1.16) |
| Uganda | 17785.88（9043.41-26877.96） | 59157.09（34937.87-89352.05） | 359.07（220.37-543.34） | 0.39   (0.14-0.65) | 58.16（23.08-128.93） | 219.86（92.46-445.78） | 1.31（0.56-2.69） | 0.79   (0.59-1) | 23.95（4.93-55.80） | 221.35（80.77-427.10） | 1.53（0.53-3.13） | 4.79   (4.72-4.86) | 1058.97（-493.28-3970.55） | 8280.05（527.67-19563.63） | 45.28（3.20-105.11） | 2.78   (2.34-3.21) | 15.91（3.75-49.52） | 107.41（26.72-308.82） | 0.82（0.21-2.28） | 3.36   (3.3-3.41) | 12567.09（4003.94-21083.10） | 32105.92（13247.89-51545.17） | 208.77（83.95-334.95） | -0.23   (-0.45--0.01) | 4061.81（1403.23-7155.59） | 18222.49（6597.94-31081.38） | 101.36（36.69-173.83） | 1.08   (0.65-1.51) |
| Ukraine | 794050.07（293964.15-1339069.84） | 1125772.61（441253.66-1923874.59） | 1480.13（579.11-2525.22） | 0.27   (-0.22-0.76) | 3495.86（1886.56-5918.02） | 5528.86（2689.74-9822.77） | 7.69（3.73-13.69） | 1.06   (0.72-1.41) | 6914.50（2860.59-11422.10） | 12019.23（5073.49-20789.46） | 15.05（6.35-26.14） | 1.1   (0.96-1.24) | 176250.23（18718.35-352343.28） | 158744.41（15133.99-322466.44） | 215.78（19.31-434.63） | -1.1   (-1.4--0.8) | 5953.22（1288.28-15006.21） | 11329.80（2821.36-27850.80） | 13.98（3.47-34.33） | 1.44   (1.29-1.59) | 52563.99（41992.13-63286.02） | 70685.47（48194.30-97015.91） | 94.41（64.64-128.57） | 0.52   (0.26-0.79) | 548872.26（211889.49-896506.27） | 867464.85（341324.99-1463023.37） | 1133.23（447.52-1914.89） | 0.54   (-0.03-1.11) |
| United Arab Emirates | 7951.43（3695.41-13013.57） | 53050.02（26536.86-83992.33） | 1245.66（668.10-1883.86） | 0.91   (0.48-1.34) | 21.23（9.36-40.66） | 234.67（120.10-426.87） | 4.26（2.21-7.32） | 2.97   (2.43-3.51) | 20.70（8.22-38.19） | 437.04（199.71-738.90） | 17.91（8.18-30.72） | 5.29   (4.67-5.91) | 1339.49（91.68-2893.97） | 10956.27（902.99-21017.48） | 195.21（19.17-380.14） | 1.06   (0.7-1.43) | 15.47（3.76-43.53） | 273.82（91.47-482.37） | 10.58（3.53-19.59） | 5.62   (4.74-6.51) | 1666.38（1160.24-2430.87） | 11290.29（8289.49-15157.51） | 369.47（251.20-491.63） | 1.57   (1.02-2.13) | 4888.18（1847.96-7956.25） | 29857.92（13724.22-47159.62） | 648.22（274.33-1067.10） | 0.41   (0.03-0.8) |
| United Kingdom | 612891.75（249791.07-1024613.82） | 345714.18（162131.86-563423.22） | 283.73（132.68-458.42） | -3.24   (-3.41--3.07) | 17550.31（9285.27-29436.52） | 8545.63（4445.86-15024.96） | 6.40（3.36-11.19） | -4.3   (-4.64--3.95) | 8715.65（3438.17-16301.56） | 21278.20（8803.11-39200.21） | 14.77（6.11-27.19） | 1.5   (1.41-1.59) | 75930.26（7013.26-159803.23） | 48273.43（3205.70-100856.15） | 40.79（2.24-85.59） | -2.7   (-2.85--2.55) | 6345.96（1605.69-17806.47） | 7410.13（2038.13-19035.23） | 5.12（1.41-13.12） | -1.29   (-1.52--1.07) | 30389.44（22953.98-37608.41） | 47225.54（33275.57-59586.70） | 38.24（29.47-46.21） | 0.86   (0.7-1.02) | 473960.14（186164.99-780721.85） | 212981.25（84038.05-347915.94） | 178.40（71.25-286.79） | -4.03   (-4.22--3.84) |
| United Republic of Tanzania | 51248.10（30913.70-71438.38） | 176521.69（102754.37-272941.22） | 664.87（397.81-1009.90） | 1.12   (1.06-1.17) | 159.91（70.80-309.46） | 806.12（327.65-1677.50） | 2.93（1.22-6.12） | 2.06   (1.95-2.17) | 135.64（45.71-272.63） | 1155.58（470.29-2052.20） | 5.26（2.14-9.39） | 4.33   (4.23-4.43) | 4930.30（110.20-12578.83） | 27267.31（1536.07-61103.15） | 96.21（5.80-214.17） | 2.54   (2.34-2.74) | 54.26（9.79-160.18） | 430.44（66.42-1326.94） | 1.97（0.32-5.98） | 3.8   (3.74-3.87) | 36186.15（16351.50-52874.47） | 87628.08（38263.54-131385.98） | 353.84（153.42-540.51） | 0.13   (0.07-0.18) | 9781.84（3332.13-16792.96） | 59234.15（21222.64-106220.02） | 204.66（74.47-371.64） | 2.85   (2.69-3.02) |
| United States of America | 2246060.40（1054158.83-3598683.88） | 3415390.31（1933350.14-5036824.26） | 641.98（368.97-936.04） | -0.61   (-0.71--0.5) | 29678.97（15724.73-50741.68） | 27119.12（14469.06-45713.11） | 5.19（2.79-8.71） | -2.63   (-2.94--2.32) | 38132.81（14850.00-71113.86） | 152715.79（64969.83-258787.45） | 24.74（10.59-41.79） | 2.39   (2.22-2.55) | 219432.13（14408.32-461195.87） | 397340.88（25616.07-780580.14） | 77.60（4.45-151.21） | -0.1   (-0.26-0.07) | 29161.87（8505.82-63699.05） | 68163.46（21498.28-130942.27） | 11.24（3.57-21.46） | 0.04   (-0.26-0.35) | 311355.17（248534.13-373660.51） | 957209.81（758537.18-1146594.93） | 187.98（155.90-219.82） | 2.34   (2.2-2.48) | 1618299.44（642107.51-2651881.46） | 1812841.25（762868.62-2832221.04） | 335.24（143.10-518.55） | -1.82   (-1.95--1.69) |
| United States Virgin Islands | 1035.50（552.52-1601.26） | 1120.90（606.31-1700.50） | 707.63（383.18-1073.23） | -1.48   (-1.63--1.33) | 4.57（2.30-8.01） | 7.10（3.42-12.41） | 4.15（2.00-7.26） | -0.98   (-1.18--0.77) | 11.38（4.78-20.99） | 30.50（12.75-54.91） | 17.27（7.14-30.81） | 0.13   (0.01-0.24) | 125.92（3.43-264.57） | 116.02（5.77-244.75） | 79.34（2.79-167.94） | -1.54   (-1.7--1.38) | 4.57（1.31-11.64） | 14.68（4.37-30.60） | 8.30（2.41-17.11） | 1.01   (0.86-1.17) | 297.05（229.90-386.45） | 336.10（228.73-466.83） | 207.53（142.96-286.96） | -1.32   (-1.47--1.17) | 592.01（240.64-960.06） | 616.49（262.37-1015.61） | 391.03（168.19-645.19） | -1.64   (-1.8--1.48) |
| Uruguay | 24769.79（10635.48-41138.94） | 23522.39（12728.42-36535.06） | 442.11（230.62-694.73） | -1.43   (-1.55--1.32) | 387.94（205.46-659.48） | 490.39（255.78-824.28） | 9.49（5.01-15.98） | -0.59   (-0.83--0.35) | 232.12（93.49-399.97） | 618.40（239.91-1080.50） | 9.97（3.94-17.55） | 1.68   (1.57-1.79) | 5635.58（363.63-12150.25） | 4725.06（297.51-9922.95） | 94.16（5.07-199.32） | -1.91   (-2.09--1.74) | 44.22（8.87-123.85） | 77.67（18.09-200.59） | 1.33（0.31-3.42） | 0.55   (0.46-0.64) | 3451.19（2559.05-4321.88） | 6577.03（3978.14-8854.53） | 110.70（76.54-141.88） | 0.67   (0.52-0.83) | 15018.75（5929.95-24663.58） | 11033.84（4397.18-18007.79） | 216.47（87.09-349.92） | -2.08   (-2.25--1.92) |
| Uzbekistan | 139424.09（57202.14-225800.75） | 383998.93（175948.06-622368.01） | 1446.11（663.45-2330.05） | 0.5   (0.15-0.84) | 44.59（22.06-82.42） | 590.54（310.48-1099.42） | 2.15（1.12-3.99） | 6.48   (5.75-7.22) | 432.98（169.16-780.10） | 1566.99（604.44-2923.59） | 6.87（2.63-12.59） | 1.77   (1.69-1.85) | 25586.98（1798.84-54512.97） | 56579.04（4485.77-119247.65） | 197.07（16.53-419.58） | -0.77   (-1.1--0.45) | 55.26（10.14-161.08） | 221.40（54.40-552.91） | 0.97（0.24-2.54） | 2.02   (1.82-2.21) | 19395.98（13075.14-27192.65） | 63157.89（43476.35-88022.32） | 241.96（167.97-334.49） | 1.38   (1.13-1.63) | 93908.29（36327.15-153510.61） | 261883.08（104132.15-435014.93） | 997.08（393.06-1665.49） | 0.59   (0.19-0.99) |
| Vanuatu | 988.30（447.06-1786.83） | 3790.83（1611.82-6282.11） | 1752.67（752.69-2921.84） | 0.94   (0.86-1.01) | 2.01（0.88-4.08） | 8.50（3.93-15.23） | 4.35（2.00-7.96） | 1.04   (0.92-1.15) | 2.36（0.82-5.15） | 15.39（6.17-28.37） | 8.61（3.35-16.03） | 2.88   (2.81-2.96) | 148.59（6.46-382.50） | 650.26（10.67-1477.76） | 286.70（6.30-656.38） | 1.31   (1.21-1.41) | 0.64（0.16-1.67） | 3.10（0.80-7.75） | 1.76（0.48-4.38） | 1.32   (1.19-1.46) | 214.05（128.51-325.05） | 655.91（454.78-889.16） | 325.67（220.35-440.92） | 0.19   (0.12-0.27) | 620.66（222.62-1121.17） | 2457.66（1007.95-4147.88） | 1125.59（461.53-1907.96） | 1.08   (1.01-1.16) |
| Venezuela (Bolivarian Republic of) | 100485.81（57105.84-151834.50） | 292393.18（148908.70-474206.29） | 963.61（497.58-1548.76） | -0.54   (-0.83--0.24) | 445.06（241.35-768.05） | 1372.23（635.33-2453.13） | 4.58（2.13-8.20） | -0.59   (-0.88--0.29) | 807.32（309.11-1535.65） | 4686.44（2004.34-8451.70） | 16.40（6.93-29.51） | 1.76   (1.69-1.83) | 11826.98（426.09-25988.47） | 36683.62（938.38-79185.06） | 120.18（3.38-260.57） | -0.2   (-0.48-0.08) | 175.01（42.80-421.29） | 599.37（162.54-1402.29） | 2.05（0.55-4.82） | -0.21   (-0.4--0.01) | 32345.03（26223.47-38694.40） | 66934.89（47228.05-94252.27） | 228.02（157.99-323.83） | -1.65   (-2--1.29) | 54886.42（22045.84-90394.97） | 182116.64（73151.35-309691.71） | 592.38（236.94-1011.32） | -0.1   (-0.38-0.18) |
| Viet Nam | 40844.79（26506.63-62431.26） | 191843.54（114354.22-293664.46） | 186.83（112.80-281.46） | 2.61   (2.26-2.97) | 58.06（27.26-110.46） | 463.88（231.21-806.36） | 0.47（0.23-0.83） | 4.43   (4.23-4.63) | 2.77（-32.87-45.33） | 872.87（246.46-1790.12） | 0.94（0.25-2.06） | 18.46   (16.78-20.16) | -3528.33（-11129.71-4461.98） | 33674.84（837.39-86815.58） | 31.50（1.33-78.36） | NA | 38.01（11.02-95.53） | 227.84（59.69-586.70） | 0.24（0.06-0.62） | 3.13   (3-3.26) | 35016.98（20915.61-56528.60） | 100161.37（56888.20-161293.68） | 100.48（57.12-158.84） | 0.83   (0.56-1.09) | 9257.30（2741.69-15899.56） | 56442.73（18784.06-94259.45） | 53.19（17.86-88.74） | 3.75   (3.35-4.14) |
| Yemen | 60714.57（35353.06-92306.82） | 239450.88（127466.06-375054.06） | 1567.00（858.82-2422.85） | 0.83   (0.67-0.99) | 5.26（1.77-11.39） | 93.48（38.29-182.59） | 0.55（0.23-1.06） | 6.6   (6.26-6.94) | 58.72（19.53-122.88） | 677.97（268.83-1171.73） | 6.24（2.50-11.30） | 4.92   (4.67-5.17) | 6274.32（553.52-13691.68） | 33682.91（2347.69-70159.98） | 202.62（15.31-410.86） | 1.78   (1.62-1.93) | 12.85（2.50-35.22） | 89.91（18.93-234.65） | 0.75（0.16-1.95） | 2.89   (2.75-3.04) | 27134.12（13112.49-42123.90） | 89228.43（45625.35-148663.58） | 643.38（336.39-1081.30） | 0.32   (0.16-0.48) | 27229.30（9244.78-47951.90） | 115678.18（39400.06-193667.47） | 713.46（244.29-1207.35） | 1.08   (0.92-1.24) |
| Zambia | 16027.16（10965.40-21952.38） | 65580.65（34557.19-101268.97） | 857.92（507.91-1283.78） | 1.36   (1.22-1.49) | 33.35（14.66-64.22） | 312.52（98.71-731.30） | 3.79（1.24-8.62） | 4.22   (3.69-4.75) | 16.35（3.58-37.01） | 331.34（125.31-657.56） | 5.26（1.95-10.16） | 7.15   (7.03-7.26) | 926.95（-230.86-3250.58） | 11851.31（519.22-26101.62） | 139.15（6.67-313.55） | 4.73   (4.38-5.08) | 17.82（4.93-51.00） | 227.11（42.33-667.99） | 3.60（0.70-10.03） | 5.5   (5.14-5.86) | 12898.15（7525.10-17939.59） | 36650.07（18575.56-53881.02） | 512.88（267.16-749.03） | 0.31   (0.17-0.45) | 2134.55（685.61-3613.42） | 16208.30（6364.15-28697.73） | 193.24（74.08-344.82） | 3.42   (3.29-3.55) |
| Zimbabwe | 16483.39（11200.77-22484.05） | 78472.14（45835.79-119937.01） | 1062.83（635.79-1622.03） | 4.03   (3.34-4.72) | 91.34（48.05-153.84） | 463.69（208.14-921.88） | 6.40（2.95-12.65） | 3.58   (3.22-3.94) | 60.22（22.15-104.73） | 413.53（171.95-714.63） | 6.93（2.76-12.24） | 5.14   (4.75-5.52) | 2089.17（176.73-4643.43） | 16150.93（490.36-35003.48） | 205.59（6.94-445.41） | 5.9   (4.98-6.83) | 10.06（2.29-29.78） | 42.70（8.91-120.61） | 0.69（0.15-1.93） | 3.09   (3.03-3.16) | 10573.13（7520.09-14322.73） | 38533.74（24148.04-57152.60） | 540.18（338.44-796.76） | 3.13   (2.53-3.73) | 3659.49（1365.51-6187.28） | 22867.55（8542.96-39377.30） | 303.04（111.23-514.80） | 4.91   (4.11-5.73) |

CVD, cardiovascular disease; HBMI, high body mass index; EAPC, estimated annual percentage change; DALYs, disability-adjusted life years; ASDR, age-standardized DALYs (disability-adjusted life years) rate.

Table S5. Deaths of CVD attributable to HBMI in 1990 and 2021, and EAPC of ASMR from 1990 to 2021 in 204 countries and territories

|  | CVD | | | | Aortic aneurysm | | | | Atrial fibrillation and flutter | | | | Stroke | | | | Lower extremity peripheral arterial disease | | | | Hypertensive heart disease | | | | Ischemic heart disease | | | |
| --- | --- | --- | --- | --- | --- | --- | --- | --- | --- | --- | --- | --- | --- | --- | --- | --- | --- | --- | --- | --- | --- | --- | --- | --- | --- | --- | --- | --- |
| location | Death number in 1990 | Death number in 2021 | ASMR in 2021 (per 100,000) | EAPC, 1990–2021 | Death number in 1990 | Death number in 2021 | ASMR in 2021 (per 100,000) | EAPC, 1990–2021 | Death number in 1990 | Death number in 2021 | ASMR in 2021 (per 100,000) | EAPC, 1990–2021 | Death number in 1990 | Death number in 2021 | ASMR in 2021 (per 100,000) | EAPC, 1990–2021 | Death number in 1990 | Death number in 2021 | ASMR in 2021 (per 100,000) | EAPC, 1990–2021 | Death number in 1990 | Death number in 2021 | ASMR in 2021 (per 100,000) | EAPC, 1990–2021 | Death number in 1990 | Death number in 2021 | ASMR in 2021 (per 100,000) | EAPC, 1990–2021 |
| Afghanistan | 4921.83（2610.63-7585.75） | 7287.01（4030.39-11249.33） | 77.40（43.01-117.53） | 0.01  (-0.09-0.12) | 0.27（0.11-0.57） | 1.94（0.86-3.85） | 0.02（0.01-0.04） | 5.91   (5.61-6.2) | 3.90（1.17-8.71） | 11.90（4.27-22.92） | 0.19（0.06-0.37） | 3.48  (3.3-3.67) | 517.62（37.54-1109.60） | 945.95（67.25-2049.91） | 8.89（0.75-18.99） | 0.42   (0.25-0.59) | 0.01（0.00-0.02） | 0.20（0.04-0.55） | 0.00（0.00-0.01） | 12.17   (11.32-13.03) | 2338.50（867.80-3961.77） | 2995.13（1427.50-4803.28） | 36.60（17.84-58.05） | -0.12   (-0.24-0) | 2061.53（764.51-3578.21） | 3331.87（1298.56-5872.57） | 31.70（12.00-55.55） | 0.05   (-0.06-0.16) |
| Albania | 626.19（285.33-1051.70） | 1433.36（613.82-2514.30） | 34.67（14.73-60.86） | 0.27   (0.07-0.47) | 1.47（0.76-2.63） | 5.03（2.29-9.49） | 0.12（0.05-0.22） | 1.36   (1.22-1.5) | 4.90（1.88-9.40） | 21.50（8.23-42.07） | 0.56（0.22-1.10） | 2.05   (1.84-2.26) | 128.82（5.84-300.44） | 275.56（9.22-665.41） | 6.54（0.22-15.77） | -0.1   (-0.32-0.11) | 0.53（0.12-1.34） | 2.88（0.66-6.65） | 0.07（0.02-0.17） | 2.76   (2.5-3.02) | 124.54（71.20-188.80） | 236.57（105.54-398.09） | 5.79（2.45-9.91） | -0.42   (-0.66--0.18) | 365.94（136.98-604.61） | 891.82（332.20-1523.92） | 21.59（8.04-37.07） | 0.56   (0.36-0.75) |
| Algeria | 5034.71（3193.81-7591.91） | 17417.10（9851.02-25853.33） | 63.56（35.44-96.75） | 0.66   (0.59-0.72) | 0.92（0.44-1.67） | 11.77（5.65-21.01） | 0.04（0.02-0.07） | 5.68   (5.34-6.02) | 12.13（4.21-25.05） | 143.57（56.32-263.71） | 0.72（0.29-1.30） | 5.17   (4.84-5.51) | 506.63（46.81-1164.96） | 2008.47（215.61-4330.09） | 6.75（0.79-14.45） | 1.07   (1.03-1.11) | 0.06（0.01-0.18） | 2.96（0.78-6.76） | 0.01（0.00-0.03） | 10.94   (10.12-11.78) | 2237.43（1375.08-3318.47） | 7083.97（3893.75-10625.74） | 27.42（12.35-42.80） | 0.57   (0.42-0.73) | 2277.55（818.86-3888.01） | 8166.37（3274.77-13887.03） | 28.62（11.27-48.16） | 0.59   (0.51-0.66) |
| American Samoa | 12.79（5.55-19.71） | 29.54（13.63-46.59） | 62.14（28.74-98.01） | 0.44   (0.35-0.53) | 0.07（0.04-0.12） | 0.16（0.08-0.26） | 0.35（0.18-0.58） | -0.3   (-0.51--0.09) | 0.09（0.03-0.16） | 0.36（0.15-0.64） | 1.03（0.42-1.83） | 2.28   (2.21-2.35) | 2.84（-0.02-5.84） | 5.43（-0.04-10.71） | 11.15（0.01-21.93） | -0.19   (-0.34--0.05) | 0.04（0.01-0.09） | 0.11（0.04-0.21） | 0.29（0.09-0.56） | 0.36   (-0.33-1.05) | 2.32（1.66-2.92） | 4.01（3.03-5.13） | 8.89（6.26-11.59） | -0.98   (-1.25--0.71) | 7.42（3.33-11.45） | 19.47（9.00-30.52） | 40.44（18.28-64.30） | 1.03   (0.97-1.09) |
| Andorra | 7.72（3.78-13.04） | 17.77（9.18-30.74） | 10.06（5.32-17.46） | -1.16   (-1.38--0.95) | 0.27（0.12-0.51） | 0.50（0.23-0.96） | 0.31（0.14-0.60） | -1.36   (-1.59--1.12) | 0.10（0.04-0.20） | 0.54（0.20-1.11） | 0.29（0.11-0.59） | 0.77   (0.6-0.94) | 0.75（0.07-1.75） | 1.56（0.15-3.52） | 0.92（0.08-2.06） | -1.3   (-1.53--1.06) | 0.12（0.03-0.33） | 0.40（0.09-0.98） | 0.22（0.05-0.53） | -0.54   (-0.75--0.33) | 2.18（1.02-3.60） | 6.76（2.29-11.93） | 3.59（1.35-6.25） | -0.74   (-0.92--0.55) | 4.30（1.52-7.65） | 8.01（3.00-14.73） | 4.74（1.76-8.79） | -1.54   (-1.78--1.29) |
| Angola | 843.49（529.78-1224.66） | 3370.41（2089.52-4921.13） | 33.68（21.70-49.60） | 0.75   (0.62-0.87) | 2.28（1.03-4.47） | 13.23（6.31-24.83） | 0.13（0.06-0.24） | 1.89   (1.77-2.02) | 0.39（0.07-1.07） | 8.30（2.94-16.67） | 0.11（0.03-0.24） | 7.33   (7.2-7.46) | 29.06（-10.25-101.96） | 338.16（21.82-789.77） | 2.86（0.22-6.47） | 3.88   (3.69-4.08) | 0.78（0.24-2.43） | 8.24（2.06-21.48） | 0.10（0.02-0.25） | 4.08   (3.87-4.29) | 649.82（339.62-967.28） | 2099.34（1288.38-3056.63） | 22.51（13.29-35.02） | 0.19   (0.08-0.31) | 161.15（50.39-278.74） | 903.14（317.67-1575.14） | 7.98（2.75-14.13） | 1.83   (1.71-1.96) |
| Antigua and Barbuda | 17.36（11.26-25.29） | 37.41（26.17-50.65） | 38.71（25.36-53.21） | 0.68   (0.36-1.01) | 0.09（0.05-0.16） | 0.16（0.08-0.26） | 0.15（0.08-0.26） | -0.65   (-0.93--0.37) | 0.13（0.04-0.27） | 0.47（0.18-0.88） | 0.58（0.23-1.10） | 3.21   (2.96-3.46) | 2.10（0.15-4.66） | 4.03（0.20-8.54） | 3.92（0.22-8.36） | -0.26   (-0.48--0.05) | 0.08（0.02-0.19） | 0.23（0.07-0.55） | 0.25（0.07-0.62） | 2.14   (1.9-2.38) | 8.86（5.52-12.26） | 23.39（16.30-30.31） | 24.66（15.05-33.16） | 1.76   (1.31-2.22) | 6.11（2.35-10.07） | 9.14（3.63-14.80） | 9.14（3.55-14.93） | -1.07   (-1.28--0.86) |
| Argentina | 10299.72（5358.07-16395.45） | 12567.04（7183.34-18785.30） | 21.91（12.52-32.76） | -1.02   (-1.15--0.9) | 107.25（56.43-186.00） | 157.80（79.72-277.54） | 0.28（0.14-0.49） | -0.57   (-0.8--0.34) | 68.41（27.77-129.64） | 253.61（102.94-475.33） | 0.43（0.17-0.80） | 2.4   (2-2.8) | 1736.98（90.54-3844.36） | 1630.92（82.19-3459.81） | 2.91（0.14-6.13） | -1.94   (-2.1--1.79) | 21.78（5.84-53.13） | 63.50（18.84-143.40） | 0.11（0.03-0.24） | 1.45   (1.05-1.86) | 2700.78（1778.56-3528.97） | 4862.98（2451.57-6898.62） | 8.31（4.25-11.72） | 0.16   (-0.02-0.34) | 5664.53（2220.36-9451.30） | 5598.23（2199.66-9148.49） | 9.88（3.90-16.10） | -1.66   (-1.81--1.52) |
| Armenia | 1285.04（606.57-2051.86） | 1840.39（915.62-2917.76） | 42.83（21.23-67.74） | -1.22   (-1.45--1) | 10.09（5.23-17.51） | 38.72（20.04-66.53） | 0.89（0.46-1.53） | 3.07   (2.75-3.38) | 2.93（1.25-5.14） | 12.34（5.25-21.66） | 0.29（0.12-0.51） | 2.68   (2.33-3.04) | 165.66（13.67-354.28） | 191.01（22.53-397.69） | 4.44（0.52-9.22） | -2.29   (-2.68--1.9) | 5.96（1.37-16.56） | 16.39（3.98-42.17） | 0.38（0.09-0.99） | 1.56   (1.2-1.92) | 221.12（136.06-309.01） | 356.80（187.39-514.65） | 8.28（4.51-11.85） | -0.27   (-0.63-0.1) | 879.29（338.23-1461.36） | 1225.12（476.90-2034.77） | 28.55（11.14-47.54） | -1.46   (-1.68--1.23) |
| Australia | 4595.23（1878.73-7853.40） | 5582.05（2643.03-9273.90） | 11.05（5.14-18.26） | -2.7   (-2.81--2.6) | 111.22（58.45-199.25） | 109.76（56.09-190.94） | 0.22（0.12-0.39） | -3.61   (-3.79--3.42) | 76.05（29.51-149.57） | 482.72（196.80-897.22） | 0.87（0.35-1.61） | 2.27   (1.96-2.58) | 474.02（44.73-993.24） | 665.78（53.41-1453.87） | 1.33（0.09-2.88） | -2.29   (-2.38--2.19) | 111.35（27.96-270.66） | 271.58（79.38-548.18） | 0.49（0.15-0.98） | -0.97   (-1.1--0.84) | 279.27（154.05-404.51） | 728.21（284.90-1068.73） | 1.36（0.62-1.95） | -0.1   (-0.51-0.31) | 3543.30（1356.19-5909.21） | 3324.00（1330.65-5557.20） | 6.78（2.78-11.16） | -3.49   (-3.59--3.38) |
| Austria | 3133.02（1491.60-5201.15） | 3506.03（1952.68-5551.36） | 15.66（8.84-24.96） | -1.63   (-1.77--1.49) | 30.07（15.96-52.02） | 30.84（15.36-53.33） | 0.16（0.08-0.27） | -1.73   (-1.92--1.54) | 44.14（17.79-82.35） | 150.06（63.97-290.91） | 0.62（0.26-1.19） | 1.78   (1.43-2.13) | 424.44（47.00-912.53） | 202.65（18.34-461.55） | 0.96（0.08-2.17） | -4.53   (-4.88--4.19) | 46.06（8.91-138.93） | 73.83（16.69-187.76） | 0.32（0.07-0.79） | 0.04   (-0.78-0.86) | 566.80（277.32-826.77） | 1372.13（431.07-2209.96） | 5.65（2.05-8.88） | 1.59   (1.22-1.97) | 2021.52（741.96-3406.02） | 1676.52（642.96-2936.24） | 7.95（3.07-13.75） | -2.82   (-3.02--2.62) |
| Azerbaijan | 2804.18（1355.76-4489.40） | 5267.96（2453.61-8486.67） | 60.05（28.53-95.94） | -0.05   (-0.28-0.18) | 2.73（1.40-4.78） | 14.13（6.24-27.43） | 0.14（0.07-0.27） | 3.58   (3.28-3.89) | 4.27（1.75-7.80） | 14.92（5.77-26.48） | 0.21（0.08-0.37） | 2.26   (2.04-2.47) | 287.32（13.61-649.22） | 551.83（21.45-1263.66） | 5.81（0.26-13.42） | -0.1   (-0.42-0.23) | 1.10（0.20-3.15） | 5.44（1.09-16.38） | 0.07（0.01-0.18） | 3.5   (3.23-3.77) | 587.31（379.72-838.69） | 959.00（610.56-1433.67） | 11.47（6.75-17.63） | -0.21   (-0.44-0.02) | 1921.44（737.20-3240.97） | 3722.65（1527.24-6331.57） | 42.36（16.86-71.10） | -0.01   (-0.24-0.22) |
| Bahamas | 73.39（52.62-100.93） | 212.87（148.57-290.19） | 55.22（37.31-76.19） | 0.55   (0.39-0.71) | 0.37（0.19-0.66） | 1.05（0.52-1.89） | 0.28（0.14-0.50） | 0.16   (-0.19-0.52) | 0.37（0.15-0.68） | 2.16（0.86-4.11） | 0.67（0.26-1.29） | 2.64   (2.34-2.94) | 6.73（0.31-14.77） | 17.14（0.59-36.13） | 4.22（0.18-8.91） | -0.06   (-0.14-0.03) | 0.30（0.08-0.72） | 1.35（0.41-3.30） | 0.39（0.12-0.96） | 1.9   (1.72-2.07) | 41.78（31.49-51.90） | 140.81（100.63-184.58） | 37.10（24.71-50.31） | 1.12   (0.87-1.38) | 23.83（9.41-38.85） | 50.35（20.25-83.21） | 12.57（5.00-21.13） | -0.72   (-0.9--0.54) |
| Bahrain | 100.60（49.42-159.41） | 312.83（160.33-476.99） | 51.28（28.00-78.10） | -1.62   (-1.92--1.33) | 0.08（0.04-0.14） | 0.69（0.35-1.18） | 0.08（0.04-0.15） | 2.03   (1.63-2.43) | 0.30（0.10-0.62） | 2.71（0.98-5.18） | 0.96（0.30-1.94） | 2.37   (1.92-2.82) | 10.71（0.74-22.51） | 43.20（3.56-87.91） | 6.43（0.70-13.51） | -1   (-1.4--0.59) | 0.06（0.02-0.16） | 2.22（0.70-4.17） | 0.44（0.14-0.86） | 9.68   (8.24-11.13) | 19.17（13.73-25.10） | 68.62（45.14-97.86） | 15.32（7.41-23.37） | -0.8   (-1.09--0.51) | 70.28（28.64-115.34） | 195.40（79.58-309.99） | 28.05（11.11-45.81） | -2.28   (-2.61--1.94) |
| Bangladesh | 2481.06（1633.34-3544.05） | 13717.95（7248.92-21908.65） | 10.49（5.69-17.11） | 2.43   (2.28-2.58) | 4.04（1.54-8.60） | 36.26（15.71-66.78） | 0.03（0.01-0.05） | 3.77   (3.56-3.98) | 0.23（-0.96-1.62） | 36.07（8.44-92.45） | 0.03（0.01-0.09） | 15.37   (14.14-16.61) | -51.76（-316.22-348.93） | 1958.17（146.75-4646.79） | 1.42（0.12-3.37） | NA | 1.15（0.22-3.72） | 16.01（4.32-42.53） | 0.01（0.00-0.04） | 5.32   (5.12-5.53) | 1406.96（807.30-2288.87） | 5593.56（2923.86-10799.38） | 4.68（2.25-9.36） | 1.21   (0.97-1.45) | 1120.44（343.81-1863.20） | 6077.87（2115.84-10554.77） | 4.31（1.49-7.42） | 2.72   (2.54-2.91) |
| Barbados | 77.20（42.19-123.16） | 134.26（73.72-208.99） | 26.05（14.34-40.57） | 0   (-0.14-0.14) | 0.52（0.28-0.89） | 1.14（0.56-2.03） | 0.21（0.11-0.38） | 0.18   (-0.14-0.51) | 0.76（0.26-1.46） | 3.29（1.37-6.27） | 0.64（0.27-1.22） | 2.94   (2.64-3.24) | 13.32（1.13-28.93） | 24.80（1.82-54.00） | 4.86（0.34-10.56） | -0.08   (-0.24-0.08) | 2.54（0.79-5.56） | 6.85（2.12-14.78） | 1.33（0.41-2.88） | 1.24   (0.94-1.53) | 25.92（15.75-35.94） | 50.07（28.83-71.45） | 9.71（5.54-13.91） | 0.91   (0.75-1.07) | 34.13（12.80-57.72） | 48.12（19.03-82.32） | 9.29（3.68-15.92） | -1.03   (-1.31--0.75) |
| Belarus | 5935.49（2189.06-10245.38） | 10283.40（3685.32-17907.74） | 63.39（22.72-110.94） | 0.38   (-0.05-0.82) | 28.96（15.34-50.97） | 71.63（35.29-127.08） | 0.45（0.22-0.80） | 1.87   (1.48-2.27) | 33.40（13.61-61.68） | 91.14（40.04-164.92） | 0.55（0.24-0.99） | 1.83   (1.68-1.98) | 989.58（111.80-2033.55） | 1273.40（136.31-2680.71） | 7.88（0.82-16.71） | -0.78   (-1.29--0.26) | 35.93（7.86-104.55） | 114.40（25.31-330.11） | 0.68（0.15-1.98） | 2.58   (2.34-2.81) | 328.59（233.84-438.20） | 108.61（72.90-144.79） | 0.69（0.48-0.91） | -5.33   (-6.45--4.2) | 4519.03（1720.96-7574.44） | 8624.23（3275.57-14596.91） | 53.14（20.18-90.54） | 0.74   (0.33-1.16) |
| Belgium | 2463.61（980.63-4136.65） | 2080.10（986.72-3486.12） | 7.54（3.41-12.72） | -2.47   (-2.65--2.29) | 46.08（23.89-77.47） | 48.41（24.71-84.54） | 0.19（0.10-0.32） | -1.71   (-1.91--1.52) | 35.64（15.22-65.32） | 131.79（52.49-233.77） | 0.40（0.16-0.71） | 2.1   (1.75-2.45) | 387.87（42.59-800.17） | 315.84（27.09-686.10） | 1.18（0.09-2.53） | -2.43   (-2.49--2.36) | 20.14（4.68-51.78） | 48.37（12.19-119.46） | 0.16（0.04-0.38） | 0.54   (-0.1-1.19) | 173.91（79.64-270.71） | 338.55（114.82-546.84） | 1.05（0.44-1.64） | -0.36   (-0.95-0.23) | 1799.98（664.62-2976.93） | 1197.15（449.92-2035.94） | 4.57（1.73-7.62） | -3.14   (-3.31--2.97) |
| Belize | 27.03（15.41-40.97） | 92.08（59.35-129.18） | 32.49（20.71-45.47） | 0.2   (-0.11-0.51) | 0.07（0.03-0.12） | 0.24（0.12-0.40） | 0.09（0.04-0.15） | -0.01   (-0.73-0.71) | 0.25（0.09-0.55） | 1.56（0.66-2.73） | 0.65（0.27-1.14） | 2.58   (1.94-3.22) | 3.60（0.13-7.83） | 12.57（0.41-25.49） | 4.18（0.18-8.54） | -0.14   (-0.66-0.38) | 0.03（0.01-0.08） | 0.22（0.06-0.52） | 0.08（0.02-0.20） | 2.89   (2.15-3.64) | 9.08（6.42-11.66） | 44.68（31.63-57.32） | 16.13（10.59-21.24） | 2.11   (1.84-2.38) | 14.01（5.44-23.40） | 32.81（13.85-53.01） | 11.36（4.73-18.59） | -1.49   (-1.85--1.13) |
| Benin | 330.82（194.19-490.98） | 1104.51（631.93-1685.00） | 22.55（12.86-34.76） | 0.89   (0.8-0.98) | 1.06（0.44-2.15） | 4.03（1.42-8.58） | 0.09（0.03-0.18） | 1.17   (1.01-1.34) | 0.66（0.22-1.44） | 4.07（1.43-7.65） | 0.12（0.04-0.25） | 3.59   (3.41-3.77) | 79.97（4.79-178.72） | 260.37（12.52-576.75） | 5.06（0.29-11.11） | 0.72   (0.57-0.88) | 0.38（0.07-1.22） | 2.65（0.52-8.06） | 0.07（0.01-0.20） | 3.63   (3.52-3.75) | 170.84（98.44-242.71） | 516.09（293.73-733.12） | 10.78（6.06-15.67） | 0.61   (0.5-0.73) | 77.92（26.31-128.36） | 317.30（116.46-553.91） | 6.44（2.30-11.17） | 1.55   (1.34-1.76) |
| Bermuda | 23.60（10.66-39.19） | 31.02（16.03-50.00） | 21.19（11.09-34.08） | -2.01   (-2.37--1.66) | 0.50（0.27-0.86） | 0.64（0.31-1.14） | 0.43（0.21-0.76） | -2.3   (-2.45--2.15) | 0.20（0.07-0.41） | 0.95（0.38-1.80） | 0.58（0.23-1.09） | 0.97   (0.88-1.07) | 2.26（0.19-4.98） | 3.39（0.33-7.08） | 2.39（0.21-4.92） | -1.67   (-1.82--1.52) | 0.44（0.12-1.03） | 1.41（0.41-2.90） | 0.87（0.26-1.80） | 0.32   (0.06-0.58) | 2.96（1.98-3.88） | 7.93（4.25-11.22） | 5.26（3.00-7.30） | 0.47   (-0.08-1.02) | 17.25（6.64-28.73） | 16.69（6.65-28.39） | 11.66（4.70-19.73） | -2.95   (-3.3--2.6) |
| Bhutan | 38.74（19.99-64.45） | 111.92（60.41-183.85） | 18.85（10.45-30.81） | 0.5   (0.46-0.55) | 0.06（0.03-0.12） | 0.43（0.20-0.78） | 0.07（0.03-0.13） | 3.36   (3.29-3.44) | 0.04（0.01-0.09） | 0.49（0.16-0.98） | 0.09（0.03-0.19） | 5.42   (5.28-5.55) | 4.77（0.33-12.03） | 13.79（0.93-34.31） | 2.26（0.16-5.64） | 0.63   (0.56-0.7) | 0.01（0.00-0.03） | 0.09（0.02-0.28） | 0.02（0.00-0.05） | 4.28   (4.1-4.46) | 15.13（7.07-23.57） | 37.93（22.36-58.03） | 6.78（3.89-10.56） | -0.28   (-0.34--0.22) | 18.73（6.51-32.98） | 59.19（22.28-104.85） | 9.63（3.56-17.04） | 1.11   (1.07-1.14) |
| Bolivia (Plurinational State of) | 659.49（345.09-1035.40） | 1756.14（896.99-2878.75） | 21.11（11.02-34.13） | -0.11   (-0.23-0.02) | 1.51（0.64-2.86） | 7.77（3.72-14.18） | 0.09（0.04-0.17） | 2.06   (2.01-2.11) | 2.52（0.84-5.20） | 26.15（10.41-47.53） | 0.38（0.15-0.69） | 4.65   (4.49-4.81) | 115.41（6.47-269.33） | 320.89（11.71-722.98） | 3.49（0.16-7.91） | -0.06   (-0.15-0.04) | 0.14（0.03-0.36） | 1.35（0.34-3.76） | 0.02（0.00-0.05） | 4.4   (4.27-4.54) | 205.05（84.34-321.16） | 562.87（278.21-865.12） | 7.32（3.35-11.52） | -0.03   (-0.07-0.01) | 334.87（122.05-595.98） | 837.11（299.37-1495.80） | 9.82（3.44-17.53） | -0.3   (-0.51--0.08) |
| Bosnia and Herzegovina | 1348.10（600.24-2195.76） | 2146.68（1025.39-3528.71） | 33.94（16.19-55.69） | -0.54   (-0.73--0.35) | 6.65（3.21-12.57） | 19.24（8.43-35.64） | 0.30（0.13-0.56） | 2.02   (1.87-2.18) | 6.17（2.56-10.99） | 30.59（13.44-52.72） | 0.48（0.21-0.83） | 2.77   (2.6-2.94) | 286.42（28.90-614.26） | 439.31（54.31-925.46） | 6.88（0.84-14.47） | -0.8   (-0.97--0.63) | 1.15（0.24-3.18） | 5.39（1.42-12.11） | 0.08（0.02-0.19） | 2.95   (2.76-3.13) | 234.19（154.12-338.30） | 492.84（246.63-775.25） | 7.73（3.94-12.11） | 0.69   (0.51-0.87) | 813.51（316.80-1340.99） | 1159.30（425.28-1958.16） | 18.46（6.79-31.09） | -0.97   (-1.23--0.71) |
| Botswana | 157.42（99.58-239.67） | 495.19（314.42-704.52） | 41.74（25.88-60.31） | 1.03   (0.71-1.35) | 0.45（0.20-0.89） | 2.08（0.97-3.86） | 0.18（0.08-0.32） | 1.82   (1.64-2) | 0.33（0.13-0.69） | 3.28（1.30-5.85） | 0.41（0.16-0.74） | 5.55   (5.09-6.01) | 20.87（1.38-50.87） | 74.20（3.52-158.79） | 5.85（0.35-12.55） | 1.59   (1.17-2.02) | 0.26（0.07-0.75） | 2.55（0.64-6.41） | 0.23（0.05-0.57） | 4.83   (4.59-5.07) | 106.24（67.97-157.11） | 288.55（195.98-406.06） | 25.32（14.94-36.56） | 0.61   (0.3-0.93) | 29.27（10.00-52.04） | 124.53（45.78-208.18） | 9.76（3.50-16.20） | 1.82   (1.5-2.14) |
| Brazil | 25484.56（14003.69-39112.19） | 53384.28（30139.73-82272.21） | 21.54（12.20-33.06） | -1.05   (-1.1--0.99) | 194.41（105.77-325.17） | 953.85（495.98-1673.38） | 0.38（0.20-0.67） | 1.47   (1.18-1.75) | 112.09（41.99-200.98） | 1238.46（497.29-2150.52） | 0.53（0.21-0.92） | 3.43   (3.17-3.69) | 4908.20（324.27-10686.02） | 8725.89（518.04-18801.49） | 3.49（0.21-7.50） | -1.58   (-1.64--1.52) | 120.81（32.66-320.74） | 714.15（197.93-1619.85） | 0.30（0.08-0.67） | 1.6   (1.4-1.81) | 8247.89（6241.63-10305.36） | 16585.67（10636.69-21884.77） | 6.83（4.26-9.14） | -1.2   (-1.35--1.05) | 11901.17（4619.18-19320.47） | 25166.26（9806.75-41034.53） | 10.01（3.88-16.35） | -0.99   (-1.04--0.94) |
| Brunei Darussalam | 15.35（8.53-23.01） | 51.71（24.74-77.88） | 14.49（7.73-21.72） | 0.3   (0.12-0.47) | 0.17（0.08-0.30） | 0.80（0.43-1.38） | 0.23（0.12-0.41） | 1.27   (1.1-1.43) | 0.03（0.01-0.06） | 0.53（0.21-0.86） | 0.15（0.06-0.26） | 5.72   (5.41-6.02) | 2.03（0.14-4.80） | 8.93（0.23-17.99） | 2.17（0.10-4.44） | 0.79   (0.66-0.92) | 0.02（0.01-0.07） | 0.12（0.03-0.26） | 0.05（0.02-0.11） | 1.99   (1.78-2.2) | 4.68（2.98-6.62） | 12.03（8.50-16.87） | 4.58（2.53-6.96） | -0.12   (-0.34-0.11) | 8.41（3.06-13.43） | 29.30（11.52-46.79） | 7.31（2.79-11.79） | 0.36   (0.19-0.53) |
| Bulgaria | 8551.11（4337.09-13568.62） | 14847.47（9561.06-21083.60） | 105.44（67.21-150.34） | 0.7   (0.42-0.99) | 21.79（11.30-38.90） | 40.66（20.35-72.81） | 0.29（0.15-0.52） | 1.18   (0.87-1.49) | 42.92（17.45-73.13） | 119.44（50.79-211.35） | 0.82（0.35-1.44） | 1.22   (1.06-1.38) | 1719.33（125.83-3638.95） | 1898.57（219.73-4023.05） | 13.29（1.49-28.38） | -0.81   (-0.97--0.65) | 11.33（3.09-24.79） | 28.87（7.92-66.95） | 0.20（0.05-0.47） | 0.73   (0.03-1.43) | 2157.41（1605.99-2775.90） | 8511.24（4944.69-11903.85） | 59.71（34.53-83.60） | 4.28   (3.49-5.07) | 4598.33（1741.12-7372.48） | 4248.70（1675.25-7304.35） | 31.13（12.42-53.47） | -2.15   (-2.48--1.81) |
| Burkina Faso | 392.29（268.22-555.10） | 1268.81（815.65-1871.83） | 14.44（9.29-21.24） | 1.58   (1.49-1.67) | 1.26（0.50-2.78） | 4.42（1.71-9.47） | 0.05（0.02-0.12） | 1.8   (1.73-1.87) | 0.09（-0.05-0.34） | 1.24（0.10-3.53） | 0.02（0.00-0.06） | 7.76   (7.65-7.87) | 11.78（-9.66-57.44） | 83.05（3.21-230.06） | 0.86（0.05-2.39） | 3.9   (3.82-3.98) | 0.34（0.05-1.22） | 2.01（0.35-7.19） | 0.03（0.00-0.10） | 3.47   (3.33-3.61) | 288.42（167.12-431.01） | 869.17（455.90-1391.34） | 10.01（5.18-16.63） | 1.38   (1.28-1.48) | 90.40（29.54-154.42） | 308.92（103.21-555.94） | 3.46（1.13-6.18） | 1.75   (1.68-1.82) |
| Burundi | 283.65（100.13-452.12） | 566.53（370.38-817.12） | 13.54（8.31-20.32） | -0.6   (-0.82--0.37) | 0.86（0.33-1.79） | 1.46（0.58-3.20） | 0.04（0.01-0.08） | -0.92   (-1.26--0.58) | 0.07（-0.02-0.29） | 0.55（0.10-1.57） | 0.02（0.00-0.06） | 4.7   (4.38-5.02) | 5.28（-18.72-47.86） | 41.70（-1.85-112.66） | 0.93（0.04-2.31） | 2.31   (1.95-2.67) | 0.24（0.05-0.69） | 0.68（0.16-2.08） | 0.02（0.00-0.06） | 1.31   (0.87-1.74) | 218.25（17.56-379.02） | 377.67（178.71-567.39） | 9.53（4.43-15.62） | -0.89   (-1.09--0.68) | 58.95（17.75-103.57） | 144.46（46.77-255.57） | 3.00（0.94-5.23） | -0.16   (-0.41-0.1) |
| Cabo Verde | 34.38（21.73-49.41） | 115.36（67.45-181.35） | 26.70（15.64-41.79） | 1.51   (1.22-1.8) | 0.17（0.05-0.39） | 0.70（0.27-1.54） | 0.16（0.06-0.36） | 2.34   (2.09-2.58) | 0.12（0.03-0.28） | 1.38（0.49-2.65） | 0.33（0.12-0.63） | 6.24   (5.94-6.55) | 3.88（0.40-8.82） | 18.56（1.59-40.06） | 4.19（0.38-9.01） | 2.58   (2.3-2.86) | 0.11（0.01-0.34） | 0.92（0.23-2.59） | 0.22（0.06-0.62） | 5.05   (4.81-5.29) | 21.58（12.84-32.28） | 44.95（28.09-64.39） | 10.48（6.42-15.04） | 0.09   (-0.21-0.39) | 8.53（3.09-14.62） | 48.84（18.80-85.54） | 11.31（4.35-19.76） | 3.02   (2.67-3.37) |
| Cambodia | 460.24（270.26-669.83） | 1491.31（941.96-2163.09） | 12.66（8.01-18.76） | 0.56   (0.42-0.7) | 0.32（0.12-0.63） | 1.59（0.70-3.14） | 0.01（0.01-0.03） | 2.01   (1.83-2.2) | 0.13（-0.02-0.40） | 2.00（0.43-4.83） | 0.02（0.00-0.06） | 7.66   (7.57-7.74) | 12.92（-27.05-83.78） | 161.76（4.64-389.44） | 1.28（0.06-3.03） | 4.59   (4.39-4.78) | 0.03（0.01-0.08） | 0.18（0.04-0.49） | 0.00（0.00-0.00） | 2.37   (2.21-2.52) | 318.56（148.63-502.32） | 854.28（444.04-1281.50） | 7.53（3.89-11.72） | 0   (-0.15-0.15) | 128.28（39.24-215.73） | 471.50（154.50-805.52） | 3.81（1.23-6.58） | 0.99   (0.87-1.12) |
| Cameroon | 1197.24（712.81-1770.03） | 4906.53（2558.26-8105.31） | 43.44（22.88-69.99） | 1.32   (0.99-1.65) | 4.65（2.05-9.04） | 25.23（10.97-51.48） | 0.23（0.10-0.46） | 1.93   (1.7-2.17) | 5.07（1.84-9.47） | 34.23（13.58-60.41） | 0.53（0.20-0.95） | 3.18   (3.13-3.24) | 224.13（11.25-494.09） | 1140.51（38.17-2481.47） | 9.25（0.44-20.27） | 2.04   (1.63-2.44) | 2.95（0.51-9.52） | 30.23（7.35-76.27） | 0.34（0.08-0.93） | 4.28   (4.19-4.38) | 670.23（341.21-951.67） | 2032.02（949.93-3077.61） | 18.44（8.55-28.66） | 0.27   (0.08-0.46) | 290.21（98.99-506.62） | 1644.31（609.25-3018.21） | 14.65（5.27-26.63） | 2.48   (1.91-3.05) |
| Canada | 7354.43（3009.15-12550.16） | 8709.01（4047.81-14159.08） | 11.26（5.28-18.11） | -2.57   (-2.74--2.39) | 169.25（85.97-298.78） | 152.93（78.85-268.39） | 0.20（0.10-0.35） | -3.8   (-4.09--3.51) | 121.37（54.40-226.87） | 453.15（181.95-821.95） | 0.52（0.21-0.94） | 0.57   (0.37-0.77) | 695.61（62.23-1504.34） | 904.72（73.34-1982.29） | 1.17（0.08-2.58） | -2.45   (-2.66--2.25) | 91.83（20.84-267.79） | 192.24（50.88-500.74） | 0.23（0.06-0.59） | -1.18   (-1.47--0.89) | 319.90（189.95-439.19） | 1261.56（670.60-1736.50） | 1.66（0.99-2.19） | 1.97   (1.56-2.37) | 5956.47（2324.76-10018.56） | 5744.40（2252.77-9486.68） | 7.49（2.96-12.18） | -3.21   (-3.36--3.06) |
| Central African Republic | 243.23（122.18-365.49） | 730.29（408.09-1136.97） | 37.40（21.63-57.56） | 1.38   (1.32-1.43) | 0.57（0.26-1.13） | 1.57（0.67-3.37） | 0.08（0.03-0.17） | 0.83   (0.71-0.94) | 0.16（0.03-0.45） | 1.29（0.44-2.89） | 0.09（0.03-0.21） | 5.49   (5.41-5.57) | 11.20（-2.76-37.53） | 83.63（3.59-194.64） | 3.52（0.21-8.15） | 4.19   (4.07-4.31) | 0.28（0.06-0.93） | 1.38（0.31-4.30） | 0.09（0.02-0.26） | 3.17   (3.06-3.29) | 176.51（57.66-299.33） | 445.89（169.18-750.02） | 24.91（9.44-42.94） | 0.97   (0.91-1.03) | 54.51（16.41-97.98） | 196.54（61.06-366.58） | 8.71（2.56-16.31） | 1.86   (1.8-1.92) |
| Chad | 424.68（253.98-624.36） | 1206.35（690.95-1811.40） | 22.43（12.96-34.38） | 1   (0.8-1.21) | 1.04（0.38-2.40） | 2.72（1.02-6.08） | 0.05（0.02-0.12） | 0.74   (0.63-0.85) | 0.80（0.21-1.78） | 3.04（1.02-6.31） | 0.09（0.03-0.21） | 2.69   (2.64-2.74) | 51.59（3.50-124.45） | 182.99（12.89-410.78） | 3.24（0.25-7.14） | 1.68   (1.55-1.81) | 0.64（0.10-2.26） | 2.52（0.42-9.13） | 0.06（0.01-0.23） | 2.5   (2.47-2.53) | 269.94（119.90-400.98） | 690.29（322.29-1066.31） | 12.97（5.85-20.24） | 0.68   (0.44-0.91) | 100.68（29.50-175.08） | 324.80（98.34-554.17） | 6.00（1.81-10.49） | 1.43   (1.24-1.62) |
| Chile | 2476.01（1274.74-4024.43） | 4554.06（2567.59-6903.52） | 17.47（9.88-26.51） | -1.09   (-1.2--0.98) | 23.70（12.72-41.72） | 69.83（35.73-124.42） | 0.27（0.14-0.48） | 0.08   (-0.29-0.46) | 19.75（8.16-37.84） | 132.55（53.01-238.17） | 0.50（0.20-0.89） | 3.45   (2.86-4.04) | 506.61（32.57-1099.81） | 715.76（48.45-1496.62） | 2.78（0.18-5.81） | -1.7   (-1.85--1.55) | 13.74（3.51-35.24） | 42.59（11.67-102.71） | 0.16（0.04-0.39） | 0.52   (-0.45-1.49) | 624.19（389.98-835.61） | 1817.10（824.48-2586.42） | 6.87（3.16-9.76） | 0.28   (0-0.56) | 1288.03（497.51-2130.72） | 1776.22（707.61-2861.82） | 6.89（2.74-11.08） | -2.14   (-2.23--2.05) |
| China | 88033.66（61877.55-122803.50） | 354345.48（188060.30-564964.38） | 18.80（10.07-29.43） | 1.2   (1.02-1.39) | 69.65（34.48-125.06） | 530.80（269.78-961.71） | 0.03（0.01-0.05） | 3.91   (3.73-4.08) | 51.36（9.67-116.38） | 2378.46（942.02-4211.56） | 0.15（0.06-0.27） | 8.34   (8.12-8.56) | 5222.79（-1601.74-20839.38） | 71095.43（6018.54-157250.16） | 3.47（0.30-7.53） | 5.11   (4.66-5.56) | 27.66（8.42-72.16） | 269.00（73.64-641.35） | 0.01（0.00-0.04） | 3.68   (3.56-3.79) | 58894.17（33793.57-82599.91） | 124763.89（63333.52-198270.70） | 6.90（3.14-11.62） | -1.17   (-1.58--0.75) | 23768.03（8048.79-40103.79） | 155307.90（55188.46-261864.77） | 8.24（2.91-14.00） | 3.41   (3.14-3.67) |
| Colombia | 4548.29（2852.59-6524.31） | 9800.94（5126.68-15981.11） | 17.33（9.09-28.24） | -1.94   (-2.14--1.74) | 33.23（17.80-57.22） | 157.91（79.98-272.81） | 0.29（0.15-0.49） | 0.13   (-0.25-0.5) | 24.00（9.17-43.07） | 249.85（109.43-458.91） | 0.43（0.19-0.79） | 2.59   (2.46-2.72) | 448.23（34.58-956.19） | 1020.50（61.52-2257.65） | 1.83（0.11-4.06） | -1.67   (-1.86--1.48) | 8.90（2.42-23.49） | 40.33（10.94-106.50） | 0.07（0.02-0.19） | -0.12   (-0.74-0.5) | 1863.79（1274.28-2480.42） | 2275.91（1171.65-3344.68） | 3.98（2.09-5.81） | -4.08   (-4.3--3.85) | 2170.14（822.33-3472.45） | 6056.43（2398.07-10253.36） | 10.73（4.27-18.06） | -0.83   (-1.02--0.63) |
| Comoros | 36.80（21.16-54.34） | 119.93（73.34-178.82） | 27.56（16.95-41.97） | 0.5   (0.31-0.69) | 0.11（0.04-0.24） | 0.47（0.16-1.02） | 0.11（0.04-0.23） | 1.31   (1.11-1.52) | 0.03（0.01-0.09） | 0.43（0.15-0.89） | 0.12（0.04-0.25） | 4.82   (4.64-5) | 2.25（-0.13-7.08） | 15.83（0.95-34.72） | 3.30（0.23-7.23） | 2.77   (2.59-2.95) | 0.02（0.00-0.06） | 0.17（0.02-0.47） | 0.05（0.01-0.13） | 3.11   (2.93-3.28) | 28.30（13.28-44.69） | 73.75（39.73-118.21） | 17.80（8.76-29.31） | -0.14   (-0.33-0.05) | 6.08（1.98-10.11） | 29.27（10.81-51.13） | 6.19（2.27-10.91） | 1.86   (1.7-2.01) |
| Congo | 377.33（233.53-554.34） | 1229.64（773.67-1844.91） | 51.93（33.14-76.20） | 0.69   (0.54-0.83) | 1.33（0.66-2.48） | 4.83（2.28-8.86） | 0.20（0.09-0.35） | 0.87   (0.68-1.06) | 0.41（0.13-0.88） | 5.13（1.92-9.58） | 0.30（0.10-0.60） | 6.62   (6.43-6.8) | 26.11（1.29-67.25） | 149.84（5.74-322.26） | 5.44（0.26-11.86） | 2.27   (1.92-2.61) | 0.57（0.15-1.61） | 3.85（1.03-10.09） | 0.19（0.05-0.48） | 3.29   (3.11-3.46) | 255.17（116.42-396.58） | 689.37（384.27-1033.26） | 31.50（16.46-49.46） | 0.24   (0.12-0.37) | 93.73（30.04-164.41） | 376.62（135.03-649.76） | 14.31（5.11-24.52） | 1.23   (1.06-1.41) |
| Cook Islands | 12.12（8.62-16.18） | 16.57（11.23-22.80） | 66.23（44.67-91.69） | -1.32   (-1.45--1.2) | 0.03（0.02-0.06） | 0.09（0.03-0.18） | 0.35（0.14-0.72） | 0.7   (0.64-0.77) | 0.06（0.02-0.11） | 0.20（0.08-0.36） | 0.85（0.34-1.52） | 1.26   (1.21-1.3) | 1.23（0.00-2.57） | 1.45（0.03-2.97） | 5.87（0.08-11.93） | -1.45   (-1.68--1.21) | 0.01（0.00-0.01） | 0.02（0.01-0.05） | 0.09（0.03-0.19） | 2.04   (1.74-2.34) | 7.28（5.36-9.50） | 9.19（6.30-12.20） | 36.45（24.19-48.43） | -1.78   (-1.88--1.68) | 3.51（1.44-5.57） | 5.62（2.41-9.07） | 22.62（9.72-36.59） | -0.51   (-0.66--0.36) |
| Costa Rica | 341.57（181.87-535.69） | 894.20（508.68-1372.51） | 15.83（9.02-24.28） | -1.27   (-1.44--1.1) | 2.81（1.45-4.89） | 13.25（6.67-23.09） | 0.24（0.12-0.42） | 0.62   (0.36-0.89) | 3.25（1.29-6.53） | 26.93（10.83-48.69） | 0.46（0.19-0.84） | 2.29   (2.04-2.54) | 32.72（2.20-72.01） | 95.90（6.22-208.15） | 1.72（0.11-3.73） | -0.83   (-1.17--0.48) | 1.12（0.31-2.95） | 4.45（1.23-11.01） | 0.08（0.02-0.19） | -0.61   (-1.05--0.16) | 91.00（58.65-125.47） | 285.39（146.84-410.81） | 4.99（2.63-7.12） | -1.41   (-1.85--0.96) | 210.68（80.76-355.76） | 468.28（187.43-787.46） | 8.34（3.33-14.01） | -1.42   (-1.6--1.24) |
| Coted'Ivoire | 767.32（471.21-1143.17） | 3352.71（1896.21-5183.47） | 32.43（19.26-48.88） | 1.37   (1.15-1.6) | 3.28（1.30-6.58） | 15.37（5.68-34.20） | 0.15（0.06-0.34） | 1.12   (0.97-1.28) | 1.63（0.60-3.15） | 13.32（5.08-23.95） | 0.24（0.09-0.45） | 2.89   (2.77-3.01) | 127.92（10.50-289.92） | 627.19（32.57-1355.41） | 5.48（0.35-11.61） | 1.85   (1.66-2.05) | 1.22（0.23-3.94） | 10.11（1.97-30.22） | 0.13（0.02-0.39） | 2.89   (2.76-3.01) | 385.84（209.59-551.32） | 1534.34（856.67-2217.15） | 15.38（8.21-22.68） | 1.06   (0.81-1.31) | 247.44（83.53-410.10） | 1152.38（401.59-1990.36） | 11.04（3.85-19.06） | 1.59   (1.36-1.81) |
| Croatia | 3103.29（1579.15-5024.85） | 3174.74（1616.14-5061.05） | 33.21（16.90-52.41） | -1.62   (-1.75--1.5) | 15.86（8.09-27.26） | 36.98（18.38-63.93） | 0.40（0.20-0.68） | 1.08   (0.82-1.35) | 11.47（4.69-20.99） | 49.02（20.78-84.40） | 0.48（0.20-0.82） | 2.47   (2.11-2.84) | 545.78（47.16-1159.32） | 424.36（45.73-945.52） | 4.40（0.45-9.84） | -2.87   (-3.06--2.68) | 31.59（8.62-77.16） | 86.30（25.47-185.49） | 0.88（0.26-1.87） | 0.95   (0.7-1.2) | 745.38（404.18-1080.40） | 752.42（338.87-1103.38） | 7.74（3.74-11.21） | -1.06   (-1.51--0.6) | 1753.22（670.07-2943.44） | 1825.67（706.76-3123.37） | 19.31（7.55-32.80） | -1.7   (-1.79--1.62) |
| Cuba | 2023.18（879.67-3166.83） | 5037.56（2843.54-7747.74） | 24.85（14.06-38.27） | 0.63   (0.42-0.83) | 22.64（12.23-36.42） | 49.81（25.92-85.86） | 0.25（0.13-0.43） | -0.04   (-0.24-0.17) | 12.54（4.52-22.75） | 90.34（38.26-155.63） | 0.41（0.17-0.70） | 3.6   (3.42-3.78) | 235.73（17.41-485.10） | 595.02（47.22-1284.54） | 3.03（0.23-6.44） | 0.74   (0.63-0.84) | 16.13（4.61-39.84） | 102.57（30.33-249.08） | 0.48（0.14-1.15） | 3.83   (3.63-4.04) | 243.52（183.86-314.87） | 1616.73（1002.42-2254.47） | 7.84（5.06-10.72） | 4.32   (4.12-4.53) | 1492.63（570.40-2366.83） | 2583.08（979.44-4345.46） | 12.84（4.88-21.54） | -0.73   (-0.98--0.47) |
| Cyprus | 185.95（97.92-281.44） | 329.98（182.08-530.35） | 20.38（11.48-32.26） | -1.87   (-2.06--1.67) | 2.83（1.38-4.77） | 6.61（3.27-11.62） | 0.32（0.16-0.57） | -1.25   (-1.49--1) | 2.13（0.79-4.16） | 10.60（4.25-19.44） | 0.72（0.29-1.33） | 1.38   (1.16-1.6) | 20.30（1.97-41.46） | 32.35（3.21-73.34） | 1.93（0.20-4.37） | -2.33   (-2.54--2.12) | 0.80（0.25-2.24） | 3.38（0.99-8.95） | 0.24（0.07-0.64） | 0.82   (0.65-0.99) | 50.73（20.23-93.88） | 101.41（40.94-162.12） | 7.20（2.32-11.94） | -1.45   (-1.8--1.1) | 109.17（40.62-175.78） | 175.64（68.13-303.07） | 9.96（3.81-17.14） | -2.26   (-2.42--2.1) |
| Czechia | 8246.06（2939.10-14213.97） | 6702.76（3445.04-10763.09） | 29.29（15.01-46.93） | -2.33   (-2.43--2.24) | 44.23（23.44-76.41） | 73.18（35.92-127.91） | 0.33（0.16-0.57） | 0.02   (-0.34-0.39) | 54.20（22.43-103.43） | 150.72（61.13-270.85） | 0.63（0.26-1.13） | 1.57   (1.46-1.68) | 1751.59（195.46-3641.48） | 650.34（71.85-1427.03） | 2.86（0.30-6.27） | -5.11   (-5.4--4.81) | 72.09（18.86-162.31） | 141.16（40.32-316.38） | 0.60（0.17-1.35） | -0.45   (-1.34-0.45) | 297.76（213.41-379.72） | 1302.32（688.60-1868.49） | 5.58（2.98-7.97） | 2.81   (1.95-3.69) | 6026.19（2293.96-9875.21） | 4385.03（1694.84-7418.14） | 19.29（7.46-32.56） | -2.54   (-2.65--2.43) |
| Democratic People's Republic of Korea | 1197.14（717.52-1869.80） | 6060.01（3456.25-10300.55） | 21.43（11.58-36.27） | 2.55   (2.48-2.62) | 2.07（0.94-3.87） | 6.62（3.35-12.13） | 0.02（0.01-0.04） | 1.61   (1.48-1.74) | 1.08（-0.06-3.82） | 56.83（16.00-135.16） | 0.26（0.06-0.62） | 10.21   (10.01-10.41) | -16.89（-119.84-180.85） | 783.27（78.10-2205.39） | 2.70（0.29-7.43） | 9.66   (9.1-10.22) | 0.39（0.11-1.12） | 2.68（0.69-7.88） | 0.01（0.00-0.03） | 3.58   (3.48-3.67) | 763.02（407.21-1246.65） | 2876.55（1593.05-4572.59） | 10.34（5.43-17.02） | 1.65   (1.54-1.77) | 447.47（133.67-811.90） | 2334.06（779.09-4406.36） | 8.10（2.75-15.51） | 2.86   (2.81-2.92) |
| Democratic Republic of the Congo | 2834.21（1661.25-4205.91） | 12124.87（7051.20-18147.10） | 40.86（25.03-61.85） | 2.11   (2.08-2.14) | 9.34（4.03-18.63） | 33.10（13.89-69.38） | 0.11（0.04-0.22） | 1.07   (0.71-1.44) | 3.16（0.81-7.13） | 46.91（15.36-98.73） | 0.22（0.07-0.47） | 6.43   (6.21-6.65) | 161.84（-1.24-470.92） | 1347.83（63.44-3121.05） | 4.00（0.22-8.99） | 4.03   (3.99-4.07) | 8.17（1.43-26.08） | 53.26（9.41-161.86） | 0.21（0.04-0.61） | 3.18   (2.65-3.72) | 1962.57（857.81-3121.27） | 7561.81（4059.45-11812.30） | 27.04（13.58-43.96） | 1.92   (1.85-1.99) | 689.13（214.33-1215.14） | 3081.96（1023.08-5419.83） | 9.28（3.05-16.54） | 2.01   (1.86-2.16) |
| Denmark | 1951.78（775.63-3214.03） | 1011.00（451.51-1731.13） | 7.78（3.40-13.34） | -3.89   (-4.01--3.78) | 40.63（21.67-68.00） | 48.49（24.86-85.25） | 0.37（0.19-0.64） | -1.46   (-1.79--1.13) | 20.94（8.71-35.62） | 68.13（26.16-125.19） | 0.48（0.18-0.87） | 2.31   (1.82-2.81) | 202.73（20.46-419.51） | 159.05（14.23-342.03） | 1.23（0.10-2.68） | -2.52   (-2.73--2.3) | 25.17（5.54-73.82） | 37.07（9.06-95.29） | 0.26（0.06-0.67） | -0.89   (-1.32--0.46) | 96.92（51.75-142.21） | 141.93（54.36-224.25） | 1.02（0.42-1.58） | -0.27   (-0.57-0.02) | 1565.39（594.27-2564.86） | 556.34（211.91-960.20） | 4.43（1.70-7.57） | -5.11   (-5.29--4.93) |
| Djibouti | 15.16（10.24-22.73） | 83.07（52.51-123.63） | 15.75（9.44-23.82） | 0.23   (0.16-0.31) | 0.04（0.02-0.08） | 0.30（0.12-0.61） | 0.05（0.02-0.11） | 0.91   (0.8-1.02) | 0.00（0.00-0.01） | 0.09（0.02-0.21） | 0.02（0.00-0.07） | 7.81   (7.5-8.12) | -0.18（-1.09-1.21） | 5.51（-0.06-15.07） | 0.90（0.06-2.29） | 14.27   (9.67-19.06) | 0.01（0.00-0.03） | 0.18（0.05-0.47） | 0.05（0.01-0.12） | 3.12   (3-3.25) | 13.11（7.96-19.89） | 54.91（32.80-88.55） | 11.28（5.89-19.64） | -0.45   (-0.53--0.37) | 2.17（0.64-4.14） | 22.08（6.71-38.68） | 3.45（1.04-6.08） | 2.36   (2.24-2.48) |
| Dominica | 33.19（21.51-47.56） | 44.82（28.25-63.42） | 56.65（35.46-80.16） | -0.05   (-0.23-0.13) | 0.18（0.09-0.34） | 0.35（0.18-0.60） | 0.43（0.22-0.75） | 0.87   (0.68-1.07) | 0.35（0.14-0.64） | 0.82（0.37-1.46） | 1.17（0.52-2.05） | 1.71   (1.58-1.85) | 3.96（0.24-8.17） | 6.13（0.41-12.88） | 7.57（0.53-16.18） | 0.3   (0.18-0.42) | 0.10（0.03-0.26） | 0.30（0.09-0.62） | 0.39（0.12-0.82） | 2.39   (2.29-2.5) | 17.59（11.39-23.63） | 24.05（15.43-32.77） | 30.76（18.20-42.31） | 0.14   (-0.02-0.31) | 11.02（4.32-17.84） | 13.17（5.43-21.42） | 16.34（6.73-26.78） | -0.69   (-0.95--0.43) |
| Dominican Republic | 707.40（396.95-1104.45） | 3077.69（1605.81-4951.74） | 30.75（16.22-49.39） | 1.93   (1.76-2.1) | 2.04（1.10-3.59） | 11.19（5.52-19.84） | 0.11（0.06-0.20） | 2.04   (1.85-2.23) | 1.56（0.52-3.20） | 22.10（8.12-42.86） | 0.23（0.08-0.45） | 5.08   (4.82-5.34) | 77.59（5.79-189.27） | 399.74（17.27-889.09） | 3.92（0.18-8.70） | 2.53   (2.36-2.69) | 0.56（0.14-1.70） | 5.06（1.28-12.44） | 0.05（0.01-0.13） | 3.78   (3.46-4.1) | 260.48（182.52-361.95） | 872.14（529.61-1282.13） | 8.89（5.34-13.28） | 0.94   (0.73-1.15) | 365.17（130.25-608.62） | 1767.47（668.73-2968.12） | 17.55（6.63-29.46） | 2.39   (2.22-2.56) |
| Ecuador | 1101.27（671.31-1647.46） | 3387.54（1723.44-5414.13） | 22.33（11.78-35.43） | 0.65   (0.28-1.01) | 3.28（1.69-5.65） | 17.99（8.97-32.44） | 0.11（0.06-0.20） | 2.45   (2.03-2.87) | 5.98（2.03-11.72） | 64.54（25.27-123.19） | 0.46（0.18-0.88） | 4.1   (3.87-4.33) | 166.96（8.99-370.17） | 468.06（16.11-1021.81） | 2.90（0.11-6.27） | -0.06   (-0.31-0.19) | 0.13（0.03-0.31） | 6.39（1.72-13.75） | 0.04（0.01-0.10） | 10.57   (9.7-11.44) | 478.05（322.58-651.53） | 1050.61（555.77-1592.81） | 7.32（3.52-11.22） | 0.41   (-0.44-1.26) | 446.88（175.18-739.98） | 1779.94（679.08-2998.96） | 11.50（4.37-19.61） | 0.81   (0.28-1.35) |
| Egypt | 25431.16（15001.45-37174.95） | 69444.37（36811.32-104589.06） | 136.89（75.82-203.74） | 0.84   (0.71-0.98) | 6.19（2.89-11.45） | 34.45（17.47-60.20） | 0.06（0.03-0.10） | 2.94   (2.86-3.03) | 50.32（20.30-90.22） | 208.66（93.04-351.15） | 0.73（0.32-1.24） | 1.91   (1.78-2.05) | 3620.95（326.59-7735.64） | 10102.58（1237.59-19729.65） | 19.03（2.49-38.03） | 0.96   (0.83-1.1) | 2.12（0.43-5.81） | 11.89（3.50-25.81） | 0.03（0.01-0.06） | 2.45   (2.33-2.58) | 9907.91（6764.53-14098.15） | 18826.63（13393.95-25036.16） | 42.72（25.00-60.52） | -0.33   (-0.49--0.17) | 11843.67（4748.68-18945.88） | 40260.16（17302.93-64049.82） | 74.32（30.79-119.96） | 1.68   (1.48-1.88) |
| El Salvador | 621.59（280.43-1022.24） | 1437.70（664.12-2383.52） | 21.99（10.06-36.35） | 0.04   (-0.2-0.28) | 1.10（0.59-1.91） | 3.52（1.73-6.26） | 0.06（0.03-0.10） | 1.1   (0.95-1.26) | 8.15（3.05-15.13） | 49.50（20.92-93.28） | 0.67（0.28-1.26） | 2.61   (2.49-2.74) | 93.90（4.13-211.37） | 160.80（7.47-350.74） | 2.54（0.11-5.53） | -0.83   (-1.21--0.46) | 1.27（0.26-3.58） | 5.04（1.21-12.74） | 0.07（0.02-0.17） | 1.23   (1.09-1.37) | 103.64（68.30-144.11） | 206.53（110.19-317.41） | 3.05（1.71-4.54） | -0.59   (-0.81--0.37) | 413.53（162.64-688.19） | 1012.30（409.36-1704.94） | 15.61（6.36-26.03） | 0.26   (0.04-0.49) |
| Equatorial Guinea | 73.20（45.42-107.84） | 207.28（113.73-336.49） | 48.71（27.83-77.51） | 0.27   (-0.05-0.59) | 0.17（0.08-0.35） | 1.21（0.46-2.63） | 0.27（0.10-0.58） | 3.32   (3.2-3.43) | 0.10（0.03-0.21） | 1.39（0.52-2.54） | 0.47（0.18-0.87） | 6.93   (6.82-7.03) | 6.53（0.29-15.23） | 24.65（1.00-53.59） | 5.15（0.28-10.97） | 1.01   (0.68-1.34) | 0.11（0.02-0.35） | 1.91（0.43-4.59） | 0.55（0.12-1.31） | 7.28   (7.1-7.46) | 49.39（25.70-78.99） | 102.83（52.66-170.17） | 25.79（12.44-46.01） | -0.65   (-0.96--0.33) | 16.91（5.93-28.91） | 75.29（26.23-136.52） | 16.48（5.79-28.92） | 1.83   (1.57-2.09) |
| Eritrea | 149.74（81.30-219.64） | 436.65（271.79-641.13） | 19.01（11.63-28.96） | 0.58   (0.47-0.7) | 0.23（0.09-0.49） | 1.00（0.36-2.15） | 0.04（0.02-0.09） | 1.82   (1.69-1.96) | 0.07（0.02-0.15） | 0.63（0.20-1.41） | 0.04（0.01-0.09） | 5.02   (4.91-5.12) | 2.56（-5.64-18.58） | 36.39（1.15-88.53） | 1.32（0.07-3.08） | 4.65   (4.57-4.73) | 0.04（0.01-0.12） | 0.39（0.09-1.11） | 0.02（0.01-0.06） | 4.62   (4.55-4.69) | 128.23（54.10-199.42） | 307.09（168.09-453.97） | 14.36（7.24-23.70） | 0.09   (-0.02-0.21) | 18.62（5.17-32.69） | 91.14（27.68-156.95） | 3.23（0.98-5.55） | 2.32   (2.27-2.38) |
| Estonia | 1264.53（519.98-2063.10） | 1799.42（974.92-2498.04） | 56.17（33.30-76.69） | -0.12   (-0.4-0.16) | 5.81（3.05-10.45） | 10.92（5.53-18.97） | 0.39（0.20-0.67） | 0.6   (0.24-0.95) | 7.19（3.02-12.38） | 24.66（10.31-44.69） | 0.70（0.29-1.26） | 1.75   (1.65-1.85) | 215.50（24.54-439.27） | 74.52（8.08-157.25） | 2.59（0.26-5.54） | -6.08   (-6.66--5.5) | 2.28（0.51-6.07） | 7.97（1.98-19.01） | 0.24（0.06-0.57） | 2.55   (1.78-3.33) | 132.99（97.00-165.54） | 1273.33（550.56-1845.48） | 38.87（19.96-54.60） | 8.19   (7.09-9.31) | 900.77（345.40-1485.13） | 408.01（149.78-692.82） | 13.37（5.04-22.70） | -4.4   (-4.81--3.98) |
| Eswatini | 127.58（77.91-184.38） | 368.08（210.63-572.98） | 79.55（46.79-121.87） | 1.64   (1.13-2.15) | 0.39（0.18-0.73） | 1.23（0.55-2.31） | 0.27（0.12-0.50） | 1.43   (1.18-1.68) | 0.64（0.27-1.13） | 2.23（0.93-3.78） | 0.79（0.35-1.31） | 2.58   (2.18-2.97) | 20.07（1.09-44.60） | 71.95（2.31-156.53） | 14.80（0.69-32.20） | 2.33   (1.72-2.94) | 0.25（0.05-0.80） | 1.51（0.35-4.44） | 0.34（0.08-0.96） | 3.69   (3.34-4.04) | 81.58（50.30-111.90） | 189.83（98.52-289.05） | 42.48（22.43-64.28） | 0.99   (0.53-1.44) | 24.66（9.45-42.60） | 101.33（38.69-180.86） | 20.87（8.13-37.14） | 2.78   (2.18-3.39) |
| Ethiopia | 2996.84（1850.31-4060.10） | 4260.71（2980.25-5822.41） | 10.69（7.30-14.88） | -1.99   (-2.19--1.79) | 3.16（1.34-6.35） | 12.25（5.35-23.18） | 0.03（0.01-0.06） | 1.5   (1.28-1.72) | 1.10（0.20-2.72） | 7.49（2.63-14.86） | 0.02（0.01-0.05） | 4.18   (3.99-4.38) | 51.97（-100.86-372.42） | 307.72（11.92-789.32） | 0.71（0.04-1.72） | 2.57   (2.18-2.96) | 1.69（0.41-4.99） | 11.62（2.85-31.27） | 0.03（0.01-0.09） | 3.43   (3.19-3.68) | 2414.39（1179.14-3457.09） | 2888.45（1829.40-4284.73） | 7.54（4.35-12.04） | -2.55   (-2.76--2.34) | 524.54（160.81-886.99） | 1033.18（353.95-1809.81） | 2.35（0.81-4.16） | -0.6   (-0.76--0.44) |
| Fiji | 250.55（120.57-401.30） | 582.01（278.38-934.92） | 81.65（40.55-131.64） | 0.38   (0.24-0.53) | 0.78（0.38-1.49） | 2.88（1.42-5.00） | 0.45（0.22-0.77） | 1.71   (1.5-1.92) | 0.98（0.40-1.94） | 4.77（2.17-7.97） | 1.13（0.48-1.96） | 2.84   (2.5-3.18) | 33.48（0.31-77.59） | 78.19（0.69-159.73） | 10.64（0.23-21.98） | 0.42   (0.26-0.58) | 0.14（0.04-0.31） | 0.55（0.18-1.08） | 0.10（0.03-0.21） | 2.18   (1.69-2.69) | 57.94（43.68-75.50） | 108.60（78.50-145.28） | 16.60（10.87-22.68） | -0.5   (-0.68--0.33) | 157.23（60.43-265.78） | 387.02（163.51-644.00） | 52.72（21.81-88.64） | 0.66   (0.52-0.79) |
| Finland | 2244.06（979.83-3718.22） | 2899.43（1583.65-4496.70） | 18.97（10.51-29.11） | -1.52   (-1.65--1.4) | 52.03（27.46-89.97） | 47.20（23.44-84.35） | 0.34（0.17-0.60） | -2.36   (-2.55--2.17) | 37.55（14.77-67.04） | 83.01（34.46-149.20） | 0.49（0.21-0.88） | -0.57   (-0.95--0.18) | 251.82（26.93-539.14） | 225.55（21.22-484.81） | 1.56（0.13-3.37） | -2.7   (-2.78--2.61) | 30.79（7.83-69.80） | 53.30（14.07-131.38） | 0.31（0.08-0.78） | 0.02   (-0.81-0.85) | 217.14（121.47-314.55） | 1057.15（376.52-1635.49） | 6.49（2.76-9.65） | 3.6   (2.87-4.33) | 1654.73（627.18-2738.76） | 1433.23（554.78-2461.48） | 9.78（3.87-16.40） | -2.82   (-2.89--2.75) |
| France | 10174.33（5097.37-15871.41） | 13496.74（6757.31-21290.52） | 7.24（3.79-11.64） | -1.63   (-1.76--1.51) | 191.82（103.26-324.61） | 253.10（126.64-455.37） | 0.16（0.08-0.29） | -1.65   (-1.93--1.37) | 223.15（87.13-392.15） | 906.93（371.27-1584.39） | 0.43（0.18-0.75） | 1.82   (1.64-2.01) | 1524.73（164.34-3120.79） | 1718.05（156.26-3746.28） | 0.99（0.08-2.11） | -1.82   (-1.9--1.74) | 57.50（13.49-160.47） | 203.85（46.30-493.26） | 0.10（0.02-0.24） | 1.64   (0.82-2.46) | 2383.71（892.41-3846.43） | 4384.11（1239.38-7303.41） | 2.00（0.70-3.18） | -0.86   (-1.01--0.71) | 5793.42（2154.87-9207.95） | 6030.71（2245.40-10286.71） | 3.57（1.37-5.96） | -2.24   (-2.39--2.1) |
| Gabon | 248.04（162.63-350.37） | 523.45（320.84-815.33） | 59.94（36.34-91.96） | 0.56   (0.4-0.73) | 1.15（0.54-1.98） | 3.05（1.37-5.84） | 0.33（0.15-0.63） | 1.03   (0.88-1.17) | 0.90（0.34-1.70） | 4.24（1.79-7.76） | 0.64（0.26-1.23） | 3.69   (3.55-3.83) | 22.98（1.43-53.74） | 64.86（2.81-146.63） | 6.58（0.37-14.65） | 1.32   (1.09-1.55) | 0.82（0.18-2.15） | 4.50（1.18-10.44） | 0.59（0.15-1.33） | 3.89   (3.77-4.01) | 165.73（95.37-243.14） | 287.38（162.18-451.35） | 35.02（18.98-58.78） | 0.06   (-0.1-0.22) | 56.47（20.81-94.70） | 159.42（60.98-278.21） | 16.78（6.38-29.19） | 1.4   (1.23-1.57) |
| Gambia | 73.51（45.13-107.81） | 346.19（197.93-541.38） | 37.32（21.48-58.15） | 1.54   (1.36-1.72) | 0.27（0.10-0.62） | 1.33（0.52-2.74） | 0.15（0.06-0.29） | 1.38   (1.21-1.56) | 0.14（0.04-0.28） | 1.25（0.44-2.34） | 0.20（0.07-0.39） | 3.63   (3.53-3.72) | 12.27（0.81-26.52） | 60.68（3.25-134.85） | 6.17（0.37-13.31） | 1.8   (1.64-1.97) | 0.12（0.02-0.40） | 0.99（0.21-2.92） | 0.13（0.03-0.39） | 3.39   (3.28-3.5) | 38.20（24.07-55.26） | 166.71（91.95-248.93） | 18.30（9.88-27.85） | 1.26   (1.06-1.46) | 22.51（7.61-37.94） | 115.22（41.35-200.20） | 12.37（4.36-21.48） | 1.83   (1.65-2.01) |
| Georgia | 3385.22（1411.57-5529.18） | 3143.69（1897.96-4603.21） | 50.13（30.52-73.63） | -0.15   (-0.54-0.25) | 3.62（1.88-6.25） | 17.36（8.89-30.60） | 0.29（0.15-0.51） | 7.49   (6.16-8.83) | 14.48（5.68-28.29） | 34.39（14.60-59.29） | 0.51（0.22-0.89） | 1.9   (1.04-2.76) | 528.62（22.62-1137.90） | 534.50（45.88-1144.37） | 8.71（0.70-18.68） | -0.26   (-0.57-0.05) | 0.09（0.02-0.27） | 6.36（1.69-16.81） | 0.10（0.03-0.26） | 17.08   (14.55-19.66) | 407.28（292.70-549.34） | 1526.10（775.50-2158.70） | 23.52（13.24-32.44） | 6.67   (5.37-7.99) | 2431.14（941.97-3967.31） | 1024.98（401.60-1709.83） | 17.00（6.72-28.36） | -3.66   (-4.12--3.19) |
| Germany | 45727.45（22690.27-73251.37） | 40351.46（21616.95-62483.61） | 17.25（9.39-27.04） | -2.09   (-2.18--2.01) | 427.58（225.67-735.07） | 457.95（224.55-803.83） | 0.22（0.11-0.38） | -1.51   (-1.79--1.22) | 690.87（270.99-1270.94） | 2070.82（863.94-3980.34） | 0.80（0.33-1.53） | 1.79   (1.59-1.99) | 5411.73（595.71-11125.18） | 3076.81（305.53-6736.90） | 1.44（0.13-3.11） | -3.56   (-3.78--3.33) | 326.98（86.28-874.09） | 724.91（212.06-1896.48） | 0.30（0.09-0.80） | 1.68   (0.68-2.68) | 10032.32（5080.69-14664.39） | 15952.62（4782.68-25532.23） | 6.21（2.14-9.66） | 0.49   (0.11-0.87) | 28837.97（10898.06-48582.91） | 18068.36（6903.88-30444.95） | 8.28（3.18-13.70） | -3.4   (-3.53--3.28) |
| Ghana | 1314.39（839.62-1928.19） | 5042.42（2823.04-7997.45） | 33.09（18.22-51.41） | 1.09   (0.88-1.3) | 5.52（2.28-10.74） | 25.71（10.14-52.17） | 0.18（0.07-0.36） | 1.56   (1.31-1.81) | 1.32（0.29-3.12） | 23.43（8.57-43.07） | 0.28（0.10-0.55） | 6.28   (6.02-6.55) | 177.59（12.55-412.00） | 1220.99（68.98-2682.45） | 7.42（0.48-16.15） | 3.5   (3.22-3.77) | 2.59（0.69-7.56） | 33.54（7.30-84.66） | 0.31（0.07-0.78） | 5.04   (4.89-5.19) | 728.23（444.20-1061.73） | 2344.78（1392.02-3343.67） | 15.77（9.06-23.46） | 0.55   (0.31-0.79) | 399.14（143.16-659.35） | 1393.96（502.14-2434.91） | 9.13（3.25-16.18） | 0.49   (0.13-0.85) |
| Greece | 3322.31（1438.11-5533.72） | 5483.58（2863.36-8806.98） | 19.25（10.03-30.83） | -0.65   (-0.9--0.4) | 39.91（21.31-69.41） | 87.27（44.86-151.70） | 0.35（0.18-0.60） | 0.29   (-0.09-0.68) | 30.82（11.63-56.28） | 146.66（57.03-286.84） | 0.42（0.16-0.82） | 1.85   (1.76-1.94) | 712.77（66.73-1536.60） | 761.23（66.52-1737.85） | 2.62（0.18-5.79） | -2.68   (-2.98--2.37) | 9.17（2.27-23.61） | 44.13（11.16-103.57） | 0.13（0.03-0.30） | 2.06   (1.59-2.53) | 493.72（230.29-753.03） | 1697.65（527.47-2663.64） | 4.92（1.93-7.37） | 1.76   (1.21-2.32) | 2035.92（773.07-3389.88） | 2746.64（1054.06-4621.32） | 10.81（4.27-18.03） | -0.98   (-1.27--0.68) |
| Greenland | 13.76（5.72-22.86） | 13.41（5.75-22.07） | 21.38（9.27-35.94） | -2.46   (-2.57--2.35) | 0.09（0.05-0.17） | 0.10（0.05-0.17） | 0.16（0.08-0.28） | -2.74   (-2.93--2.55) | 0.17（0.07-0.31） | 0.38（0.16-0.67） | 0.83（0.33-1.47） | 0.12   (-0.01-0.26) | 2.82（0.11-6.40） | 2.49（0.11-5.27） | 4.09（0.25-8.75） | -2.86   (-3--2.72) | 0.08（0.02-0.24） | 0.11（0.03-0.31） | 0.21（0.05-0.60） | -1.74   (-1.95--1.53) | 1.90（1.45-2.48） | 2.33（1.72-3.01） | 3.78（2.52-5.12） | -1.3   (-1.52--1.08) | 8.69（3.45-14.14） | 8.00（3.09-13.63） | 12.31（4.70-21.16） | -2.77   (-2.93--2.6) |
| Grenada | 21.05（12.01-31.18） | 36.73（23.86-52.39） | 34.69（22.20-49.81） | 0.64   (0.4-0.89) | 0.16（0.09-0.28） | 0.36（0.19-0.61） | 0.33（0.17-0.56） | 0.65   (0.05-1.26) | 0.09（0.03-0.20） | 0.40（0.16-0.73） | 0.48（0.18-0.91） | 4.79   (4.49-5.08) | 3.71（0.31-8.17） | 4.95（0.28-10.44） | 4.50（0.29-9.66） | -0.44   (-0.64--0.24) | 0.11（0.03-0.28） | 0.36（0.11-0.86） | 0.39（0.11-0.94） | 3.47   (3.31-3.63) | 8.42（5.78-11.30） | 17.83（12.76-22.50） | 17.43（11.71-23.22） | 1.86   (1.6-2.12) | 8.56（3.17-13.97） | 12.84（5.00-20.70） | 11.57（4.44-18.75） | -0.44   (-0.76--0.13) |
| Guam | 37.47（22.50-54.52） | 74.57（37.17-114.63） | 35.82（17.76-54.91） | -0.97   (-1.36--0.58) | 0.29（0.15-0.49） | 0.39（0.19-0.68） | 0.19（0.10-0.33） | -2.67   (-2.83--2.52) | 0.19（0.07-0.37） | 0.57（0.23-1.07） | 0.25（0.10-0.46） | -1.16   (-1.63--0.69) | 3.64（0.10-7.98） | 7.04（0.13-14.71） | 3.48（0.05-7.20） | -0.83   (-1.17--0.49) | 0.00（0.00-0.01） | 0.05（0.02-0.10） | 0.02（0.01-0.05） | 5.22   (2.51-8) | 14.98（9.26-19.40） | 14.47（11.06-22.20） | 6.85（5.25-10.48） | -3.91   (-4.56--3.26) | 18.37（7.47-29.87） | 52.05（21.57-84.45） | 25.03（10.45-40.48） | 0.57   (0.3-0.83) |
| Guatemala | 657.81（306.88-1073.01） | 1857.07（835.55-3134.63） | 18.87（8.73-31.27） | -0.46   (-0.88--0.04) | 0.96（0.50-1.65） | 3.99（2.01-7.02） | 0.04（0.02-0.07） | 0.09   (-0.16-0.33) | 4.74（1.98-9.32） | 35.38（15.49-65.30） | 0.42（0.18-0.77） | 1.63   (1.46-1.81) | 97.66（4.21-212.56） | 282.55（7.83-618.20） | 2.60（0.10-5.72） | -0.86   (-1.18--0.54) | 0.22（0.06-0.58） | 0.86（0.25-2.14） | 0.01（0.00-0.03） | -0.7   (-0.97--0.43) | 104.07（77.30-129.93） | 240.22（142.69-333.91） | 2.68（1.38-3.81） | -0.76   (-1.25--0.27) | 450.18（179.38-733.46） | 1294.07（511.04-2181.13） | 13.12（5.08-22.14） | -0.35   (-0.83-0.13) |
| Guinea | 569.75（351.42-841.63） | 1371.19（836.31-2008.64） | 25.48（15.42-37.28） | 1.28   (1.16-1.4) | 1.56（0.61-3.32） | 4.02（1.39-8.56） | 0.08（0.03-0.16） | 1.22   (1.11-1.33) | 1.05（0.28-2.44） | 4.58（1.50-9.55） | 0.12（0.04-0.26） | 3.5   (3.39-3.61) | 77.83（4.78-178.26） | 228.68（14.19-503.54） | 4.03（0.27-8.86） | 2.06   (1.96-2.16) | 0.76（0.14-2.49） | 3.64（0.75-11.26） | 0.08（0.02-0.26） | 3.53   (3.49-3.57) | 343.22（176.82-520.03） | 725.54（387.41-1068.47） | 13.66（7.34-20.98） | 0.77   (0.57-0.96) | 145.32（48.90-240.65） | 404.73（131.65-682.81） | 7.51（2.48-12.62） | 1.93   (1.84-2.03) |
| Guinea-Bissau | 104.63（57.02-155.26） | 278.00（159.47-416.34） | 39.82（23.71-59.02） | 1.26   (1.13-1.39) | 0.28（0.12-0.58） | 0.72（0.31-1.42） | 0.11（0.05-0.23） | 0.95   (0.84-1.06) | 0.11（0.03-0.24） | 0.52（0.20-0.95） | 0.15（0.05-0.30） | 3.97   (3.89-4.05) | 13.97（0.57-35.40） | 48.93（3.04-106.35） | 6.37（0.44-13.91） | 2.18   (2.13-2.23) | 0.09（0.02-0.29） | 0.40（0.09-1.15） | 0.09（0.02-0.25） | 3.16   (3.14-3.19) | 61.55（29.12-96.01） | 143.33（68.89-221.39） | 21.16（10.06-33.13） | 0.84   (0.66-1.02) | 28.63（9.54-51.23） | 84.10（29.99-143.23） | 11.95（4.18-20.14） | 1.63   (1.54-1.71) |
| Guyana | 240.53（158.99-339.62） | 367.21（229.31-539.85） | 60.35（38.26-88.11） | 0.2   (0-0.41) | 0.37（0.19-0.62） | 1.61（0.78-2.84） | 0.26（0.13-0.46） | 2.63   (1.98-3.29) | 0.40（0.15-0.74） | 1.84（0.77-3.29） | 0.39（0.16-0.71） | 3.76   (3.45-4.07) | 35.08（2.54-76.96） | 47.12（2.60-101.42） | 7.38（0.48-16.12） | -0.14   (-0.34-0.07) | 0.21（0.06-0.50） | 0.98（0.27-2.01） | 0.18（0.05-0.38） | 3.9   (3.5-4.3) | 126.41（95.12-162.02） | 198.92（143.86-265.50） | 33.71（23.00-46.64） | 0.43   (0.07-0.79) | 78.06（29.17-131.91） | 116.76（45.54-196.12） | 18.43（7.16-31.10） | -0.09   (-0.33-0.15) |
| Haiti | 625.71（341.54-967.02） | 1968.13（1057.96-3099.73） | 27.78（15.11-43.82） | 1.27   (1.18-1.36) | 1.38（0.58-2.90） | 5.59（2.45-11.45） | 0.09（0.04-0.18） | 2.06   (2-2.12) | 0.43（0.03-1.03） | 4.58（1.69-9.59） | 0.09（0.03-0.21） | 6.31   (6.15-6.46) | 54.49（-5.39-161.05） | 287.89（19.63-629.53） | 3.61（0.31-7.97） | 3.06   (2.94-3.19) | 0.57（0.16-1.72） | 2.74（0.76-7.87） | 0.05（0.02-0.14） | 2.35   (2.23-2.48) | 298.06（107.56-513.77） | 785.95（352.49-1290.57） | 11.87（5.03-20.65） | 0.64   (0.59-0.7) | 270.80（90.90-448.39） | 881.38（317.94-1546.39） | 12.08（4.20-21.34） | 1.5   (1.37-1.64) |
| Honduras | 471.83（282.26-719.81） | 2512.28（1388.46-3788.16） | 44.96（25.80-68.27） | 2.02   (1.82-2.22) | 0.87（0.41-1.58） | 5.73（2.51-10.91） | 0.10（0.04-0.19） | 2.56   (2.44-2.69) | 2.30（0.87-4.45） | 27.50（10.89-51.81） | 0.64（0.25-1.21） | 4.59   (4.21-4.98) | 68.26（4.89-157.13） | 396.10（17.83-837.85） | 6.44（0.34-13.81） | 2.27   (2.06-2.48) | 0.60（0.15-1.72） | 3.43（0.94-8.93） | 0.07（0.02-0.19） | 1.66   (1.5-1.82) | 201.62（141.24-296.55） | 874.90（598.30-1242.79） | 16.44（9.86-25.21） | 1.34   (1.15-1.53) | 198.17（75.12-324.13） | 1204.62（456.77-1999.45） | 21.27（7.96-35.47） | 2.52   (2.3-2.73) |
| Hungary | 9567.88（4808.75-15031.18） | 10073.44（5953.51-15083.17） | 48.57（28.75-72.82） | -1.01   (-1.14--0.88) | 56.59（30.15-94.08） | 81.15（41.23-132.96） | 0.41（0.21-0.67） | -0.24   (-0.4--0.08) | 60.96（25.45-104.29） | 117.20（52.31-202.35） | 0.53（0.24-0.91） | 0.47   (0.33-0.61) | 1737.25（165.86-3618.05） | 917.14（104.95-1842.73） | 4.57（0.49-9.25） | -3.62   (-3.81--3.42) | 166.42（41.30-404.47） | 158.44（40.83-375.20） | 0.77（0.20-1.79） | -2.71   (-3.48--1.94) | 2288.34（1478.43-3026.19） | 3598.35（1969.21-4964.83） | 16.90（9.68-22.93） | 1.03   (0.55-1.52) | 5258.32（2078.05-8598.08） | 5201.15（2068.59-8728.81） | 25.40（10.17-42.28） | -1.38   (-1.51--1.26) |
| Iceland | 72.09（29.50-123.42） | 76.63（35.09-131.05） | 11.53（5.33-19.59） | -2.39   (-2.48--2.31) | 0.98（0.49-1.73） | 1.49（0.72-2.56） | 0.24（0.12-0.41） | -1.5   (-1.81--1.19) | 1.29（0.52-2.45） | 5.33（2.04-10.01） | 0.72（0.28-1.35） | 2.19   (1.99-2.4) | 7.70（0.75-16.52） | 7.04（0.62-15.97） | 1.06（0.08-2.40） | -2.99   (-3.15--2.83) | 0.29（0.07-0.76） | 0.71（0.16-1.76） | 0.10（0.02-0.24） | 0.21   (-0.28-0.7) | 4.91（2.28-7.30） | 12.20（4.45-18.92） | 1.68（0.70-2.53） | 1.11   (0.77-1.44) | 56.91（21.86-97.44） | 49.87（18.74-86.19） | 7.74（2.99-13.03） | -3.06   (-3.13--2.98) |
| India | 29063.04（17212.21-42605.28） | 161910.58（87259.37-241010.54） | 14.10（7.78-21.10） | 2.8   (2.68-2.92) | 46.29（18.93-90.44） | 420.53（209.80-737.40） | 0.04（0.02-0.07） | 4.26   (4.12-4.39) | 26.48（7.46-55.18） | 674.84（284.28-1273.66） | 0.08（0.03-0.16） | 7.58   (7.38-7.78) | 1220.81（-309.87-3944.48） | 13717.71（1301.89-29220.34） | 1.16（0.12-2.47） | 4.55   (4.33-4.77) | 12.43（3.29-35.25） | 159.09（42.57-409.38） | 0.02（0.00-0.04） | 5.39   (5.18-5.6) | 10367.05（5646.25-15882.56） | 45930.80（29768.00-67057.33） | 4.35（2.56-6.74） | 1.89   (1.74-2.05) | 17389.97（5924.26-27536.48） | 101007.61（37297.42-164249.43） | 8.46（3.10-13.87） | 3.14   (3.01-3.27) |
| Indonesia | 8774.44（5723.35-12099.53） | 45829.84（27573.29-67711.64） | 19.63（12.14-28.61） | 2.76   (2.58-2.93) | 11.19（5.07-20.70） | 76.13（39.04-125.41） | 0.04（0.02-0.07） | 3.44   (3.38-3.51) | 2.32（-0.73-7.26） | 106.07（33.12-203.36） | 0.07（0.02-0.15） | 12.26   (11.52-13.01) | 278.57（-506.83-1764.81） | 7709.68（599.98-16619.06） | 3.01（0.28-6.44） | 7.88   (6.97-8.79) | 1.21（0.35-3.30） | 12.65（3.14-31.10） | 0.01（0.00-0.02） | 4.67   (4.52-4.82) | 5476.34（2814.59-8193.54） | 19834.34（11646.74-28219.97） | 9.29（5.22-13.91） | 1.55   (1.41-1.68) | 3004.82（965.49-4887.83） | 18090.98（6840.31-29275.00） | 7.21（2.71-11.44） | 3.43   (3.26-3.61) |
| Iran (Islamic Republic of) | 9100.45（5517.89-13043.10） | 30813.60（18213.82-44975.67） | 44.10（26.17-64.64） | 0.22   (0.13-0.31) | 3.42（1.67-5.91） | 36.58（19.52-63.12） | 0.05（0.03-0.08） | 4.79   (4.44-5.14) | 20.39（8.08-35.23） | 273.60（115.93-484.64） | 0.46（0.19-0.81） | 3.73   (3.63-3.84) | 1026.00（113.16-2030.69） | 3467.41（416.11-6897.58） | 4.81（0.60-9.64） | 0.18   (0.03-0.34) | 0.65（0.14-1.91） | 15.94（4.64-35.17） | 0.02（0.01-0.05） | 7.63   (6.94-8.33) | 3215.59（2327.62-4402.67） | 10993.90（6670.73-14833.62） | 16.48（9.17-22.89） | 0.43   (0.26-0.61) | 4834.40（1909.04-7700.67） | 16026.17（6381.29-25870.39） | 22.29（8.75-36.11） | 0.02   (-0.14-0.19) |
| Iraq | 5724.38（2769.72-9423.18） | 16063.67（7377.29-26244.76） | 79.95（39.21-128.73） | -0.29   (-0.46--0.13) | 2.39（1.10-4.20） | 11.62（5.23-20.32） | 0.05（0.02-0.08） | 1.49   (1.41-1.57) | 26.24（9.20-55.21） | 99.21（41.15-182.58） | 0.73（0.30-1.37） | 1.65   (1.44-1.86) | 1143.86（88.41-2410.15） | 3094.28（302.95-6401.84） | 14.33（1.56-30.32） | -0.61   (-0.8--0.43) | 1.71（0.46-3.79） | 6.91（2.23-14.25） | 0.04（0.01-0.08） | 1.24   (0.94-1.55) | 1562.49（859.01-2245.67） | 3614.86（2330.52-4922.86） | 20.54（11.11-28.94） | -0.66   (-0.83--0.49) | 2987.69（1141.63-4930.11） | 9236.78（3717.84-15470.94） | 44.26（17.39-74.47） | -0.01   (-0.17-0.15) |
| Ireland | 1145.82（463.72-1957.23） | 854.05（376.61-1413.48） | 10.29（4.53-17.01） | -3.35   (-3.52--3.19) | 20.71（10.60-35.72） | 22.09（10.70-38.97） | 0.26（0.13-0.46） | -2.57   (-2.98--2.15) | 12.55（5.05-22.55） | 47.03（19.47-82.91） | 0.54（0.22-0.95） | 1.65   (1.31-1.98) | 121.31（12.25-260.57） | 89.29（7.66-197.79） | 1.07（0.09-2.38） | -3.39   (-3.59--3.19) | 21.26（5.54-54.55） | 33.12（8.66-75.84） | 0.38（0.10-0.87） | -1.28   (-1.5--1.06) | 60.27（30.76-87.71） | 97.45（38.32-150.78） | 1.13（0.46-1.74） | -0.11   (-0.37-0.14) | 909.72（346.46-1499.04） | 565.07（219.36-960.18） | 6.90（2.71-11.69） | -3.97   (-4.15--3.78) |
| Israel | 1158.80（509.53-1904.43） | 998.86（462.92-1683.22） | 7.27（3.36-12.23） | -4.38   (-4.62--4.15) | 8.51（4.48-14.79） | 15.14（7.67-26.71） | 0.11（0.06-0.20） | -1.71   (-1.88--1.55) | 19.21（8.29-33.36） | 73.63（31.79-133.76） | 0.50（0.22-0.91） | 0.43   (0.25-0.61) | 116.51（8.94-243.94） | 127.68（9.46-281.82） | 0.95（0.07-2.10） | -3.7   (-3.9--3.49) | 12.29（3.35-30.19） | 35.94（10.33-84.93） | 0.25（0.07-0.59） | -0.11   (-0.83-0.61) | 116.27（63.11-167.53） | 177.19（66.91-276.73） | 1.22（0.50-1.86） | -2.52   (-3.29--1.76) | 886.01（339.34-1476.25） | 569.27（216.10-981.40） | 4.23（1.62-7.19） | -5.32   (-5.54--5.11) |
| Italy | 16725.88（8879.62-25684.55） | 28884.02（14703.39-44347.63） | 14.63（7.90-22.04） | -0.93   (-0.99--0.86) | 231.98（126.06-384.21） | 274.52（136.36-485.86） | 0.17（0.09-0.30） | -2.14   (-2.48--1.8) | 167.03（68.88-296.10） | 887.87（360.32-1680.96） | 0.41（0.17-0.77） | 2.7   (2.55-2.86) | 2458.20（266.20-5163.69） | 2429.14（234.66-5412.74） | 1.30（0.11-2.92） | -2.67   (-2.84--2.51) | 337.62（86.72-832.64） | 556.23（153.77-1210.61） | 0.26（0.07-0.58） | -1.35   (-1.64--1.07) | 5033.24（2525.09-7599.29） | 15576.89（4617.11-25733.50） | 7.32（2.57-11.66） | 0.95   (0.83-1.07) | 8497.81（3243.92-14141.66） | 9159.37（3442.34-15860.50） | 5.15（1.99-8.84） | -2.28   (-2.38--2.17) |
| Jamaica | 519.20（356.63-724.09） | 1023.19（620.25-1544.36） | 31.22（19.25-47.20） | 0.95   (0.41-1.5) | 1.62（0.84-2.79） | 4.69（2.23-8.39） | 0.15（0.07-0.27） | 1.63   (1.34-1.92) | 3.41（1.22-6.73） | 20.19（9.12-36.23） | 0.55（0.25-0.99） | 3.59   (3.41-3.77) | 78.24（5.52-165.71） | 173.95（11.85-368.24） | 5.45（0.36-11.60） | 0.98   (0.55-1.41) | 2.14（0.66-5.76） | 8.79（2.29-23.88） | 0.25（0.07-0.69） | 2.61   (2.39-2.84) | 324.98（205.57-453.39） | 574.72（361.41-810.41） | 17.39（11.35-24.15） | 0.87   (0.18-1.57) | 108.82（41.64-180.72） | 240.84（93.94-413.15） | 7.42（2.92-12.71） | 1.01   (0.55-1.47) |
| Japan | 9721.01（5582.01-14691.73） | 14790.06（7761.73-23482.27） | 3.28（1.70-5.11） | -1.95   (-2.34--1.56) | 194.80（105.44-319.33） | 993.72（484.42-1613.27） | 0.23（0.12-0.37） | 2.18   (2.12-2.25) | 29.67（8.52-66.78） | 245.68（84.11-507.91） | 0.04（0.02-0.09） | 1.89   (1.53-2.25) | 1341.27（173.05-3034.42） | 1996.32（258.31-4440.04） | 0.48（0.05-1.06） | -2.2   (-2.39--2) | 54.94（17.07-131.98） | 294.58（86.98-685.78） | 0.05（0.01-0.11） | 1.18   (0.78-1.59) | 3691.34（1741.98-5851.18） | 4835.84（1439.78-8577.87） | 0.84（0.36-1.35） | -3.14   (-4.07--2.21) | 4408.98（1550.12-7274.27） | 6423.92（2264.71-10490.22） | 1.63（0.60-2.61） | -1.6   (-1.78--1.42) |
| Jordan | 857.72（502.67-1298.23） | 3166.09（1917.43-4753.31） | 53.35（32.60-80.19） | -1.49   (-1.78--1.2) | 1.36（0.63-2.42） | 9.24（4.83-16.02） | 0.13（0.07-0.22） | 0.51   (0.31-0.71) | 2.73（1.15-4.86） | 22.05（9.70-38.83） | 0.56（0.24-1.01） | 1.47   (1.18-1.77) | 133.16（13.41-269.55） | 455.88（52.71-928.55） | 7.29（0.89-15.15） | -1.99   (-2.4--1.57) | 0.03（0.01-0.06） | 0.87（0.31-1.63） | 0.02（0.01-0.03） | 7.99   (6.99-9) | 333.96（224.67-456.78） | 1295.51（857.93-1723.27） | 24.41（13.70-34.49） | -1.04   (-1.24--0.83) | 386.48（148.44-629.69） | 1382.53（580.50-2261.84） | 20.95（8.52-34.36） | -1.88   (-2.24--1.51) |
| Kazakhstan | 5824.39（2411.34-9719.07） | 7300.74（2990.49-12496.27） | 49.66（20.37-85.63） | -1.16   (-1.9--0.42) | 15.00（7.42-26.19） | 41.54（21.53-73.82） | 0.24（0.13-0.43） | 1.42   (1.05-1.79) | 23.07（9.56-41.33） | 50.56（22.97-86.20） | 0.43（0.19-0.74） | 1.32   (1.05-1.6) | 1167.89（100.33-2510.22） | 1662.66（130.27-3552.79） | 10.76（0.92-23.10） | -0.44   (-0.95-0.08) | 1.11（0.25-2.84） | 5.10（1.35-12.89） | 0.04（0.01-0.10） | 3.01   (2.14-3.88) | 637.10（494.91-781.16） | 780.17（545.37-1031.09） | 5.25（3.34-7.14） | -1.13   (-2.81-0.58) | 3980.23（1535.58-6556.85） | 4760.72（1930.15-7863.91） | 32.94（13.07-53.93） | -1.43   (-2.06--0.79) |
| Kenya | 822.32（574.56-1109.62） | 4092.01（2652.71-5867.75） | 21.23（13.56-31.18） | 2.49   (2.33-2.66) | 3.12（1.44-5.78） | 18.79（8.28-33.11） | 0.09（0.04-0.16） | 2.44   (2.34-2.54) | 0.88（0.24-1.92） | 14.46（5.73-25.80） | 0.10（0.04-0.18） | 6.72   (6.61-6.83) | 53.72（0.57-144.92） | 552.77（30.39-1238.67） | 2.39（0.16-5.29） | 4.57   (4.34-4.81) | 1.17（0.32-3.31） | 13.52（3.01-34.75） | 0.08（0.02-0.21） | 4.81   (4.7-4.92) | 643.22（408.44-915.33） | 2509.12（1517.55-3508.49） | 14.10（7.37-21.83） | 1.9   (1.72-2.07) | 120.21（41.73-203.26） | 983.33（372.96-1687.35） | 4.46（1.65-7.77） | 3.89   (3.59-4.18) |
| Kiribati | 18.75（7.35-31.30） | 49.84（19.02-83.65） | 62.43（24.30-103.85） | 1.04   (1-1.09) | 0.01（0.00-0.02） | 0.03（0.01-0.05） | 0.04（0.02-0.07） | 1.52   (1.41-1.62) | 0.04（0.02-0.09） | 0.18（0.08-0.33） | 0.37（0.15-0.73） | 3.61   (3.53-3.68) | 4.63（-0.01-10.67） | 12.73（-0.08-27.31） | 14.96（0.10-32.46） | 1.15   (1.06-1.25) | 0.01（0.00-0.04） | 0.06（0.02-0.17） | 0.08（0.02-0.22） | 3.3   (2.88-3.73) | 3.18（2.09-4.11） | 6.15（4.03-8.34） | 9.00（5.61-12.22） | 0.09   (0.07-0.11) | 10.87（4.09-18.07） | 30.67（12.92-49.81） | 37.98（15.75-62.70） | 1.24   (1.2-1.29) |
| Kuwait | 319.42（195.02-458.91） | 1155.48（616.98-1778.58） | 41.30（22.79-63.61） | -0.95   (-1.34--0.56) | 0.62（0.33-1.07） | 3.63（1.91-6.22） | 0.12（0.06-0.21） | 1.32   (0.42-2.23) | 0.78（0.30-1.52） | 13.28（5.87-23.04） | 0.65（0.29-1.14） | 4.1   (3.51-4.69) | 21.61（1.78-42.71） | 106.06（10.80-208.38） | 3.79（0.44-7.60） | 0.49   (-0.53-1.51) | 0.07（0.02-0.17） | 1.25（0.41-2.40） | 0.06（0.02-0.11） | 8.32   (3.96-12.87) | 107.67（81.61-134.15） | 247.00（140.86-356.28） | 11.22（5.77-16.40） | -2.16   (-2.5--1.82) | 188.66（75.86-296.62） | 784.25（337.10-1255.03） | 25.46（10.82-41.35） | -0.44   (-0.87--0.02) |
| Kyrgyzstan | 1300.08（505.23-2171.86） | 2341.10（1064.33-3730.39） | 55.90（26.30-89.69） | 0.4   (0.04-0.76) | 0.62（0.31-1.05） | 3.53（1.80-6.28） | 0.08（0.04-0.14） | 4.74   (4.16-5.33) | 3.80（1.48-6.76） | 8.96（3.60-15.20） | 0.26（0.10-0.43） | 1.47   (1.29-1.66) | 305.85（21.63-657.68） | 367.00（30.57-748.96） | 7.52（0.67-15.54） | -1.87   (-2.47--1.27) | 0.97（0.19-2.78） | 3.87（0.87-9.91） | 0.10（0.02-0.25） | 2.58   (1.59-3.59) | 164.96（118.88-214.69） | 422.44（299.74-552.50） | 10.21（6.70-13.68） | 1.39   (1.02-1.75) | 823.88（317.06-1351.68） | 1535.29（610.94-2519.98） | 37.73（14.76-63.00） | 0.8   (0.37-1.23) |
| Lao People's Democratic Republic | 366.01（187.95-560.76） | 974.30（551.32-1541.50） | 21.69（12.73-34.04） | 0.53   (0.5-0.56) | 0.25（0.10-0.48） | 1.08（0.53-1.87） | 0.03（0.01-0.05） | 2.19   (2.14-2.25) | 0.15（0.01-0.42） | 2.17（0.65-4.50） | 0.07（0.02-0.15） | 6.86   (6.67-7.06) | 15.27（-13.80-68.85） | 134.41（9.19-321.84） | 2.74（0.22-6.60） | 4.03   (3.61-4.46) | 0.03（0.01-0.08） | 0.21（0.06-0.57） | 0.01（0.00-0.01） | 3.88   (3.78-3.97) | 212.44（74.41-347.56） | 432.94（246.77-623.15） | 10.27（5.73-15.24） | -0.27   (-0.33--0.21) | 137.87（45.62-235.31） | 403.48（131.82-704.88） | 8.58（2.80-15.02） | 0.87   (0.83-0.92) |
| Latvia | 2111.86（726.47-3690.71） | 2053.18（1073.69-3370.05） | 46.62（23.99-76.77） | -1.09   (-1.39--0.79) | 7.72（3.95-13.50） | 14.69（7.79-25.63） | 0.37（0.20-0.64） | 1.23   (0.89-1.56) | 12.52（5.25-23.61） | 30.87（12.85-55.35） | 0.62（0.26-1.11） | 1.8   (1.62-1.98) | 441.56（47.00-935.36） | 355.87（43.52-746.66） | 8.07（0.95-16.82） | -2.09   (-2.42--1.76) | 10.39（2.32-29.28） | 23.74（6.15-54.98） | 0.51（0.13-1.16） | 2   (1.38-2.62) | 59.54（43.72-74.30） | 529.44（272.72-733.53） | 11.67（6.73-15.63） | 8.46   (7.23-9.71) | 1580.14（597.98-2676.51） | 1098.58（426.15-1878.25） | 25.39（10.03-42.97） | -2.29   (-2.64--1.95) |
| Lebanon | 1056.46（517.65-1689.01） | 1814.39（937.88-2854.90） | 28.16（14.57-44.14） | -2.26   (-2.55--1.96) | 4.79（1.56-10.64） | 15.61（7.90-28.01） | 0.25（0.13-0.45） | 0.51   (0.18-0.84) | 6.01（2.06-12.48） | 36.70（14.92-67.74） | 0.53（0.22-0.97） | 0.73   (0.56-0.9) | 148.74（9.71-336.32） | 225.56（19.81-478.31） | 3.56（0.31-7.50） | -2.55   (-2.89--2.21) | 1.07（0.24-3.00） | 7.02（2.13-15.06） | 0.11（0.03-0.23） | 2.39   (1.99-2.8) | 281.08（106.45-469.32） | 491.85（236.04-728.79） | 7.38（3.82-10.77） | -2.77   (-2.95--2.59) | 614.77（232.16-1025.46） | 1037.66（409.75-1715.21） | 16.33（6.51-26.92） | -2.04   (-2.4--1.68) |
| Lesotho | 260.35（166.16-377.99） | 644.06（387.16-947.04） | 68.95（42.23-101.90） | 3.19   (2.65-3.74) | 0.64（0.26-1.36） | 1.40（0.54-2.95） | 0.16（0.06-0.33） | 2.22   (1.96-2.48) | 0.89（0.33-1.90） | 2.76（1.14-5.02） | 0.43（0.17-0.77） | 4.52   (3.98-5.07) | 36.33（2.45-83.60） | 126.23（4.77-278.61） | 12.47（0.59-27.02） | 4.49   (3.83-5.16) | 0.27（0.05-0.87） | 1.15（0.19-3.38） | 0.13（0.02-0.37） | 4.55   (4.2-4.9) | 188.98（119.47-268.42） | 388.81（204.21-567.44） | 43.12（21.12-64.86） | 2.57   (2.06-3.08) | 33.24（11.91-57.87） | 123.71（44.06-218.11） | 12.64（4.47-21.75） | 4.65   (4-5.31) |
| Liberia | 317.71（204.06-476.77） | 883.56（497.68-1373.50） | 43.08（24.83-67.84） | 1.26   (1.13-1.38) | 1.17（0.46-2.50） | 2.89（1.01-6.41） | 0.15（0.05-0.34） | 1.08   (0.88-1.27) | 0.64（0.21-1.21） | 3.25（1.16-6.16） | 0.26（0.09-0.51） | 4.02   (3.87-4.17) | 57.99（3.20-131.76） | 181.44（6.34-388.94） | 8.00（0.40-16.97） | 1.39   (1.25-1.52) | 0.54（0.09-1.80） | 2.40（0.44-7.00） | 0.15（0.03-0.45） | 3.42   (3.23-3.62) | 165.02（100.65-236.46） | 415.13（222.94-632.71） | 20.92（11.06-32.76） | 0.99   (0.84-1.13) | 92.36（35.19-152.42） | 278.45（106.26-470.47） | 13.59（5.03-23.60） | 1.59   (1.48-1.71) |
| Libya | 679.24（355.55-1041.54） | 3377.86（1845.88-5279.45） | 69.82（37.75-108.86） | 2.44   (2.26-2.62) | 0.17（0.07-0.34） | 2.52（0.98-5.34） | 0.05（0.02-0.10） | 6.4   (5.98-6.82) | 2.43（0.80-4.72） | 21.05（7.68-40.43） | 0.57（0.21-1.09） | 4.89   (4.63-5.16) | 75.39（6.70-164.24） | 435.81（38.94-899.80） | 8.47（0.85-17.57） | 2.75   (2.58-2.92) | 0.01（0.00-0.01） | 0.24（0.06-0.59） | 0.01（0.00-0.01） | 11.45   (10.63-12.28) | 252.78（137.15-390.91） | 1036.27（555.14-1609.54） | 23.79（12.04-37.29） | 2   (1.84-2.16) | 348.47（130.16-585.49） | 1881.96（768.87-3076.86） | 36.95（14.77-61.43） | 2.65   (2.42-2.88) |
| Lithuania | 2264.17（863.03-3891.51） | 2785.18（1325.08-4598.95） | 43.41（20.77-71.64） | -0.55   (-0.82--0.29) | 8.97（4.51-15.55） | 22.73（11.46-38.49） | 0.39（0.20-0.66） | 2.04   (1.8-2.27) | 14.28（5.87-25.36） | 47.32（20.10-84.64） | 0.66（0.28-1.16） | 2.22   (2.08-2.37) | 268.53（30.28-559.63） | 327.79（37.71-714.87） | 5.25（0.59-11.28） | -0.72   (-1.07--0.38) | 9.61（2.11-27.31） | 26.17（6.53-64.51） | 0.39（0.10-0.96） | 2.06   (1.43-2.7) | 86.14（61.89-110.30） | 409.61（228.76-560.09） | 6.41（4.07-8.41） | 4.87   (4.19-5.55) | 1876.63（736.03-3165.85） | 1951.55（774.61-3295.89） | 30.31（12.08-50.81） | -1.12   (-1.39--0.85) |
| Luxembourg | 129.11（52.95-214.43） | 132.06（67.70-212.54） | 10.95（5.59-17.82） | -2.56   (-2.67--2.44) | 1.83（0.96-3.15） | 2.34（1.19-4.03） | 0.21（0.11-0.36） | -2.04   (-2.29--1.79) | 2.41（0.97-4.27） | 9.52（3.71-16.50） | 0.73（0.28-1.26） | 1.71   (1.44-1.98) | 25.84（2.66-52.12） | 15.07（1.32-34.25） | 1.27（0.10-2.89） | -4.28   (-4.37--4.19) | 0.85（0.19-2.12） | 1.52（0.42-3.73） | 0.12（0.03-0.30） | -0.89   (-1.49--0.28) | 14.21（6.66-21.53） | 34.84（11.67-54.08） | 2.70（1.01-4.10） | 0.35   (0.12-0.59) | 83.96（31.17-140.14） | 68.77（25.74-117.74） | 5.92（2.23-10.08） | -3.33   (-3.49--3.16) |
| Madagascar | 1019.47（718.84-1457.09） | 3418.69（2114.79-4905.80） | 36.15（22.41-52.71） | 1.4   (1.32-1.47) | 2.48（1.05-5.01） | 8.23（3.30-15.80） | 0.08（0.03-0.16） | 1.36   (1.17-1.55) | 1.17（0.30-2.55） | 8.36（2.90-16.63） | 0.14（0.04-0.31） | 4.47   (4.27-4.68) | 44.04（-11.94-150.73） | 367.73（20.59-890.17） | 3.42（0.26-7.83） | 3.75   (3.66-3.84) | 0.88（0.20-2.67） | 4.62（0.96-13.58） | 0.07（0.01-0.20） | 3.35   (3.09-3.61) | 856.79（525.87-1254.78） | 2457.14（1517.38-3652.99） | 27.12（15.71-41.70） | 1.05   (0.97-1.12) | 114.10（37.71-186.07） | 572.61（207.86-1040.52） | 5.31（1.89-9.56） | 2.3   (2.16-2.43) |
| Malawi | 402.27（241.99-591.65） | 1419.04（890.96-2142.71） | 21.40（13.45-31.33） | 1.53   (1.37-1.7) | 1.31（0.51-2.85） | 5.96（2.26-12.19） | 0.09（0.03-0.18） | 2.46   (2.31-2.61) | 0.25（0.03-0.74） | 4.00（1.31-8.04） | 0.09（0.03-0.20） | 6.08   (5.93-6.23) | 10.57（-12.81-63.71） | 196.54（15.60-431.93） | 2.71（0.26-5.99） | 5.69   (5.24-6.15) | 0.32（0.06-1.03） | 3.03（0.53-9.59） | 0.06（0.01-0.19） | 4.76   (4.64-4.87) | 300.41（130.64-463.68） | 794.47（410.06-1166.48） | 12.76（6.40-19.95） | 0.75   (0.6-0.9) | 89.41（29.13-151.66） | 415.05（156.53-702.28） | 5.69（2.11-9.82） | 2.43   (2.16-2.71) |
| Malaysia | 1535.99（692.69-2426.96） | 5714.47（2359.09-9116.52） | 20.79（8.64-33.46） | 0.62   (0.49-0.76) | 9.48（4.76-15.55） | 61.22（32.24-104.62） | 0.24（0.13-0.41） | 2.41   (2.22-2.61) | 3.23（1.23-6.77） | 56.32（22.36-96.21） | 0.28（0.11-0.49） | 6.66   (6.28-7.04) | 255.73（19.59-557.17） | 974.93（55.90-2088.28） | 3.47（0.21-7.41） | 0.99   (0.87-1.1) | 0.66（0.19-1.77） | 4.84（1.44-10.96） | 0.02（0.01-0.05） | 3.08   (2.92-3.24) | 271.01（173.13-368.86） | 690.58（495.54-911.70） | 2.65（1.75-3.73） | -1.13   (-1.42--0.83) | 995.88（376.56-1569.25） | 3926.58（1522.56-6325.72） | 14.13（5.40-22.79） | 0.87   (0.74-0.99) |
| Maldives | 14.24（7.09-22.23） | 34.78（17.69-56.22） | 9.58（5.22-15.12） | -1.67   (-1.85--1.48) | 0.01（0.01-0.02） | 0.09（0.03-0.20） | 0.02（0.01-0.05） | 1.07   (0.9-1.23) | 0.01（0.00-0.02） | 0.12（0.04-0.25） | 0.04（0.01-0.10） | 6.68   (6.54-6.81) | 2.43（0.05-6.38） | 6.70（0.35-15.23） | 1.69（0.11-3.79） | -1.14   (-1.32--0.97) | 0.00（0.00-0.00） | 0.01（0.00-0.03） | 0.00（0.00-0.01） | 4.5   (4.36-4.64) | 4.16（1.42-6.66） | 9.14（6.32-13.47） | 2.93（1.86-4.46） | -1.9   (-2.12--1.69) | 7.61（2.73-12.16） | 18.72（7.15-31.30） | 4.88（1.80-8.25） | -1.72   (-1.9--1.54) |
| Mali | 598.09（339.32-877.14） | 1411.78（831.55-2111.32） | 16.68（9.90-24.95） | 0.12   (0.03-0.21) | 1.33（0.42-3.13） | 3.45（1.34-7.55） | 0.04（0.02-0.10） | 0.38   (0.11-0.64) | 0.77（0.20-1.65） | 2.78（0.88-5.36） | 0.05（0.01-0.11） | 1.52   (1.22-1.83) | 69.56（2.49-178.44） | 197.36（11.54-436.00） | 2.19（0.15-4.75） | 0.74   (0.52-0.96) | 0.79（0.11-2.65） | 4.11（0.86-12.51） | 0.06（0.01-0.19） | 2.89   (2.68-3.1) | 390.93（144.84-602.80） | 850.74（373.20-1301.54） | 10.15（4.41-15.68） | -0.16   (-0.22--0.1) | 134.71（43.79-236.36） | 353.33（117.08-605.70） | 4.19（1.38-7.19） | 0.53   (0.39-0.68) |
| Malta | 92.95（40.38-148.42） | 141.41（72.08-230.83） | 13.25（6.78-21.56） | -1.69   (-1.9--1.48) | 0.65（0.35-1.14） | 1.11（0.55-1.95） | 0.11（0.05-0.19） | -1.78   (-2.16--1.4) | 0.80（0.28-1.45） | 5.39（2.22-9.91） | 0.45（0.19-0.83） | 2.6   (2.32-2.88) | 10.46（1.09-21.53） | 11.51（1.11-25.73） | 1.09（0.09-2.39） | -2.87   (-3.17--2.57) | 0.85（0.22-2.30） | 2.72（0.72-7.04） | 0.23（0.06-0.60） | 0.17   (-0.48-0.82) | 10.46（5.75-15.05） | 30.92（12.47-47.28） | 2.69（1.17-4.03） | 0.65   (0.35-0.96) | 69.73（24.65-112.47） | 89.77（34.84-153.05） | 8.68（3.40-14.66） | -2.19   (-2.41--1.96) |
| Marshall Islands | 12.78（6.28-20.28） | 34.32（14.98-56.44） | 93.44（43.39-152.66） | 0.62   (0.51-0.72) | 0.03（0.01-0.06） | 0.09（0.04-0.18） | 0.29（0.12-0.57） | 0.81   (0.74-0.88) | 0.06（0.02-0.11） | 0.20（0.08-0.37） | 0.94（0.38-1.78） | 2.09   (1.99-2.18) | 2.66（-0.01-5.86） | 7.00（-0.10-15.25） | 17.40（0.01-38.59） | 0.57   (0.45-0.68) | 0.00（0.00-0.01） | 0.02（0.01-0.04） | 0.06（0.02-0.14） | 3.39   (3.1-3.68) | 3.38（2.10-4.80） | 6.79（4.10-9.91） | 21.05（12.69-30.30） | -0.35   (-0.44--0.25) | 6.65（2.65-10.94） | 20.22（8.31-33.25） | 53.70（21.79-89.21） | 1.07   (0.96-1.19) |
| Mauritania | 403.33（231.73-616.58） | 893.79（495.87-1409.02） | 46.37（25.48-72.47） | -0.02   (-0.12-0.09) | 1.48（0.61-2.91） | 4.12（1.44-8.73） | 0.22（0.08-0.47） | 0.55   (0.35-0.74) | 1.93（0.73-3.58） | 9.66（3.73-16.81） | 0.71（0.27-1.26） | 2.42   (2.31-2.52) | 71.77（3.44-156.19） | 162.61（8.75-361.59） | 8.08（0.50-17.81） | 0.09   (-0.07-0.25) | 0.96（0.19-3.07） | 5.65（1.20-16.31） | 0.35（0.07-1.02） | 2.96   (2.84-3.07) | 204.19（116.22-306.81） | 412.04（230.90-654.96） | 21.39（11.12-34.38） | -0.26   (-0.36--0.17) | 123.00（42.99-208.59） | 299.72（103.95-528.68） | 15.61（5.34-27.59） | 0.18   (-0.01-0.37) |
| Mauritius | 253.40（164.99-359.41） | 466.71（293.48-661.00） | 26.81（16.78-37.63） | -1.24   (-1.65--0.83) | 0.37（0.20-0.61） | 0.98（0.51-1.72） | 0.06（0.03-0.10） | 0.25   (-0.18-0.67) | 0.58（0.23-1.05） | 4.73（1.86-8.38） | 0.30（0.12-0.53） | 2.95   (2.73-3.16) | 33.94（3.01-72.15） | 56.21（3.47-118.93） | 3.16（0.19-6.72） | -2.42   (-3.02--1.83) | 0.08（0.02-0.21） | 2.60（0.79-5.97） | 0.15（0.04-0.34） | 7.97   (6.29-9.68) | 109.36（83.50-136.77） | 217.83（151.44-291.07） | 12.73（8.26-17.58） | -0.49   (-1.13-0.14) | 109.06（42.23-174.67） | 184.38（72.71-300.56） | 10.42（4.08-17.10） | -1.69   (-1.99--1.38) |
| Mexico | 7956.88（4263.39-12486.25） | 30430.91（15246.28-48705.01） | 25.52（12.93-40.91） | 0.49   (0.25-0.74) | 19.17（10.25-33.10） | 77.93（40.22-132.03） | 0.06（0.03-0.11） | 0.56   (0.39-0.72) | 82.92（32.42-159.21） | 642.24（278.82-1139.19） | 0.59（0.26-1.05） | 2.52   (2.38-2.67) | 1195.54（82.00-2545.45） | 3128.49（149.43-6386.92） | 2.49（0.13-5.08） | -0.71   (-0.84--0.57) | 45.80（14.59-108.10） | 116.48（35.61-241.48） | 0.10（0.03-0.22） | -1.61   (-1.91--1.3) | 2051.42（1258.35-2842.34） | 5058.65（2918.50-6925.77） | 4.50（2.39-6.26） | -0.77   (-1.05--0.49) | 4562.04（1792.38-7488.29） | 21407.11（8859.33-34876.63） | 17.76（7.31-29.20） | 1.15   (0.89-1.41) |
| Micronesia (Federated States of) | 40.71（20.15-67.30） | 72.26（31.20-116.83） | 93.54（42.95-148.95） | 0.47   (0.44-0.51) | 0.11（0.05-0.22） | 0.21（0.10-0.40） | 0.32（0.16-0.59） | 0.54   (0.47-0.61) | 0.18（0.07-0.34） | 0.50（0.20-0.90） | 0.96（0.38-1.79） | 2.83   (2.81-2.85) | 8.71（-0.03-19.67） | 14.76（-0.10-31.26） | 17.72（0.06-37.66） | 0.28   (0.2-0.35) | 0.02（0.01-0.05） | 0.06（0.02-0.14） | 0.08（0.02-0.18） | 2.14   (1.83-2.46) | 11.13（6.35-15.71） | 14.63（9.25-20.47） | 21.38（12.92-29.61） | -0.43   (-0.46--0.39) | 20.56（8.02-35.08） | 42.10（17.31-68.91） | 53.08（21.07-88.23） | 0.96   (0.93-0.99) |
| Monaco | 16.35（6.56-28.63） | 16.80（8.17-28.52） | 14.50（7.23-24.55） | -1.32   (-1.37--1.27) | 0.38（0.17-0.73） | 0.54（0.27-0.94） | 0.49（0.24-0.84） | -0.05   (-0.21-0.1) | 0.31（0.11-0.59） | 0.66（0.26-1.22） | 0.49（0.20-0.90） | 1.13   (0.89-1.36) | 3.75（0.37-8.28） | 2.79（0.32-6.07） | 2.45（0.26-5.31） | -2.35   (-2.5--2.19) | 0.29（0.06-0.74） | 0.56（0.15-1.22） | 0.43（0.12-0.95） | 0.78   (0.57-0.99) | 2.47（0.96-4.22） | 4.71（1.60-7.81） | 3.67（1.49-5.98） | 0.83   (0.4-1.27) | 9.15（3.38-15.92） | 7.54（2.90-12.85） | 6.97（2.70-11.67） | -2.01   (-2.08--1.93) |
| Mongolia | 416.96（167.30-708.65） | 665.12（244.16-1140.91） | 33.44（12.85-56.55） | -1.27   (-1.59--0.95) | 0.21（0.10-0.38） | 1.29（0.67-2.33） | 0.06（0.03-0.11） | 3.7   (3.36-4.04) | 0.80（0.31-1.45） | 1.98（0.80-3.57） | 0.14（0.06-0.25） | 0.57   (0.33-0.82) | 72.12（1.83-170.78） | 134.60（1.71-305.65） | 5.50（0.10-12.46） | -0.99   (-1.52--0.46) | 0.24（0.04-0.79） | 2.84（0.62-7.43） | 0.18（0.04-0.47） | 6.69   (6-7.38) | 61.15（36.12-93.20） | 82.27（50.76-124.75） | 4.49（2.40-7.17） | -1.68   (-1.92--1.43) | 282.44（107.19-478.40） | 442.14（167.33-743.45） | 23.09（8.52-39.50） | -1.29   (-1.59--1) |
| Montenegro | 239.39（97.44-405.33） | 527.48（215.26-894.66） | 59.90（25.05-101.05） | 1.37   (1.21-1.53) | 4.69（2.34-8.26） | 10.87（5.29-19.96） | 1.13（0.56-2.11） | 1.57   (1.42-1.72) | 6.95（2.88-12.42） | 21.37（8.97-37.92） | 2.72（1.14-4.89） | 2.8   (2.38-3.22) | 52.63（0.91-123.80） | 115.51（2.45-259.43） | 12.79（0.30-29.03） | 1.13   (0.91-1.35) | 0.24（0.06-0.54） | 0.55（0.16-1.19） | 0.07（0.02-0.14） | 1.63   (1.23-2.04) | 28.68（16.48-41.82） | 64.03（35.06-96.65） | 7.56（3.79-11.73） | 1.52   (1.32-1.72) | 146.21（58.05-248.07） | 315.15（132.54-530.58） | 35.64（14.69-60.15） | 1.34   (1.19-1.49) |
| Morocco | 7085.56（4032.35-10838.97） | 21503.97（12262.55-32579.00） | 68.70（39.06-103.35） | 0.95   (0.88-1.02) | 1.06（0.48-2.14） | 12.54（5.95-22.65） | 0.04（0.02-0.07） | 6.01   (5.76-6.27) | 10.96（3.82-22.82） | 89.00（35.42-167.81） | 0.36（0.14-0.69） | 4.5   (4.34-4.67) | 772.47（65.69-1770.72） | 2658.81（250.08-5690.89） | 8.07（0.81-17.45） | 1.34   (1.28-1.4) | 0.06（0.01-0.16） | 1.92（0.59-4.49） | 0.01（0.00-0.02） | 10.22   (9.56-10.88) | 2789.34（1466.75-4249.27） | 7418.12（4275.50-11151.41） | 25.18（13.48-39.38） | 0.71   (0.56-0.86) | 3511.65（1331.81-5845.01） | 11323.58（4261.30-18940.37） | 35.04（13.09-59.12） | 1.02   (0.98-1.06) |
| Mozambique | 853.18（550.16-1204.97） | 2923.45（1786.88-4335.76） | 29.80（18.16-45.40） | 2.33   (2.13-2.53) | 2.31（0.89-5.16） | 10.41（3.87-23.06） | 0.10（0.04-0.21） | 2.87   (2.8-2.93) | 0.44（0.05-1.25） | 5.77（1.77-12.21） | 0.07（0.02-0.16） | 7.71   (7.46-7.97) | 49.92（-16.31-177.66） | 541.53（31.56-1306.95） | 4.41（0.30-10.18） | 6.09   (5.87-6.3) | 0.46（0.09-1.46） | 3.88（0.86-12.32） | 0.05（0.01-0.14） | 5.05   (4.84-5.27) | 741.18（408.49-1068.72） | 2046.61（1150.50-3209.50） | 22.43（11.81-37.13） | 1.78   (1.59-1.98) | 58.87（20.23-101.61） | 315.25（109.82-545.07） | 2.76（0.95-4.76） | 3.72   (3.55-3.9) |
| Myanmar | 3914.93（2222.04-6079.09） | 7592.74（4492.76-11832.84） | 15.81（9.53-24.52） | -0.5   (-0.62--0.38) | 3.11（1.18-5.96） | 11.33（5.79-19.99） | 0.03（0.01-0.04） | 1.67   (1.64-1.7) | 1.52（0.02-4.10） | 13.86（3.70-32.46） | 0.03（0.01-0.09） | 5.7   (5.46-5.94) | 272.92（-96.09-894.69） | 1054.74（61.06-2587.37） | 2.03（0.13-4.97） | 1.62   (1.29-1.96) | 0.55（0.16-1.55） | 2.35（0.76-6.44） | 0.01（0.00-0.01） | 2.15   (2.04-2.26) | 2116.17（772.66-3417.17） | 3566.96（1747.45-5467.19） | 7.70（3.61-12.09） | -1.03   (-1.15--0.9) | 1520.66（510.04-2566.18） | 2943.50（1048.83-5090.21） | 6.02（2.13-10.32） | -0.33   (-0.47--0.19) |
| Namibia | 189.33（127.74-273.17） | 623.02（377.19-933.34） | 55.39（32.51-83.63） | 1.25   (0.91-1.6) | 0.54（0.27-1.01） | 2.24（1.03-4.20） | 0.20（0.09-0.37） | 1.86   (1.66-2.07) | 0.43（0.16-0.82） | 3.81（1.60-6.79） | 0.50（0.21-0.90） | 4.37   (4.06-4.68) | 25.36（1.78-56.73） | 101.51（4.84-218.88） | 8.47（0.49-17.81） | 1.85   (1.48-2.22) | 0.50（0.10-1.50） | 3.52（0.87-9.73） | 0.32（0.08-0.87） | 3.97   (3.72-4.21) | 123.19（80.96-174.24） | 345.77（215.53-509.32） | 32.17（18.00-49.60） | 0.79   (0.45-1.13) | 39.31（13.62-65.71） | 166.16（60.89-286.78） | 13.73（5.05-24.02） | 2.05   (1.69-2.41) |
| Nauru | 6.55（2.87-10.08） | 9.80（4.20-15.52） | 151.95（67.12-239.36） | 0.64   (0.31-0.98) | 0.02（0.01-0.04） | 0.03（0.02-0.06） | 0.68（0.33-1.20） | 1.33   (1.16-1.51) | 0.02（0.01-0.04） | 0.05（0.02-0.10） | 1.34（0.50-2.85） | 2.93   (2.81-3.05) | 1.32（-0.01-2.74） | 1.89（-0.03-3.74） | 27.08（-0.03-54.47） | 0.48   (0.04-0.94) | 0.00（0.00-0.01） | 0.01（0.00-0.01） | 0.10（0.03-0.21） | 1.86   (1.65-2.07) | 1.09（0.52-1.59） | 1.23（0.61-1.83） | 22.32（11.34-33.87） | -0.31   (-0.56--0.05) | 4.10（1.71-6.55） | 6.58（2.79-10.39） | 100.42（41.81-160.38） | 0.92   (0.58-1.25) |
| Nepal | 614.09（351.84-966.04） | 2682.76（1510.33-4182.91） | 11.67（6.70-18.21） | 2.37   (2.08-2.67) | 0.72（0.25-1.56） | 5.79（2.66-11.71） | 0.03（0.01-0.05） | 4.18   (4.03-4.33) | 0.15（-0.02-0.54） | 4.48（1.13-9.17） | 0.02（0.00-0.05） | 11.23   (11.05-11.4) | 31.09（-12.69-119.95） | 237.03（17.56-558.10） | 0.97（0.08-2.27） | 4.05   (3.9-4.2) | 0.19（0.04-0.63） | 2.37（0.68-6.24） | 0.01（0.00-0.03） | 4.87   (4.78-4.97) | 257.02（129.10-409.52） | 943.85（615.15-1382.78） | 4.48（2.77-6.94） | 1.64   (1.24-2.04) | 324.91（92.57-574.61） | 1489.25（520.19-2579.68） | 6.15（2.16-10.66） | 2.74   (2.49-2.98) |
| Netherlands | 3589.03（1430.69-6133.65） | 3109.98（1479.72-5244.12） | 7.99（3.75-13.45） | -3.01   (-3.19--2.84) | 92.45（48.82-156.54） | 113.43（56.76-189.76） | 0.29（0.15-0.49） | -2.26   (-2.72--1.8) | 75.85（28.93-135.13） | 203.20（78.35-373.58） | 0.49（0.19-0.90） | 0.54   (0.41-0.67) | 454.65（47.59-972.97） | 491.98（50.68-1072.00） | 1.28（0.12-2.75） | -2.34   (-2.59--2.08) | 76.01（18.35-198.39） | 174.54（46.42-442.98） | 0.43（0.11-1.08） | 0.39   (0.26-0.52) | 210.48（106.46-315.29） | 570.80（177.05-926.06） | 1.39（0.46-2.22） | 1.37   (1.08-1.66) | 2679.58（977.00-4489.37） | 1556.03（584.55-2643.88） | 4.12（1.55-6.92） | -4.36   (-4.57--4.15) |
| New Zealand | 1138.77（479.39-1890.23） | 1197.93（519.91-2006.94） | 13.43（5.84-22.27） | -2.74   (-2.86--2.62) | 34.70（18.49-59.41） | 32.85（16.96-57.34） | 0.37（0.19-0.64） | -3.18   (-3.34--3.02) | 21.06（8.62-36.13） | 80.49（33.04-147.20） | 0.84（0.35-1.53） | 1.46   (1.29-1.63) | 115.13（10.00-238.82） | 144.83（11.96-327.10） | 1.63（0.12-3.59） | -2.1   (-2.25--1.95) | 18.63（5.02-42.46） | 44.83（12.93-94.66） | 0.47（0.13-0.98） | -0.25   (-0.91-0.4) | 76.49（48.86-105.33） | 109.70（50.49-160.64） | 1.19（0.60-1.70） | -2.12   (-2.44--1.79) | 872.77（334.23-1442.70） | 785.22（310.49-1291.82） | 8.93（3.58-14.53） | -3.19   (-3.29--3.09) |
| Nicaragua | 282.54（152.66-435.63） | 950.54（479.18-1535.33） | 21.46（11.31-34.85） | 0.38   (0.2-0.56) | 0.36（0.18-0.63） | 1.68（0.82-2.87） | 0.04（0.02-0.06） | 1.22   (0.98-1.45) | 2.54（1.01-4.79） | 16.84（6.80-31.29） | 0.44（0.17-0.83） | 2.7   (2.42-2.97) | 46.77（2.56-103.42） | 118.55（6.17-254.06） | 2.47（0.15-5.30） | -0.59   (-0.74--0.44) | 0.12（0.03-0.34） | 0.57（0.15-1.52） | 0.01（0.00-0.04） | 1.3   (1.12-1.48) | 88.69（58.11-120.73） | 249.01（154.76-356.35） | 5.91（3.37-8.61） | -0.46   (-0.65--0.27) | 144.06（55.55-236.44） | 563.88（221.69-953.88） | 12.58（4.87-21.70） | 1.04   (0.84-1.25) |
| Niger | 347.69（195.97-513.58） | 1201.22（653.63-1823.14） | 15.70（8.75-24.00） | 0.33   (0.19-0.48) | 0.92（0.29-2.22） | 3.04（1.01-7.42） | 0.04（0.01-0.11） | 0.06   (-0.1-0.21) | 0.48（0.13-1.08） | 2.23（0.64-5.20） | 0.05（0.01-0.12） | 0.97   (0.67-1.27) | 45.53（1.72-115.35） | 180.94（8.56-404.14） | 2.20（0.13-5.02） | 0.92   (0.79-1.04) | 0.25（0.02-0.84） | 1.39（0.11-4.77） | 0.02（0.00-0.09） | 1.87   (1.65-2.08) | 223.15（76.82-358.84） | 703.96（232.33-1138.96） | 9.35（3.07-15.41） | -0.02   (-0.18-0.14) | 77.37（23.70-137.83） | 309.66（94.55-583.09） | 4.03（1.20-7.25） | 0.93   (0.79-1.06) |
| Nigeria | 8007.48（5305.19-11469.35） | 21323.94（11781.89-30951.38） | 27.66（15.36-40.37） | 0.68   (0.51-0.85) | 36.52（13.91-75.39） | 136.24（49.01-285.77） | 0.18（0.07-0.38） | 1.95   (1.82-2.08) | 32.96（11.57-62.20） | 183.35（76.21-318.66） | 0.38（0.16-0.65） | 2.92   (2.71-3.13) | 964.32（94.36-2114.03） | 3279.45（260.52-7021.09） | 4.10（0.36-8.67） | 1.38   (1.2-1.55) | 16.49（3.31-49.53） | 83.47（15.90-233.68） | 0.14（0.03-0.40） | 2.9   (2.78-3.02) | 4639.33（2829.19-7000.66） | 9083.50（5167.47-13060.77） | 11.76（6.60-17.55） | -0.51   (-0.73--0.29) | 2317.86（886.33-3861.25） | 8557.94（3102.80-14244.78） | 11.09（3.96-18.82） | 2.09   (1.96-2.22) |
| Niue | 1.31（0.66-2.06） | 1.54（0.74-2.46） | 73.04（35.42-117.48） | 0.43   (0.36-0.51) | 0.01（0.00-0.01） | 0.01（0.00-0.01） | 0.37（0.17-0.64） | 0.68   (0.55-0.8) | 0.01（0.00-0.02） | 0.02（0.01-0.03） | 0.93（0.37-1.69） | 2.83   (2.7-2.97) | 0.20（0.00-0.46） | 0.23（0.00-0.49） | 10.88（0.14-23.31） | 0.07   (-0.03-0.17) | 0.00（0.00-0.00） | 0.00（0.00-0.00） | 0.09（0.03-0.17） | 3.11   (2.77-3.45) | 0.36（0.22-0.51） | 0.30（0.20-0.42） | 14.57（9.22-20.04） | -0.47   (-0.52--0.42) | 0.73（0.28-1.22） | 0.98（0.42-1.62） | 46.20（19.85-76.73） | 0.83   (0.74-0.92) |
| North Macedonia | 1059.97（534.26-1659.47） | 1792.30（932.05-2893.81） | 71.28（36.34-114.36） | 0.3   (-0.1-0.69) | 3.10（1.56-5.45） | 8.73（3.91-16.51） | 0.28（0.13-0.52） | 1.2   (0.98-1.42) | 5.47（2.27-10.70） | 19.71（8.09-37.50） | 0.96（0.42-1.79） | 2.99   (2.39-3.6) | 257.41（23.56-530.11） | 464.30（50.06-998.29） | 17.19（1.92-36.86） | 0.21   (-0.2-0.62) | 1.20（0.28-3.15） | 3.17（0.83-7.76） | 0.14（0.04-0.35） | 1.43   (1.18-1.68) | 312.67（179.93-458.70） | 570.91（327.04-873.68） | 25.01（10.33-40.14） | 0.89   (0.46-1.32) | 480.12（192.15-808.03） | 725.48（293.13-1256.68） | 27.70（11.20-48.54） | -0.21   (-0.6-0.17) |
| Northern Mariana Islands | 8.06（2.82-14.03） | 23.76（9.85-37.97） | 46.55（19.36-75.04） | 0.89   (0.74-1.04) | 0.07（0.03-0.12） | 0.18（0.09-0.31） | 0.39（0.21-0.68） | -1.13   (-1.45--0.81) | 0.06（0.02-0.11） | 0.37（0.16-0.60） | 1.12（0.48-1.96） | 2.63   (2.34-2.92) | 2.33（-0.03-5.20） | 4.61（-0.02-9.46） | 8.79（0.08-18.24） | -0.53   (-0.75--0.31) | 0.00（0.00-0.01） | 0.04（0.01-0.08） | 0.09（0.03-0.18） | 7.3   (6.22-8.4) | 0.78（0.54-1.07） | 2.02（1.53-2.45） | 4.47（2.88-5.83） | -0.12   (-0.25-0.02) | 4.82（1.96-8.03） | 16.54（7.46-25.52） | 31.69（13.89-50.03） | 1.55   (1.38-1.72) |
| Norway | 1546.78（619.58-2590.96） | 797.58（378.32-1330.60） | 6.88（3.16-11.44） | -4.02   (-4.15--3.9) | 43.04（23.29-72.09） | 40.50（21.02-69.55） | 0.36（0.19-0.62） | -2.29   (-2.59--1.99) | 24.70（10.29-41.92） | 52.33（21.66-89.48） | 0.40（0.17-0.68） | 0.56   (0.31-0.81) | 186.22（20.82-393.42） | 95.81（8.97-209.23） | 0.83（0.07-1.81） | -3.91   (-4.07--3.74) | 42.61（11.76-101.52） | 25.69（7.22-56.11） | 0.20（0.06-0.44） | -3.82   (-4.27--3.37) | 89.18（41.98-135.86） | 116.22（40.31-191.78） | 0.90（0.34-1.45） | -0.41   (-0.97-0.16) | 1161.03（433.86-1912.36） | 467.04（176.18-784.05） | 4.18（1.58-6.99） | -4.78   (-4.92--4.64) |
| Oman | 345.75（191.07-549.47） | 1080.41（609.83-1578.02） | 66.23（39.65-96.63） | 1.2   (1-1.41) | 0.07（0.03-0.13） | 1.55（0.61-3.48） | 0.07（0.03-0.15） | 8.1   (7.59-8.62) | 0.82（0.32-1.48） | 7.47（3.20-13.41） | 0.71（0.30-1.29） | 5.22   (4.8-5.63) | 34.31（2.77-72.79） | 120.12（11.48-233.03） | 6.46（0.73-13.01） | 1.48   (1.28-1.69) | 0.02（0.00-0.05） | 0.24（0.06-0.60） | 0.02（0.00-0.03） | 6.49   (6.22-6.76) | 122.03（76.89-194.15） | 370.20（246.74-502.60） | 26.71（15.22-37.67） | 1.6   (1.1-2.1) | 188.51（65.47-326.38） | 580.83（235.22-935.01） | 32.26（12.84-53.08） | 0.8   (0.64-0.96) |
| Pakistan | 6507.23（3643.21-9958.61） | 32442.25（17622.02-50731.25） | 27.56（15.71-42.27） | 2.77   (2.56-2.99) | 13.10（5.46-24.41） | 82.93（41.72-150.93） | 0.08（0.04-0.14） | 3.62   (3.39-3.85) | 3.55（0.46-10.32） | 107.49（41.81-204.64） | 0.13（0.05-0.27） | 10.22   (9.52-10.92) | 355.21（1.82-1099.92） | 3632.87（240.61-7892.98） | 2.93（0.21-6.25） | 5.23   (4.8-5.66) | 4.35（1.08-12.10） | 31.13（8.20-82.47） | 0.03（0.01-0.09） | 4.37   (3.91-4.83) | 2699.46（1438.71-4052.35） | 8926.82（5743.03-13253.57） | 8.93（5.27-13.88） | 1.49   (1.18-1.81) | 3431.56（1248.61-5912.67） | 19661.01（7079.38-33315.45） | 15.45（5.53-25.90） | 3.31   (3.15-3.48) |
| Palau | 5.97（2.17-10.29） | 14.39（5.77-24.36） | 66.56（26.83-112.21） | 0.45   (0.37-0.53) | 0.02（0.01-0.04） | 0.06（0.03-0.11） | 0.33（0.17-0.60） | 0.87   (0.79-0.96) | 0.03（0.01-0.05） | 0.09（0.04-0.16） | 0.66（0.27-1.23） | 1.88   (1.8-1.96) | 1.33（0.02-2.88） | 2.91（0.04-6.13） | 13.01（0.33-27.04） | 0.18   (0.11-0.24) | 0.00（0.00-0.01） | 0.01（0.01-0.03） | 0.09（0.03-0.17） | 2.37   (2.06-2.68) | 0.57（0.41-0.78） | 1.09（0.79-1.44） | 5.54（3.81-7.60） | -0.36   (-0.42--0.31) | 4.02（1.57-6.54） | 10.22（4.39-16.93） | 46.93（19.44-77.99） | 0.62   (0.53-0.71) |
| Palestine | 609.87（350.31-987.32） | 1394.86（777.19-2062.61） | 70.67（38.68-104.40） | -0.49   (-0.71--0.26) | 0.38（0.18-0.71） | 1.99（1.02-3.63） | 0.08（0.04-0.15） | 2.03   (1.92-2.14) | 2.66（1.02-5.27） | 12.26（5.26-20.61） | 0.94（0.39-1.60） | 2.18   (1.98-2.38) | 88.24（8.85-191.23） | 210.18（22.58-414.44） | 10.17（1.21-20.79） | -0.35   (-0.63--0.07) | 0.02（0.01-0.07） | 0.11（0.03-0.25） | 0.01（0.00-0.01） | 1.7   (1.59-1.8) | 222.89（128.63-344.18） | 416.06（262.08-562.00） | 24.12（12.35-34.76） | -0.82   (-1.02--0.62) | 295.68（115.73-528.98） | 754.26（318.65-1198.53） | 35.35（14.70-58.45） | -0.33   (-0.56--0.09) |
| Panama | 286.81（130.02-489.54） | 878.01（439.27-1398.74） | 19.26（9.65-30.67） | -0.26   (-0.48--0.04) | 2.60（1.30-4.50） | 9.47（4.67-16.45） | 0.21（0.11-0.37） | 0.02   (-0.19-0.23) | 5.23（2.14-9.91） | 36.28（15.02-63.15） | 0.76（0.32-1.31） | 2.02   (1.89-2.15) | 49.84（3.58-111.15） | 155.89（10.55-333.48） | 3.45（0.23-7.34） | -0.34   (-0.51--0.16) | 2.94（0.74-7.52） | 11.15（2.88-27.24） | 0.24（0.06-0.58） | -0.59   (-1.03--0.15) | 44.22（28.16-58.60） | 222.21（119.40-313.66） | 4.84（2.67-6.73） | 0.96   (0.42-1.49) | 181.97（69.05-306.55） | 443.01（180.20-740.63） | 9.77（4.00-16.29） | -0.81   (-1.06--0.55) |
| Papua New Guinea | 385.45（194.13-677.19） | 1396.83（664.10-2526.38） | 23.67（11.51-41.50） | 0.63   (0.54-0.72) | 0.77（0.32-1.57） | 3.38（1.61-6.52） | 0.06（0.03-0.12） | 1.02   (0.96-1.07) | 0.52（0.15-1.15） | 3.32（1.14-6.73） | 0.06（0.02-0.12） | 2.56   (2.49-2.63) | 53.40（-0.11-160.33） | 242.86（6.75-647.21） | 3.78（0.15-10.24） | 1.28   (1.11-1.46) | 0.06（0.01-0.20） | 0.41（0.09-1.23） | 0.01（0.00-0.02） | 2.35   (2.02-2.68) | 147.70（72.91-237.91） | 397.80（216.68-669.88） | 7.40（4.09-12.49） | -0.31   (-0.35--0.28) | 183.00（63.14-337.52） | 749.05（285.97-1376.56） | 12.36（4.60-22.24） | 1.11   (0.97-1.26) |
| Paraguay | 509.48（271.43-820.35） | 1579.53（885.45-2578.22） | 28.18（16.08-45.65） | 0.78   (0.66-0.91) | 2.94（1.50-4.95） | 15.84（7.74-29.59） | 0.28（0.14-0.52） | 2.51   (2.39-2.63) | 3.36（1.29-7.12） | 27.37（10.32-56.30） | 0.53（0.20-1.09） | 4.01   (3.84-4.18) | 94.44（5.79-210.80） | 254.80（14.62-569.24） | 4.43（0.28-9.88） | 0.18   (0.08-0.28) | 0.83（0.21-2.23） | 4.32（1.12-10.86） | 0.08（0.02-0.20） | 2.4   (2.29-2.51) | 168.74（109.69-236.85） | 542.96（325.71-773.79） | 10.02（5.70-14.42） | 0.94   (0.82-1.06) | 239.17（91.05-410.08） | 734.24（282.85-1273.21） | 12.84（4.89-22.34） | 0.76   (0.56-0.96) |
| Peru | 1496.09（749.33-2473.09） | 3506.94（1688.07-5759.76） | 10.30（5.01-16.88） | -1.09   (-1.5--0.68) | 3.93（1.88-7.08） | 16.62（7.74-29.87） | 0.05（0.02-0.09） | 1.23   (1.08-1.37) | 11.83（4.38-23.82） | 91.37（35.54-166.69） | 0.27（0.11-0.50） | 2.62   (2.36-2.88) | 255.57（15.06-612.76） | 633.53（27.66-1391.07） | 1.83（0.08-4.03） | -0.95   (-1.35--0.55) | 0.69（0.17-2.08） | 5.80（1.36-14.89） | 0.02（0.00-0.04） | 3.49   (3.1-3.87) | 411.26（259.58-586.61） | 861.88（459.65-1325.90） | 2.56（1.37-3.92） | -1.41   (-1.71--1.11) | 812.82（309.21-1367.02） | 1897.74（732.33-3303.44） | 5.56（2.15-9.69） | -1.12   (-1.6--0.63) |
| Philippines | 4845.96（3437.12-6607.48） | 22022.42（13830.92-32092.00） | 27.32（17.66-39.36） | 1.66   (1.54-1.78) | 7.79（4.02-12.64） | 39.59（21.55-64.86） | 0.05（0.03-0.09） | 1.82   (1.73-1.92) | 6.07（1.54-11.56） | 79.66（31.53-150.95） | 0.14（0.05-0.28） | 5   (4.66-5.35) | 349.14（12.02-850.62） | 2569.14（159.84-5604.08） | 2.94（0.20-6.44） | 3.28   (2.92-3.65) | 1.08（0.31-3.00） | 8.14（2.08-23.24） | 0.01（0.00-0.03） | 3.05   (2.8-3.29) | 2459.33（1841.28-3168.32） | 10203.18（7271.68-13304.31） | 13.31（9.00-17.91） | 1.42   (1.3-1.54) | 2022.55（716.39-3305.37） | 9122.71（3288.12-15055.62） | 10.86（3.87-18.03） | 1.57   (1.46-1.67) |
| Poland | 21389.35（9665.43-35283.53） | 22867.35（12359.79-35756.09） | 29.87（16.33-46.60） | -2.03   (-2.17--1.9) | 175.75（90.96-299.13） | 245.03（126.65-430.34） | 0.33（0.17-0.58） | -1.16   (-1.49--0.82) | 233.74（100.54-410.49） | 500.09（211.40-898.51） | 0.62（0.26-1.11） | 0.28   (-0.27-0.83) | 3718.26（387.02-7815.29） | 2897.31（319.34-6370.60） | 3.85（0.41-8.39） | -3.17   (-3.31--3.03) | 272.10（76.18-628.39） | 837.41（264.67-1755.93） | 1.05（0.33-2.21） | 1.36   (1.15-1.57) | 2959.28（2017.81-3845.21） | 6179.62（3200.67-8565.13） | 8.00（4.43-10.94） | 0.24   (-0.04-0.52) | 14030.22（5469.27-23075.57） | 12207.90（4766.78-20457.56） | 16.03（6.26-26.68） | -2.7   (-2.84--2.56) |
| Portugal | 2745.78（1120.43-4848.08） | 3269.24（1628.90-5321.51） | 11.12（5.54-18.37） | -2.56   (-2.75--2.37) | 15.12（8.39-25.61） | 31.60（16.20-55.27） | 0.12（0.06-0.22） | -0.01   (-0.22-0.19) | 22.54（8.14-43.54） | 112.66（47.42-206.68） | 0.33（0.14-0.62） | 1.36   (1.08-1.63) | 895.33（88.49-1906.28） | 613.37（55.73-1343.61） | 2.13（0.17-4.70） | -4.42   (-4.66--4.19) | 63.60（17.62-157.32） | 162.90（43.64-369.19） | 0.49（0.13-1.11） | -0.65   (-1.03--0.27) | 419.78（212.07-628.55） | 1068.21（302.45-1749.10） | 3.19（1.07-5.06） | -0.3   (-0.47--0.13) | 1329.40（494.24-2260.22） | 1280.51（474.73-2211.01） | 4.85（1.84-8.28） | -2.98   (-3.25--2.7) |
| Puerto Rico | 1079.57（579.28-1697.43） | 1474.84（862.88-2188.39） | 18.86（11.06-28.03） | -1.83   (-2--1.65) | 6.29（3.30-11.03） | 7.86（3.93-13.51） | 0.10（0.05-0.17） | -2.55   (-2.8--2.31) | 13.32（4.99-27.13） | 51.44（21.80-93.93） | 0.52（0.22-0.94） | 0.11   (-0.08-0.29) | 89.21（5.78-192.50） | 107.42（7.23-229.64） | 1.50（0.07-3.14） | -2.4   (-2.67--2.13) | 8.07（2.26-18.46） | 37.40（11.15-79.58） | 0.41（0.12-0.86） | 1.08   (0.86-1.31) | 258.31（183.23-329.81） | 553.73（290.03-794.95） | 6.61（4.23-8.99） | -0.06   (-0.41-0.3) | 704.37（276.94-1174.56） | 716.99（291.61-1213.16） | 9.72（4.03-16.02） | -2.81   (-3.05--2.56) |
| Qatar | 65.78（30.86-105.63） | 266.35（124.76-422.94） | 40.28（20.11-63.35） | -2.82   (-3.48--2.15) | 0.13（0.06-0.24） | 1.26（0.57-2.43） | 0.13（0.06-0.25） | -0.13   (-0.6-0.33) | 0.25（0.07-0.55） | 2.27（0.87-4.42） | 0.88（0.22-1.81） | -0.1   (-0.8-0.6) | 8.01（0.32-17.17） | 38.20（1.67-79.78） | 5.01（0.48-10.67） | -2.15   (-2.83--1.47) | 0.03（0.01-0.07） | 1.00（0.30-2.03） | 0.23（0.07-0.43） | 7.37   (6.14-8.62) | 10.60（7.23-14.02） | 40.10（26.78-57.83） | 9.18（4.68-13.45） | -2.61   (-3.18--2.04) | 46.76（19.01-76.19） | 183.51（79.62-292.64） | 24.84（10.20-41.58） | -3.1   (-3.81--2.38) |
| Republic of Korea | 1737.09（1067.88-2799.57） | 4069.24（2256.74-6551.62） | 4.58（2.51-7.38） | -2.11   (-2.3--1.92) | 13.73（5.63-26.60） | 67.39（32.14-116.49） | 0.07（0.03-0.13） | 0.37   (0.14-0.6) | 1.62（0.18-4.22） | 91.42（29.29-197.64） | 0.10（0.03-0.22） | 9.27   (8.88-9.66) | 268.54（-20.62-873.65） | 700.40（78.89-1539.88） | 0.77（0.09-1.70） | -2.38   (-2.67--2.08) | 4.15（1.23-10.99） | 29.99（8.55-69.68） | 0.03（0.01-0.08） | 1.04   (0.78-1.29) | 885.03（487.18-1290.73） | 1644.28（495.30-3077.89） | 1.89（0.56-3.59） | -2.71   (-2.97--2.45) | 564.03（189.58-979.52） | 1535.75（542.08-2629.91） | 1.71（0.60-2.94） | -1.48   (-1.73--1.23) |
| Republic of Moldova | 2293.64（839.14-3835.69） | 4094.39（2248.64-6181.97） | 67.87（37.34-102.34） | -0.18   (-0.45-0.09) | 3.64（1.93-6.41） | 12.34（6.20-20.99） | 0.20（0.10-0.35） | 2.72   (2.32-3.11) | 11.19（4.86-19.51） | 34.33（14.72-58.57） | 0.56（0.24-0.95） | 0.85   (0.7-1) | 384.89（19.04-813.33） | 462.00（34.76-945.53） | 7.65（0.57-15.71） | -1.09   (-1.46--0.71) | 3.72（0.86-10.11） | 13.34（3.38-31.69） | 0.22（0.05-0.52） | 2.46   (1.92-3) | 115.87（92.42-138.58） | 1174.19（765.44-1554.43） | 19.39（12.75-25.69） | 7.24   (6.83-7.66) | 1774.32（690.36-2869.19） | 2398.18（976.18-3916.32） | 39.84（16.19-65.16） | -1.35   (-1.71--0.98) |
| Romania | 15985.74（8967.27-24435.21） | 21225.41（12098.86-32265.96） | 53.41（30.45-81.59） | -0.9   (-1.09--0.71) | 36.02（19.22-61.76） | 82.71（40.62-149.14） | 0.22（0.11-0.40） | 1.19   (0.97-1.41) | 60.24（23.97-101.82） | 186.94（78.61-319.34） | 0.44（0.19-0.75） | 0.77   (0.58-0.96) | 2879.10（236.34-6071.15） | 3330.75（309.59-7175.90） | 8.49（0.72-17.96） | -1.68   (-2.03--1.33) | 86.59（19.85-208.23） | 320.52（94.83-658.04） | 0.81（0.24-1.66） | 1.85   (1.31-2.4) | 5263.68（3559.00-6987.99） | 8389.85（4041.02-11997.33） | 20.39（10.44-28.56） | 0.44   (0.07-0.82) | 7660.10（2924.52-12496.16） | 8914.64（3430.63-14950.19） | 23.05（8.91-38.60） | -1.65   (-1.88--1.41) |
| Russian Federation | 84806.40（28547.62-145459.70） | 119568.61（47148.43-200548.71） | 50.03（19.69-84.12） | -0.61   (-1.21--0.01) | 404.73（221.75-676.65） | 1183.19（601.42-1996.70） | 0.49（0.25-0.83） | 2.16   (1.71-2.61) | 473.13（195.80-812.13） | 1585.66（671.81-2739.81） | 0.65（0.28-1.12） | 1.99   (1.79-2.19) | 20944.46（2373.03-42490.39） | 24261.34（2509.80-50329.57） | 10.20（1.03-21.16） | -1.57   (-2.18--0.95) | 965.15（213.19-2656.95） | 1235.63（317.76-2920.72） | 0.50（0.13-1.19） | -1.35   (-1.79--0.91) | 3658.81（2874.56-4376.22） | 9038.34（5878.61-11707.91） | 3.78（2.50-4.88） | 1.75   (0.44-3.07) | 58360.12（22321.94-96440.73） | 82264.44（32035.58-135655.28） | 34.41（13.44-56.98） | -0.57   (-1.14--0.01) |
| Rwanda | 551.41（211.95-801.13） | 881.08（498.85-1328.53） | 17.04（8.72-26.23） | -1.84   (-2.24--1.45) | 1.47（0.58-3.09） | 3.30（1.52-6.04） | 0.06（0.03-0.11） | -0.61   (-1.01--0.22) | 0.15（0.00-0.52） | 1.23（0.24-3.25） | 0.03（0.00-0.08） | 4.03   (3.55-4.51) | 8.98（-30.03-86.54） | 80.01（3.04-216.49） | 1.34（0.10-3.53） | 2.3   (1.82-2.78) | 0.21（0.03-0.61） | 0.96（0.11-3.24） | 0.02（0.00-0.07） | 1.92   (1.52-2.31) | 452.36（101.96-717.00） | 608.34（152.73-963.43） | 12.44（3.35-21.62） | -2.23   (-2.6--1.85) | 88.24（23.71-151.61） | 187.23（61.20-354.15） | 3.15（1.02-5.85） | -1.12   (-1.56--0.68) |
| Saint Kitts and Nevis | 15.65（8.17-24.65） | 23.38（13.56-34.80） | 38.85（22.85-58.18） | -0.06   (-0.34-0.23) | 0.08（0.04-0.14） | 0.13（0.06-0.23） | 0.22（0.11-0.38） | -0.18   (-0.41-0.06) | 0.10（0.03-0.18） | 0.33（0.13-0.59） | 0.75（0.30-1.35） | 3.3   (3.01-3.6) | 3.15（0.23-6.80） | 4.80（0.27-10.05） | 7.43（0.52-15.75） | -0.65   (-0.85--0.45) | 0.09（0.03-0.23） | 0.31（0.09-0.70） | 0.61（0.18-1.43） | 2.7   (2.54-2.86) | 4.84（3.41-6.33） | 9.74（6.87-12.71） | 17.10（10.07-23.31） | 1.81   (1.34-2.27) | 7.39（2.73-11.98） | 8.07（3.18-13.25） | 12.74（4.86-20.91） | -1.57   (-1.8--1.34) |
| Saint Lucia | 26.56（17.25-38.73） | 62.00（37.96-91.77） | 26.65（16.22-39.59） | -0.99   (-1.42--0.57) | 0.42（0.23-0.72） | 0.82（0.40-1.40） | 0.35（0.17-0.60） | -2   (-2.33--1.66) | 0.11（0.04-0.22） | 0.97（0.36-1.82） | 0.44（0.16-0.83） | 2.43   (2.16-2.71) | 4.23（0.34-8.99） | 9.66（0.64-20.84） | 4.08（0.28-8.86） | -1.29   (-1.59--0.99) | 0.20（0.06-0.48） | 1.00（0.27-2.44） | 0.45（0.12-1.08） | 0.55   (0.11-0.98) | 13.54（9.38-18.36） | 36.66（21.66-52.31） | 15.87（8.99-22.88） | -0.23   (-0.75-0.29) | 8.06（3.06-13.34） | 12.89（4.95-21.97） | 5.45（2.09-9.34） | -2.56   (-2.93--2.19) |
| Saint Vincent and the Grenadines | 20.42（14.08-29.03） | 52.85（36.33-72.92） | 40.31（26.69-56.52） | 0.86   (0.47-1.25) | 0.07（0.04-0.12） | 0.17（0.08-0.30） | 0.12（0.06-0.21） | 0.29   (-0.05-0.62) | 0.06（0.02-0.13） | 0.40（0.15-0.74） | 0.35（0.13-0.65） | 4.61   (4.38-4.84) | 1.91（0.21-4.14） | 4.87（0.32-10.39） | 3.56（0.25-7.56） | 0.75   (0.59-0.92) | 0.06（0.02-0.13） | 0.18（0.05-0.44） | 0.15（0.04-0.36） | 1.8   (1.43-2.16) | 10.84（7.63-14.61） | 34.20（23.09-46.41） | 26.32（16.30-36.80） | 1.62   (1.05-2.19) | 7.48（2.68-12.47） | 13.02（4.94-22.13） | 9.79（3.71-16.60） | -0.61   (-0.85--0.37) |
| Samoa | 55.87（27.60-86.15） | 113.20（52.40-177.67） | 78.62（36.96-123.33） | 0.62   (0.55-0.69) | 0.21（0.10-0.38） | 0.43（0.21-0.83） | 0.32（0.14-0.61） | 0.44   (0.37-0.52) | 0.32（0.12-0.60） | 1.00（0.40-1.86） | 0.87（0.33-1.70） | 2.27   (2.2-2.34) | 11.34（-0.02-23.92） | 20.91（-0.11-43.06） | 13.83（0.04-28.76） | 0.34   (0.2-0.48) | 0.03（0.01-0.06） | 0.09（0.02-0.21） | 0.07（0.02-0.15） | 2.48   (2.26-2.69) | 15.85（10.45-21.17） | 23.29（15.13-31.19） | 17.16（10.82-23.51） | -0.51   (-0.64--0.38) | 28.13（11.38-45.78） | 67.47（28.49-109.32） | 46.37（19.35-74.99） | 1.23   (1.17-1.28) |
| San Marino | 5.80（2.94-9.33） | 7.61（3.65-13.19） | 7.47（3.63-12.99） | -1.67   (-2.02--1.32) | 0.09（0.04-0.16） | 0.13（0.06-0.26） | 0.15（0.07-0.31） | -0.5   (-0.87--0.12) | 0.16（0.06-0.31） | 0.40（0.14-0.78） | 0.33（0.12-0.64） | -0.06   (-0.44-0.33) | 1.00（0.11-2.21） | 1.10（0.10-2.63） | 1.15（0.10-2.67） | -2.25   (-2.58--1.92) | 0.08（0.02-0.19） | 0.19（0.04-0.47） | 0.16（0.03-0.38） | -0.03   (-0.43-0.37) | 1.50（0.62-2.50） | 2.91（0.87-4.93） | 2.53（1.00-4.10） | -0.34   (-0.74-0.07) | 2.96（1.09-5.07） | 2.89（1.02-5.42） | 3.14（1.14-5.76） | -2.53   (-2.87--2.19) |
| Sao Tome and Principe | 10.61（6.33-16.17） | 28.57（14.14-44.86） | 27.97（14.41-44.55） | 1.46   (1.34-1.58) | 0.05（0.02-0.12） | 0.18（0.07-0.38） | 0.18（0.07-0.39） | 2.22   (2.1-2.34) | 0.04（0.01-0.08） | 0.23（0.09-0.43） | 0.35（0.13-0.67） | 5.17   (4.91-5.43) | 2.25（0.18-5.05） | 7.32（0.40-15.57） | 6.69（0.43-13.92） | 1.87   (1.7-2.03) | 0.02（0.00-0.07） | 0.16（0.03-0.45） | 0.20（0.04-0.58） | 5.13   (4.79-5.47) | 4.70（3.29-6.21） | 9.17（6.32-12.66） | 9.20（5.89-13.23） | 0.43   (0.31-0.55) | 3.55（1.36-5.90） | 11.52（4.56-19.61） | 11.36（4.39-19.47） | 2.17   (2.05-2.3) |
| Saudi Arabia | 3808.70（2061.49-6036.70） | 16354.68（8852.23-24853.22） | 88.75（50.72-132.81） | 0.58   (0.4-0.77) | 0.36（0.16-0.72） | 5.87（2.95-10.50） | 0.03（0.01-0.05） | 5.74   (5.32-6.16) | 8.96（3.67-16.29） | 55.48（24.40-94.51） | 0.70（0.30-1.22） | 3.4   (3.27-3.53) | 516.13（37.57-1126.35） | 2236.19（160.54-4397.51） | 11.11（1.10-22.77） | 0.55   (0.38-0.71) | 0.20（0.05-0.53） | 2.32（0.75-4.45） | 0.02（0.01-0.03） | 4.38   (4.2-4.55) | 1529.21（879.00-2358.38） | 4582.14（2410.52-6871.66） | 33.52（15.82-50.48） | -0.07   (-0.21-0.08) | 1753.83（659.72-2950.92） | 9472.67（4098.32-14890.53） | 43.37（18.02-68.02） | 1.16   (0.92-1.4) |
| Senegal | 667.04（423.65-974.99） | 2101.64（1274.61-3170.70） | 29.15（17.76-42.98） | 0.89   (0.82-0.97) | 2.61（1.09-5.42） | 8.42（3.00-18.74） | 0.12（0.04-0.27） | 0.71   (0.58-0.85) | 1.41（0.44-2.86） | 9.48（3.91-17.17） | 0.19（0.07-0.35） | 3.26   (3.14-3.38) | 108.29（7.97-234.55） | 358.67（19.83-739.21） | 4.70（0.30-9.72） | 1.11   (1-1.23) | 1.60（0.26-4.93） | 9.22（1.90-26.73） | 0.16（0.03-0.46） | 2.68   (2.54-2.82) | 329.01（213.22-460.74） | 1010.93（590.85-1458.41） | 14.19（8.05-20.96） | 0.88   (0.79-0.97) | 224.12（78.28-367.64） | 704.92（254.11-1183.65） | 9.79（3.50-16.72） | 0.77   (0.68-0.85) |
| Serbia | 5628.45（2754.92-9061.18） | 9172.70（4762.14-14404.96） | 53.49（27.65-83.77） | -1.16   (-1.43--0.9) | 36.75（19.33-64.85） | 80.20（39.89-135.56） | 0.47（0.24-0.80） | 0.75   (0.67-0.82) | 26.67（10.81-51.71） | 109.53（45.57-196.05） | 0.62（0.26-1.12） | 1.01   (0.65-1.37) | 1278.25（123.98-2771.15） | 1956.31（230.93-4052.99） | 11.33（1.31-23.50） | -1.36   (-1.69--1.03) | 11.92（3.32-31.46） | 32.74（9.49-73.25） | 0.19（0.05-0.42） | 0.03   (-0.37-0.43) | 1408.53（782.28-2155.89） | 2561.68（1257.55-3812.83） | 14.76（7.16-22.04） | -0.89   (-1.07--0.71) | 2866.34（1107.81-4803.67） | 4432.24（1765.90-7531.91） | 26.12（10.42-44.23） | -1.3   (-1.59--1.01) |
| Seychelles | 26.55（19.67-34.48） | 48.90（33.75-65.89） | 45.22（30.23-61.61） | -0.07   (-0.2-0.06) | 0.03（0.02-0.05） | 0.09（0.04-0.16） | 0.08（0.04-0.15） | 1.78   (1.6-1.97) | 0.05（0.02-0.11） | 0.41（0.15-0.72） | 0.46（0.17-0.84） | 5.75   (5.46-6.04) | 2.04（0.17-4.29） | 5.46（0.24-11.43） | 4.67（0.24-9.67） | 1.17   (1-1.33) | 0.01（0.00-0.02） | 0.03（0.01-0.08） | 0.03（0.01-0.07） | 2.48   (2.26-2.7) | 18.60（13.38-24.78） | 29.73（20.55-39.75） | 28.42（18.31-39.92） | -0.44   (-0.6--0.29) | 5.82（2.28-9.23） | 13.18（5.24-21.16） | 11.55（4.54-18.82） | 0.4   (0.28-0.52) |
| Sierra Leone | 325.26（199.82-479.16） | 931.01（515.75-1438.94） | 26.01（15.02-40.43） | 1.54   (1.34-1.73) | 1.61（0.68-3.51） | 3.49（1.32-8.01） | 0.10（0.04-0.23） | 0.32   (0.13-0.52) | 0.46（0.11-1.10） | 2.35（0.77-4.57） | 0.10（0.03-0.20） | 3.72   (3.58-3.85) | 41.14（3.46-96.62） | 161.93（11.49-355.00） | 4.27（0.31-9.19） | 2.55   (2.4-2.7) | 0.51（0.09-1.58） | 2.13（0.50-6.44） | 0.07（0.02-0.22） | 2.95   (2.83-3.07) | 167.93（97.45-247.93） | 423.98（224.96-642.39） | 12.06（6.27-18.47） | 1.11   (0.86-1.37) | 113.60（37.17-197.07） | 337.14（118.86-577.77） | 9.40（3.32-16.06） | 1.73   (1.57-1.89) |
| Singapore | 253.96（150.16-372.96） | 649.32（391.28-945.81） | 7.61（4.55-11.14） | -1.49   (-1.63--1.35) | 1.85（0.98-3.06） | 8.36（4.11-14.05） | 0.10（0.05-0.17） | 0.3   (0.11-0.48) | 0.12（0.03-0.28） | 3.90（1.48-7.68） | 0.05（0.02-0.09） | 6.47   (5.93-7.01) | 17.47（1.91-40.36） | 37.79（2.00-81.78） | 0.44（0.02-0.96） | -2.26   (-2.7--1.82) | 0.99（0.31-2.40） | 9.01（2.37-20.54） | 0.11（0.03-0.25） | 2.57   (2.29-2.84) | 81.10（56.95-109.36） | 232.12（150.94-316.81） | 2.73（1.75-3.78） | -1.04   (-1.44--0.65) | 152.42（54.60-246.20） | 358.15（131.14-594.27） | 4.18（1.53-6.91） | -1.8   (-2.06--1.55) |
| Slovakia | 3825.34（1618.90-6270.67） | 4047.71（1967.74-6618.12） | 42.45（20.75-69.34） | -1.36   (-1.43--1.28) | 15.67（8.04-27.39） | 26.83（13.25-46.32） | 0.28（0.14-0.49） | 0.2   (0.08-0.32) | 37.64（15.98-66.22） | 83.88（37.58-144.97） | 0.89（0.40-1.54） | 0.98   (0.87-1.08) | 575.99（49.40-1205.53） | 491.17（53.88-1025.96） | 5.09（0.54-10.63） | -2.25   (-2.35--2.16) | 13.13（3.23-28.62） | 32.62（9.59-65.18） | 0.34（0.10-0.68） | 1.32   (1.18-1.45) | 383.41（239.47-597.58） | 684.63（367.60-1023.19） | 7.18（3.83-10.75） | 0.81   (0.54-1.07) | 2799.52（1096.71-4545.43） | 2728.58（1119.52-4638.59） | 28.67（11.80-48.76） | -1.68   (-1.76--1.6) |
| Slovenia | 845.08（444.73-1330.06） | 1125.01（574.51-1733.91） | 20.60（10.74-31.47） | -1.72   (-1.81--1.63) | 7.35（3.85-12.62） | 12.45（5.97-22.20） | 0.26（0.13-0.46） | -0.68   (-1--0.36) | 9.31（3.84-16.56） | 30.54（13.18-52.65） | 0.53（0.23-0.91） | 1.26   (0.84-1.68) | 169.08（17.57-358.15） | 114.21（13.08-252.17） | 2.19（0.24-4.81） | -3.88   (-4.02--3.74) | 8.33（2.29-18.86） | 35.51（10.63-72.27） | 0.63（0.19-1.28） | 0.59   (-0.37-1.55) | 222.65（133.12-299.08） | 571.61（177.47-893.87） | 9.83（3.49-15.10） | 0.94   (0.64-1.23) | 428.36（166.76-708.04） | 360.70（139.49-614.90） | 7.16（2.79-12.08） | -3.43   (-3.62--3.24) |
| Solomon Islands | 58.08（25.96-102.66） | 200.03（82.25-346.95） | 52.99（23.12-91.39） | 0.93   (0.81-1.06) | 0.08（0.03-0.16） | 0.33（0.14-0.64） | 0.11（0.05-0.20） | 1.5   (1.36-1.64) | 0.07（0.02-0.18） | 0.51（0.17-1.07） | 0.17（0.05-0.38） | 3.74   (3.63-3.86) | 8.85（0.20-24.42） | 37.80（0.39-90.17） | 9.25（0.22-22.00） | 1.78   (1.64-1.91) | 0.01（0.00-0.03） | 0.05（0.01-0.16） | 0.02（0.00-0.05） | 2.71   (2.41-3.02) | 13.62（6.61-20.94） | 36.37（21.52-54.99） | 10.63（6.08-15.89） | 0.1   (0.05-0.15) | 35.45（12.46-63.23） | 124.98（47.09-216.01） | 32.81（12.11-57.10） | 1.02   (0.87-1.17) |
| Somalia | 441.65（240.73-664.08） | 1181.98（657.45-1786.11） | 21.28（11.74-33.02） | 0.29   (0.19-0.4) | 0.55（0.20-1.25） | 1.65（0.57-4.47） | 0.03（0.01-0.08） | 0.32   (0.21-0.43) | 0.17（0.03-0.45） | 1.10（0.26-2.77） | 0.02（0.00-0.06） | 4.16   (3.99-4.33) | 20.54（-10.55-86.47） | 127.63（1.64-338.10） | 1.76（0.06-4.60） | 3.32   (3.23-3.41) | 0.13（0.02-0.45） | 0.56（0.08-2.02） | 0.01（0.00-0.05） | 1.75   (1.58-1.92) | 362.69（158.52-572.63） | 816.62（340.35-1322.70） | 15.98（6.94-26.04） | -0.12   (-0.24-0) | 57.57（17.13-105.69） | 234.42（72.72-451.01） | 3.47（1.07-6.45） | 1.57   (1.48-1.66) |
| South Africa | 6452.43（4348.90-8997.76） | 20381.56（13388.36-28358.41） | 50.22（32.69-71.03） | 1.38   (0.93-1.83) | 48.23（24.56-80.63） | 106.71（55.80-179.94） | 0.25（0.13-0.43） | -0.76   (-1.13--0.4) | 32.90（14.06-57.95） | 184.43（84.20-308.23） | 0.60（0.27-1.00） | 3.27   (2.84-3.71) | 971.77（48.56-2125.11） | 3417.23（177.93-7210.04） | 7.88（0.49-16.64） | 1.81   (1.31-2.31) | 28.55（6.90-75.38） | 152.24（39.13-372.40） | 0.37（0.10-0.93） | 3.08   (2.8-3.36) | 3538.83（2431.24-4874.70） | 11033.56（7724.64-14190.00） | 28.15（17.06-38.09） | 1.38   (0.91-1.87) | 1832.14（734.93-2997.97） | 5487.39（2275.52-8930.88） | 12.95（5.33-21.22） | 1.07   (0.68-1.46) |
| South Sudan | 249.77（135.10-392.16） | 411.68（242.93-621.82） | 11.90（6.82-17.72） | 0.04   (-0.3-0.38) | 0.70（0.28-1.45） | 1.11（0.43-2.45） | 0.03（0.01-0.07） | 0   (-0.23-0.23) | 0.03（-0.04-0.17） | 0.27（0.02-0.73） | 0.01（0.00-0.03） | 9   (8.64-9.35) | -5.10（-19.69-18.40） | 16.69（-8.96-68.49） | 0.46（-0.15-1.62） | #VALUE! | 0.23（0.06-0.67） | 0.58（0.12-1.84） | 0.02（0.00-0.07） | 1.94   (1.75-2.13) | 213.58（99.34-343.82） | 288.19（123.31-443.89） | 8.73（3.58-13.77） | -0.5   (-0.86--0.14) | 40.33（11.61-71.90） | 104.85（31.50-191.13） | 2.64（0.79-4.70） | 1.46   (1.27-1.65) |
| Spain | 9782.76（4141.07-16853.16） | 14098.47（7283.47-22661.80） | 11.33（5.98-17.99） | -1.74   (-1.85--1.62) | 111.76（59.24-194.07） | 212.54（109.08-372.79） | 0.21（0.10-0.36） | -0.66   (-1.01--0.31) | 204.58（83.36-379.60） | 830.33（328.05-1526.38） | 0.56（0.23-1.04） | 1.02   (0.88-1.15) | 2149.20（216.62-4584.54） | 1642.03（141.02-3673.36） | 1.39（0.10-3.09） | -3.58   (-3.72--3.44) | 145.62（40.84-375.25） | 320.81（86.25-778.66） | 0.24（0.06-0.57） | -1.28   (-1.59--0.96) | 1371.00（630.89-2042.12） | 5112.59（1503.75-8053.39） | 3.52（1.26-5.37） | 1.08   (0.91-1.25) | 5800.60（2186.13-9839.06） | 5980.17（2308.40-10353.31） | 5.40（2.14-9.24） | -2.6   (-2.78--2.42) |
| Sri Lanka | 1476.40（1030.39-2041.67） | 3928.69（1963.13-6627.32） | 15.35（7.59-26.19） | 0.18   (0.03-0.32) | 0.70（0.38-1.15） | 3.72（1.71-6.84） | 0.01（0.01-0.03） | 2.66   (2.46-2.85) | 2.25（0.63-4.80） | 32.71（10.79-70.82） | 0.16（0.05-0.36） | 5.98   (5.71-6.26) | 149.40（16.47-334.69） | 623.21（50.24-1411.57） | 2.50（0.21-5.79） | 1.93   (1.68-2.17) | 0.16（0.04-0.41） | 1.37（0.36-3.75） | 0.01（0.00-0.02） | 4.4   (4.13-4.68) | 767.66（532.63-1035.52） | 1516.73（866.21-2671.16） | 5.97（3.23-10.68） | -1.11   (-1.31--0.91) | 556.23（194.58-917.94） | 1750.95（659.82-3326.21） | 6.70（2.50-12.70） | 1.03   (0.82-1.24) |
| Sudan | 5955.90（3495.12-9133.17） | 14043.73（7265.45-22196.52） | 77.61（41.81-123.47） | 0.31   (0.22-0.39) | 0.57（0.19-1.42） | 6.93（2.90-13.15） | 0.03（0.01-0.07） | 6.47   (6.2-6.73) | 9.21（3.32-18.45） | 49.04（18.14-85.95） | 0.38（0.14-0.68） | 2.99   (2.83-3.15) | 699.83（52.81-1537.92） | 1758.28（153.72-3767.55） | 8.96（0.89-18.95） | 0.36   (0.29-0.44) | 0.02（0.00-0.06） | 0.73（0.18-1.78） | 0.00（0.00-0.01） | 11.48   (10.85-12.12) | 2297.15（1175.86-3505.89） | 4671.52（2628.50-7206.80） | 28.70（15.27-44.64） | 0.04   (-0.11-0.18) | 2949.13（1125.23-5236.18） | 7557.23（3002.18-13412.20） | 39.52（15.39-70.53） | 0.49   (0.42-0.56) |
| Suriname | 63.36（37.91-94.83） | 155.86（84.36-243.73） | 24.79（13.39-38.41） | 0.09   (-0.13-0.31) | 0.23（0.12-0.40） | 0.72（0.35-1.34） | 0.12（0.06-0.22） | 0.56   (0.38-0.73) | 0.16（0.05-0.35） | 1.38（0.46-2.66） | 0.24（0.08-0.48） | 4.25   (4.08-4.41) | 6.84（0.54-15.76） | 25.77（1.43-56.50） | 4.02（0.24-8.88） | 1.23   (0.93-1.53) | 0.04（0.01-0.12） | 0.21（0.06-0.49） | 0.03（0.01-0.08） | 2.12   (1.96-2.28) | 24.71（17.30-33.59） | 62.12（40.50-90.72） | 10.12（6.49-15.11） | 0.29   (0.12-0.46) | 31.37（12.08-52.48） | 65.67（25.08-111.88） | 10.25（3.89-17.53） | -0.51   (-0.8--0.21) |
| Sweden | 3418.52（1328.78-5815.73） | 2892.94（1488.14-4638.70） | 10.80（5.53-17.26） | -2.01   (-2.1--1.92) | 90.75（47.00-154.11） | 78.21（39.08-139.27） | 0.31（0.16-0.56） | -2.45   (-2.76--2.14) | 46.09（19.08-85.76） | 227.23（90.33-423.41） | 0.76（0.31-1.41） | 3.7   (3.38-4.02) | 371.13（38.08-806.32） | 255.83（24.28-559.61） | 1.00（0.08-2.18） | -2.85   (-3.08--2.62) | 57.14（13.68-162.34） | 41.25（10.36-104.64） | 0.14（0.04-0.36） | -2.54   (-2.75--2.33) | 140.75（62.33-220.46） | 857.68（260.84-1415.63） | 2.90（0.98-4.68） | 5.53   (4.93-6.14) | 2712.65（977.91-4475.43） | 1432.74（545.20-2497.86） | 5.69（2.23-9.87） | -3.51   (-3.58--3.43) |
| Switzerland | 2143.35（1091.73-3427.27） | 2240.83（1189.25-3452.31） | 9.27（5.13-14.24） | -2.3   (-2.36--2.24) | 33.80（18.23-60.25） | 32.77（16.25-60.01） | 0.16（0.08-0.28） | -2.21   (-2.31--2.1) | 19.37（7.97-34.91） | 61.95（24.59-112.91） | 0.24（0.10-0.43） | 1.53   (1.39-1.67) | 223.38（23.78-482.74） | 150.15（15.24-333.72） | 0.66（0.06-1.50） | -3.48   (-3.59--3.36) | 26.47（7.39-68.08） | 32.35（8.47-81.30） | 0.13（0.03-0.33） | -2.05   (-2.27--1.84) | 410.00（161.76-646.99） | 1047.30（310.78-1785.23） | 3.99（1.41-6.59） | 0.86   (0.46-1.27) | 1430.33（530.13-2391.68） | 916.31（329.99-1591.90） | 4.09（1.50-6.97） | -3.94   (-4--3.87) |
| Syrian Arab Republic | 3777.21（1947.48-5774.53） | 11961.56（5701.28-19497.74） | 108.59（52.88-174.49） | 0.69   (0.5-0.88) | 1.33（0.62-2.43） | 7.07（3.44-12.77） | 0.06（0.03-0.10） | 2.42   (2.33-2.51) | 12.59（4.93-23.65） | 64.53（25.81-116.48） | 0.87（0.35-1.60） | 2.65   (2.42-2.89) | 396.80（30.52-825.16） | 1278.33（90.93-2663.81） | 10.71（0.87-22.69） | 0.55   (0.26-0.83) | 0.04（0.01-0.10） | 1.12（0.33-2.57） | 0.01（0.00-0.03） | 9.43   (8.84-10.01) | 1052.45（643.80-1461.84） | 2327.29（1409.66-3440.17） | 24.78（12.68-36.81） | -0.42   (-0.71--0.12) | 2314.00（883.85-3842.74） | 8283.22（3441.70-13938.07） | 72.15（29.39-121.23） | 1.17   (1.03-1.31) |
| Taiwan (Province of China) | 1668.18（1124.73-2363.74） | 4253.73（2587.11-6109.85） | 9.76（5.99-13.93） | -0.25   (-0.7-0.2) | 7.97（4.37-13.05） | 59.96（32.21-105.44） | 0.14（0.08-0.26） | 2.31   (1.6-3.03) | 5.81（1.86-13.73） | 108.51（43.40-198.28） | 0.23（0.09-0.42） | 3.74   (3.17-4.31) | 258.29（23.28-607.24） | 477.56（33.92-1065.16） | 1.15（0.08-2.57） | -1.47   (-1.66--1.28) | 0.19（0.05-0.49） | 2.57（0.73-6.18） | 0.01（0.00-0.01） | 6.1   (3.76-8.5) | 892.96（634.69-1187.70） | 2190.43（1051.45-3297.83） | 4.92（2.52-7.23） | -0.03   (-0.84-0.78) | 502.95（182.11-805.94） | 1414.70（537.15-2352.50） | 3.31（1.26-5.49） | -0.25   (-0.47--0.02) |
| Tajikistan | 1582.69（848.75-2411.92） | 2642.89（1330.36-4234.18） | 56.35（28.76-89.25） | -0.66   (-1.04--0.27) | 0.48（0.25-0.84） | 1.12（0.58-2.03） | 0.02（0.01-0.04） | 0.42   (0.15-0.68) | 2.08（0.82-3.83） | 4.27（1.78-7.68） | 0.12（0.05-0.21） | 0.41   (-0.02-0.83) | 206.86（13.05-437.46） | 391.10（27.30-901.81） | 7.45（0.59-16.70） | -0.55   (-0.96--0.13) | 0.63（0.12-1.67） | 1.48（0.32-3.81） | 0.04（0.01-0.09） | 0.93   (0.53-1.33) | 531.33（293.87-903.51） | 769.06（482.32-1106.18） | 17.72（9.29-26.41） | -0.91   (-1.32--0.5) | 841.31（325.39-1369.56） | 1475.85（575.91-2479.10） | 31.00（11.72-51.98） | -0.53   (-0.9--0.17) |
| Thailand | 1706.04（800.10-2777.68） | 7914.42（3249.91-13818.03） | 7.44（3.04-12.91） | 0.79   (0.5-1.07) | 11.32（5.48-19.68） | 87.24（41.40-151.65） | 0.08（0.04-0.14） | 2.16   (2.03-2.3) | 5.60（0.64-15.77） | 184.46（54.63-386.61） | 0.17（0.05-0.35） | 6.49   (6.18-6.8) | 305.99（-8.68-798.14） | 2107.86（148.55-4665.18） | 2.01（0.14-4.44） | 2.32   (1.89-2.74) | 0.24（0.06-0.66） | 3.82（0.97-9.82） | 0.00（0.00-0.01） | 4.4   (3.95-4.86) | 363.48（224.64-521.28） | 1440.93（889.56-2199.92） | 1.33（0.82-2.04） | 0.13   (-0.02-0.28) | 1019.40（337.55-1645.52） | 4090.11（1510.39-7188.15） | 3.85（1.43-6.76） | 0.29   (-0.03-0.6) |
| Timor-Leste | 15.16（7.79-23.35） | 81.38（52.11-120.22） | 9.97（6.38-14.42） | 1.85   (1.52-2.17) | 0.01（0.01-0.03） | 0.10（0.05-0.19） | 0.01（0.01-0.02） | 2.59   (2.37-2.8) | 0.00（-0.01-0.00） | 0.02（-0.01-0.08） | 0.00（0.00-0.01） | NA | -1.60（-3.61-0.82） | 3.97（-1.55-14.48） | 0.46（-0.15-1.63） | NA | 0.00（0.00-0.00） | 0.01（0.00-0.03） | 0.00（0.00-0.00） | 3.26   (3.15-3.37) | 11.87（5.18-19.52） | 46.53（28.04-70.17） | 5.81（3.43-8.97） | 0.71   (0.44-0.99) | 4.89（1.29-8.57） | 30.75（9.48-55.39） | 3.68（1.13-6.54） | 3   (2.66-3.33) |
| Togo | 234.27（149.02-345.54） | 1128.70（652.38-1698.36） | 33.24（19.05-50.18） | 1.53   (1.38-1.69) | 0.93（0.38-1.89） | 4.38（1.61-9.78） | 0.14（0.05-0.31） | 1.17   (1.04-1.3) | 0.44（0.14-0.87） | 4.13（1.61-7.73） | 0.23（0.09-0.47） | 4.28   (4.22-4.35) | 34.45（2.36-79.83） | 202.34（11.26-430.96） | 5.48（0.35-11.45） | 2.16   (2.08-2.23) | 0.35（0.07-1.15） | 3.21（0.58-10.27） | 0.14（0.02-0.46） | 3.61   (3.56-3.66) | 130.78（78.66-193.36） | 569.81（294.03-873.91） | 17.18（8.93-26.42） | 1.34   (1.15-1.52) | 67.32（24.52-109.23） | 344.83（116.66-601.11） | 10.07（3.40-17.34） | 1.53   (1.36-1.69) |
| Tokelau | 0.78（0.41-1.31） | 0.94（0.44-1.53） | 64.08（30.21-104.57） | 0.14   (0.08-0.2) | 0.00（0.00-0.00） | 0.01（0.00-0.01） | 0.35（0.17-0.62） | 1.38   (1.24-1.53) | 0.00（0.00-0.01） | 0.01（0.01-0.02） | 0.86（0.35-1.64） | 2.82   (2.67-2.96) | 0.14（0.00-0.32） | 0.15（0.00-0.34） | 10.56（0.12-23.25） | -0.22   (-0.29--0.15) | 0.00（0.00-0.00） | 0.00（0.00-0.00） | 0.08（0.02-0.17） | 2.78   (2.44-3.12) | 0.24（0.16-0.33） | 0.19（0.12-0.27） | 13.15（8.28-18.69） | -1.2   (-1.23--1.16) | 0.40（0.16-0.68） | 0.57（0.24-0.97） | 39.08（16.59-66.12） | 0.8   (0.71-0.89) |
| Tonga | 19.76（8.25-31.48） | 35.78（16.39-57.82） | 44.68（20.50-72.52） | 0.91   (0.8-1.02) | 0.12（0.06-0.21） | 0.31（0.14-0.56） | 0.39（0.18-0.71） | 1.68   (1.49-1.87) | 0.16（0.07-0.31） | 0.59（0.24-1.06） | 0.81（0.33-1.45） | 3.01   (2.81-3.2) | 3.71（0.04-7.91） | 6.09（0.08-12.23） | 7.57（0.13-15.32） | 0.67   (0.57-0.76) | 0.02（0.00-0.04） | 0.05（0.01-0.11） | 0.06（0.02-0.14） | 2   (1.58-2.43) | 2.62（1.78-3.53） | 3.98（2.73-5.36） | 5.07（3.40-6.89） | 0.06   (-0.09-0.22) | 13.13（5.52-20.78） | 24.76（11.14-39.80） | 30.78（13.72-49.78） | 1.08   (0.97-1.19) |
| Trinidad and Tobago | 381.39（233.23-545.93） | 619.30（336.45-961.04） | 32.69（17.88-50.86） | -1.85   (-2.12--1.59) | 2.89（1.57-4.87） | 7.53（3.64-13.25） | 0.39（0.19-0.69） | -0.44   (-0.82--0.06) | 2.06（0.86-3.48） | 9.89（4.27-17.44） | 0.55（0.24-0.99） | 1.34   (1.24-1.44) | 46.22（3.40-93.87） | 86.64（5.49-178.97） | 4.57（0.29-9.43） | -1.28   (-1.56--1) | 0.98（0.30-2.43） | 3.89（1.03-9.39） | 0.21（0.06-0.50） | 0.89   (0.69-1.08) | 139.34（99.24-181.40） | 204.11（135.97-286.61） | 10.90（7.07-15.42） | -2   (-2.27--1.73) | 189.90（75.06-295.39） | 307.25（118.66-508.38） | 16.07（6.19-26.63） | -2.01   (-2.31--1.71) |
| Tunisia | 1653.13（1032.78-2444.72） | 6138.00（3334.37-9622.44） | 50.66（28.02-79.79） | 0.81   (0.74-0.87) | 0.38（0.18-0.71） | 4.84（2.20-8.87） | 0.04（0.02-0.07） | 5.75   (5.46-6.05) | 3.37（1.29-6.90） | 55.51（21.34-105.31） | 0.54（0.20-1.06） | 5.65   (5.39-5.92) | 167.36（14.98-380.32） | 680.52（71.29-1487.15） | 5.44（0.59-11.94） | 1.19   (1.08-1.31) | 0.02（0.00-0.05） | 0.98（0.23-2.63） | 0.01（0.00-0.02） | 11.57   (10.89-12.26) | 701.93（441.87-1022.10） | 2471.21（1161.71-4552.36） | 21.10（9.45-39.42） | 0.68   (0.62-0.75) | 780.08（296.37-1320.88） | 2924.93（1139.33-5079.81） | 23.53（9.24-40.98） | 0.78   (0.65-0.9) |
| Turkey | 15845.77（8984.29-24064.76） | 38205.95（21113.32-58421.62） | 44.72（24.91-68.28） | -0.46   (-0.82--0.1) | 53.48（24.03-100.26） | 238.27（123.17-434.79） | 0.25（0.13-0.46） | 1.5   (1.36-1.65) | 72.78（29.44-130.91） | 459.18（190.68-800.32） | 0.60（0.25-1.05） | 2.66   (2.06-3.27) | 2299.91（162.67-4923.54） | 4892.27（398.82-10286.46） | 5.55（0.48-11.79） | -0.95   (-1.3--0.61) | 30.53（6.88-86.62） | 189.31（59.36-393.22） | 0.22（0.07-0.46） | 2.85   (2.59-3.11) | 5354.84（2828.10-7865.62） | 12263.66（6190.11-17882.91） | 15.21（6.84-22.63） | -0.6   (-1.06--0.14) | 8034.23（3123.62-12943.11） | 20163.26（7947.69-33343.62） | 22.88（8.98-38.05） | -0.32   (-0.62--0.03) |
| Turkmenistan | 1075.35（457.95-1753.37） | 2428.87（1027.38-4153.31） | 65.82（28.34-110.93） | -0.55   (-0.91--0.18) | 1.48（0.77-2.62） | 6.98（3.32-12.83） | 0.18（0.08-0.33） | 2.6   (2.43-2.76) | 1.98（0.77-3.48） | 7.98（3.09-14.73） | 0.26（0.10-0.48） | 1.53   (1.29-1.78) | 143.25（11.63-309.96） | 433.19（31.17-954.30） | 10.62（0.83-23.49） | 0.88   (0.39-1.37) | 0.40（0.09-1.16） | 1.93（0.47-5.36） | 0.06（0.01-0.16） | 2.39   (2.18-2.6) | 148.73（94.64-205.81） | 406.43（260.79-611.30） | 11.39（6.98-17.47） | 0.55   (0.31-0.8) | 779.51（304.91-1265.35） | 1572.36（608.62-2664.90） | 43.32（16.29-74.12） | -1.09   (-1.51--0.67) |
| Tuvalu | 5.17（2.51-8.27） | 8.81（4.15-13.93） | 85.21（40.58-135.30） | 0.54   (0.49-0.58) | 0.01（0.00-0.02） | 0.03（0.01-0.06） | 0.30（0.15-0.57） | 1.58   (1.48-1.69) | 0.02（0.01-0.04） | 0.06（0.03-0.11） | 0.74（0.30-1.47） | 3.27   (3.23-3.3) | 1.05（0.00-2.43） | 1.60（-0.01-3.39） | 14.79（-0.02-31.00） | 0.25   (0.17-0.33) | 0.00（0.00-0.00） | 0.00（0.00-0.01） | 0.04（0.01-0.10） | 3.44   (3.08-3.79) | 1.45（0.85-2.02） | 1.70（1.14-2.31） | 17.50（11.36-24.10） | -0.83   (-0.9--0.76) | 2.64（1.02-4.28） | 5.41（2.31-8.67） | 51.84（21.36-84.91） | 1.21   (1.16-1.26) |
| Uganda | 630.30（334.57-964.56） | 1986.36（1209.31-3051.63） | 15.02（8.77-23.72） | 0.47   (0.25-0.7) | 2.22（0.88-4.68） | 7.70（3.30-15.76） | 0.06（0.02-0.11） | 0.74   (0.55-0.94) | 0.29（0.01-0.89） | 3.88（0.97-9.01） | 0.04（0.01-0.09） | 6.63   (6.43-6.84) | 27.86（-16.33-115.04） | 217.14（12.51-521.81） | 1.41（0.10-3.44） | 2.88   (2.41-3.34) | 0.52（0.11-1.70） | 3.84（0.87-11.68） | 0.03（0.01-0.10） | 3.72   (3.62-3.82) | 465.14（138.42-775.22） | 1186.31（447.10-1943.70） | 9.71（3.49-17.74） | 0.02   (-0.17-0.21) | 134.28（45.22-236.08） | 567.50（205.39-979.74） | 3.77（1.35-6.55） | 1.13   (0.73-1.54) |
| Ukraine | 36489.04（13782.07-62000.12） | 55754.73（21966.21-94472.95） | 71.00（27.97-120.53） | 0.27   (-0.22-0.75) | 135.70（72.00-229.27） | 216.89（107.37-379.87） | 0.29（0.14-0.50） | 1   (0.71-1.3) | 232.73（101.11-386.79） | 468.62（194.32-813.29） | 0.59（0.25-1.03） | 0.82   (0.54-1.1) | 7413.06（847.21-14625.72） | 6425.77（671.90-13190.46） | 8.31（0.84-17.12） | -1.56   (-1.87--1.24) | 312.91（67.37-788.54） | 650.76（157.94-1579.54） | 0.80（0.20-1.95） | 1.41   (1.26-1.55) | 2269.88（1670.39-2889.81） | 3329.98（2029.73-4839.85） | 4.28（2.67-6.15） | 0.55   (0.27-0.83) | 26124.75（9936.08-42889.00） | 44662.71（17333.98-75888.03） | 56.73（22.00-96.36） | 0.58   (0.02-1.13) |
| United Arab Emirates | 228.50（109.95-373.97） | 1419.48（731.83-2210.86） | 63.75（35.49-94.89） | 2.01   (1.46-2.56) | 0.56（0.25-1.10） | 6.67（3.46-11.99） | 0.19（0.10-0.34） | 4   (3.31-4.68) | 0.51（0.20-0.96） | 7.02（3.11-11.67） | 0.77（0.31-1.39） | 6.69   (5.6-7.79) | 30.98（2.09-67.35） | 209.63（12.99-422.72） | 8.25（0.82-17.06） | 2.16   (1.65-2.67) | 0.47（0.12-1.35） | 8.10（2.76-14.71） | 0.50（0.16-0.96） | 7.17   (5.98-8.37) | 56.62（37.18-82.44） | 345.93（245.57-461.74） | 21.20（12.20-29.33） | 2.43   (1.75-3.12) | 139.36（52.34-228.22） | 842.13（371.07-1342.23） | 32.83（13.27-54.00） | 1.59   (1.12-2.07) |
| United Kingdom | 28906.89（11797.11-49193.26） | 17668.13（8378.58-29442.07） | 12.59（6.03-20.81） | -3.27   (-3.44--3.1) | 971.07（515.48-1616.39） | 534.01（272.22-948.41） | 0.36（0.19-0.64） | -4.1   (-4.45--3.75) | 365.54（144.54-687.59） | 1053.12（418.50-1954.63） | 0.65（0.26-1.20） | 1.76   (1.66-1.86) | 3576.55（384.34-7578.81） | 2123.99（165.49-4621.22） | 1.49（0.10-3.24） | -3.31   (-3.51--3.12) | 326.68（81.20-957.81） | 435.92（115.53-1176.44） | 0.28（0.07-0.74） | -1.2   (-1.47--0.92) | 1520.19（990.54-2020.24） | 2665.03（1392.69-3698.15） | 1.86（1.10-2.48） | 0.96   (0.78-1.13) | 22146.86（8548.44-36978.85） | 10856.07（4237.52-18250.02） | 7.96（3.12-13.20） | -4.02   (-4.2--3.84) |
| United Republic of Tanzania | 1894.27（1136.70-2674.85） | 6645.55（4005.09-10075.73） | 30.28（18.43-46.13） | 1.23   (1.18-1.29) | 6.18（2.73-11.97） | 30.26（12.75-62.93） | 0.13（0.05-0.27） | 2   (1.86-2.14) | 3.30（0.94-6.95） | 32.99（11.41-63.76） | 0.19（0.07-0.38） | 4.63   (4.48-4.78) | 148.03（3.32-379.18） | 847.72（46.87-1899.47） | 3.55（0.22-7.85） | 2.93   (2.72-3.14) | 1.97（0.35-6.70） | 17.09（2.51-50.84） | 0.09（0.01-0.28） | 3.81   (3.64-3.98) | 1404.37（616.79-2122.77） | 3699.12（1539.67-5731.58） | 18.12（7.00-29.89） | 0.41   (0.35-0.48) | 330.42（113.66-566.41） | 2018.37（727.16-3700.81） | 8.20（2.99-15.03） | 3.02   (2.87-3.17) |
| United States of America | 99181.77（47397.00-162300.23） | 152511.93（87452.64-228828.32） | 25.66（14.80-38.01） | -0.86   (-1--0.72) | 1474.67（766.82-2548.15） | 1295.54（670.20-2208.05） | 0.22（0.12-0.38） | -3.06   (-3.38--2.74) | 1118.61（448.67-2165.25） | 5278.12（2227.72-9081.77） | 0.79（0.34-1.36） | 2.7   (2.55-2.85) | 7687.56（614.48-16601.88） | 13886.38（923.21-28511.67） | 2.35（0.14-4.79） | -0.48   (-0.7--0.27) | 1294.61（359.28-3066.74） | 3098.64（979.93-6409.74） | 0.49（0.16-0.99） | -0.13   (-0.53-0.28) | 13299.11（8979.27-17356.04） | 43430.80（27558.67-56335.19） | 7.44（5.13-9.40） | 2.09   (1.94-2.24) | 74307.22（29007.12-124380.69） | 85522.45（34851.23-140224.76） | 14.36（5.90-23.35） | -1.92   (-2.07--1.77) |
| United States Virgin Islands | 38.57（21.23-59.39） | 51.47（28.18-78.98） | 30.75（16.77-47.32） | -1.57   (-1.72--1.43) | 0.20（0.10-0.35） | 0.36（0.18-0.62） | 0.20（0.10-0.34） | -1.05   (-1.24--0.86) | 0.38（0.16-0.72） | 1.07（0.44-1.95） | 0.71（0.29-1.30） | -0.21   (-0.41-0) | 4.06（0.17-8.80） | 4.50（0.30-9.82） | 2.75（0.16-5.92） | -1.86   (-2.02--1.69) | 0.20（0.06-0.52） | 0.77（0.22-1.62） | 0.47（0.13-1.01） | 1.05   (0.89-1.22) | 11.77（8.45-15.80） | 16.44（9.72-24.11） | 9.75（5.48-14.34） | -1.39   (-1.55--1.23) | 21.96（8.81-36.57） | 28.31（11.80-47.78） | 16.87（7.07-28.53） | -1.74   (-1.89--1.58) |
| Uruguay | 1083.95（505.37-1779.01） | 1219.26（666.74-1861.79） | 19.90（10.95-30.38） | -1.27   (-1.38--1.16) | 17.05（8.89-29.33） | 23.89（12.34-41.45） | 0.42（0.22-0.70） | -0.52   (-0.78--0.26) | 8.25（3.52-14.83） | 30.38（12.51-55.64） | 0.42（0.17-0.77） | 2.18   (2-2.35) | 215.28（17.01-464.93） | 200.23（16.50-439.15） | 3.43（0.25-7.36） | -1.95   (-2.17--1.74) | 0.28（0.07-0.75） | 0.71（0.19-1.87） | 0.01（0.00-0.03） | 1.01   (0.49-1.54) | 176.94（113.27-238.33） | 435.73（189.38-643.32） | 6.47（3.34-9.17） | 1.05   (0.92-1.17) | 666.15（258.42-1104.43） | 528.31（207.18-863.31） | 9.15（3.61-15.02） | -2.17   (-2.33--2.01) |
| Uzbekistan | 5478.09（2345.24-8932.20） | 14770.17（6907.26-23809.64） | 65.65（31.00-107.66） | 0.8   (0.45-1.15) | 1.67（0.82-3.09） | 22.46（11.62-42.04） | 0.10（0.05-0.18） | 6.82   (6.06-7.59) | 7.06（2.38-16.00） | 27.38（11.24-50.47） | 0.15（0.06-0.28） | 2.56   (2.37-2.76) | 810.19（55.08-1741.44） | 1743.88（141.26-3704.65） | 7.05（0.63-15.28） | -0.64   (-1.04--0.24) | 0.36（0.07-1.05） | 3.23（0.87-8.62） | 0.02（0.00-0.04） | 4.82   (3.95-5.69) | 786.16（499.21-1154.91） | 2524.73（1715.67-3550.58） | 11.34（7.19-16.28） | 1.69   (1.44-1.94) | 3872.64（1497.09-6378.14） | 10448.50（4114.47-17460.57） | 47.00（18.23-79.91） | 0.86   (0.47-1.25) |
| Vanuatu | 28.27（13.59-50.29） | 108.48（47.55-179.85） | 58.45（26.88-97.04） | 0.9   (0.84-0.97) | 0.07（0.03-0.13） | 0.28（0.13-0.51） | 0.18（0.08-0.33） | 1   (0.89-1.1) | 0.04（0.01-0.11） | 0.32（0.12-0.61） | 0.24（0.09-0.49） | 3.4   (3.35-3.45) | 3.65（0.14-9.72） | 16.02（0.27-36.88） | 8.02（0.24-18.53） | 1.29   (1.2-1.38) | 0.01（0.00-0.02） | 0.04（0.01-0.11） | 0.02（0.00-0.06） | 2.16   (1.81-2.51) | 6.77（4.08-10.14） | 20.83（14.09-28.54） | 12.44（7.97-17.10） | 0.18   (0.13-0.24) | 17.73（6.38-32.16） | 70.99（29.03-119.95） | 37.55（14.98-64.72） | 1.09   (1.02-1.15) |
| Venezuela (Bolivarian Republic of) | 3755.49（2289.99-5642.34） | 12010.17（6351.14-18993.37） | 41.27（21.74-65.00） | -0.43   (-0.72--0.14) | 17.54（9.28-30.37） | 60.94（27.98-109.82） | 0.21（0.10-0.38） | -0.24   (-0.53-0.05) | 22.99（9.55-45.39） | 159.18（65.83-303.98） | 0.60（0.25-1.15） | 2   (1.87-2.13) | 365.98（15.48-825.07） | 1275.66（56.06-2805.89） | 4.32（0.21-9.49） | -0.09   (-0.37-0.2) | 5.25（1.34-14.21） | 18.32（4.67-47.60） | 0.07（0.02-0.17） | -0.58   (-0.94--0.21) | 1380.40（987.46-1788.44） | 3354.05（1952.70-4864.43） | 11.96（6.53-17.62） | -1.3   (-1.63--0.98) | 1963.32（767.22-3277.00） | 7142.03（2851.11-12270.36） | 24.12（9.57-41.46） | 0   (-0.28-0.29) |
| Viet Nam | 1704.93（1071.40-2690.06） | 7319.69（4424.23-10881.77） | 8.02（4.71-11.99） | 2.29   (2.02-2.57) | 2.66（1.23-5.05） | 20.44（10.05-35.90） | 0.02（0.01-0.04） | 4.22   (4.08-4.37) | -0.16（-1.42-1.18） | 24.99（4.38-65.43） | 0.03（0.01-0.10） | NA | -90.81（-324.32-163.67） | 1090.55（51.14-2698.02） | 1.14（0.08-2.84） | NA | 0.30（0.10-0.77） | 2.58（0.72-6.34） | 0.00（0.00-0.01） | 4.32   (4.22-4.42) | 1452.86（795.51-2405.44） | 4144.68（2207.97-6677.66） | 4.69（2.41-7.93） | 0.87   (0.66-1.07) | 340.08（96.63-581.25） | 2036.45（676.38-3440.11） | 2.14（0.69-3.71） | 3.66   (3.33-3.99) |
| Yemen | 2085.78（1248.43-3086.30） | 8610.35（4739.51-13218.37） | 68.95（39.36-107.54） | 1.04   (0.85-1.23) | 0.17（0.06-0.36） | 3.05（1.26-5.90） | 0.02（0.01-0.04） | 6.84   (6.52-7.16) | 1.72（0.49-3.82） | 23.60（9.16-44.89） | 0.29（0.10-0.57） | 5.25   (4.92-5.59) | 190.52（18.12-426.13） | 1066.74（81.38-2181.24） | 7.81（0.64-16.21） | 2.07   (1.87-2.27) | 0.01（0.00-0.02） | 0.40（0.09-1.10） | 0.00（0.00-0.01） | 12.18   (11.53-12.83) | 1022.58（494.74-1568.38） | 3613.85（1866.50-6084.72） | 31.93（15.18-54.51） | 0.56   (0.37-0.74) | 870.79（303.41-1538.52） | 3902.71（1316.14-6610.23） | 28.90（9.57-49.44） | 1.38   (1.18-1.57) |
| Zambia | 549.55（380.94-746.59） | 2236.89（1303.55-3367.06） | 37.11（21.87-55.10） | 1.5   (1.37-1.63) | 1.24（0.56-2.43） | 10.31（3.37-23.36） | 0.15（0.05-0.33） | 3.85   (3.34-4.36) | 0.39（0.07-0.96） | 9.24（3.14-20.94） | 0.19（0.06-0.43） | 7.77   (7.53-8) | 25.55（-6.26-91.44） | 341.03（14.71-771.72） | 4.77（0.26-10.93） | 5.01   (4.68-5.34) | 0.66（0.18-1.82） | 8.44（1.48-24.34） | 0.16（0.03-0.43） | 5.61   (5.18-6.04) | 454.03（266.99-637.44） | 1356.23（694.32-1993.80） | 24.44（12.11-37.63） | 0.67   (0.54-0.8) | 67.68（21.61-116.13） | 511.64（194.63-918.35） | 7.40（2.77-13.15） | 3.57   (3.44-3.69) |
| Zimbabwe | 615.49（417.64-834.11） | 2728.71（1614.07-4153.28） | 44.40（27.27-66.29） | 3.83   (3.22-4.45) | 3.65（1.90-6.04） | 17.09（7.86-33.44） | 0.29（0.13-0.55） | 3.39   (3.13-3.64) | 1.27（0.41-2.33） | 10.15（4.00-18.85） | 0.24（0.09-0.44） | 5.81   (5.31-6.32) | 65.03（5.31-145.38） | 506.91（15.23-1118.41） | 7.42（0.29-16.38） | 6.01   (5.11-6.91) | 0.08（0.02-0.22） | 0.42（0.10-1.22） | 0.01（0.00-0.02） | 3.53   (3.29-3.77) | 409.83（285.63-577.90） | 1405.26（878.69-2087.78） | 23.98（14.67-36.18） | 3   (2.47-3.54) | 135.64（50.54-227.94） | 788.89（292.26-1335.60） | 12.47（4.49-20.88） | 4.74   (4.02-5.46) |

CVD, cardiovascular disease; HBMI, high body mass index; EAPC, estimated annual percentage change; ASMR, age-standardized mortality rate.

Table S6. Change in DALYs of CVD attributable to HBMI, decomposed by three population-level determinants: population aging, population growth, and epidemiological change from 1990 to 2021 at the global level and five SDI regions

| Variables | Overll difference | Population Aging | Population Growth | Epidemiological change |
| --- | --- | --- | --- | --- |
| CVD |  |  |  |  |
| High-middle SDI | 4764879.39 | 2997997.51  (62.92%) | 3298749.74  (69.23%) | -1531867.86  (-32.15%) |
| Global | 24344222.1 | 7618810.57  (31.3%) | 16944387.4  (69.6%) | -218975.85  (-0.9%) |
| High SDI | 1419569.43 | 1979125.07  (139.42%) | 2184205.13  (153.86%) | -2743760.77  (-193.28%) |
| Middle SDI | 9716892.55 | 3059709.29  (31.49%) | 4850536.09  (49.92%) | 1806647.17  (18.59%) |
| Low-middle SDI | 6565449.7 | 733923.17  (11.18%) | 3690551.08  (56.21%) | 2140975.45  (32.61%) |
| Low SDI | 1862067.19 | -78788.55  (-4.23%) | 1473222.93  (79.12%) | 467632.81  (25.11%) |
| Hypertensive heart disease | |  |  |  |
| Global | 6885735.24 | 2230842.35  (32.4%) | 4604307.73  (66.87%) | 50585.16  (0.73%) |
| High SDI | 1029823.49 | 401908.37  (39.03%) | 412269.77  (40.03%) | 215645.36  (20.94%) |
| High-middle SDI | 1092467.39 | 646347.29  (59.16%) | 618273.81  (56.59%) | -172153.71  (-15.76%) |
| Middle SDI | 2276473.73 | 1227407.34  (53.92%) | 1714249.06  (75.3%) | -665182.67  (-29.22%) |
| Low-middle SDI | 1710351.14 | 287022.49  (16.78%) | 1247245.35  (72.92%) | 176083.31  (10.3%) |
| Low SDI | 767812.28 | -41449.68  (-5.4%) | 771335.05  (100.46%) | 37926.9  (4.94%) |
| Ischemic heart disease | |  |  |  |
| Global | 11999481.6 | 3997868.38  (33.32%) | 9245468.41  (77.05%) | -1243855.17  (-10.37%) |
| High SDI | -189973.93 | 1197981.47  (-630.6%) | 1360340.27  (-716.07%) | -2748295.67  (1446.67%) |
| High-middle SDI | 2486466.95 | 1687846.85  (67.88%) | 1922475.75  (77.32%) | -1123855.65  (-45.2%) |
| Middle SDI | 5207190.55 | 1352944.46  (25.98%) | 2335419.13  (44.85%) | 1518826.96  (29.17%) |
| Low-middle SDI | 3730848.3 | 345734.18  (9.27%) | 1927963.88  (51.68%) | 1457150.24  (39.06%) |
| Low SDI | 761141.35 | -27225.12  (-3.58%) | 523000.11  (68.71%) | 265366.36  (34.86%) |
| Stroke |  |  |  |  |
| Global | 4625736.03 | 1129378.47  (24.42%) | 2679152.02  (57.92%) | 817205.55  (17.67%) |
| High SDI | 272817.33 | 235959.48  (86.49%) | 291904.01  (107%) | -255046.16  (-93.49%) |
| High-middle SDI | 969127.8 | 570221.21  (58.84%) | 674013.19  (69.55%) | -275106.6  (-28.39%) |
| Middle SDI | 2024216.22 | 426581.01  (21.07%) | 736842.29  (36.4%) | 860792.92  (42.52%) |
| Low-middle SDI | 1042388.34 | 91888.12  (8.82%) | 485159.48  (46.54%) | 465340.74  (44.64%) |
| Low SDI | 315500.32 | -9569.67  (-3.03%) | 169883.47  (53.85%) | 155186.53  (49.19%) |
| Atrial fibrillation and flutter | |  |  |  |
| Global | 549541.98 | 101114.61  (18.4%) | 245667.5  (44.7%) | 202759.86  (36.9%) |
| High SDI | 228407.7 | 62040.26  (27.16%) | 74935.36  (32.81%) | 91432.07  (40.03%) |
| High-middle SDI | 128426.39 | 32723.06  (25.48%) | 53544.71  (41.69%) | 42158.62  (32.83%) |
| Middle SDI | 134071.28 | 21770.97  (16.24%) | 46993.62  (35.05%) | 65306.69  (48.71%) |
| Low-middle SDI | 49678.57 | 4070.81  (8.19%) | 18966.58  (38.18%) | 26641.18  (53.63%) |
| Low SDI | 8359.33 | -177.46  (-2.12%) | 3672.82  (43.94%) | 4863.97  (58.19%) |
| Lower extremity peripheral arterial disease | | |  |  |
| Global | 173607.5 | 31888.34  (18.37%) | 146302.45  (84.27%) | -4583.29  (-2.64%) |
| High SDI | 73234.66 | 21627.17  (29.53%) | 48048.29  (65.61%) | 3559.2  (4.86%) |
| High-middle SDI | 45775.03 | 11753.66  (25.68%) | 43951.78  (96.02%) | -9930.41  (-21.69%) |
| Middle SDI | 36197.44 | 3954.25  (10.92%) | 19170.23  (52.96%) | 13072.95  (36.12%) |
| Low-middle SDI | 13947.08 | 773.8  (5.55%) | 6268.29  (44.94%) | 6904.99  (49.51%) |
| Low SDI | 4113.83 | -99.17  (-2.41%) | 2063.62  (50.16%) | 2149.38  (52.25%) |
| Aortic aneurysm |  |  |  |  |
| Global | 110119.75 | 38877.7  (35.3%) | 111395.92  (101.16%) | -40153.88  (-36.46%) |
| High SDI | 5260.19 | 23944.99  (455.21%) | 32298.35  (614.02%) | -50983.15  (-969.23%) |
| High-middle SDI | 42615.83 | 12247.72  (28.74%) | 23367.84  (54.83%) | 7000.27  (16.43%) |
| Middle SDI | 38743.34 | 6858.28  (17.7%) | 17286.49  (44.62%) | 14598.57  (37.68%) |
| Low-middle SDI | 18236.26 | 1240.18  (6.8%) | 7865.45  (43.13%) | 9130.63  (50.07%) |
| Low SDI | 5140.08 | -176.64  (-3.44%) | 3187.45  (62.01%) | 2129.27  (41.42%) |

Cardiovascular disease (CVD); High body mass index (HBMI); DALYs, disability-adjusted life years; SDI, socio-demographic index.
